# Identification of a novel chemotherapy benefit index for patients with advanced ovarian cancer based on Bayesian network analysis

**DOI:** 10.1371/journal.pone.0322130

**Published:** 2025-05-27

**Authors:** Shuxiao Ma, Lu Zhou, Yi Liu, Hui Jie, Min Yi, Chenglin Guo, Jiandong Mei, Chuan Li, Lei Zhu, Senyi Deng

**Affiliations:** Department of Thoracic Surgery and Institute of Thoracic Oncology, West China Hospital, Sichuan University, Chengdu, China; University of Mauritius, MAURITIUS

## Abstract

**Background:**

This study aims to evaluate the efficacy of chemotherapy and optimize treatment strategies for patients with advanced ovarian cancer.

**Methods:**

Based on The Cancer Genome Atlas (TCGA) transcriptome data, we conducted correlation and Bayesian network analyses to identify key genes strongly associated with chemotherapy prognosis. Reverse Transcription Quantitative Polymerase Chain Reaction (RT-qPCR) was used to verify the expression of these key genes. The Chemotherapy Benefit Index (CBI) was developed using these genes via multivariable Cox regression analysis, and validated using both internal and external validation sets (GSE32062 and GSE30161) with a random forest model. Subsequently, we analyzed distinct molecular characteristics and explored additional immunotherapy in CBI-high and CBI-low subgroups.

**Results:**

Based on the network and machine learning analyses, CBI was developed from the following ten genes: *COL6A3*, *SPI1*, *HSF1*, *CD3E*, *PIK3R4*, *MZB1*, *FERMT3*, *GZMA*, *PSMB9* and *RSF1*. Significant differences in overall survival were observed among the CBI-high, medium, and low subgroups (P < 0.001), which were consistent with the two external validation sets (P < 0.001 and P = 0.003). The AUC of internal validation and two external validation cohorts were 0.87, 0.71 and 0.70, respectively. Molecular function analysis indicated that the CBI-low subgroup is characterized by the activation of cancer-related signaling pathways, immune-related biological processes, higher *TP53* mutation rate, particularly with a better response to immune checkpoint blockade (ICB) treatment, while the CBI-high subgroup is characterized by inhibition of cell cycle, less response to ICB treatment, and potential therapeutic targets.

**Conclusions:**

This study provided a novel CBI for patients with advanced ovarian cancer through network analyses and machine learning. CBI could serve as a prognostic prediction tool for patients with advanced ovarian cancer, and also as a potential indicator for immunotherapy.

## 1. Introduction

Ovarian cancer is a gynecologic malignancy with an unfavorable prognosis and high fatality worldwide [1]. Due to the paucity of effective screening methods and “silent symptoms”, only 20% of ovarian cancer patients are diagnosed at an early stage [2]. Furthermore, the five-year overall survival rate for patients diagnosed with advanced ovarian cancer falls below 40% [3]. As a result, ovarian cancer is considered to be one of the most intricate tumors to manage [4]. With the unrelenting advancement of medical technology, cancer therapy has evolved into a multidisciplinary approach that incorporates surgery, chemotherapy, radiotherapy, and immunotherapy [4,5]. Past studies have reported an improvement in the prognosis of advanced ovarian cancer patients who received multidisciplinary treatment [5,6]. In the treatment strategy for advanced ovarian cancer, chemotherapy remains the standard and necessary approach, especially for patients who are not suitable for surgery or other therapies [7]. Primary surgical cytoreduction combined with platinum-based chemotherapy is the first-line treatment for newly diagnosed advanced ovarian cancer [8]. In patients who receive chemotherapy, complete clinical remission (CCR) is observed in 75% of cases. Unfortunately, 75% of patients who respond to chemotherapy will experience a relapse within 28 months [9]. For recurrent ovarian cancer, platinum-based chemotherapy remains the fundamental treatment to extend disease control. Nevertheless, it is worth noting that late-stage ovarian cancer patients exhibit significant heterogeneity in their response to chemotherapy [10]. Thus, identifying patients who would benefit from chemotherapy is crucial in the management of ovarian cancer. Based on previous studies, bioinformatics analysis has demonstrated its efficacy in identifying prognostic gene features associated with ovarian cancer [11–14]. Disease stratification based on genetic features has increasingly gained attention [15], with machine learning techniques, such as random forests and convolutional neural networks (CNN), being explored for the analysis of multi-omics data [16–18]. Furthermore, the application of these techniques in multimodal data integration is continuously expanding [19].Therefore, there is an urgent need to identify molecular features directly associated with chemotherapy benefits and develop a reliable assessment tool that enables more precise evaluation of chemotherapy response in clinical practice. Such a tool would facilitate the multidisciplinary management of advanced ovarian cancer and ultimately improve patient prognosis.

Network analysis of cancer omics data could isolate nodes that play crucial role in cancer pathophysiological processes, usually achieved by introducing correlation and causality relationships [20,21]. Correlation network describes the pairwise relationships between genes using the Pearson coefficient and is used to identify the core associated molecules in the network [20]. However, a limitation of correlation networks is their inability to determine the directionality of the relationships. Bayesian network analysis constructs a directed acyclic graph and reveals the directed relationships between each pair of genes and the strength of dependence between variables using conditional probability distribution [21]. Comparing to the classic differential analysis, network analysis could better model the tumor processes and identify node genes that were missed by the classic difference analysis. Recently, network analysis has been used to identify node genes in multiple cancers, and has demonstrated a strong capability for node gene screening [22,23].

In this study, we conducted correlation and Bayesian causal network analyses to identify chemotherapy prognosis-related genes, and further developed CBI. Survival analysis was employed to test the prognostic stratification capability of the CBI. Moreover the efficiency and stability were verified by internal and external validations using the random forest model. Furthermore, we explored the diverse molecular characteristics and additional immunotherapy benefits of different CBI subgroups. Our work provided a novel clinical decision tool to assess the individual chemotherapy benefits of patients with advanced ovarian cancer. CBI can be used as a prognostic indicator, and our results revealed that immunotherapy can be used as an additional treatment option to improve the prognosis for patients with low CBI.

## 2. Materials and methods

### 2.1. Patients and database

The transcriptome data, as well as clinical (survival) and mutation information of patients with ovarian cancer were obtained from The Cancer Genome Atlas (TCGA) project (https://portal.gdc.cancer.gov/). In order to verify the accuracy and stability of the model, we downloaded the survival information and transcriptome data of four external validation cohorts (GSE17260, GSE26193, GSE30161 and GSE32062) from the Gene Expression Omnibus database (https://www.ncbi.nlm.nih.gov/geo/). The inclusion criteria require patients to have a confirmed diagnosis of stage II-IV ovarian cancer and to have undergone chemotherapy. Patients who have undergone radiotherapy or have incomplete clinical information are excluded.

### 2.2. Identified chemotherapy prognosis-related genes through network analysis

A total of 146 patients with advanced ovarian cancer who received chemotherapy were included in our study cohort. Based on the 3-year overall survival of these patients, 146 patients were divided into a cohort with better prognosis (n = 76) and a cohort with poor prognosis (n = 70). Next, we performed correlation analysis for the two cohorts by package “WGCNA” and extracted the core correlation network (weight of gene-pairs>0.85), respectively [20]. Package “cluster Profiler” was used to perform the Gene Ontology (GO) functional enrichment analyses for these genes with strong correlation. Based on the top 200 enriched biological processes, these genes were subsequently grouped into five modules. R package “bnlearn” was applied to perform Bayesian causal network analysis for the purpose of selecting core node genes from the five biological modules (strength>0.85 and direction>0.5). Finally, we identified node genes between two cohorts based on screening the network degree≥3 (both in-degree and out-degree). Cox proportional hazard regression analysis was performed for these different node genes, and we finally identified ten genes as the chemotherapy prognosis-related genes.

### 2.3. Development and validation of CBI

Based on the expression of the chemotherapy prognosis-related genes, we developed the CBI to predict chemotherapy benefit for patients with advanced ovarian cancer. The calculation formula is CBI = 1/Σ(C×Exp) [24]. In this formula, C refers to the coefficient of multivariate Cox regression, and Exp refers the expression of each feature gene. By the correction of important clinical features, we confirmed that CBI could be used as an independent index to predict the chemotherapy benefit. And based on the CBI score, patients were divided into three subgroups (CBI-high, CBI-medium and CBI-low). R package “survminer” and “survival” were used to perform the survival analyses of CBI subgroups, and the survival difference was assessed by log-rank test.

We developed a random forest classifier model based on the expression of ten chemotherapy prognosis-related genes. Patients in this study were divided into training set and validation set in a 7:3 ratio. Training set was used to develop the prediction model, and validation set was used to verify model. In order to achieve the best classification effect, we applied learning curve and grid search to match the best hyperparameters. Function “feature importances” displayed the weight of each feature gene. In addition, we also curated GSE17260 (ovarian cancer, N = 110), GSE26193 (ovarian cancer, N = 107), GSE30161 (ovarian cancer, N = 58) and GSE32062 (ovarian cancer, N = 260) from GEO database as external validation sets. Area under the receiver operating characteristic curve (AUC) were applied to assess predictive accuracy in each cohort.

### 2.4. Molecular characteristic analysis in CBI subgroups

We identified 346 CBI expanded genes based on the strongly causal relationship (strength>0.85) with chemotherapy prognosis-related genes. In order to explore the biological function, we used R package “cluster Profiler” and Gene Set Enrichment Analysis (GSEA) software to perform GO, Kyoto Encyclopedia of Genes and Genomes (KEGG) and GSEA functional enrichment analysis [25,26]. To explore the therapeutic strategies which could further improve the prognosis, we assessed the potential responsiveness to immune checkpoint blockade (ICB) treatment in CBI subgroups. Firstly, we explored the expression of immune-checkpoint molecules, including *PDCD1*, *CD274*, *PDCD1LG2*, and *CTLA4*. Additionally, we investigated the verified biomarker of ICB treatment, including the expression of CD8, tumor mutational burden (TMB), immune cytolytic activity (CYT), antigen presenting machinery and T cell-inﬂamed gene expression proﬁles. Furthermore, we analyzed the summary information of mutation in different CBI subgroups to investigate the potential molecular targets. We demonstrated the top ten mutation genes in CBI-high and CBI-low groups and compared the different mutation frequency between the two groups. The summary-level mutation information was visualized using the R package “maftools”.

### 2.5. RNA isolation and gene expression analysis

The RNA of ovarian epithelial cells (IOSE-80) and ovarian cancer cells (SKOV-3) were extracted from cells using Multisource Total RNA Mini Kit (Axygen Scientific, CA, USA) and reverse transcription was accomplished with PrimeScript™ RT reagent Kit with gDNA Eraser (Takara, Beijing, China) according to the manufacturer’s instructions. Finally, qPCR was performed using an Applied Biosystems CFX96 Fast Real-Time PCR system (Bio-Rad, CA, USA) with universal SYBR Green Supermix (Takara, Beijing, China). Expression results obtained were normalized to ACTB levels and triplicate assays were performed. Solubility curves were analyzed to exclude the possibility of nonspeciﬁc ampliﬁcation products. Primers sequences were listed in Table 1. Sequences were synthesized and purified by Shanghai Sangon Biotech (Shanghai, China).

**Table 1:** Primers sequences for RT-qPCR.

Gene	Forword (5’- 3’)	Reword (5’- 3’)
*COL6A3*	TTAGTGGCCGTCTTTTGCCT	CGCACCATTTTTGACATCTGCT
*SPI1*	CCTGAGGGGCTCTGCATTGG	CAGGTCTTCTGATGGCTGAGGG
*HSF1*	CCCGGATTCAGGGAAGCAG	AAGTAGGAGCCCTCTCCCAG
*CD3E*	CTGGCCTCCGCCATCTTAG	CCCCCAAACGCCAACTGAT
*PIK3R4*	AGATGACAAACGGGCCAGAA	CTTTAGGTAGGGCCTGTGGC
*MZB1*	AGCTGTGGCTTACCAGATGT	ATCCGTGTAGACCAACTCGC
*FERMT3*	GGGGACTACATCGACTCGTCA	GTCCTGCTTGCGATTGATCTG
*PSMB9*	CGCTTCACCACAGACGCTAT	CCACACCGGCAGCTGTAATA
*RSF1*	CTTCGCCGTAGTCTGCTCCT	ACCAATTCTTTTGGTACTTCTCCG
*GZMA*	AAGAGACTCGTGCAATGGAGA	AAGGCCAAAGGAAGTGACCC
*ACTB*	CATGTACGTTGCTATCCAGGC	CTCCTTAATGTCACGCACGAT

### 2.6. Statistical analysis

Statistical analysis was carried out in R 4.1.0 (https://www.R-project.org/). The demographic and clinical characteristics of cohorts with different prognosis were compared by chi-square statistical test and two-sampled t-test. And python version 3.10 was used to construct the random forest model. Unpaired/paired Student’s t test was performed to compare two groups to each other. If the variance between the two groups was unequal, a Welch’s correction was applied. To compare more than two groups, statistical significance was determined using one/two-way ANOVA with Tukey’s multiple comparison test. p value below 0.05 was considered statistically significant. Significance is indicated as follows: *p < 0.05, **p < 0.01, ***p < 0.001, ****p < 0.0001, ns: no significance.

### 2.7. Ethics statement

Our study is based on publicly available data from The Cancer Genome Atlas (TCGA) and the Gene Expression Omnibus (GEO). It does not involve direct interaction with human participants or the collection of new clinical data. Therefore, it did not require ethical approval.

## 3. Results

### 3.1. Identified chemotherapy prognosis-related genes through network analysis

**Fig 1:**
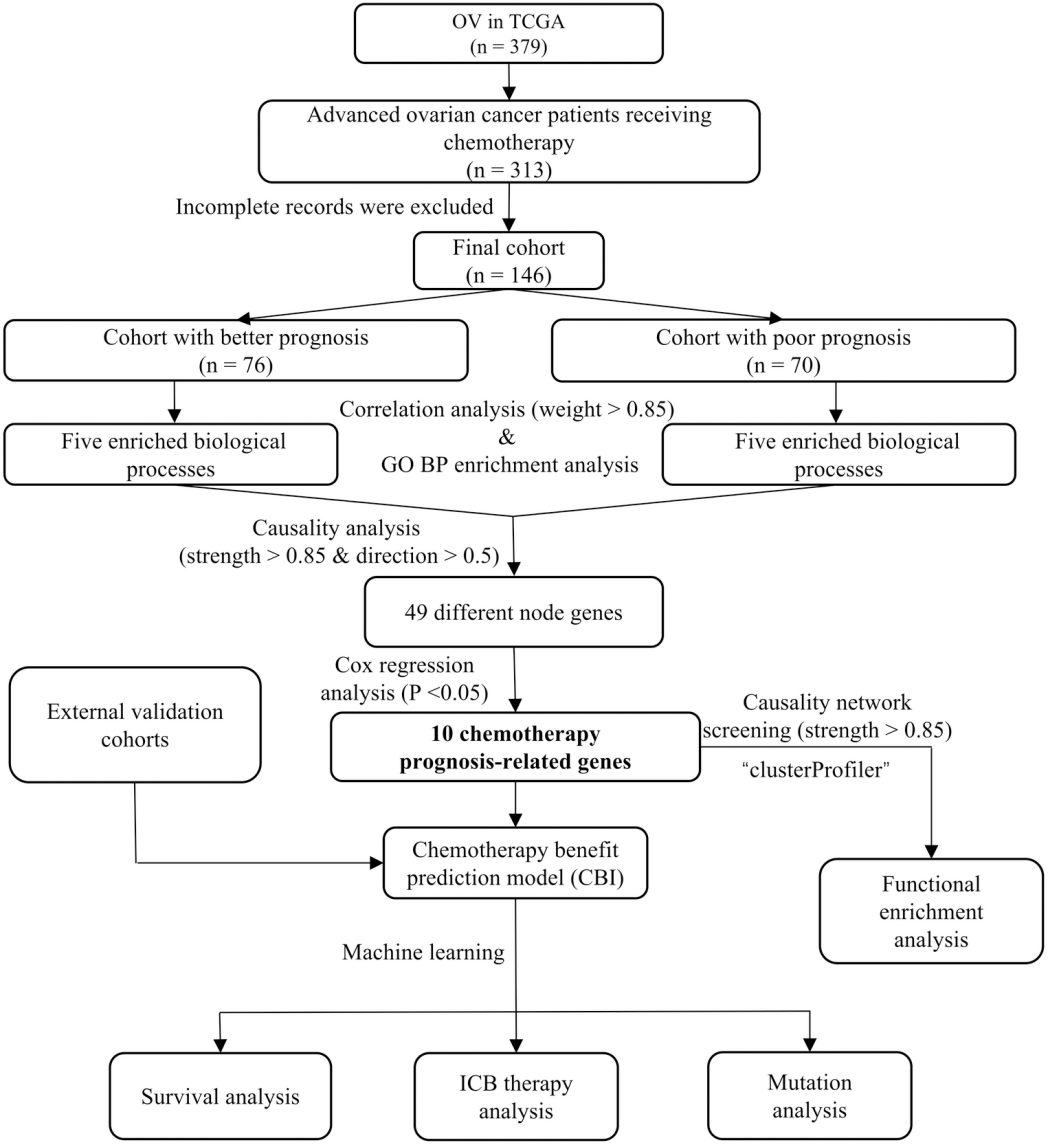
Cohort screening and analyses workflow.

**Fig 2:**
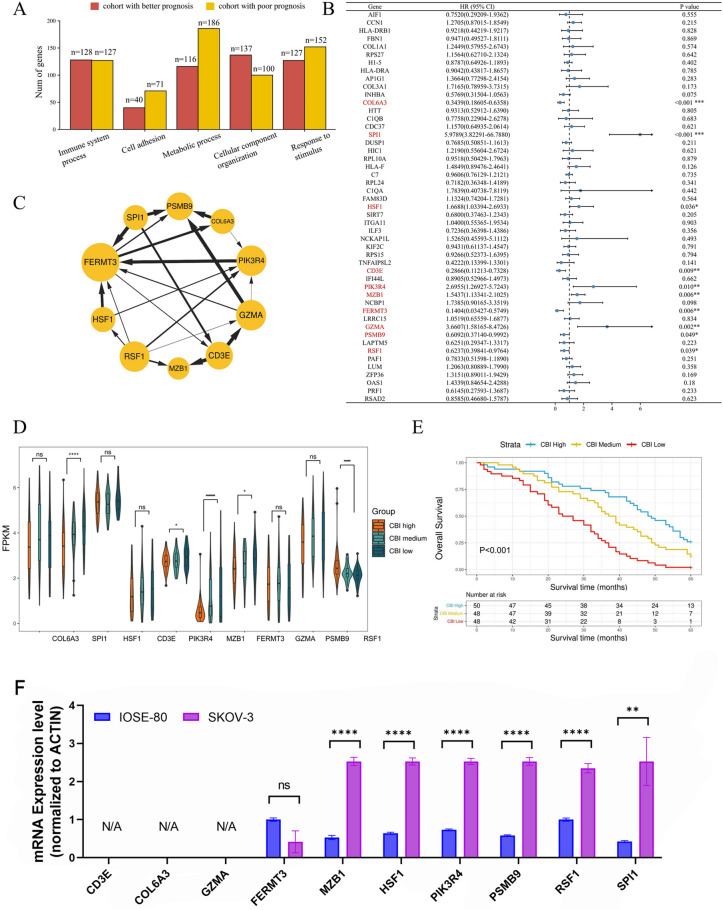
Developing CBI for ovarian cancer patients receiving chemotherapy. (A) The histogram showed the number of genes isolated by Bayesian causal network (strength > 0.85) in 5 enriched biological processes. (B) Multivariate Cox regression analysis of the 49 different chemotherapy node genes to select chemotherapy prognosis-related genes. (C) The causality relationship of ten chemotherapy prognosis-related genes. (D) The expression of chemotherapy prognosis-related genes among CBI subgroups. (E) The overall survival was significantly different among CBI subgroups. (F) Gene expression was detected by RT-qPCR in normal and ovarian cancer cells. Data are presented as mean ± SD of triplicate cultures. N/A indicates that these genes are not expressed in either cell lines (cycles > 35). (**p < 0.01, ****p < 0.0001; unpaired two-sided Student’s t test.).

The cohort screening and analyses workflow were demonstrated in Fig 1. To investigate the correlation between gene expression and the prognosis of ovarian cancer patients who received chemotherapy, we divided the patients into two cohorts based on 3-year overall survival: a cohort with a favorable prognosis (N = 76) and a cohort with a poor prognosis (N = 70). Clinical characteristics were evenly distributed between the two cohorts (Table 2). Correlation network analysis identified 4,415 strongly related gene-pairs in cohort with better prognosis and 4,050 strongly related gene-pairs in cohort with poor prognosis (weight>0.85) (Supplementary material, S1 Table in S1 File). We conducted GO enrichment analyses on the strongly related genes and found that each gene set was enriched in 442 and 441 biological processes, respectively. Based on the top 200 enriched biological processes (P < 0.05), we could divide these correlation genes into five biological modules: “immune system process”, “cell adhesion”, “metabolic process”, “cellular component organization”, “response to stimulus”. (Supplementary material, S1 and S3 Tables in S1 File) Bayesian causal network analyses were further performed to define the causality nodes of each biological module (Fig 2A and Supplementary material, S4–S8 Tables in S1 File). Finally, we identified 49 different node genes from the five biological modules (strength>0.85, degree≥3) (Supplementary material, S9 Table in S1 File).

**Table 2:** Clinical characteristics in cohorts with different prognosis.

Characteristics	Cohort with better prognosis (n = 76)	Cohort with poor prognosis (n = 70)	P value
**Survival time (median, months)**	50	20.5	<0.001
**Age (IQR, years)**	57 (51 ~ 67)	61 (53.5 ~ 71)	0.143
**Race (n, %)**			0.871
White	69 (90.79)	63 (90.00)	
Black or African American	7 (9.21)	7 (10.00)	
**Histologic grade (n, %)**			0.232
Grade II	10 (13.16)	5 (7.14)	
Grade III	66 (86.84)	65 (92.86)	
**Tumor residual (n, %)**			0.288
No Macroscopic disease	11 (14.47)	12 (17.14)	
1-10 mm	47 (61.84)	36 (51.43)	
11-20 mm	7 (9.21)	4 (5.71)	
>20 mm	11 (14.47)	18 (25.71)	
**Laterality (n, %)**			0.868
Left	9 (11.84)	9 (12.86)	
Right	8 (10.53)	7 (10.00)	
Bilateral	59 (77.63)	54 (77.14)	
**Stage (n, %)**			0.459
Stage II	3 (3.95)	1 (1.43)	
Stage III	63 (82.89)	56 (80.00)	
Stage IV	10 (13.16)	13 (18.57)	

### 3.2. Development and validation of the chemotherapy benefit prediction model

We employed Cox regression analysis to investigate the association between the 49 identified node genes and prognosis. Subsequently, we identified ten genes (*COL6A3*, *SPI1*, *HSF1*, *CD3E*, *PIK3R4*, *MZB1*, *FERMT3*, *GZMA*, *PSMB9* and *RSF1*) which could serve as independent prognostic factors for patients with advanced ovarian cancer (P < 0.05) (**Fig 2B**). In addition, we explored the causal relationships among these ten genes and found that four immune-related genes (*CD3E*, *GZMA*, *MZB1*, *PSMB9*) shown strong causal relationships (strength > 0.75) (**Fig 2C**). Based on the expression of these genes, we constructed a multivariate Cox regression model and developed an index to predict chemotherapy benefit. The CBI index was derived from the Risk Score calculated using the Cox proportional hazards model in the “survival” package in R. Specifically, the Risk Score was determined as a linear combination of the model coefficients (βi) and their corresponding feature values (xi), based on the following formula: $\text{Risk Score}=\beta_{1}\cdot x_{1}+\beta_{2}\cdot x_{2}+\ldots +\beta_{n}\cdot x_{n}$ [27]. The CBI index was subsequently defined as the reciprocal of the Risk Score, expressed as:

$\text{CBI}=\frac{1}{\text{Risk Score}}$

According to the CBI score, we divided these patients into three subgroups (CBI high, medium and low) and investigated the expression levels of the identified chemotherapy prognosis-related genes across these subgroups (**Fig 2D**). The survival analysis shown the overall survival was significantly different among three subgroups, indicating CBI had good capability of prognostic stratification (p < 0.001, **Fig 2E**). Moreover, the expression of these genes in normal and tumor cells was measured by RT-qPCR. The results shown that gene *MZB1*, *HSF1*, *PIK3R4*, *PSMB9*, *RSF1* and *SPI1*were significantly overexpressed in ovarian cancer cells SKOV-3 (**Fig 2F**). We noted that some genes positively related to immunity were negatively related to CBI score and up-regulated in CBI-low group. These results suggested that tumor immune escape may play an important role in chemotherapy benefit.

**Fig 3:**
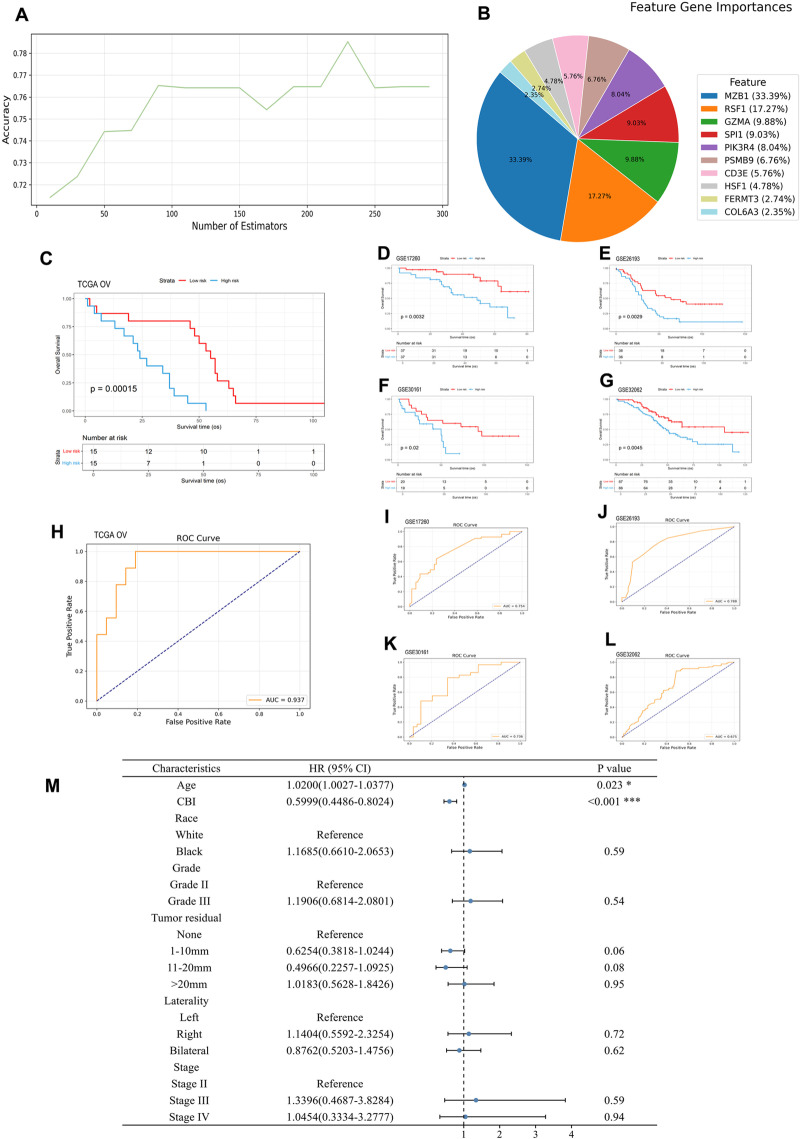
Constructing random forest model and verifying CBI. (A) Learning curve of estimators from 0 to 300. (B) The importance of each chemotherapy prognosis-related gene in random forest model. **(C, D**, E) Survival analyses of CBI subgroups in the internal validation set (P < 0.001), GSE17260 (P < 0.005), GSE30161 (P < 0.05), GSE26193 (P < 0.005), and GSE32062 (P < 0.005), respectively. (C-G) AUC of the internal validation set, GSE17260, GSE26193, GSE30161 and GSE32062 are 0.94, 0.75, 0.79, 0.74 and 0.68 respectively. (I) Multivariate COX regression analysis confirmed CBI could serve as the independent prognostic factor of advanced ovarian cancer patients receiving chemotherapy.

For improved discriminability, we selected the CBI-high and CBI-low groups to develop the random forest classifier model. We randomly sampled 70% of the total patients as the training set and 30% as the validation set. By the application of grid search and the learning curve (Fig 3A), the best hyperparameters of the classifier model was identified as follow: Random Forest Classifier (class weight = ‘balanced’, criterion=’entropy’, n_estimators = 230, max_features = 10, min_samples_leaf = 3, max_depth = 5). The importance of each feature gene was 33.39%,17.27%, 9.88%, 9.03%, 8.04%, 6.76%, 5.76%, 4.78%, 2.74% and 2.35%, respectively (Fig 3B). CBI shown great stability and prognostic stratification ability in both the internal (p < 0.001 Fig 3C) and four external validation sets (GSE17260, GSE26193, GSE30161 and GSE32062) (p < 0.02, Fig 3D–G), the AUC were 0.94, 0.75, 0.79, 0.74 and 0.68, respectively (Fig 3H–L). Moreover, after adjusting for clinical characteristics, CBI was validated as an independent prognostic predictor for advanced ovarian cancer (internal validation: HR = 0.60, p < 0.001) (Fig 3I). This was further supported in four external validation cohorts (HR, 0.360 to 0.443, p < 0.02) (Supplementary material, S1–S4 Figs in S1 File).

### 3.3. Function analyses of CBI expended genes

**Fig 4:**
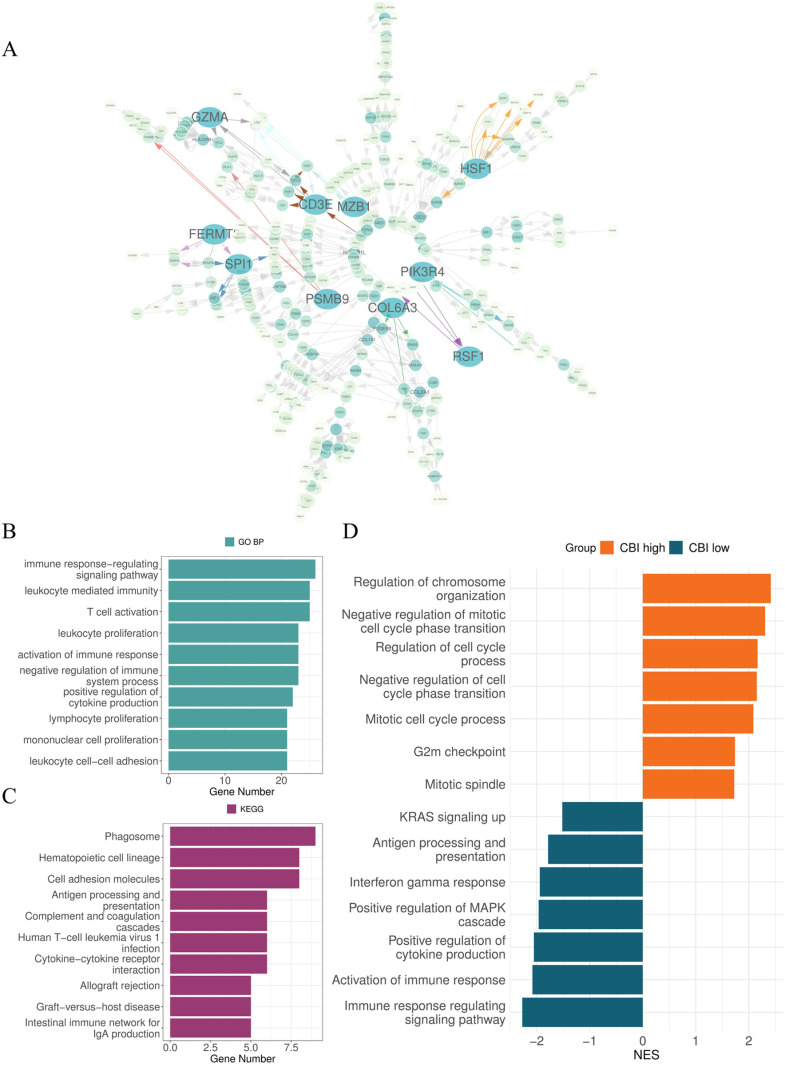
Functional enrichment analyses of CBI expanded genes. (A) The causality relationship of the 346 CBI expanded genes (strength > 0.85). (B) Top 10 GO biological processes (P < 0.001) and **(C)** top 10 KEGG pathways of CBI expanded genes (P < 0.001). (D) Top up-regulated pathways in CBI-high group and CBI-low group in GSEA (p < 0.05, FDR < 0.25).

In order to better understand the different molecular characteristics of CBI subgroups, we screened the causal network relationship (strength>0.85) with the ten chemotherapy-related genes and finally identified 346 CBI expended genes (Fig 4A and S10 Table in S1 File). We performed GO biological processes enrichment analysis of these genes and found that were mainly enriched in “immune response-regulating signaling pathway”, “leukocyte mediated immunity”, “T cell activation”, “leukocyte proliferation”, “activation of immune response” (Fig 4B). And the result of KEGG enrichment analysis shown these genes were mainly enriched in “Phagosome”, “Hematopoietic cell lineage”, “Cell adhesion molecules”, “Antigen processing and presentation”, “Cytokine-cytokine receptor interaction” (Fig 4C). In addition, we explored the up-regulated pathways in CBI-high and CBI-low groups by GSEA. The findings demonstrated that the CBI-high group was mainly enriched in pathways that exhibited negative regulation of the cell cycle, while the CBI-low group was enriched in signal pathways related to cancer that promoted the cell cycle. (P < 0.05, FDR < 0.25) (Fig 4D).

### 3.4. Patients with low CBI may benefit from ICB treatment

**Fig 5:**
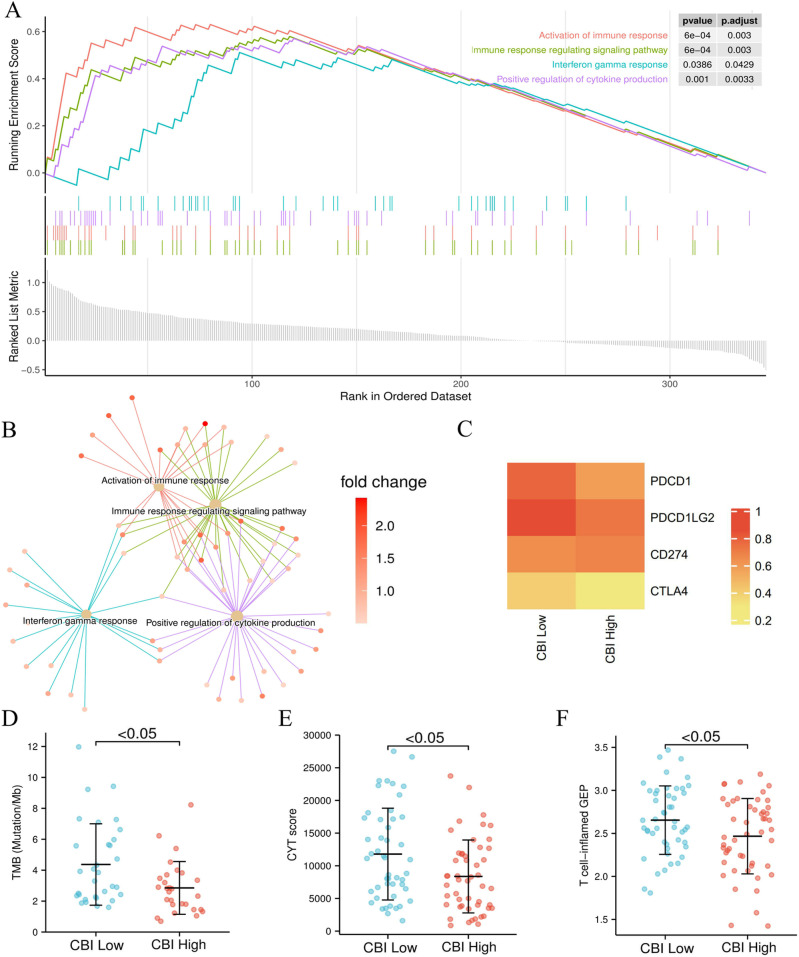
CBI-low group may benefit more from ICB treatment. (A-B) Immune-related pathways are up-regulated in the CBI-low group (P < 0.05, FDR < 0.25). (C) High expression of immune-checkpoint molecules in CBI-low group. (D-E) The biomarker scores of ICB treatment response are higher in CBI-low group (P < 0.05).

In addition to the cancer-related signal pathways, we made a surprising discovery that immune-related pathways were enriched in CBI-low group (**Fig 5A**). These pathways shown a number of genes were shared, and the expression of these genes was significantly higher in CBI-low group (**Fig 5B**). Therefore, we further explored whether patients with low CBI score could benefit from immune checkpoint blockade (ICB) therapy. We compared the expression level of immune-checkpoint molecules between CBI subgroups and found that immune-checkpoint molecules were highly expressed in CBI-low group (**Fig 5C**). Then, we analyzed the transcriptomic biomarkers of ICB response, and observed that the scores of TMB, CYT and T cell-inﬂamed GEP were significantly higher in CBI-low group. Tumor mutation could generate new antigens and activate immune reaction of anti-tumor. CYT and T cell-inﬂamed GEP could reflect the T cells related anti-tumor effects. Based on the findings, it is hypothesized that the increased TMB in the CBI-low group could be responsible for the observed elevation of CYT and T cell-inflamed GEP. (**Fig 5D**–F). In general, CBI-low group had stronger immune characteristics, which was consisted with our above results. However, the higher expression of immune-checkpoint molecules may impede the immune response and eventually lead to poor prognosis. Therefore, this finding indicated that patients with low CBI score may benefit more from ICB treatment.

### 3.5. Mutation and target therapy of each CBI subgroup

**Fig 6:**
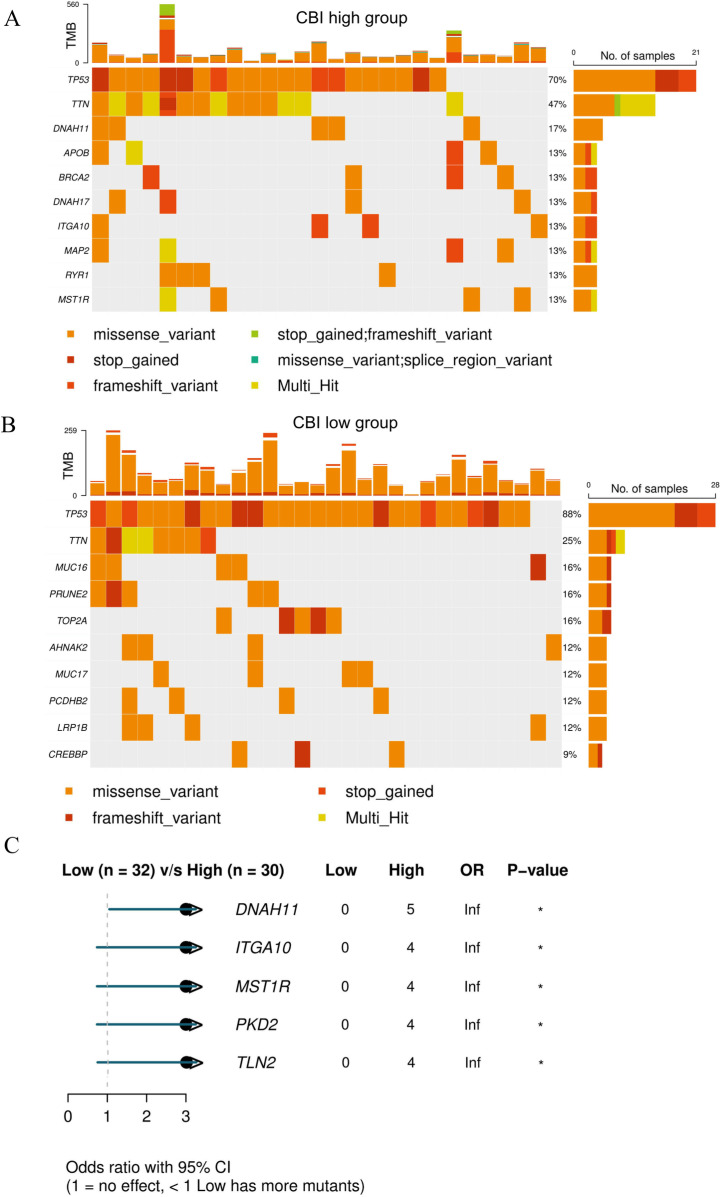
Mutation analysis of CBI-high and CBI-low groups. (A-B) The top 10 mutation genes and types in CBI-high group and CBI-low group, respectively. (C) The mutant differential gene between CBI-high group and CBI-low group (P < 0.05).

We finally analyzed the mutation landscape of the two CBI subgroups. TP53 and TTN were the common high-frequency mutated genes in both CBI-high and CBI-low groups, while the other top ten mutated genes were totally different between the two subgroups. The top ten mutation genes in CBI high group were *TP53*, *TTN*, *DNAH11*, *APOB*, *BRCA2*, *DNAH17*, *ITGA10*, *MAP2*, *RYR1* and *MST1R*. The top ten mutation genes in CBI-low group were *TP53*, *TTN*, *MUC16*, *PRUNE2*, *TOP2A*, *AHNAK2*, *MUC17*, *PCDHB2*, *LRP1B* and *CREBBP*. Missense variant was the most common mutation type (**Fig 6A** and B). Besides, the mutation rate of *DNAH11*, *ITGA10*, *MST1R*, *PKD2* and *TLN2* in CBI-high group was significantly higher than that in CBI-low group (**Fig 6C**). These genes may be the potential therapeutic targets for patients with higher CBI score. Among them, macrophage stimulating 1 receptor (*MST1R*, also known as *RON*) as a receptor tyrosine kinase had been proved to be a therapeutic target for tyrosine kinase inhibitor (TKI) [28].

## 4. Discussion

Ovarian cancer is the major cause of gynecological cancer death worldwide [8]. While targeted and immunotherapies have been applied in the treatment of ovarian cancer, the mainstay of therapy for advanced ovarian cancer still remains the combination of surgical intervention and chemotherapy [1]. However, chemotherapy can also have detrimental effects. Studies have shown that chemotherapy may induce premature menopause and increase the risk of childhood cancer in offspring [29,30]. Additionally, there are still a significant number of patients who do not respond to chemotherapy [31]. Given the potential for non-response and the risk of adverse events, it is crucial to establish a means of identifying the patients who would most benefit from chemotherapy. Previous research has developed a prognostic model for chemotherapy based on immune-related genes [32]. However, the intricate interplay between chemotherapeutic drugs and tumor infiltrating immune cells (TIICs) remains controversial [33]. In this study, we identified the molecular characteristics of advanced ovarian cancer patients with varying chemotherapy prognoses and developed the CBI index, which demonstrated robust predictive performance in both internal and external validation cohorts (the lowest AUC: 0.675). While several existing models have made progress in prognostic stratification of ovarian cancer (AUC: 0.48–0.624) [14], most tools primarily focus on risk assessment. In contrast, CBI provides a promising and reliable tool for accurately predicting chemotherapy benefit, potentially addressing key challenges in the clinical management of ovarian cancer.

Comparing with the classic differentially expressed gene analysis, network analysis could isolate nodes related to event occurrence from omics data, and identify those nodes which were missed by classic difference analysis. In this study, we firstly used correlation network analysis to isolate the strongly related gene-pairs, and further used Bayesian causal network to identify nodes with strong causality relationship. Moreover, double network screening enhanced the stability and reliability of identified nodes. Based on the network analysis, we identified ten chemotherapy prognosis-related genes, and finally developed a prediction model of CBI by the ten genes. Patients with high CBI score benefited more from chemotherapy, while patients with low CBI score benefited less. Through the correction of clinical features, CBI was confirmed to be an independent chemotherapy benefit predictor for patients with advanced ovarian cancer. In a nutshell, our results shown that network analysis could better fit tumor characteristics, which is already proved with the results of previous studies [22,23].

The following ten genes were used to develop CBI: *COL6A3*, *SPI1*, *HSF1*, *CD3E*, *PIK3R4*, *MZB1*, *FERMT3*, *GZMA*, *PSMB9* and *RSF1*. *COL6A3* is the largest subunit of type VI collagen, a major protein of extracellular matrix [34]. Previous study has revealed that *COL6A3* is involved in stimulating proliferation and preventing apoptosis [35]. *SPI1* is also known as the oncogene of erythroleukemia [36]. In recent years, *SPI1* has been proved to be associated with the poor prognosis of multiple cancer types through disordering the immune processes and cell cycle [37]. *HSF1* was reported to be involved in epithelial-to-mesenchymal transition and further promote the processes of tumor metastasis. In addition, due to the close association between *HSF1* expression level and prognosis, it has been served as a prognostic biomarker and a potential target for cancer treatment [38]. *PIK3R4* is a regulatory subunit of PI3K signaling pathway that can activate amino acid-induced mTOR, and subsequently regulate the cell cycle [39]. *MZB1* and *FERMT3* were both reported to be positively associated with TIICs [40,41]. And some studies have shown that *GZMA* plays an essential role in cytolytic T lymphocytes and natural killer mediated immunity [42]. *CD3E* and *PSMB9* were involved in the processes of antigen processing and presentation, and further enhances the anti-tumor immunity mediated by T lymphocytes [43,44]. *RSF1* with the optimal expression level has the capacity to protect normal cellular process and suppress the transcription of oncogene. However, overexpressed *RSF1* will promote growth and proliferation [45]. We have made the surprising discovery that all chemotherapy prognosis-related genes are closely associated with both tumor growth and TIICs, which indicate the potential interaction between chemotherapy and the immune tumor microenvironment. This result is consistent with previous study [33]. It is worth noting that certain chemotherapy prognosis-related genes positively associated with TIICs were found to be negatively associated with CBI and significantly overexpressed in the CBI-low group. This may be related to the tumor immune escape, which is consistent with our findings on immune-checkpoint molecules [46]. Nevertheless, further mechanism research is still needed to elucidate the mechanisms underlying the relationship between immune tumor microenvironment and chemotherapy benefit.

We delved further into the functional differences between CBI subgroups. Consistent with our previous findings, we observed that the CBI-low subgroup was enriched in pathways related to cell proliferation and migration, while the CBI-high subgroup was enriched in processes that inhibit the cell cycle, potentially explaining the differing prognoses between the two subgroups. Interestingly, we also discovered that immune-related pathways were highly enriched in the CBI-low subgroup, indicating a higher level of immune infiltration. We explored the potential of the CBI-low subgroup as a biomarker for predicting the response to ICB treatment, and found that the expression of immune checkpoint molecules was higher in the CBI-low subgroup, possibly related to tumor immune escape. Additionally, the TMB, CYT, and T cell-inflamed GEP scores were higher in the CBI-low subgroup, all of which are potential biomarkers for ICB treatment response. Therefore, our research suggests that patients with a low CBI score may be a potential population for ICB treatment. However, further research is needed to validate our findings and explore potential mechanisms.

In order to comprehensive understand the molecular difference of CBI subgroups, we explored the mutational landscape of two CBI subgroups. The most frequent mutation found in both two CBI subgroups was *TP53*, with a higher incidence in the CBI-low group compared to the CBI-high group (88% versus 70%). *TP53* is a tumor suppressor gene that is frequently mutated in multiple types of cancer. Missense mutation of *TP53* allows tumor cell to escape death and promote proliferation. In recent years, many studies have focused on reactivating *TP53* mutations, yet challenges remain to be overcome [47]. Germline mutations in *BRCA1/2* are widely recognized as significant risk factors for tumorigenesis [48]. Compared to the general population, individuals with *BRCA1/2* mutations have a 70% and 40% increased risk of developing breast and ovarian cancer, respectively [49]. Previous studies have demonstrated that patients carrying *BRCA* mutations responded better to platinum-based therapy [50,51], which is in line with our findings. We have also observed a higher incidence of *BRCA2* mutations in the CBI-high cohort, which confirmed the significant impact of *BRCA* mutation detection in the treatment of ovarian cancer. Additionally, *MST1R* () is a receptor tyrosine kinase which activates the RAS-ERK and PI3K-AKT pathways, thereby further promoting cell growth. Serval studies have demonstrated that TKIs can block *MST1R* and inhibited tumor proliferation, indicating that *MST1R* is a promising target for cancer therapy. Notably, the combination of TKIs and chemotherapeutics could achieve the maximum therapeutic benefit for patients. This implies that CBI-high patients with *MST1R* mutations may benefit from improved prognosis through the use of combined TKIs [28]. However, further clinical trials are required to verify these findings.

This work has some limitations. Firstly, the sample size of our study population is limited, which may result in bias caused by small sample size. Secondly, although CBI has demonstrated strong predictive ability in the training set and two external validation sets, further validation is still required through additional external datasets or in clinical practice. Regarding the selection of immunotherapy, CBI can only serve as a reference indicator, and its underlying mechanism still requires further research for clarification.

In general, this work has provided a novel chemotherapy benefit index for patients with advanced ovarian cancer through network analyses and machine learning. CBI could serve as a prognostic prediction tool for patients with advanced ovarian cancer, and also as a potential indicator for immunotherapy.

## Supporting information

S1 File Table S1. Gene pairs of correlation network with weight > 0.85 in cohorts with different prognosis. Table S2. Top 200 GO biological processes in cohort with better prognosis. Table S3. Top 200 GO biological processes in cohort with poor prognosis. Table S4. Gene pairs of causal network with strength > 0.85 and direction > 0.5 in immune system process. Table S5. Gene pairs of causal network with strength > 0.85 and direction > 0.5 in cell adhesion. Table S6. Gene pairs of causal network with strength > 0.85 and direction > 0.5 in metabolic process. Table S7. Gene pairs of causal network with strength > 0.85 and direction > 0.5 in cellular component organization. Table S8. Gene pairs of causal network with strength > 0.85 and direction > 0.5 in response to stimulus. Table S9. Different chemotherapy node genes. Table S10. 346 chemotherapy expanded feature genes. Fig S1. Multivariate Cox regression analysis of GSE17260. Fig S2. Multivariate Cox regression analysis of GSE26193. Fig S3. Multivariate Cox regression analysis of GSE30161. Fig S4. Multivariate Cox regression analysis of GSE32062. (PDF)

## Abbreviations

CBI: chemotherapy benefit index
ICB: immune checkpoint blockade
TCGA: The Cancer Genome Atlas
GSEA: Gene Set Enrichment Analysis
GO: Gene Ontology
COL6A3: Collagen type VI alpha 3 chain
SPI1: Spi-1 proto-oncogene
HSF1: Heat shock transcription factor 1
CD3E: CD3 epsilon subunit of T-cell receptor complex
PIK3R4: Phosphoinositide-3-kinase regulatory subunit 4
MZB1: Marginal zone B and B1 cell specific protein
FERMT3: FERM domain containing kindlin 3
GZMA: Granzyme A
PSMB9: Proteasome 20S subunit beta 9
RSF1: Remodeling and spacing factor 1
MST1R: Macrophage stimulating 1 receptor
TMB: tumor mutational burden
CYT: cytolytic activity
TKI: tyrosine kinase inhibitor
TIICs: tumor infiltrating immune cells
