# Supplementary material for: Identification of a novel chemotherapy benefit index for patients with advanced ovarian cancer based on Bayesian network analysis
Source: PLoS One. 2025 May 27;20(5):e0322130. doi: 10.1371/journal.pone.0322130 (PMC12112407; doi:10.1371/journal.pone.0322130)

SUPPLEMENTAL MATERIAL

Identification of a novel chemotherapy benefit index for patients with advanced ovarian cancer based on Bayesian network analysis

Shuxiao Ma†, Lu Zhou†, Yi Liu, Hui Jie Min Yi, Chenglin Guo, Jiandong Mei, Chuan Li, Shiyou Wei, Lei Zhu* and Senyi Deng*

[1]Department of Thoracic Surgery and Institute of Thoracic Oncology, West China Hospital, Sichuan University. Chengdu, PR China.

†These authors have contributed equally to this work.

*Correspondence:

Senyi Deng, M.D., Ph.D. senyi_deng@scu.edu.cn

Lei Zhu, M.D., Ph.D. lei_zhu@scu.edu.cn

Table S1. Gene pairs of correlation network with weight>0.85 in cohorts with different prognosis.

Table S2. Top 200 GO biological processes in cohort with better prognosis.

Table S3. Top 200 GO biological processes in cohort with poor prognosis.

Table S4. Gene pairs of causal network with strength >0.85 and direction>0.5 in immune system process

Table S5. Gene pairs of causal network with strength >0.85 and direction>0.5 in cell adhesion.

Table S6. gene pairs of causal network with strength >0.85 and direction>0.5 in metabolic process.

Table S7. Gene pairs of causal network with strength >0.85 and direction>0.5 in cellular component organization.

Table S8. Gene pairs of causal network with strength >0.85 and direction>0.5 in response to stimulus.

Table S9. Different chemotherapy node genes.

Table S10. 346 Chemotherapy expanded feature genes.

Fig S1. Multivariate Cox regression analysis of GSE17260

Fig S2. Multivariate Cox regression analysis of GSE26193

Fig S3. Multivariate Cox regression analysis of GSE30161

Fig S4. Multivariate Cox regression analysis of GSE32062

Table S1. gene pairs of correlation network with weight>0.85 in cohorts with different prognosis

Cohort with better prognosis			Cohort with better prognosis		
Gene 1	Gene 2	weight	Gene 1	Gene 2	weight
H4C6	H3C7	0.875665678	H4C6	H3C11	0.858077524
NTM	FAP	0.887717207	H4C6	H3C7	0.863360707
NTM	ADAMTS12	0.856466496	NTM	FAP	0.894457282
NTM	LRRC15	0.915292934	NTM	ADAMTS12	0.902325962
NTM	INHBA	0.876950085	NTM	LRRC15	0.918044764
NTM	ITGA11	0.866427387	NTM	ADAM12	0.870380662
NTM	COL11A1	0.911655306	NTM	INHBA	0.881260804
NTM	COL5A2	0.86673342	NTM	ITGA11	0.902264652
NTM	CTSK	0.899120892	NTM	ISM1	0.86920554
ITGA4	RCSD1	0.851408475	NTM	COL11A1	0.917425175
ITGA4	GIMAP6	0.85723468	NTM	POSTN	0.875071997
SAA2	SAA1	0.885823748	NTM	THBS2	0.885422564
TPSB2	TPSAB1	0.963050467	NTM	COL5A2	0.86306146
GAPT	GPR65	0.865337386	S1PR4	CD3E	0.866935878
GAPT	TLR7	0.858838568	ITGA4	FLI1	0.865320408
GAPT	P2RY13	0.887967121	ITGA4	TLR4	0.857074258
GAPT	TAGAP	0.861774277	SAA2	SAA1	0.862401573
GAPT	LAT2	0.878124431	TPSB2	TPSAB1	0.970449203
GAPT	APBB1IP	0.882273445	GAPT	P2RY13	0.873821777
GAPT	CX3CR1	0.851253308	GPR65	BTK	0.8587332
GPR65	CD180	0.908798028	GPR65	TLR7	0.885953086
GPR65	NCF1	0.866196485	GPR65	LILRB1	0.858182912
GPR65	BTK	0.910833665	GPR65	SAMSN1	0.875817811
GPR65	CCR5	0.857035446	GPR65	FYB1	0.873387498
GPR65	TLR7	0.917791229	GPR65	LCP2	0.873877961
GPR65	RCSD1	0.869396987	GPR65	APBB1IP	0.861575599
GPR65	P2RY13	0.85293259	GPR65	CD86	0.874141198
GPR65	LILRB1	0.85928469	GPR65	MNDA	0.864407623
GPR65	TAGAP	0.877817778	GPR65	ADAP2	0.858400485
GPR65	SLA	0.86933003	GPR65	MS4A7	0.865127628
GPR65	ARHGAP9	0.85035195	GPR65	HAVCR2	0.893558837
GPR65	CD84	0.866270198	GPR65	GPR34	0.859585479
GPR65	SIGLEC9	0.886138688	GPR65	CCR1	0.859984578
GPR65	BIN2	0.881573246	GPR65	MSR1	0.863593316
GPR65	NCKAP1L	0.88656144	GPR65	MS4A6A	0.872082833
GPR65	SAMSN1	0.899736966	GPR65	EVI2B	0.886217359
GPR65	CYTH4	0.877788567	GPR65	C3AR1	0.867144297
GPR65	FCGR1A	0.886517873	GPR65	GIMAP4	0.875030222
GPR65	TMIGD3	0.870923035	GPR65	CD53	0.860218174
GPR65	PTPRC	0.901211384	GPR65	FCGR3A	0.857658036
GPR65	LAT2	0.895676905	SPRR2F	STAR	0.88429633
GPR65	FYB1	0.892277319	SPRR2F	GATA4	0.915021985
GPR65	IL10RA	0.87656793	SPRR2F	PEG3	0.861179795
GPR65	GIMAP6	0.899002332	H2AC14	H2BC10	0.859517753
GPR65	LILRB4	0.872355062	STAR	FOXL2	0.885865923
GPR65	LCP2	0.912919341	STAR	GATA4	0.917902551
GPR65	CD300A	0.879717546	STAR	PEG3	0.8912398
GPR65	LAIR1	0.891126831	TCF21	GATA4	0.85112518
GPR65	CD48	0.869160664	ZNF683	XCL2	0.856314099
GPR65	APBB1IP	0.92868488	ZNF683	CTSW	0.89491445
GPR65	CD86	0.910504909	H2BC10	H1-5	0.87792119
GPR65	TNFAIP8L2	0.913648816	H2BC10	H4C4	0.905547151
GPR65	NCF4	0.857857098	H2BC10	H3C7	0.853402823

GPR65	MNDA	0.910079996	IKZF1	TAGAP	0.85891979
GPR65	ADAP2	0.889718009	IKZF1	ITGAL	0.854639964
GPR65	LRRC25	0.888844622	IKZF1	NCKAP1L	0.86098091
GPR65	MS4A7	0.869637121	IKZF1	IL10RA	0.852183768
GPR65	HAVCR2	0.920941037	C3orf80	FAP	0.898889364
GPR65	GPR34	0.909097858	C3orf80	ADAMTS12	0.86959812
GPR65	SASH3	0.920720924	C3orf80	ADAM12	0.869120257
GPR65	HCK	0.85904848	C3orf80	COLEC12	0.85871994
GPR65	CCR1	0.861613039	C3orf80	INHBA	0.8518792
GPR65	MSR1	0.876680782	C3orf80	VCAM1	0.854507213
GPR65	ABI3	0.879891531	C3orf80	C1QTNF6	0.854093764
GPR65	FGL2	0.854455015	C3orf80	VCAN	0.851675794
GPR65	MS4A6A	0.887167019	C3orf80	TMEM158	0.858177921
GPR65	WAS	0.851528519	C3orf80	POSTN	0.875343019
GPR65	CD37	0.861714288	C3orf80	COL5A2	0.904752165
GPR65	PLEK	0.87346216	C3orf80	COL6A3	0.869028423
GPR65	LY86	0.860667865	C3orf80	COL3A1	0.856210808
GPR65	EVI2B	0.898680226	PIK3R5	NCKAP1L	0.85847226
GPR65	FERMT3	0.858267818	PIK3R5	IL10RA	0.874719819
GPR65	C3AR1	0.903138904	MYCT1	ADGRL4	0.852666115
GPR65	GIMAP4	0.909513813	ZNF763	ZNF844	0.854723337
GPR65	CYBB	0.871425255	OMD	ECM2	0.853668797
GPR65	RNASE6	0.880296866	H2BC13	H1-5	0.859897723
GPR65	CSF1R	0.860842408	ZNF549	ZNF419	0.851586594
GPR65	CD53	0.905720852	ZNF549	ZNF134	0.905173075
GPR65	CD4	0.858349055	ZEB2	HEPH	0.850060649
GPR65	AIF1	0.865954726	ZEB2	ARHGEF6	0.857421057
GPR65	FCGR3A	0.877783797	ZEB2	ANTXR2	0.873359472
GPR65	SPI1	0.857885335	ZEB2	ARHGAP31	0.86907839
GPR65	LAPTM5	0.884813982	ZEB2	IRAG1	0.866994725
ZNF527	ZNF569	0.852241271	ZEB2	DAB2	0.880440328
H2AC14	H2BC10	0.859292652	TMEM200A	MEIS3	0.874294831
H2AC14	H3C11	0.866868052	TMEM200A	ZEB1	0.877396613
H2AC14	H1-5	0.871982291	TMEM200A	HEPH	0.879606124
STAR	EFCC1	0.886152101	TMEM200A	ANTXR2	0.926628155
STAR	FOXL2	0.934308629	TMEM200A	FRMD6	0.85117934
TCF21	FOXL2	0.889356298	TMEM200A	IRAG1	0.895903562
ZNF683	CD3E	0.863438373	TMEM200A	CLMP	0.918625866
ZNF683	IL2RB	0.853683253	TMEM200A	PRRX1	0.850192639
H2BC10	H3C11	0.917820778	TMEM200A	MSRB3	0.877217372
H2BC10	H1-5	0.903187585	TMEM200A	OLFML1	0.858954029
H2BC10	H4C4	0.85470301	TMEM200A	GLT8D2	0.869883957
H2BC10	H1-4	0.889239183	TMEM200A	GASK1B	0.856752234
LY6G5B	DDX39B	0.850852284	TMEM200A	SRPX2	0.850262268
IKZF1	BTK	0.878647716	TMEM200A	CDH11	0.910071983
IKZF1	CCR5	0.900051423	TMEM200A	FILIP1L	0.879840182
IKZF1	RCSD1	0.85003654	TMEM200A	EMILIN1	0.854663844
IKZF1	CSF2RB	0.85246394	DRC1	ENKUR	0.902233555
IKZF1	DOCK8	0.850861328	DRC1	RSPH4A	0.88674172
IKZF1	LILRB1	0.865145255	DRC1	ARMC3	0.896299457
IKZF1	TAGAP	0.91410405	DRC1	CCDC96	0.854081951
IKZF1	SLA	0.85808939	DRC1	CFAP45	0.906259415
IKZF1	NFAM1	0.862176873	DRC1	WDR38	0.870341238
IKZF1	CD84	0.901076912	DRC1	ZMYND10	0.862780685
IKZF1	BIN2	0.896940143	DRC1	C5orf49	0.872069833
IKZF1	ITGAL	0.871544037	CD180	BTK	0.913772574

IKZF1	NCKAP1L	0.892746896	CD180	TLR7	0.912086901
IKZF1	SAMSN1	0.850652607	CD180	RCSD1	0.850425429
IKZF1	ITGAM	0.864168194	CD180	CD300C	0.875006464
IKZF1	PTPRC	0.921405112	CD180	SIGLEC7	0.86049558
IKZF1	FYB1	0.884229558	CD180	LILRB1	0.889914199
IKZF1	IL10RA	0.860006043	CD180	TAGAP	0.856961738
IKZF1	LCP2	0.884885996	CD180	SLA	0.89270768
IKZF1	LAIR1	0.850721916	CD180	ARHGAP9	0.850192207
IKZF1	APBB1IP	0.885876603	CD180	CD84	0.870422162
IKZF1	CD86	0.865036845	CD180	SIGLEC9	0.917694623
IKZF1	SLAMF8	0.851352892	CD180	LILRB2	0.874737803
IKZF1	HAVCR2	0.861959097	CD180	BIN2	0.895579508
IKZF1	SASH3	0.87300932	CD180	SLC37A2	0.858210009
IKZF1	HCK	0.853989295	CD180	ITGAL	0.872854042
IKZF1	MPEG1	0.870860876	CD180	NCKAP1L	0.921899731
IKZF1	WAS	0.870643882	CD180	FGR	0.854552927
IKZF1	PLEK	0.864283144	CD180	SAMSN1	0.879115633
IKZF1	EVI2B	0.887000658	CD180	CYTH4	0.903577446
IKZF1	FERMT3	0.857060878	CD180	FCGR1A	0.865849747
IKZF1	CYBB	0.876058374	CD180	PTPRC	0.891869528
IKZF1	CSF1R	0.855934634	CD180	LAT2	0.858507215
IKZF1	CD53	0.859485089	CD180	TRPV2	0.851619924
IKZF1	CD4	0.864412299	CD180	FYB1	0.863153159
C3orf80	KCNE4	0.867153001	CD180	IL10RA	0.880618974
C3orf80	FAP	0.890486479	CD180	GIMAP6	0.880649346
C3orf80	ASPN	0.858294203	CD180	LILRB4	0.903128352
C3orf80	ADAM12	0.910827051	CD180	LCP2	0.926221481
C3orf80	FBN1	0.878774557	CD180	CD300A	0.879358866
C3orf80	ADAMTS2	0.857738337	CD180	LAIR1	0.915452141
C3orf80	COL11A1	0.883106752	CD180	NCF2	0.859453769
C3orf80	LOX	0.885377582	CD180	APBB1IP	0.894979069
C3orf80	POSTN	0.862014319	CD180	CD86	0.888436442
C3orf80	THBS2	0.875999117	CD180	NCF4	0.865007061
C3orf80	FN1	0.867530069	CD180	MNDA	0.859253074
C3orf80	COL3A1	0.877039657	CD180	ADAP2	0.89450746
PIK3R5	BTK	0.912424654	CD180	SLCO2B1	0.873691407
PIK3R5	TLR7	0.851466588	CD180	LRRC25	0.881470486
PIK3R5	RCSD1	0.880875577	CD180	HAVCR2	0.902602557
PIK3R5	CD300C	0.873708584	CD180	SASH3	0.917113761
PIK3R5	SIGLEC7	0.866530405	CD180	HCK	0.89733283
PIK3R5	CSF2RB	0.880987929	CD180	CCR1	0.872301931
PIK3R5	DOCK8	0.879642527	CD180	ABI3	0.857342779
PIK3R5	TAGAP	0.870929867	CD180	MS4A6A	0.852285311
PIK3R5	FAM78A	0.914364407	CD180	WAS	0.854455216
PIK3R5	ARHGAP9	0.850906218	CD180	CD37	0.86276885
PIK3R5	NFAM1	0.882087143	CD180	LPXN	0.855753521
PIK3R5	CD84	0.906526742	CD180	PLEK	0.872886096
PIK3R5	SIGLEC9	0.909739888	CD180	EVI2B	0.877765042
PIK3R5	TLR4	0.863878513	CD180	FERMT3	0.91056913
PIK3R5	BIN2	0.882733404	CD180	SELPLG	0.896032874
PIK3R5	SLC37A2	0.908128545	CD180	C3AR1	0.915861133
PIK3R5	TBXAS1	0.869810681	CD180	FCGR2A	0.886961075
PIK3R5	ARHGEF6	0.891441422	CD180	CYBB	0.907539313
PIK3R5	NCKAP1L	0.907169566	CD180	ITGB2	0.859185133
PIK3R5	ITGAM	0.908318335	CD180	CSF1R	0.87678724
PIK3R5	CYTH4	0.907994755	CD180	CD53	0.894185126

PIK3R5	MYO1F	0.874886943	CD180	FCGR3A	0.876337292
PIK3R5	ITGAX	0.855422506	CD180	LAPTM5	0.889741544
PIK3R5	LAT2	0.850090141	PDCD1LG2	STX11	0.856332253
PIK3R5	TRPV2	0.857877277	PDCD1LG2	PTPRC	0.877696473
PIK3R5	IL10RA	0.910488267	PDCD1LG2	FPR3	0.871447666
PIK3R5	GIMAP6	0.884090517	NCF1	SIGLEC10	0.853872431
PIK3R5	LILRB4	0.862474453	NCF1	BIN2	0.858753017
PIK3R5	CD300A	0.864730367	NCF1	LILRB4	0.886362345
PIK3R5	LAIR1	0.889136161	NCF1	LCP2	0.860641291
PIK3R5	APBB1IP	0.882154629	NCF1	CD86	0.855000381
PIK3R5	ADAP2	0.889062757	NCF1	TNFAIP8L2	0.865249513
PIK3R5	SLCO2B1	0.925249931	NCF1	NCF4	0.86950309
PIK3R5	LRRC25	0.884633817	NCF1	SASH3	0.875148193
PIK3R5	SASH3	0.887300827	NCF1	ABI3	0.902270531
PIK3R5	HCK	0.893444934	NCF1	CD37	0.861675736
PIK3R5	MPEG1	0.882961762	NCF1	IL2RG	0.857432174
PIK3R5	WAS	0.850660043	NCF1	FERMT3	0.861801456
PIK3R5	PLEK	0.869911299	NCF1	SELPLG	0.851377612
PIK3R5	SH2B3	0.85526968	NCF1	CD53	0.867845819
PIK3R5	CD163	0.850835986	NCF1	AIF1	0.857447393
PIK3R5	FERMT3	0.888103821	NCF1	SPI1	0.869459318
PIK3R5	C3AR1	0.874219107	NCF1	C1QA	0.861463385
PIK3R5	CYBB	0.876303008	BTK	PTPN7	0.868715231
PIK3R5	ITGB2	0.857827956	BTK	CCR5	0.87288501
PIK3R5	CSF1R	0.912132716	BTK	TLR7	0.911186519
PIK3R5	CD4	0.860651741	BTK	RCSD1	0.877401835
OMD	FAP	0.877190793	BTK	RASAL3	0.853410637
OMD	LRRC15	0.892883144	BTK	LILRB1	0.935393605
OMD	ITGA11	0.878508438	BTK	TAGAP	0.904778921
OMD	COL11A1	0.854586599	BTK	SLA	0.895323387
OMD	COL8A1	0.8511328	BTK	ARHGAP9	0.909294923
H2BC13	H1-5	0.874511671	BTK	CD84	0.898768877
ZEB2	IFFO1	0.883657087	BTK	SIGLEC9	0.891404676
ZEB2	ANTXR2	0.889658618	BTK	SIGLEC10	0.876899888
ZEB2	PRDM1	0.874834206	BTK	LILRB2	0.893915252
ZEB2	CCN4	0.855697944	BTK	STX11	0.854619385
ZEB2	GLIPR1	0.875469411	BTK	CLEC4A	0.890399813
ZEB2	DAB2	0.85256783	BTK	BIN2	0.941173045
ZEB2	ITGA5	0.85464897	BTK	SLC37A2	0.873375368
TMEM200A	CLMP	0.867104465	BTK	TBXAS1	0.879769406
TMEM200A	OLFML1	0.854958891	BTK	ITGAL	0.895198321
DRC1	C2orf50	0.882681555	BTK	NCKAP1L	0.942249702
DRC1	ENKUR	0.859451876	BTK	FGR	0.886980226
DRC1	RSPH4A	0.899228691	BTK	AOAH	0.923171811
DRC1	C7orf57	0.883413166	BTK	SAMSN1	0.888442172
DRC1	ARMC3	0.880989858	BTK	CYTH4	0.902232879
DRC1	CFAP45	0.860808054	BTK	ITGAX	0.856513181
DRC1	ZMYND10	0.897963455	BTK	PTPRC	0.93897629
DRC1	C5orf49	0.87111286	BTK	LAT2	0.883502812
DRC1	FAM183A	0.852689481	BTK	TRPV2	0.852263333
DRC1	TEKT2	0.850686701	BTK	FYB1	0.909496552
CD180	BTK	0.925763199	BTK	IL10RA	0.933005502
CD180	TLR7	0.889776023	BTK	GIMAP6	0.871315442
CD180	CD300C	0.881510596	BTK	LILRB4	0.911564253
CD180	TAGAP	0.894387256	BTK	LCP2	0.940054744
CD180	SLA	0.855328695	BTK	CD300A	0.876609019

CD180	CD84	0.88229516	BTK	LAIR1	0.921774909
CD180	SIGLEC9	0.919958807	BTK	TLR2	0.87758065
CD180	TLR4	0.862261021	BTK	NCF2	0.862534944
CD180	BIN2	0.875059761	BTK	APBB1IP	0.900480906
CD180	SLC37A2	0.853402575	BTK	CD86	0.912658757
CD180	NCKAP1L	0.914697764	BTK	SLAMF8	0.857060164
CD180	SAMSN1	0.871009354	BTK	NCF4	0.893236683
CD180	ITGAM	0.887777862	BTK	MNDA	0.882987171
CD180	CYTH4	0.903236397	BTK	ADAP2	0.898988302
CD180	TMIGD3	0.882621169	BTK	SLCO2B1	0.855255578
CD180	PTPRC	0.873097161	BTK	LRRC25	0.867595213
CD180	LAT2	0.878839787	BTK	HAVCR2	0.914461005
CD180	IL10RA	0.861890586	BTK	SASH3	0.94443659
CD180	GIMAP6	0.86287982	BTK	HCK	0.924359595
CD180	LILRB4	0.861983775	BTK	CCR1	0.858279327
CD180	LCP2	0.895884948	BTK	ABI3	0.87943187
CD180	CD300A	0.909780891	BTK	FGL2	0.87501682
CD180	LAIR1	0.890690953	BTK	MPEG1	0.879387974
CD180	APBB1IP	0.916107962	BTK	MS4A6A	0.909107057
CD180	CD86	0.888714094	BTK	WAS	0.865644724
CD180	TNFAIP8L2	0.863248988	BTK	CD37	0.875309053
CD180	NCF4	0.862624849	BTK	LPXN	0.89961516
CD180	MNDA	0.902654418	BTK	PLEK	0.919181043
CD180	ADAP2	0.888727568	BTK	EVI2B	0.915828633
CD180	SLCO2B1	0.85097658	BTK	FERMT3	0.910601442
CD180	LRRC25	0.888822951	BTK	SELPLG	0.891418803
CD180	HAVCR2	0.910253147	BTK	C3AR1	0.89720722
CD180	GPR34	0.898700065	BTK	FCGR2A	0.872140791
CD180	SASH3	0.909780457	BTK	GIMAP4	0.874213362
CD180	HCK	0.878871421	BTK	CYBB	0.930215049
CD180	CCR1	0.867114196	BTK	ITGB2	0.871487597
CD180	MS4A6A	0.864806028	BTK	CSF1R	0.857471079
CD180	PLEK	0.893395129	BTK	CD53	0.928024137
CD180	EVI2B	0.873636028	BTK	FCGR3A	0.865398052
CD180	FERMT3	0.86542964	BTK	LAPTM5	0.889022737
CD180	C3AR1	0.907900086	BTK	C1QC	0.857228122
CD180	FCGR2A	0.852729244	GPR89B	GPR89A	0.868858971
CD180	CYBB	0.886893834	PTPN7	RASAL3	0.872500047
CD180	ITGB2	0.86147357	PTPN7	LILRB1	0.857609239
CD180	RNASE6	0.87181794	PTPN7	ARHGAP9	0.872868931
CD180	CSF1R	0.891066654	PTPN7	BIN2	0.885707688
CD180	CD53	0.892331517	PTPN7	ITGAL	0.884629421
CD180	CD4	0.87533602	PTPN7	LCP2	0.86627512
CD180	VSIG4	0.852147909	PTPN7	SASH3	0.868669839
CD180	FCGR3A	0.850262791	CCR5	TLR7	0.860170035
CD180	LAPTM5	0.875950261	CCR5	RASAL3	0.862080354
NCF1	BTK	0.914476785	CCR5	LILRB1	0.888117798
NCF1	CCR5	0.866773575	CCR5	TAGAP	0.906343867
NCF1	P2RY13	0.869377353	CCR5	CYTIP	0.850853294
NCF1	LILRB1	0.872472133	CCR5	SIGLEC10	0.898026348
NCF1	TAGAP	0.871668282	CCR5	LILRB2	0.851722682
NCF1	GPR84	0.885743766	CCR5	STX11	0.858394093
NCF1	ARHGAP9	0.885303306	CCR5	BIN2	0.902339337
NCF1	NFAM1	0.879961603	CCR5	ITGAL	0.92050734
NCF1	CD84	0.862462184	CCR5	NCKAP1L	0.866274702
NCF1	SIGLEC9	0.860138954	CCR5	AOAH	0.850591328

NCF1	FCGR2B	0.866788348	CCR5	PTPRC	0.918395104
NCF1	BIN2	0.891052441	CCR5	CD3E	0.858869708
NCF1	NCKAP1L	0.859962658	CCR5	FYB1	0.921689006
NCF1	ITGAM	0.854547959	CCR5	IL10RA	0.870174634
NCF1	CYTH4	0.88902824	CCR5	GIMAP6	0.868549799
NCF1	MYO1F	0.87144914	CCR5	LILRB4	0.850887584
NCF1	ITGAX	0.876555738	CCR5	LCP2	0.934454914
NCF1	PTPRC	0.86934643	CCR5	CD2	0.871016138
NCF1	LAT2	0.903851851	CCR5	LAIR1	0.858026121
NCF1	FYB1	0.873466949	CCR5	CD86	0.880886774
NCF1	IL10RA	0.86935937	CCR5	SLAMF8	0.873655579
NCF1	LILRB4	0.932466135	CCR5	HAVCR2	0.8585192
NCF1	LCP2	0.887291592	CCR5	SASH3	0.887407045
NCF1	LAIR1	0.894309745	CCR5	CCR1	0.870110701
NCF1	CD48	0.857246264	CCR5	IL2RG	0.87731508
NCF1	APBB1IP	0.897092744	CCR5	EVI2B	0.885495906
NCF1	CD86	0.878417794	CCR5	GIMAP4	0.883844841
NCF1	TNFAIP8L2	0.901097554	ENKUR	RSPH4A	0.898990313
NCF1	NCF4	0.867405062	ENKUR	C7orf57	0.850212943
NCF1	LRRC25	0.868029653	ENKUR	PRR29	0.903022002
NCF1	HAVCR2	0.864419773	ENKUR	ARMC3	0.893019256
NCF1	SASH3	0.89387115	ENKUR	CCDC96	0.883943841
NCF1	HCK	0.8636937	ENKUR	MORN5	0.90953462
NCF1	ABI3	0.894393461	ENKUR	ROPN1L	0.881345472
NCF1	WAS	0.889527775	ENKUR	CFAP45	0.894979271
NCF1	CD37	0.886633866	ENKUR	WDR38	0.916739024
NCF1	PLEK	0.860195527	ENKUR	ZMYND10	0.872450269
NCF1	EVI2B	0.852448213	ENKUR	C5orf49	0.93274161
NCF1	FERMT3	0.883609362	ENKUR	FAM183A	0.86789307
NCF1	SELPLG	0.854052261	ENKUR	C20orf85	0.872236707
NCF1	CYBB	0.861463698	FAM81B	RSPH4A	0.858397352
NCF1	CD53	0.874365697	FAM81B	CAPSL	0.850197736
NCF1	CD4	0.863214967	FAM81B	CFAP45	0.850659566
NCF1	AIF1	0.855086197	ZNF222	ZNF45	0.878368215
NCF1	SPI1	0.898460368	RSPH4A	C7orf57	0.851223202
BTK	CCR5	0.858158436	RSPH4A	PRR29	0.930491243
BTK	TLR7	0.893077336	RSPH4A	ARMC3	0.896831167
BTK	RCSD1	0.886567444	RSPH4A	CCDC96	0.867624004
BTK	P2RY13	0.894975571	RSPH4A	MORN5	0.884546413
BTK	CD300C	0.926560717	RSPH4A	LRRC46	0.859848638
BTK	SIGLEC7	0.854085465	RSPH4A	CAPSL	0.858100476
BTK	CSF2RB	0.857851672	RSPH4A	CFAP45	0.922374398
BTK	LILRB1	0.898159571	RSPH4A	WDR38	0.873285254
BTK	TAGAP	0.916005012	RSPH4A	CCDC65	0.880394087
BTK	GPR84	0.872394988	RSPH4A	ZMYND10	0.917848427
BTK	FAM78A	0.850423893	RSPH4A	C5orf49	0.877541519
BTK	SLA	0.913716203	RSPH4A	PIFO	0.871831596
BTK	ARHGAP9	0.912937349	RSPH4A	C20orf85	0.879380771
BTK	NFAM1	0.925783786	RSPH4A	TEKT2	0.867562305
BTK	CD84	0.939333154	GIMAP8	RCSD1	0.903618707
BTK	SIGLEC9	0.959638732	GIMAP8	NCKAP1L	0.89031843
BTK	LILRB2	0.888388614	GIMAP8	CYTH4	0.853066786
BTK	CLEC4A	0.876736681	GIMAP8	IL10RA	0.878699946
BTK	TLR4	0.883757191	GIMAP8	GIMAP6	0.947887972
BTK	FCGR2B	0.900986626	GIMAP8	LCP2	0.863365144
BTK	BIN2	0.943509888	GIMAP8	GIMAP7	0.866589788

BTK	SLC37A2	0.90303705	GIMAP8	SLCO2B1	0.867453112
BTK	TBXAS1	0.922205743	GIMAP8	MS4A4A	0.853138388
BTK	CSF2RA	0.888775617	GIMAP8	MPEG1	0.858879446
BTK	ARHGEF6	0.87787936	GIMAP8	CD163	0.894474315
BTK	IRF8	0.860722589	TLR7	RCSD1	0.880130597
BTK	NCKAP1L	0.950001263	TLR7	LILRB1	0.915560863
BTK	FGR	0.89158871	TLR7	TAGAP	0.887663765
BTK	SAMSN1	0.923131129	TLR7	SLA	0.886641119
BTK	CLEC7A	0.852913686	TLR7	CD84	0.923298024
BTK	ITGAM	0.953251879	TLR7	SIGLEC9	0.867028382
BTK	CYTH4	0.942902205	TLR7	BIN2	0.909952344
BTK	MYO1F	0.911058703	TLR7	SLC37A2	0.873732002
BTK	FCGR1A	0.854003195	TLR7	TBXAS1	0.874278746
BTK	ITGAX	0.908453266	TLR7	ITGAL	0.869326573
BTK	TMIGD3	0.879002274	TLR7	NCKAP1L	0.949026988
BTK	PTPRC	0.926989177	TLR7	AOAH	0.916980848
BTK	LAT2	0.936525648	TLR7	SAMSN1	0.901356247
BTK	FYB1	0.876233616	TLR7	CYTH4	0.899964989
BTK	IL10RA	0.939185517	TLR7	PTPRC	0.907981353
BTK	GIMAP6	0.878799234	TLR7	LAT2	0.889834964
BTK	LILRB4	0.925536177	TLR7	FYB1	0.910574215
BTK	OSCAR	0.869598783	TLR7	IL10RA	0.918562589
BTK	GNA15	0.892940628	TLR7	GIMAP6	0.878779848
BTK	LCP2	0.912876435	TLR7	LILRB4	0.860338737
BTK	CD300A	0.913663427	TLR7	LCP2	0.928365312
BTK	LAIR1	0.958588539	TLR7	LAIR1	0.895424211
BTK	TLR2	0.858026237	TLR7	APBB1IP	0.933759196
BTK	NCF2	0.870159927	TLR7	CD86	0.902127193
BTK	APBB1IP	0.940383613	TLR7	MNDA	0.895735272
BTK	CD86	0.936571668	TLR7	ADAP2	0.93752988
BTK	TNFAIP8L2	0.924765764	TLR7	SLCO2B1	0.912927972
BTK	NCF4	0.917323442	TLR7	MS4A7	0.883913799
BTK	MNDA	0.907997267	TLR7	HAVCR2	0.901850134
BTK	ADAP2	0.920020669	TLR7	GPR34	0.877511236
BTK	SLCO2B1	0.889319238	TLR7	SASH3	0.900631331
BTK	LRRC25	0.922451042	TLR7	HCK	0.867664612
BTK	MS4A7	0.8775077	TLR7	CCR1	0.856730126
BTK	HAVCR2	0.939968788	TLR7	MSR1	0.85662227
BTK	GPR34	0.912521722	TLR7	FGL2	0.877590182
BTK	SASH3	0.95244977	TLR7	MPEG1	0.890513228
BTK	HCK	0.938739557	TLR7	MS4A6A	0.883589955
BTK	ABI3	0.908112222	TLR7	PLEK	0.880039986
BTK	FGL2	0.859446453	TLR7	EVI2B	0.914654797
BTK	MPEG1	0.890627526	TLR7	SELPLG	0.879861018
BTK	MS4A6A	0.920055031	TLR7	C3AR1	0.924508174
BTK	WAS	0.913207573	TLR7	FCGR2A	0.89353251
BTK	CD37	0.903551727	TLR7	GIMAP4	0.855328626
BTK	PLEK	0.922710971	TLR7	CYBB	0.932543325
BTK	CD163	0.884887681	TLR7	CSF1R	0.905541756
BTK	EVI2B	0.915509222	TLR7	CD53	0.883048326
BTK	FPR3	0.860011327	TLR7	FCGR3A	0.877930911
BTK	FERMT3	0.932571116	TLR7	LAPTM5	0.885644929
BTK	SELPLG	0.898478623	C7orf57	CCDC96	0.86893777
BTK	C3AR1	0.937367679	C7orf57	ROPN1L	0.917357436
BTK	FCGR2A	0.897581411	C7orf57	CFAP45	0.870446767
BTK	GIMAP4	0.865186798	C7orf57	WDR38	0.885977058

BTK	LSP1	0.870828555	C7orf57	ZMYND10	0.892262637
BTK	CYBB	0.944301699	C7orf57	C5orf49	0.854139356
BTK	ITGB2	0.916453754	HK3	SIGLEC9	0.852699106
BTK	RNASE6	0.901699245	RCSD1	FLI1	0.896019784
BTK	CSF1R	0.934274424	RCSD1	CSF2RB	0.856801065
BTK	CD53	0.932393256	RCSD1	SLC9A9	0.870081814
BTK	CD4	0.930284854	RCSD1	LILRB1	0.87303919
BTK	ALOX5AP	0.886646334	RCSD1	TAGAP	0.852925485
BTK	AIF1	0.899511436	RCSD1	CD84	0.89866541
BTK	VSIG4	0.865388386	RCSD1	TLR4	0.867703237
BTK	FCGR3A	0.859173927	RCSD1	BIN2	0.859515608
BTK	SPI1	0.909294943	RCSD1	ARHGEF6	0.904757598
BTK	CD14	0.887628743	RCSD1	ITGAL	0.856150484
BTK	LAPTM5	0.91058486	RCSD1	NCKAP1L	0.933403843
BTK	C1QC	0.850882269	RCSD1	AOAH	0.908191275
EFCC1	FOXL2	0.884606278	RCSD1	CYTH4	0.871254609
ZNF571	ZNF570	0.864983785	RCSD1	PTPRC	0.884416979
PTPN7	ARHGAP9	0.870818536	RCSD1	IL10RA	0.902905761
CCR5	RCSD1	0.857344613	RCSD1	GIMAP6	0.919420803
CCR5	RASAL3	0.884846781	RCSD1	LCP2	0.879598405
CCR5	TAGAP	0.915559965	RCSD1	ADAP2	0.871778718
CCR5	SLA	0.855577583	RCSD1	SLCO2B1	0.902653827
CCR5	ARHGAP9	0.866071642	RCSD1	MS4A7	0.851658376
CCR5	CD84	0.865480911	RCSD1	MS4A4A	0.868037796
CCR5	BIN2	0.904215278	RCSD1	FGL2	0.858097073
CCR5	ITGAL	0.922584557	RCSD1	MPEG1	0.912792689
CCR5	NCKAP1L	0.873699143	RCSD1	MS4A6A	0.863630904
CCR5	SAMSN1	0.860079401	RCSD1	PLEK	0.882253716
CCR5	PTPRC	0.930538573	RCSD1	C3AR1	0.853990593
CCR5	FYB1	0.939424711	RCSD1	CYBB	0.893936503
CCR5	LCP2	0.906919868	PRR29	ARMC3	0.869603103
CCR5	GIMAP7	0.851394859	PRR29	CCDC96	0.861673971
CCR5	CD48	0.891123902	PRR29	MORN5	0.868933072
CCR5	APBB1IP	0.890222648	PRR29	LRRC10B	0.894540619
CCR5	CD86	0.867953693	PRR29	CFAP45	0.852016062
CCR5	SLAMF8	0.85197197	PRR29	WDR38	0.860760846
CCR5	MNDA	0.860976237	PRR29	CCDC65	0.891295512
CCR5	HAVCR2	0.861745869	PRR29	ZMYND10	0.859686873
CCR5	SASH3	0.889862316	PRR29	C5orf49	0.886593821
CCR5	FGL2	0.851203162	PRR29	C20orf85	0.869302717
CCR5	WAS	0.86977872	ADAMTS4	GEM	0.881661993
CCR5	CD37	0.875131924	FOXL2	GATA4	0.86466474
CCR5	IL2RG	0.855799737	FOXL2	PEG3	0.888779468
CCR5	EVI2B	0.903330152	ZNF765	ZNF813	0.851609257
CCR5	GIMAP4	0.867415282	ZNF765	ZNF28	0.879884723
CCR5	CD53	0.859835461	P2RY13	APBB1IP	0.859434828
ENKUR	RSPH4A	0.876430894	LRRC43	TEKT2	0.854621017
ENKUR	C7orf57	0.853498704	SLAMF7	CXCL9	0.858040494
ENKUR	MORN5	0.880682968	CD209	MRC1	0.860895858
ENKUR	LRRC46	0.85053572	GATA4	PEG3	0.854273491
ENKUR	CFAP45	0.882902342	ZNF697	DCHS1	0.856779526
ENKUR	C20orf85	0.879907447	CD300C	SIGLEC7	0.86477643
IGSF21	FCGR1A	0.866286813	CD300C	SIGLEC9	0.910897673
ZNF222	ZNF45	0.853987359	CD300C	SLC37A2	0.855214225
RSPH4A	C7orf57	0.874049233	CD300C	TBXAS1	0.885539847
RSPH4A	MORN5	0.850145834	CD300C	NCKAP1L	0.859180529

RSPH4A	CFAP45	0.866455646	CD300C	FGR	0.850597983
RSPH4A	ZMYND10	0.86579826	CD300C	CYTH4	0.881114119
RSPH4A	FAM183A	0.86376899	CD300C	LAT2	0.85045
GIMAP8	TLR7	0.854750974	CD300C	IL10RA	0.861257786
GIMAP8	RCSD1	0.890516334	CD300C	LILRB4	0.882261162
GIMAP8	ARHGEF6	0.875588379	CD300C	CD300A	0.903932758
GIMAP8	CYTH4	0.853894539	CD300C	LAIR1	0.890524903
GIMAP8	IL10RA	0.861939073	CD300C	NCF4	0.889440714
GIMAP8	GIMAP6	0.917752014	CD300C	LRRC25	0.862079546
GIMAP8	CSF1R	0.860286042	CD300C	HAVCR2	0.857114603
KNL1	BUB1B	0.874266702	CD300C	SASH3	0.8837929
KNL1	NUSAP1	0.876607156	CD300C	HCK	0.881808698
TLR7	RCSD1	0.86982241	CD300C	ABI3	0.850974167
TLR7	P2RY13	0.851187254	CD300C	MS4A6A	0.861736739
TLR7	MARCHF1	0.855724455	CD300C	WAS	0.85143908
TLR7	LILRB1	0.861676325	CD300C	FERMT3	0.900731034
TLR7	TAGAP	0.922207397	CD300C	SELPLG	0.869232818
TLR7	SLA	0.8559035	CD300C	C3AR1	0.885503596
TLR7	CD84	0.890299558	CD300C	CYBB	0.866974745
TLR7	SIGLEC9	0.877932672	CD300C	CSF1R	0.856953299
TLR7	TLR4	0.865244129	CD300C	CD53	0.87414462
TLR7	BIN2	0.881084611	CD300C	ALOX5AP	0.873914568
TLR7	ARHGEF6	0.862114454	CD300C	SPI1	0.858109824
TLR7	NCKAP1L	0.901438459	CD300C	LAPTM5	0.85787433
TLR7	ITGAM	0.85671397	GZMH	CD3D	0.879710706
TLR7	CYTH4	0.895039732	GZMH	CD3E	0.868768865
TLR7	MYO1F	0.85640737	GZMH	CD2	0.862987097
TLR7	FCGR1A	0.871613485	GZMH	GZMA	0.88772516
TLR7	PTPRC	0.879122595	GZMH	NKG7	0.914945475
TLR7	LAT2	0.871352438	GZMH	CCL5	0.861370584
TLR7	FYB1	0.87921378	SIGLEC7	SLA	0.85446716
TLR7	IL10RA	0.90292783	SIGLEC7	SIGLEC9	0.912642717
TLR7	GIMAP6	0.897618118	SIGLEC7	LILRB2	0.857340605
TLR7	LILRB4	0.86390216	SIGLEC7	LILRB4	0.88381293
TLR7	LCP2	0.877299943	SIGLEC7	CD300A	0.855177136
TLR7	LAIR1	0.887848187	SIGLEC7	LAIR1	0.853788811
TLR7	APBB1IP	0.924052441	SIGLEC7	HAVCR2	0.852930008
TLR7	CD86	0.893411769	SIGLEC7	FERMT3	0.853625093
TLR7	TNFAIP8L2	0.850045337	FLI1	CSF2RB	0.855455904
TLR7	MNDA	0.86365788	FLI1	SLC9A9	0.901967138
TLR7	ADAP2	0.905404913	FLI1	ARHGEF6	0.856154489
TLR7	SLCO2B1	0.867528544	FLI1	ARHGAP31	0.852299548
TLR7	LRRC25	0.875248118	FLI1	GIMAP6	0.851275041
TLR7	MS4A7	0.88041286	FLI1	WIPF1	0.866524616
TLR7	HAVCR2	0.877436008	FLI1	MPEG1	0.860135752
TLR7	GPR34	0.886533783	FLI1	PLEK	0.877947415
TLR7	SASH3	0.887101939	FLI1	DAB2	0.85670614
TLR7	HCK	0.860848157	FLI1	PECAM1	0.877532923
TLR7	FGL2	0.870272137	KCNE4	MEIS3	0.851123062
TLR7	MPEG1	0.858231997	KCNE4	FAP	0.893417427
TLR7	MS4A6A	0.859399393	KCNE4	CLMP	0.85921885
TLR7	WAS	0.851561004	KCNE4	INHBA	0.85717578
TLR7	EVI2B	0.853923973	KCNE4	CRISPLD2	0.878145748
TLR7	FERMT3	0.85030671	KCNE4	VCAN	0.88925355
TLR7	C3AR1	0.901367109	KCNE4	COL5A2	0.878321591
TLR7	GIMAP4	0.866722848	KCNE4	COL3A1	0.861835249

TLR7	CYBB	0.872361163	CSF2RB	LILRB1	0.853184269
TLR7	CSF1R	0.910882922	CSF2RB	TAGAP	0.858375775
TLR7	CD4	0.868612097	CSF2RB	CD84	0.903911895
TLR7	FCGR3A	0.875539442	CSF2RB	ITGAL	0.858345572
TLR7	LAPTM5	0.855396669	CSF2RB	NCKAP1L	0.869020171
C7orf57	ROPN1L	0.874051847	CSF2RB	AOAH	0.858915348
C7orf57	CFAP45	0.873026014	CSF2RB	PTPRC	0.911120914
C7orf57	ZMYND10	0.902299736	CSF2RB	IL10RA	0.891335424
C7orf57	FAM183A	0.858091738	CSF2RB	SLCO2B1	0.861300219
HK3	FGR	0.869617425	CSF2RB	MPEG1	0.88218984
HK3	ITGAM	0.869786211	CSF2RB	PLEK	0.87024793
RCSD1	DOCK8	0.88549753	CSF2RB	FPR3	0.855777033
RCSD1	TAGAP	0.869431794	CSF2RB	PECAM1	0.852109383
RCSD1	FAM78A	0.873362494	DIPK2B	ROBO4	0.91211969
RCSD1	CD84	0.87175403	DIPK2B	ADGRL4	0.855141028
RCSD1	TLR4	0.860035193	DIPK2B	CDH5	0.926841529
RCSD1	BIN2	0.88486741	DIPK2B	ESAM	0.873455094
RCSD1	ARHGEF6	0.931024403	ZNF799	ZNF443	0.919570519
RCSD1	ITGAL	0.852446558	RASAL3	TAGAP	0.879689043
RCSD1	NCKAP1L	0.904419046	RASAL3	ARHGAP9	0.945388988
RCSD1	CYTH4	0.872047957	RASAL3	BIN2	0.924392061
RCSD1	PTPRC	0.880018092	RASAL3	APOBR	0.85583869
RCSD1	IL10RA	0.906692236	RASAL3	ITGAL	0.93172349
RCSD1	GIMAP6	0.930888285	RASAL3	NCKAP1L	0.858449399
RCSD1	LCP2	0.852610923	RASAL3	CYTH4	0.889446602
RCSD1	LAIR1	0.859055799	RASAL3	MYO1F	0.867392895
RCSD1	APBB1IP	0.875355356	RASAL3	PTPRC	0.862750397
RCSD1	ADAP2	0.864264951	RASAL3	FYB1	0.861303675
RCSD1	SLCO2B1	0.875135892	RASAL3	IL10RA	0.872846609
RCSD1	SASH3	0.876551221	RASAL3	LCP2	0.887548873
RCSD1	FGL2	0.874928531	RASAL3	APBB1IP	0.860353755
RCSD1	MPEG1	0.868043718	RASAL3	SASH3	0.893392112
RCSD1	MS4A6A	0.853703405	RASAL3	WAS	0.877211405
RCSD1	PLEK	0.859942797	RASAL3	CD37	0.889361352
RCSD1	EVI2B	0.85162349	RASAL3	FERMT3	0.864300726
RCSD1	CYBB	0.861964001	RASAL3	SELPLG	0.874660918
RCSD1	CSF1R	0.871268377	ZXDA	ZXDB	0.883968798
PRR29	SPAG8	0.862842303	DACT3	DACT1	0.863889333
PRR29	LRRC46	0.862591124	DACT3	HIC1	0.873056258
PRR29	CFAP157	0.859862259	DOCK8	ARHGEF6	0.856743346
PRR29	CFAP45	0.866224987	ARMC3	MORN5	0.851749163
PRR29	SPEF1	0.876075618	ARMC3	CFAP45	0.882206959
FOXL2	C7	0.882248343	ARMC3	PIFO	0.890989968
ZNF765	ZNF347	0.851697928	ANKRD22	SLAMF8	0.851385421
ZNF765	ZNF845	0.880299209	SLC9A9	ARHGEF6	0.85429629
ZNF765	ZNF761	0.872028738	SLC9A9	NCKAP1L	0.87003979
P2RY13	CD84	0.851887459	SLC9A9	CYTH4	0.854198405
P2RY13	SIGLEC9	0.858613358	SLC9A9	GIMAP6	0.858305523
P2RY13	BIN2	0.869485557	SLC9A9	SLCO2B1	0.867573554
P2RY13	MYO1F	0.855825904	SLC9A9	MS4A4A	0.878360011
P2RY13	LAT2	0.894322779	SLC9A9	MPEG1	0.857947269
P2RY13	APBB1IP	0.88442431	SLC9A9	MS4A6A	0.851508279
P2RY13	TNFAIP8L2	0.876160846	SLC9A9	PLEK	0.884931693
P2RY13	MNDA	0.878131509	SLC9A9	CD163	0.870181862
P2RY13	CD37	0.852077745	SLC9A9	FCGR2A	0.875090803
P2RY13	EVI2B	0.85838618	SLC9A9	CYBB	0.860647569

P2RY13	CD4	0.859230777	SLC9A9	HMOX1	0.862090708
SLAMF7	CXCL9	0.853758166	CKMT1A	CKMT1B	0.941443755
MARCHF1	PTPRC	0.871076725	LILRB1	TAGAP	0.900264434
MARCHF1	LCP2	0.86175805	LILRB1	FAM78A	0.854660523
CD300C	NFAM1	0.867265755	LILRB1	SLA	0.907139576
CD300C	CD84	0.864572458	LILRB1	ARHGAP9	0.897830074
CD300C	SIGLEC9	0.932951322	LILRB1	NFAM1	0.85342719
CD300C	FCGR2B	0.868095901	LILRB1	CD84	0.925329495
CD300C	BIN2	0.863642258	LILRB1	SIGLEC9	0.882469541
CD300C	SLC37A2	0.852400555	LILRB1	SIGLEC10	0.913288203
CD300C	TBXAS1	0.872492189	LILRB1	LILRB2	0.916873773
CD300C	NCKAP1L	0.877421598	LILRB1	STX11	0.889817162
CD300C	FGR	0.89944107	LILRB1	BIN2	0.943114164
CD300C	SAMSN1	0.867242946	LILRB1	ITGAL	0.918496476
CD300C	ITGAM	0.930217042	LILRB1	NCKAP1L	0.939958935
CD300C	CYTH4	0.912648335	LILRB1	FGR	0.871456557
CD300C	MYO1F	0.85475359	LILRB1	AOAH	0.910975374
CD300C	ITGAX	0.874623843	LILRB1	SAMSN1	0.911186739
CD300C	PTPRC	0.854309929	LILRB1	CYTH4	0.915339119
CD300C	LAT2	0.879169998	LILRB1	ITGAX	0.85683848
CD300C	IL10RA	0.871158384	LILRB1	PTPRC	0.930462679
CD300C	FPR1	0.856782122	LILRB1	LAT2	0.859143618
CD300C	LILRB4	0.889285077	LILRB1	FYB1	0.937379193
CD300C	OSCAR	0.907611164	LILRB1	IL10RA	0.951809336
CD300C	GNA15	0.892038771	LILRB1	GIMAP6	0.877039838
CD300C	CD300A	0.926095817	LILRB1	LILRB4	0.920624878
CD300C	LAIR1	0.910730949	LILRB1	LCP2	0.941640633
CD300C	APBB1IP	0.865826807	LILRB1	LAIR1	0.932446779
CD300C	CD86	0.881556813	LILRB1	TLR2	0.885942037
CD300C	TNFAIP8L2	0.864429709	LILRB1	APBB1IP	0.925883121
CD300C	NCF4	0.890227383	LILRB1	CD86	0.909251584
CD300C	MNDA	0.862184843	LILRB1	NCF4	0.885998228
CD300C	ADAP2	0.866207662	LILRB1	MNDA	0.878060514
CD300C	SLCO2B1	0.857339562	LILRB1	ARHGAP30	0.853859048
CD300C	LRRC25	0.889466033	LILRB1	ADAP2	0.917548614
CD300C	HAVCR2	0.895155885	LILRB1	SLCO2B1	0.875297627
CD300C	GPR34	0.861731184	LILRB1	LRRC25	0.880634868
CD300C	SASH3	0.900771704	LILRB1	MS4A7	0.883862404
CD300C	HCK	0.900234506	LILRB1	HAVCR2	0.896953581
CD300C	ABI3	0.874060834	LILRB1	MS4A4A	0.869366711
CD300C	MS4A6A	0.884585448	LILRB1	SASH3	0.920564547
CD300C	CD37	0.850125084	LILRB1	HCK	0.907023673
CD300C	PLEK	0.873285702	LILRB1	CCR1	0.869652616
CD300C	CD163	0.860862173	LILRB1	ABI3	0.85992896
CD300C	FERMT3	0.887125294	LILRB1	MPEG1	0.896860722
CD300C	SELPLG	0.853494544	LILRB1	MS4A6A	0.883975598
CD300C	C3AR1	0.900868158	LILRB1	WAS	0.875486583
CD300C	FCGR2A	0.874709858	LILRB1	CD37	0.869409893
CD300C	CYBB	0.886907323	LILRB1	LPXN	0.851296071
CD300C	ITGB2	0.879544824	LILRB1	PLEK	0.9281594
CD300C	RNASE6	0.879149034	LILRB1	CD163	0.871456558
CD300C	CSF1R	0.888394982	LILRB1	EVI2B	0.92061013
CD300C	CD53	0.887699338	LILRB1	FERMT3	0.890361146
CD300C	CD4	0.854261815	LILRB1	SELPLG	0.88216664
CD300C	ALOX5AP	0.863092643	LILRB1	C3AR1	0.893345819
CD300C	AIF1	0.879770684	LILRB1	FCGR2A	0.888217119

CD300C	VSIG4	0.868413203	LILRB1	GIMAP4	0.867611485
CD300C	SPI1	0.877360073	LILRB1	CYBB	0.94040584
CD300C	CD14	0.87179593	LILRB1	ITGB2	0.864594281
CD300C	LAPTM5	0.861694974	LILRB1	CSF1R	0.882990131
GZMH	CD8A	0.884893023	LILRB1	CD53	0.905959897
GZMH	CD3D	0.905106403	LILRB1	CD4	0.851950131
GZMH	CD3E	0.872991827	LILRB1	FCGR3A	0.8905297
GZMH	CD2	0.88180408	LILRB1	LAPTM5	0.88793919
GZMH	GZMA	0.871394834	LILRB1	C1QB	0.852284375
GZMH	NKG7	0.873103229	LILRB1	C1QC	0.86139678
SIGLEC7	SIGLEC9	0.871673093	TAGAP	ARHGAP9	0.876098993
SIGLEC7	SLC37A2	0.880254207	TAGAP	CD84	0.889714127
SIGLEC7	IRF8	0.879243631	TAGAP	STX11	0.870288607
SIGLEC7	NPL	0.861219863	TAGAP	CD69	0.851196671
SIGLEC7	CD300A	0.853177868	TAGAP	BIN2	0.918598155
FLI1	AOAH	0.876435455	TAGAP	ITGAL	0.928355799
SHISA2	CLMP	0.874982563	TAGAP	NCKAP1L	0.907024101
KCNE4	ADAM12	0.887590547	TAGAP	AOAH	0.872017339
KCNE4	FBN1	0.889198967	TAGAP	SAMSN1	0.866282929
KCNE4	COL11A1	0.859409603	TAGAP	CYTH4	0.875072302
KCNE4	CRISPLD2	0.873588275	TAGAP	ITGAX	0.882419042
KCNE4	VCAN	0.859854514	TAGAP	PTPRC	0.936878841
KCNE4	LOX	0.866739982	TAGAP	FYB1	0.916442516
KCNE4	COL5A2	0.85974627	TAGAP	IL10RA	0.917922298
KCNE4	COL3A1	0.869880069	TAGAP	LILRB4	0.864074746
KCNE4	SPARC	0.859980184	TAGAP	LCP2	0.93014204
CSF2RB	NFAM1	0.874804529	TAGAP	LAIR1	0.858393304
CSF2RB	CD84	0.917144156	TAGAP	APBB1IP	0.890942868
CSF2RB	LILRB2	0.873427424	TAGAP	CD86	0.868258579
CSF2RB	CLEC4A	0.913183791	TAGAP	MNDA	0.866654871
CSF2RB	TLR4	0.879599565	TAGAP	ARHGAP30	0.858194963
CSF2RB	NCKAP1L	0.880175846	TAGAP	ADAP2	0.850301155
CSF2RB	ITGAM	0.883286521	TAGAP	HAVCR2	0.862219158
CSF2RB	CYTH4	0.869002624	TAGAP	SASH3	0.893711795
CSF2RB	ITGAX	0.860529535	TAGAP	HCK	0.852544743
CSF2RB	PTPRC	0.896315767	TAGAP	FGL2	0.888793789
CSF2RB	IL10RA	0.86374313	TAGAP	MPEG1	0.857182658
CSF2RB	LAIR1	0.872010282	TAGAP	PLEK	0.866828048
CSF2RB	NCF2	0.893093382	TAGAP	EVI2B	0.903640809
CSF2RB	SLAMF8	0.898139301	TAGAP	SELPLG	0.856521403
CSF2RB	SLCO2B1	0.862976831	TAGAP	CYBB	0.887230719
CSF2RB	HCK	0.914895461	TAGAP	CD53	0.851879236
CSF2RB	MPEG1	0.922654999	TAGAP	FCGR3A	0.852738843
CSF2RB	PLEK	0.889428861	GPR84	LAT2	0.899173442
CSF2RB	EVI2B	0.859011929	GPR84	LCP2	0.851969313
CSF2RB	FPR3	0.853450258	GPR84	CD86	0.855604574
CSF2RB	FERMT3	0.851677664	GPR84	HAVCR2	0.85017464
CSF2RB	C3AR1	0.851666894	GPR84	ABI3	0.85633539
CSF2RB	CYBB	0.871468275	GPR84	FERMT3	0.854450058
CSF2RB	ITGB2	0.886025805	GPR84	SPI1	0.860563854
CSF2RB	CSF1R	0.864368218	GPR84	LAPTM5	0.850054152
CSF2RB	CD4	0.865655872	ZNF234	ZNF227	0.908161858
CSF2RB	LYZ	0.863882682	ZNF234	ZNF45	0.850267716
ESCO2	PBK	0.88114734	FAM78A	ITGAL	0.852299351
HOXD3	HOXD4	0.936898837	FAM78A	NCKAP1L	0.89329541
DIPK2B	ROBO4	0.913565162	FAM78A	CYTH4	0.860313767

DIPK2B	CDH5	0.915833512	FAM78A	IL10RA	0.873250691
ZNF799	ZNF563	0.881705249	FAM78A	LCP2	0.850822713
ZNF799	ZNF44	0.88364962	FAM78A	HCK	0.869176857
ZNF799	ZNF443	0.919668745	LGALS7B	LGALS7	1
RASAL3	TAGAP	0.869668476	ZNF347	ZNF160	0.888013858
RASAL3	ARHGAP9	0.935805512	CXCR3	CD27	0.856718217
RASAL3	BIN2	0.913069329	CXCR3	CD3D	0.869700712
RASAL3	ITGAL	0.907496278	CXCR3	CD3E	0.919969768
RASAL3	CYTH4	0.859151378	CXCR3	CD2	0.913524175
RASAL3	MYO1F	0.885385522	CXCR3	IL2RG	0.870144768
RASAL3	LAT2	0.869918137	CXCR3	CCL5	0.850589357
RASAL3	FYB1	0.891325998	SLA	CD84	0.853278997
RASAL3	IL10RA	0.855924903	SLA	SIGLEC9	0.884722904
RASAL3	LCP2	0.878131174	SLA	SIGLEC10	0.87200247
RASAL3	APBB1IP	0.88304865	SLA	LILRB2	0.924995657
RASAL3	SASH3	0.88844687	SLA	BIN2	0.876574209
RASAL3	ABI3	0.902857245	SLA	TBXAS1	0.855809228
RASAL3	WAS	0.901230081	SLA	NCKAP1L	0.909421172
RASAL3	CD37	0.905528328	SLA	FGR	0.866802122
RASAL3	FERMT3	0.881442331	SLA	AOAH	0.897459867
RASAL3	GIMAP4	0.852062843	SLA	SAMSN1	0.895362876
RASAL3	SPI1	0.862158972	SLA	CYTH4	0.858199388
DOCK8	TAGAP	0.859115319	SLA	PTPRC	0.887150487
DOCK8	ARHGEF6	0.900134122	SLA	FYB1	0.880954432
ARMC3	TEKT2	0.856327215	SLA	IL10RA	0.908301241
SLC9A9	MS4A6A	0.857896925	SLA	GIMAP6	0.854308857
CKMT1A	CKMT1B	0.949430213	SLA	LILRB4	0.876264674
LILRB1	TAGAP	0.882908228	SLA	LCP2	0.905757942
LILRB1	SLA	0.903232179	SLA	CD300A	0.856677489
LILRB1	ARHGAP9	0.899247196	SLA	LAIR1	0.926803251
LILRB1	NFAM1	0.899774648	SLA	TLR2	0.888348204
LILRB1	CD84	0.903467258	SLA	APBB1IP	0.857102832
LILRB1	SIGLEC9	0.863833297	SLA	CD86	0.912658059
LILRB1	LILRB2	0.866938333	SLA	NCF4	0.874098993
LILRB1	BIN2	0.907961671	SLA	MNDA	0.868583571
LILRB1	ITGAL	0.863853338	SLA	ADAP2	0.878593911
LILRB1	NCKAP1L	0.898510296	SLA	SLCO2B1	0.861042646
LILRB1	SAMSN1	0.899128831	SLA	LRRC25	0.866007158
LILRB1	ITGAM	0.866030267	SLA	MS4A7	0.870181858
LILRB1	CYTH4	0.883605199	SLA	HAVCR2	0.90687321
LILRB1	MYO1F	0.856870018	SLA	MS4A4A	0.886067382
LILRB1	ITGAX	0.872011837	SLA	SASH3	0.905780279
LILRB1	PTPRC	0.903624576	SLA	HCK	0.887151018
LILRB1	LAT2	0.867146869	SLA	ABI3	0.85890774
LILRB1	FYB1	0.897911983	SLA	MS4A6A	0.882029624
LILRB1	IL10RA	0.885506226	SLA	WAS	0.857665951
LILRB1	LILRB4	0.901420225	SLA	PLEK	0.85725461
LILRB1	LCP2	0.88563201	SLA	CD163	0.888370723
LILRB1	LAIR1	0.90319009	SLA	EVI2B	0.868952999
LILRB1	APBB1IP	0.898474387	SLA	FERMT3	0.894166471
LILRB1	CD86	0.915021729	SLA	SELPLG	0.888195766
LILRB1	NCF4	0.863564249	SLA	C3AR1	0.887248491
LILRB1	ADAP2	0.859720349	SLA	FCGR2A	0.90553904
LILRB1	LRRC25	0.870770625	SLA	GIMAP4	0.861638722
LILRB1	MS4A7	0.852755732	SLA	TNFRSF1B	0.877345695
LILRB1	HAVCR2	0.889229572	SLA	CYBB	0.899023926

LILRB1	SASH3	0.887727628	SLA	CSF1R	0.861945191
LILRB1	HCK	0.851174313	SLA	CD53	0.906213843
LILRB1	ABI3	0.857195204	SLA	VSIG4	0.869594142
LILRB1	WAS	0.896758445	SLA	FCGR3A	0.877595904
LILRB1	CD37	0.873400443	SLA	LAPTM5	0.881051995
LILRB1	PLEK	0.85953686	SLA	SRGN	0.850244541
LILRB1	EVI2B	0.882631994	SLA	C1QB	0.866819163
LILRB1	FERMT3	0.871379019	SLA	C1QC	0.878775331
LILRB1	C3AR1	0.867353777	PRF1	GZMB	0.864950113
LILRB1	FCGR2A	0.8595868	PRF1	CD7	0.852952772
LILRB1	CYBB	0.882696529	PRF1	CD3E	0.879117513
LILRB1	CSF1R	0.880070542	PRF1	IL2RB	0.889358178
LILRB1	CD53	0.883992392	PRF1	CD2	0.853456008
LILRB1	CD4	0.876899109	PRF1	IL2RG	0.871276274
LILRB1	SPI1	0.860599456	PRF1	NKG7	0.893390162
LILRB1	LAPTM5	0.872717553	ARHGAP9	BIN2	0.942934178
TAGAP	SLA	0.863612908	ARHGAP9	TBXAS1	0.851692137
TAGAP	ARHGAP9	0.890449899	ARHGAP9	ITGAL	0.914167377
TAGAP	CD84	0.884772556	ARHGAP9	NCKAP1L	0.892187652
TAGAP	SIGLEC9	0.879525859	ARHGAP9	FGR	0.871605256
TAGAP	BIN2	0.91894716	ARHGAP9	AOAH	0.868167548
TAGAP	ARHGEF6	0.872891216	ARHGAP9	CYTH4	0.937869206
TAGAP	ITGAL	0.891274615	ARHGAP9	MYO1F	0.889030155
TAGAP	NCKAP1L	0.908219254	ARHGAP9	ITGAX	0.86501863
TAGAP	SAMSN1	0.887372871	ARHGAP9	PTPRC	0.895527985
TAGAP	ITGAM	0.885929467	ARHGAP9	LAT2	0.880622622
TAGAP	CYTH4	0.889217063	ARHGAP9	FYB1	0.871810823
TAGAP	MYO1F	0.88796875	ARHGAP9	IL2RB	0.852770696
TAGAP	ITGAX	0.876569725	ARHGAP9	IL10RA	0.915288183
TAGAP	PTPRC	0.912108094	ARHGAP9	LILRB4	0.881161089
TAGAP	LAT2	0.895274543	ARHGAP9	LCP2	0.91259333
TAGAP	FYB1	0.899063758	ARHGAP9	LAIR1	0.906870884
TAGAP	IL10RA	0.903194686	ARHGAP9	APBB1IP	0.88449762
TAGAP	GIMAP6	0.861940711	ARHGAP9	NCF4	0.923952412
TAGAP	LILRB4	0.884709329	ARHGAP9	ADAP2	0.857005635
TAGAP	LCP2	0.919596096	ARHGAP9	SASH3	0.939815281
TAGAP	LAIR1	0.883184651	ARHGAP9	HCK	0.876936512
TAGAP	APBB1IP	0.936962411	ARHGAP9	ABI3	0.872632295
TAGAP	CD86	0.911115459	ARHGAP9	MPEG1	0.854030941
TAGAP	MNDA	0.89099253	ARHGAP9	MS4A6A	0.881749549
TAGAP	ADAP2	0.871985191	ARHGAP9	WAS	0.912590492
TAGAP	LRRC25	0.851177778	ARHGAP9	CD37	0.907089744
TAGAP	HAVCR2	0.898689421	ARHGAP9	LPXN	0.888032139
TAGAP	SASH3	0.91807915	ARHGAP9	PLEK	0.871342153
TAGAP	HCK	0.858990509	ARHGAP9	EVI2B	0.862999864
TAGAP	ABI3	0.851385481	ARHGAP9	FERMT3	0.917909219
TAGAP	FGL2	0.865530261	ARHGAP9	SELPLG	0.903718891
TAGAP	MPEG1	0.850268355	ARHGAP9	C3AR1	0.857011194
TAGAP	WAS	0.894224749	ARHGAP9	CYBB	0.887127795
TAGAP	CD37	0.883210776	ARHGAP9	ITGB2	0.86392208
TAGAP	PLEK	0.881440901	ARHGAP9	CSF1R	0.855497008
TAGAP	EVI2B	0.886857134	ARHGAP9	CD53	0.883687756
TAGAP	FERMT3	0.884854723	ARHGAP9	SPI1	0.868177462
TAGAP	SELPLG	0.868369893	ARHGAP9	LAPTM5	0.854050475
TAGAP	C3AR1	0.879193775	ARHGAP9	C1QC	0.852772626
TAGAP	GIMAP4	0.850998464	MEIS3	HEPH	0.850782807

TAGAP	CYBB	0.881221524	MEIS3	FAP	0.875018076
TAGAP	ITGB2	0.852475089	MEIS3	HIC1	0.864871365
TAGAP	RNASE6	0.851516585	MEIS3	CLMP	0.896516248
TAGAP	CSF1R	0.890835961	MEIS3	MSC	0.86603418
TAGAP	CD53	0.877279665	MEIS3	PRRX1	0.888466968
TAGAP	CD4	0.871881935	MEIS3	GLT8D2	0.879336572
TAGAP	SPI1	0.856645943	MEIS3	CDH11	0.903257131
TAGAP	LAPTM5	0.864799994	MEIS3	VCAN	0.863233253
GPR84	SIGLEC9	0.88290128	MEIS3	COL5A2	0.893392755
GPR84	FGR	0.867083646	MEIS3	COL6A3	0.891389056
GPR84	CLEC5A	0.851111105	MEIS3	EMILIN1	0.887230978
GPR84	ITGAM	0.882866788	MEIS3	COL5A1	0.851687758
GPR84	CYTH4	0.863064544	MEIS3	MMP2	0.868505078
GPR84	MYO1F	0.871852135	MEIS3	COL3A1	0.87223247
GPR84	ITGAX	0.872224285	NFAM1	NCKAP1L	0.86777366
GPR84	LAT2	0.860212927	NFAM1	IL10RA	0.862075826
GPR84	LILRB4	0.874726821	CYTIP	PTPRC	0.867852264
GPR84	GNA15	0.861303619	CYTIP	SLAMF8	0.864627451
GPR84	CD300A	0.862493705	CYTIP	IL2RG	0.85641863
GPR84	LRRC25	0.863081053	CD68	FCER1G	0.882596755
GPR84	HCK	0.871795047	CD68	TYROBP	0.881502287
GPR84	PLEK	0.85767882	PLXDC1	PRRX1	0.855013316
GPR84	FERMT3	0.870623083	PLXDC1	PDGFRB	0.907071043
GPR84	CYBB	0.857611785	GNG2	CNRIP1	0.861246304
GPR84	ITGB2	0.8571656	CD84	TLR4	0.896322854
GPR84	CD4	0.863114853	CD84	BIN2	0.895135269
GPR84	SPI1	0.865641196	CD84	SLC37A2	0.868942581
ZNF234	ZNF112	0.860952015	CD84	ARHGEF6	0.881838457
ZNF234	ZNF227	0.893032101	CD84	ITGAL	0.878766071
ZNF234	ZNF45	0.895287747	CD84	NCKAP1L	0.954890437
FAM78A	BIN2	0.856098601	CD84	AOAH	0.91896637
FAM78A	ARHGEF6	0.867566915	CD84	SAMSN1	0.879699646
FAM78A	CYTH4	0.870233668	CD84	ITGAM	0.883028373
FAM78A	IL10RA	0.877955073	CD84	CYTH4	0.895347938
FAM78A	GIMAP6	0.881916507	CD84	ITGAX	0.880713154
FAM78A	LAIR1	0.854563179	CD84	PTPRC	0.936929904
FAM78A	ADAP2	0.851108508	CD84	FYB1	0.904220952
FAM78A	SLCO2B1	0.856405816	CD84	IL10RA	0.926096006
FAM78A	SASH3	0.858789049	CD84	GIMAP6	0.875035866
FAM78A	FERMT3	0.863120808	CD84	LILRB4	0.86146331
FAM78A	CSF1R	0.861618467	CD84	LCP2	0.906835428
SIT1	CD3D	0.869319519	CD84	LAIR1	0.898939132
SIT1	CD2	0.85307507	CD84	NCF2	0.85273637
LGALS7B	LGALS7	1	CD84	PIK3AP1	0.864546019
ZNF347	ZNF525	0.856167903	CD84	APBB1IP	0.886068102
ZNF347	ZNF761	0.868520296	CD84	CD86	0.86049654
CXCR3	CD3D	0.87131719	CD84	ARHGAP30	0.851114427
CXCR3	CD3E	0.881569984	CD84	ADAP2	0.905393628
CXCR3	CD2	0.88225202	CD84	SLCO2B1	0.927593312
SLA	ARHGAP9	0.885297467	CD84	MS4A7	0.862349434
SLA	NFAM1	0.911377207	CD84	HAVCR2	0.880693234
SLA	CD84	0.920054962	CD84	SASH3	0.857788469
SLA	SIGLEC9	0.885004151	CD84	HCK	0.886111707
SLA	LILRB2	0.861212943	CD84	CCR1	0.868800925
SLA	BIN2	0.905824509	CD84	FGL2	0.851074368
SLA	ITGAL	0.855805418	CD84	MPEG1	0.948324865

SLA	NCKAP1L	0.921363517	CD84	PLEK	0.92036692
SLA	SAMSN1	0.92504704	CD84	CD163	0.860852886
SLA	ITGAM	0.881170563	CD84	EVI2B	0.921370483
SLA	CYTH4	0.875408364	CD84	FPR3	0.864155998
SLA	MYO1F	0.860825957	CD84	SELPLG	0.854418854
SLA	ITGAX	0.880774057	CD84	C3AR1	0.901887029
SLA	PTPRC	0.915984314	CD84	FCGR2A	0.889840449
SLA	LAT2	0.856698899	CD84	CYBB	0.933553422
SLA	FYB1	0.902578549	CD84	ITGB2	0.866110299
SLA	IL10RA	0.902101766	CD84	CSF1R	0.870577328
SLA	LILRB4	0.885177652	CD84	CD53	0.864312096
SLA	LCP2	0.905490127	CD84	FCGR3A	0.87011445
SLA	CD300A	0.861201158	CD84	LAPTM5	0.879394364
SLA	LAIR1	0.924882203	ZNF829	ZNF568	0.85878841
SLA	APBB1IP	0.91325504	ZNF829	ZNF585A	0.850096928
SLA	CD86	0.919275326	ZNF568	ZNF585A	0.867122242
SLA	NCF4	0.875946118	MZB1	CD79A	0.911080243
SLA	MNDA	0.859734618	MZB1	CD27	0.867606512
SLA	ARHGAP30	0.851597892	MZB1	IGLL5	0.912610387
SLA	ADAP2	0.873129876	MZB1	JCHAIN	0.898163973
SLA	LRRC25	0.887568196	ZNF569	ZNF570	0.885862306
SLA	MS4A7	0.85944855	ZNF112	ZNF180	0.863138494
SLA	HAVCR2	0.923092794	SIGLEC9	LILRB2	0.882731597
SLA	SASH3	0.907077864	SIGLEC9	BIN2	0.851839516
SLA	HCK	0.894383552	SIGLEC9	SLC37A2	0.866971829
SLA	CCR1	0.859609766	SIGLEC9	TBXAS1	0.866988782
SLA	ABI3	0.863673179	SIGLEC9	NCKAP1L	0.901578131
SLA	MPEG1	0.85671206	SIGLEC9	FGR	0.860071932
SLA	MS4A6A	0.863025503	SIGLEC9	SAMSN1	0.878071896
SLA	WAS	0.902989431	SIGLEC9	ITGAM	0.856854223
SLA	CD37	0.888682238	SIGLEC9	CYTH4	0.905798196
SLA	PLEK	0.882715836	SIGLEC9	FCGR1A	0.89015226
SLA	CD163	0.853394092	SIGLEC9	TMIGD3	0.863599498
SLA	EVI2B	0.908424185	SIGLEC9	LAT2	0.862434448
SLA	FERMT3	0.893840754	SIGLEC9	IL10RA	0.882257452
SLA	SELPLG	0.853778461	SIGLEC9	GIMAP6	0.851254249
SLA	C3AR1	0.887829825	SIGLEC9	LILRB4	0.898299599
SLA	FCGR2A	0.876248296	SIGLEC9	LCP2	0.883140329
SLA	GIMAP4	0.855555986	SIGLEC9	CD300A	0.919359641
SLA	C1orf162	0.85845888	SIGLEC9	LAIR1	0.935815829
SLA	LSP1	0.869226643	SIGLEC9	TLR2	0.869319268
SLA	CYBB	0.901626444	SIGLEC9	NCF2	0.88104613
SLA	ITGB2	0.87754774	SIGLEC9	APBB1IP	0.865615047
SLA	RNASE6	0.862519785	SIGLEC9	CD86	0.897546705
SLA	CSF1R	0.8857652	SIGLEC9	NCF4	0.892738946
SLA	CD53	0.907220527	SIGLEC9	ADAP2	0.904733817
SLA	CD4	0.88718601	SIGLEC9	SLCO2B1	0.878495663
SLA	FCGR3A	0.850384159	SIGLEC9	LRRC25	0.93051416
SLA	SPI1	0.879630386	SIGLEC9	HAVCR2	0.918733868
SLA	LAPTM5	0.897793431	SIGLEC9	MS4A4A	0.858610446
CXCL3	CXCL2	0.859480203	SIGLEC9	SASH3	0.894719646
PRF1	GZMB	0.866034912	SIGLEC9	HCK	0.905337773
PRF1	CD3E	0.896925215	SIGLEC9	CCR1	0.873517902
PRF1	IL2RB	0.933153066	SIGLEC9	ABI3	0.860965148
PRF1	CTSW	0.877304919	SIGLEC9	MS4A6A	0.871983775
PRF1	CD2	0.862784735	SIGLEC9	WAS	0.870864788

PRF1	GZMA	0.910026497	SIGLEC9	PLEK	0.882764103
PRF1	NKG7	0.904117971	SIGLEC9	CD163	0.876794629
PRF1	CCL5	0.876722191	SIGLEC9	FERMT3	0.920230373
ARHGAP9	NFAM1	0.864143957	SIGLEC9	SELPLG	0.868898304
ARHGAP9	SIGLEC9	0.876794188	SIGLEC9	C3AR1	0.927834766
ARHGAP9	BIN2	0.962852977	SIGLEC9	FCGR2A	0.917180409
ARHGAP9	ARHGEF6	0.85337352	SIGLEC9	CYBB	0.918057521
ARHGAP9	ITGAL	0.893456607	SIGLEC9	ITGB2	0.897418444
ARHGAP9	NCKAP1L	0.882555771	SIGLEC9	CSF1R	0.905639246
ARHGAP9	SAMSN1	0.872502175	SIGLEC9	CD53	0.913028768
ARHGAP9	ITGAM	0.865528332	SIGLEC9	ALOX5AP	0.863067048
ARHGAP9	CYTH4	0.911582821	SIGLEC9	VSIG4	0.897499078
ARHGAP9	MYO1F	0.923565899	SIGLEC9	FCGR3A	0.875923583
ARHGAP9	DOK3	0.893575328	SIGLEC9	SPI1	0.859167068
ARHGAP9	ITGAX	0.890597085	SIGLEC9	CD14	0.909062273
ARHGAP9	PTPRC	0.889128993	SIGLEC9	LAPTM5	0.919261306
ARHGAP9	LAT2	0.938622181	SIGLEC9	C1QB	0.870851876
ARHGAP9	FYB1	0.896249935	SIGLEC9	C1QC	0.895806282
ARHGAP9	IL10RA	0.900462266	ROBO4	PCDH12	0.863052217
ARHGAP9	LILRB4	0.889133086	ROBO4	CDH5	0.918969235
ARHGAP9	LCP2	0.922841236	ROBO4	ESAM	0.852979635
ARHGAP9	LAIR1	0.909464033	SIGLEC10	LILRB2	0.87328034
ARHGAP9	APBB1IP	0.918905088	SIGLEC10	BIN2	0.893504529
ARHGAP9	CD86	0.89967791	SIGLEC10	ITGAL	0.873597428
ARHGAP9	TNFAIP8L2	0.903378109	SIGLEC10	NCKAP1L	0.874965134
ARHGAP9	NCF4	0.914665166	SIGLEC10	AOAH	0.867866664
ARHGAP9	ADAP2	0.865414346	SIGLEC10	PTPRC	0.877373256
ARHGAP9	LRRC25	0.854302345	SIGLEC10	FYB1	0.903279242
ARHGAP9	HAVCR2	0.884507612	SIGLEC10	IL10RA	0.875686366
ARHGAP9	SASH3	0.932317544	SIGLEC10	LILRB4	0.891839369
ARHGAP9	ABI3	0.928072321	SIGLEC10	LCP2	0.90437419
ARHGAP9	WAS	0.953408975	SIGLEC10	LAIR1	0.897853532
ARHGAP9	CD37	0.93467053	SIGLEC10	TLR2	0.863110006
ARHGAP9	LPXN	0.854420426	SIGLEC10	CD86	0.874007236
ARHGAP9	EVI2B	0.859421735	SIGLEC10	SLAMF8	0.881457077
ARHGAP9	FERMT3	0.932044142	SIGLEC10	HAVCR2	0.857011019
ARHGAP9	SELPLG	0.889141128	SIGLEC10	SASH3	0.88966293
ARHGAP9	C3AR1	0.855107785	SIGLEC10	HCK	0.860127064
ARHGAP9	GIMAP4	0.860758647	SIGLEC10	ABI3	0.863778414
ARHGAP9	C1orf162	0.857098225	SIGLEC10	CD37	0.852727529
ARHGAP9	LSP1	0.874781711	SIGLEC10	EVI2B	0.872715516
ARHGAP9	CYBB	0.863720755	SIGLEC10	FERMT3	0.863137708
ARHGAP9	CSF1R	0.883361232	SIGLEC10	SELPLG	0.856315754
ARHGAP9	CD53	0.882448259	SIGLEC10	GIMAP4	0.866102986
ARHGAP9	CD4	0.882679818	SIGLEC10	CYBB	0.870963654
ARHGAP9	AIF1	0.86241036	SIGLEC10	CD53	0.867825161
ARHGAP9	SPI1	0.917218781	ZEB1	RECK	0.860812503
XCL2	GZMA	0.860452999	ZEB1	ANTXR2	0.863070737
MEIS3	ITGA11	0.884546806	ZEB1	HIC1	0.868960587
MEIS3	FBN1	0.863681591	ZEB1	CNRIP1	0.871516842
MEIS3	PODN	0.873093302	ZEB1	CLMP	0.862794723
MEIS3	COL8A1	0.855155443	ZEB1	DDR2	0.861440749
MEIS3	COL10A1	0.867222126	ZEB1	PDGFRA	0.859326426
MEIS3	THBS2	0.890996482	ZEB1	FBN1	0.850908003
MEIS3	DCN	0.860871628	ZEB1	GASK1B	0.862292612
MEIS3	COL1A1	0.869307148	ZEB1	LHFPL6	0.867004029

NFAM1	CD84	0.932978232	ZEB1	PDGFRB	0.866024122
NFAM1	SIGLEC9	0.891351745	ZEB1	EMILIN1	0.865702443
NFAM1	LILRB2	0.897532032	KIF14	ASPM	0.882122425
NFAM1	FCGR2B	0.886923957	CCDC96	ROPN1L	0.885576019
NFAM1	BIN2	0.906354112	CCDC96	CFAP45	0.885189347
NFAM1	SLC37A2	0.861306087	CCDC96	WDR38	0.909199216
NFAM1	NCKAP1L	0.914667152	CCDC96	C5orf49	0.865831928
NFAM1	FGR	0.887559932	PCDH12	CDH5	0.885891658
NFAM1	SAMSN1	0.876615101	PCDH12	CD93	0.861959389
NFAM1	ITGAM	0.928134571	HEPH	FAP	0.871936624
NFAM1	CYTH4	0.910147885	HEPH	ANTXR2	0.893414936
NFAM1	MYO1F	0.882094027	HEPH	IRAG1	0.908992857
NFAM1	ITGAX	0.895296799	HEPH	CLMP	0.883517535
NFAM1	PTPRC	0.89482036	HEPH	PRRX1	0.874673864
NFAM1	FYB1	0.858650793	HEPH	ITGA11	0.862326192
NFAM1	IL10RA	0.919112862	HEPH	FBN1	0.892616995
NFAM1	LILRB4	0.900022603	HEPH	OLFML1	0.862616547
NFAM1	GNA15	0.860175958	HEPH	GASK1B	0.850687555
NFAM1	LCP2	0.851988159	HEPH	CDH11	0.922162774
NFAM1	LAIR1	0.930801592	HEPH	COL8A1	0.875321046
NFAM1	APBB1IP	0.8845411	HEPH	FILIP1L	0.928247898
NFAM1	CD86	0.891444998	HEPH	VCAN	0.854901653
NFAM1	SLAMF8	0.890312734	HEPH	COL5A2	0.87653643
NFAM1	TNFAIP8L2	0.858219856	HEPH	COL6A3	0.880439251
NFAM1	NCF4	0.879876686	HEPH	ANTXR1	0.879047608
NFAM1	ADAP2	0.866777302	HEPH	CTSK	0.855843761
NFAM1	SLCO2B1	0.874023721	HEPH	MMP2	0.878749729
NFAM1	LRRC25	0.909214206	HEPH	AEBP1	0.868860595
NFAM1	HAVCR2	0.885992877	HEPH	TIMP2	0.867534283
NFAM1	SASH3	0.899160618	HEPH	COL1A2	0.855137449
NFAM1	HCK	0.924492349	ZNF780A	ZNF780B	0.870938004
NFAM1	MPEG1	0.895038287	LILRB2	BIN2	0.859456646
NFAM1	MS4A6A	0.870042852	LILRB2	NCKAP1L	0.876968979
NFAM1	WAS	0.904228818	LILRB2	FGR	0.864725516
NFAM1	CD37	0.874519674	LILRB2	AOAH	0.875406275
NFAM1	PLEK	0.892598909	LILRB2	SAMSN1	0.881790916
NFAM1	CD163	0.869159486	LILRB2	CYTH4	0.850714241
NFAM1	EVI2B	0.889544409	LILRB2	FCGR1A	0.86331131
NFAM1	FERMT3	0.909553999	LILRB2	PTPRC	0.893145396
NFAM1	SELPLG	0.863375179	LILRB2	FYB1	0.880083657
NFAM1	C3AR1	0.889170934	LILRB2	IL10RA	0.90471995
NFAM1	FCGR2A	0.875007676	LILRB2	GIMAP6	0.854737403
NFAM1	LSP1	0.871235748	LILRB2	LILRB4	0.908304918
NFAM1	CYBB	0.915949532	LILRB2	LCP2	0.902728109
NFAM1	ITGB2	0.910215386	LILRB2	CD300A	0.859112161
NFAM1	CSF1R	0.907561129	LILRB2	LAIR1	0.92519143
NFAM1	CD53	0.891330311	LILRB2	TLR2	0.856328269
NFAM1	CD4	0.896506316	LILRB2	CD86	0.914301329
NFAM1	AIF1	0.863691116	LILRB2	SLAMF8	0.872149454
NFAM1	SPI1	0.885756191	LILRB2	NCF4	0.889323879
NFAM1	LYZ	0.854952429	LILRB2	LRRC25	0.872122334
NFAM1	CD14	0.856989771	LILRB2	HAVCR2	0.877456701
NFAM1	LAPTM5	0.884161469	LILRB2	MS4A4A	0.88799938
CYTIP	PTPRC	0.88063318	LILRB2	SASH3	0.904203179
CYTIP	EVI2B	0.875431893	LILRB2	HCK	0.896838866
CD68	FCER1G	0.856695019	LILRB2	CCR1	0.869418342

CD68	TYROBP	0.866939881	LILRB2	ABI3	0.864907007
PLXDC1	ADAMTS12	0.864054736	LILRB2	MS4A6A	0.870525537
PLXDC1	OLFML2B	0.86902928	LILRB2	WAS	0.861491328
PLXDC1	PDGFRB	0.88445499	LILRB2	PLEK	0.895892934
CD84	SIGLEC9	0.904324262	LILRB2	CD163	0.88992167
CD84	LILRB2	0.90495949	LILRB2	EVI2B	0.855252701
CD84	CLEC4A	0.868320885	LILRB2	FPR3	0.878294437
CD84	TLR4	0.912096356	LILRB2	FERMT3	0.901295945
CD84	FCGR2B	0.859394952	LILRB2	C3AR1	0.86801077
CD84	BIN2	0.922061635	LILRB2	FCGR2A	0.882087389
CD84	SLC37A2	0.90873536	LILRB2	GIMAP4	0.861452457
CD84	TBXAS1	0.850442875	LILRB2	CYBB	0.884852732
CD84	CSF2RA	0.870834861	LILRB2	CD53	0.910978159
CD84	ARHGEF6	0.869978699	LILRB2	VSIG4	0.865317774
CD84	NCKAP1L	0.960113965	LILRB2	FCGR3A	0.86361758
CD84	FGR	0.861509893	LILRB2	LAPTM5	0.865896149
CD84	SAMSN1	0.889778542	LILRB2	SRGN	0.873651616
CD84	ITGAM	0.938360365	LILRB2	C1QA	0.879419588
CD84	CYTH4	0.905423913	LILRB2	C1QB	0.8833983
CD84	MYO1F	0.888292452	LILRB2	C1QC	0.886604892
CD84	IGSF6	0.854783275	ERCC4	MRTFB	0.909579589
CD84	ITGAX	0.909555771	FAP	ADAMTS12	0.924951821
CD84	PTPRC	0.939868606	FAP	CLMP	0.871403027
CD84	FYB1	0.901060187	FAP	ADAM12	0.934481774
CD84	IL10RA	0.915214004	FAP	COLEC12	0.873888898
CD84	GIMAP6	0.870712877	FAP	INHBA	0.936433729
CD84	LILRB4	0.909370333	FAP	PRRX1	0.885655565
CD84	GNA15	0.851508088	FAP	ITGA11	0.903051813
CD84	LCP2	0.891263206	FAP	PDLIM3	0.863892573
CD84	CD300A	0.863686247	FAP	FBN1	0.882534753
CD84	LAIR1	0.936545524	FAP	ADAMTS2	0.891996327
CD84	TLR2	0.850498261	FAP	VCAM1	0.872854773
CD84	NCF2	0.880257988	FAP	ISM1	0.905337958
CD84	APBB1IP	0.920547365	FAP	COL11A1	0.900653702
CD84	CD86	0.909429101	FAP	CRISPLD2	0.898693269
CD84	SLAMF8	0.906576954	FAP	C1QTNF6	0.8643376
CD84	TNFAIP8L2	0.850870167	FAP	COPZ2	0.863491765
CD84	NCF4	0.851212083	FAP	CDH11	0.864934604
CD84	MNDA	0.883592492	FAP	COL8A1	0.892872997
CD84	ADAP2	0.890181638	FAP	VCAN	0.943208632
CD84	SLCO2B1	0.903045519	FAP	LOX	0.866410443
CD84	LRRC25	0.909049856	FAP	TMEM158	0.851770411
CD84	MS4A7	0.857148257	FAP	POSTN	0.921266284
CD84	HAVCR2	0.915765108	FAP	ECM1	0.865749668
CD84	GPR34	0.872899643	FAP	THBS2	0.928473169
CD84	SASH3	0.904832231	FAP	COL5A2	0.95481696
CD84	HCK	0.945720038	FAP	SFRP2	0.877096706
CD84	FGL2	0.851339987	FAP	COL6A3	0.893623188
CD84	MPEG1	0.941842844	FAP	CTSK	0.903007156
CD84	MS4A6A	0.884873424	FAP	COL5A1	0.898234536
CD84	WAS	0.885272565	FAP	CTHRC1	0.866389151
CD84	LHFPL2	0.850886022	FAP	MMP2	0.91808073
CD84	CD37	0.866986852	FAP	LUM	0.894154645
CD84	PLEK	0.925457326	FAP	FN1	0.904371045
CD84	SH2B3	0.852218302	FAP	COL3A1	0.918252932
CD84	CD163	0.881394151	FAP	COL1A2	0.855889349

CD84	EVI2B	0.926887433	FAP	SPARC	0.909477623
CD84	FPR3	0.911192482	FAP	COL1A1	0.863573599
CD84	FERMT3	0.905703201	STX11	ITGAL	0.879121125
CD84	SELPLG	0.863907338	STX11	SAMSN1	0.88947002
CD84	C3AR1	0.922472345	STX11	PTPRC	0.89280806
CD84	FCGR2A	0.894175577	STX11	FYB1	0.873477483
CD84	LSP1	0.8748107	STX11	IL10RA	0.851353807
CD84	CYBB	0.946596589	STX11	LCP2	0.88569295
CD84	ITGB2	0.92257212	STX11	CD86	0.867167898
CD84	CSF1R	0.923862187	STX11	HCK	0.865706689
CD84	CD53	0.910922632	STX11	EVI2B	0.855412785
CD84	CD4	0.929063537	ZNF324B	ZNF324	0.904204157
CD84	FCGR3A	0.856589697	H3C11	H1-5	0.90156449
CD84	SPI1	0.861564237	H3C11	H3C7	0.874285356
CD84	LYZ	0.85201544	H3C11	H1-4	0.857040021
CD84	LAPTM5	0.905610222	CLEC4A	AOAH	0.877297249
MZB1	CD79A	0.850603561	CLEC4A	PTPRC	0.863270941
MZB1	IGLL5	0.873782569	CLEC4A	MS4A6A	0.875156617
MZB1	JCHAIN	0.879553196	CLEC4A	EVI2B	0.853867273
ZNF569	ZNF570	0.891386503	CLEC4A	CD53	0.869405994
ZNF569	ZNF420	0.851027323	SP110	SAMD9	0.859427497
ZNF112	ZNF227	0.871426831	SP110	SP100	0.856960509
ECM2	HEPH	0.862565073	HOXA4	HOXA3	0.900087547
ECM2	DACT1	0.862604709	HOXA4	HOXA5	0.894695219
ECM2	ANTXR2	0.888991602	ZNF563	ZNF136	0.862252374
ECM2	IRAG1	0.851936461	ZNF563	ZNF791	0.850466445
ECM2	CCN4	0.860644542	CFAP73	LRRC46	0.860034631
ECM2	ITGA11	0.870053288	CFAP73	CFAP45	0.861810064
ECM2	FBN1	0.889342939	TLR4	ARHGEF6	0.870466911
ECM2	FNDC1	0.888688151	TLR4	NCKAP1L	0.853198177
ECM2	THBS2	0.854937178	TLR4	AOAH	0.850890332
ECM2	COL1A2	0.856580111	TLR4	PTPRC	0.856015036
ECM2	COL1A1	0.858938039	TLR4	GIMAP6	0.850149335
SIGLEC9	LILRB2	0.867317474	TLR4	SLCO2B1	0.860960768
SIGLEC9	TLR4	0.852710817	TLR4	MS4A7	0.853460894
SIGLEC9	FCGR2B	0.871565776	TLR4	MPEG1	0.884215493
SIGLEC9	BIN2	0.898023032	TLR4	PLEK	0.864258539
SIGLEC9	SLC37A2	0.916348995	FCGR2B	TBXAS1	0.852826155
SIGLEC9	TBXAS1	0.913538286	FCGR2B	FCGR1A	0.863841374
SIGLEC9	CSF2RA	0.850177444	FCGR2B	CD300A	0.877272113
SIGLEC9	NCKAP1L	0.923259078	FCGR2B	LAIR1	0.85550539
SIGLEC9	FGR	0.883651671	FCGR2B	CD86	0.874631639
SIGLEC9	CLEC5A	0.871075337	FCGR2B	TNFAIP8L2	0.864256938
SIGLEC9	SAMSN1	0.888693926	FCGR2B	HAVCR2	0.868346028
SIGLEC9	ITGAM	0.950023901	FCGR2B	SELPLG	0.855065106
SIGLEC9	CYTH4	0.938363195	FCGR2B	RNASE6	0.850466754
SIGLEC9	MYO1F	0.900328527	FCGR2B	CD53	0.877936808
SIGLEC9	FCGR1A	0.857720398	FCGR2B	ALOX5AP	0.862397132
SIGLEC9	ITGAX	0.889784147	FCGR2B	AIF1	0.872758198
SIGLEC9	TMIGD3	0.900274466	FCGR2B	SPI1	0.862544627
SIGLEC9	PTPRC	0.866503324	MRC1	F13A1	0.875548136
SIGLEC9	LAT2	0.927383722	PPP1R32	LRRC46	0.86219975
SIGLEC9	IL10RA	0.908464433	BIN2	SLC37A2	0.852793696
SIGLEC9	FPR1	0.865820947	BIN2	APOBR	0.878838532
SIGLEC9	GIMAP6	0.850528416	BIN2	TBXAS1	0.854449066
SIGLEC9	LILRB4	0.906792687	BIN2	ITGAL	0.940154872

SIGLEC9	OSCAR	0.873685892	BIN2	NCKAP1L	0.93490151
SIGLEC9	GNA15	0.900866654	BIN2	FGR	0.89520051
SIGLEC9	LCP2	0.885337031	BIN2	AOAH	0.903044804
SIGLEC9	CD300A	0.943657383	BIN2	SAMSN1	0.882268425
SIGLEC9	LAIR1	0.935373138	BIN2	CYTH4	0.928316122
SIGLEC9	APBB1IP	0.928074266	BIN2	MYO1F	0.877887478
SIGLEC9	CD86	0.910488358	BIN2	ITGAX	0.865910406
SIGLEC9	TNFAIP8L2	0.892007187	BIN2	PTPRC	0.927379788
SIGLEC9	NCF4	0.908518053	BIN2	LAT2	0.916872053
SIGLEC9	MNDA	0.874638747	BIN2	FYB1	0.941762902
SIGLEC9	ADAP2	0.933029413	BIN2	IL10RA	0.925054748
SIGLEC9	SLCO2B1	0.892213707	BIN2	GIMAP6	0.862027329
SIGLEC9	LRRC25	0.941833305	BIN2	LILRB4	0.894971702
SIGLEC9	MS4A7	0.864636129	BIN2	LCP2	0.948704608
SIGLEC9	HAVCR2	0.924947344	BIN2	LAIR1	0.933043345
SIGLEC9	GPR34	0.907601034	BIN2	APBB1IP	0.949424334
SIGLEC9	SASH3	0.929153872	BIN2	CD86	0.896126585
SIGLEC9	HCK	0.920776132	BIN2	TNFAIP8L2	0.851892416
SIGLEC9	ABI3	0.872478444	BIN2	NCF4	0.903777612
SIGLEC9	MPEG1	0.858854278	BIN2	MNDA	0.865397511
SIGLEC9	MS4A6A	0.884343048	BIN2	ARHGAP30	0.869265686
SIGLEC9	WAS	0.895482148	BIN2	ADAP2	0.909459918
SIGLEC9	CD37	0.856692163	BIN2	SLCO2B1	0.852264893
SIGLEC9	PLEK	0.909643703	BIN2	LRRC25	0.869836561
SIGLEC9	CD163	0.895662188	BIN2	HAVCR2	0.900807598
SIGLEC9	EVI2B	0.861159633	BIN2	SASH3	0.949830338
SIGLEC9	STAB1	0.874418962	BIN2	HCK	0.905042267
SIGLEC9	FPR3	0.854479524	BIN2	ABI3	0.893997261
SIGLEC9	FERMT3	0.922272061	BIN2	FGL2	0.852425282
SIGLEC9	SELPLG	0.879374084	BIN2	MPEG1	0.876429742
SIGLEC9	C3AR1	0.927860312	BIN2	MS4A6A	0.892973887
SIGLEC9	FCGR2A	0.897830279	BIN2	WAS	0.918016187
SIGLEC9	LSP1	0.865114413	BIN2	CD37	0.929460362
SIGLEC9	CYBB	0.92378158	BIN2	LPXN	0.89608628
SIGLEC9	ITGB2	0.900445386	BIN2	PLEK	0.896123829
SIGLEC9	RNASE6	0.878419451	BIN2	EVI2B	0.93525222
SIGLEC9	CSF1R	0.936655363	BIN2	FERMT3	0.908524807
SIGLEC9	CD53	0.904676908	BIN2	SELPLG	0.933823731
SIGLEC9	CD4	0.911180291	BIN2	C3AR1	0.899056285
SIGLEC9	ALOX5AP	0.886267047	BIN2	FCGR2A	0.873031962
SIGLEC9	AIF1	0.868147721	BIN2	GIMAP4	0.878550481
SIGLEC9	VSIG4	0.887580339	BIN2	CYBB	0.926210762
SIGLEC9	SPI1	0.893063527	BIN2	ITGB2	0.875081371
SIGLEC9	CD14	0.887252773	BIN2	CSF1R	0.89005417
SIGLEC9	LAPTM5	0.893726544	BIN2	CD53	0.912369615
ZNF585A	ZNF585B	0.871004407	BIN2	CD4	0.864633061
ZNF585A	ZNF420	0.874659257	BIN2	FCGR3A	0.857543102
ROBO4	PCDH12	0.875196965	BIN2	SPI1	0.884979016
ROBO4	CDH5	0.914601997	BIN2	CD14	0.851517165
ROBO4	ESAM	0.861456531	BIN2	LAPTM5	0.888329846
SIGLEC10	AOAH	0.912996968	BIN2	C1QC	0.859932304
ZEB1	DACT1	0.863511282	SLC37A2	NCKAP1L	0.90188209
ZEB1	ANTXR2	0.900678212	SLC37A2	ITGAM	0.879985369
ZEB1	HIC1	0.856359756	SLC37A2	CYTH4	0.864816222
ZEB1	CCN4	0.879700082	SLC37A2	ITGAX	0.85851325
ZEB1	MSRB3	0.85480225	SLC37A2	IL10RA	0.882431992

ZEB1	FBN1	0.888616529	SLC37A2	LILRB4	0.850223651
ZEB1	CDH11	0.864002898	SLC37A2	APBB1IP	0.865573713
ZEB1	FILIP1L	0.862040394	SLC37A2	ADAP2	0.871721532
ZEB1	VCAN	0.860818718	SLC37A2	SLCO2B1	0.86856542
ZEB1	LHFPL6	0.853044917	SLC37A2	MPEG1	0.851465662
ZEB1	COL6A3	0.916335058	SLC37A2	CYBB	0.88009189
ZEB1	DCN	0.867754844	SLC37A2	CSF1R	0.884272029
ZEB1	COL5A1	0.872773481	ZFP14	ZNF566	0.888720377
ZEB1	COL6A2	0.853742405	ZFP14	ZNF529	0.865932291
ZEB1	COL3A1	0.880954819	APOBR	LAT2	0.86876719
ZEB1	COL1A2	0.87568782	APOBR	APBB1IP	0.867093485
ZEB1	COL1A1	0.893670342	APOBR	WAS	0.855577862
KIF14	ASPM	0.900509815	DACT1	CLMP	0.869396428
KIF14	CENPF	0.865335193	TBXAS1	NCKAP1L	0.873363258
PCDH12	CDH5	0.880622283	TBXAS1	AOAH	0.857416438
HEPH	ANTXR2	0.875069514	TBXAS1	CYTH4	0.864906377
HEPH	IRAG1	0.861618792	TBXAS1	FCGR1A	0.855228038
HEPH	FNDC1	0.872931447	TBXAS1	LAT2	0.899009902
HEPH	COL6A3	0.853800631	TBXAS1	IL10RA	0.864587069
HEPH	COL1A1	0.857356854	TBXAS1	CD300A	0.873957532
LILRB2	TLR4	0.850804597	TBXAS1	LAIR1	0.876487666
LILRB2	FCGR2B	0.855506696	TBXAS1	APBB1IP	0.874952532
LILRB2	BIN2	0.879146775	TBXAS1	CD86	0.862790488
LILRB2	NCKAP1L	0.895258511	TBXAS1	TNFAIP8L2	0.881570358
LILRB2	SAMSN1	0.864349097	TBXAS1	NCF4	0.884839407
LILRB2	ITGAM	0.892746407	TBXAS1	ADAP2	0.854484102
LILRB2	CYTH4	0.923293681	TBXAS1	LRRC25	0.850870245
LILRB2	FCGR1A	0.850963866	TBXAS1	HAVCR2	0.859010995
LILRB2	ITGAX	0.881219012	TBXAS1	SASH3	0.880607305
LILRB2	PTPRC	0.890861974	TBXAS1	HCK	0.854343806
LILRB2	IL10RA	0.908478893	TBXAS1	ABI3	0.85640208
LILRB2	LILRB4	0.902642322	TBXAS1	MS4A6A	0.904313349
LILRB2	LCP2	0.863510184	TBXAS1	WAS	0.87685067
LILRB2	CD300A	0.853297683	TBXAS1	CD37	0.855631999
LILRB2	LAIR1	0.931316861	TBXAS1	FERMT3	0.870413171
LILRB2	CD86	0.904486202	TBXAS1	SELPLG	0.875555002
LILRB2	SLAMF8	0.867450071	TBXAS1	C3AR1	0.878405446
LILRB2	TNFAIP8L2	0.851175799	TBXAS1	FCGR2A	0.853407943
LILRB2	NCF4	0.893461449	TBXAS1	CYBB	0.874769069
LILRB2	ADAP2	0.853253149	TBXAS1	RNASE6	0.861865799
LILRB2	SLCO2B1	0.874743164	TBXAS1	CSF1R	0.884007532
LILRB2	LRRC25	0.900144524	TBXAS1	CD53	0.87994628
LILRB2	MS4A7	0.869834051	TBXAS1	AIF1	0.866907081
LILRB2	HAVCR2	0.870364848	TBXAS1	SPI1	0.863802534
LILRB2	SASH3	0.882933993	TBXAS1	CD14	0.860133824
LILRB2	HCK	0.907686036	TBXAS1	C1QC	0.851702005
LILRB2	MPEG1	0.911951231	ZNF493	ZNF429	0.861787014
LILRB2	MS4A6A	0.879498709	ZNF493	ZNF43	0.857145064
LILRB2	WAS	0.876094139	DDX60L	DDX60	0.861349257
LILRB2	PLEK	0.898171445	CD8A	CD2	0.854019189
LILRB2	CD163	0.901252885	ARHGEF6	NCKAP1L	0.895900943
LILRB2	EVI2B	0.850436959	ARHGEF6	ARHGAP31	0.874802744
LILRB2	FPR3	0.879464983	ARHGEF6	IL10RA	0.8625336
LILRB2	FERMT3	0.90363283	ARHGEF6	GIMAP6	0.862029638
LILRB2	SELPLG	0.85175265	ARHGEF6	SLCO2B1	0.883188743
LILRB2	C3AR1	0.90292569	ARHGEF6	MPEG1	0.855894407

LILRB2	FCGR2A	0.891185564	ARHGEF6	DAB2	0.880408158
LILRB2	CYBB	0.898039246	ITGAL	NCKAP1L	0.900749442
LILRB2	ITGB2	0.874113448	ITGAL	FGR	0.856446068
LILRB2	RNASE6	0.852160647	ITGAL	AOAH	0.87359639
LILRB2	CSF1R	0.90745604	ITGAL	SAMSN1	0.85224137
LILRB2	CD53	0.892780814	ITGAL	CYTH4	0.901817705
LILRB2	CD4	0.890187812	ITGAL	PTPRC	0.926426882
LILRB2	AIF1	0.855790963	ITGAL	FYB1	0.914233502
LILRB2	SPI1	0.870218029	ITGAL	IL2RB	0.882858859
LILRB2	CD14	0.895498085	ITGAL	IL10RA	0.91197151
LILRB2	LAPTM5	0.875326612	ITGAL	GIMAP6	0.872208681
LILRB2	C1QB	0.852061429	ITGAL	LILRB4	0.865424953
LILRB2	C1QC	0.852795556	ITGAL	LCP2	0.938651177
FAP	ADAMTS12	0.899897693	ITGAL	LAIR1	0.876227502
FAP	LRRC15	0.942689845	ITGAL	APBB1IP	0.888672755
FAP	ADAM12	0.903470069	ITGAL	CD86	0.856143205
FAP	INHBA	0.909999039	ITGAL	SIGLEC1	0.856002621
FAP	ITGA11	0.914874885	ITGAL	ARHGAP30	0.873204212
FAP	PDLIM3	0.860236973	ITGAL	ADAP2	0.870570261
FAP	FBN1	0.899566445	ITGAL	SASH3	0.906972326
FAP	ADAMTS2	0.864575291	ITGAL	HCK	0.864657402
FAP	COL11A1	0.935480058	ITGAL	MPEG1	0.868562269
FAP	CRISPLD2	0.878028918	ITGAL	CD37	0.871337242
FAP	COL8A1	0.877670472	ITGAL	LPXN	0.850944602
FAP	COL10A1	0.896954383	ITGAL	PLEK	0.865469836
FAP	VCAN	0.870809721	ITGAL	EVI2B	0.880908103
FAP	LOX	0.861654565	ITGAL	FERMT3	0.859385989
FAP	POSTN	0.872511313	ITGAL	SELPLG	0.883208461
FAP	ECM1	0.891542902	ITGAL	GIMAP4	0.851988196
FAP	THBS2	0.941347362	ITGAL	CYBB	0.881302243
FAP	COL5A2	0.878113085	ITGAL	CD53	0.851052161
FAP	MMP11	0.882003811	ZFP30	ZNF607	0.869659235
FAP	CTSK	0.870663931	ZNF813	ZNF525	0.868075387
FAP	DCN	0.85804539	ZNF813	ZNF761	0.858485639
FAP	MMP2	0.852566806	ZNF10	ZNF84	0.907908812
FAP	LUM	0.887691791	MORN5	LRRC10B	0.851266939
FAP	FN1	0.889309028	MORN5	CAPSL	0.872757511
FAP	COL3A1	0.885450786	MORN5	WDR38	0.870493163
FAP	SPARC	0.851164033	MORN5	C5orf49	0.897904443
FAP	COL1A1	0.850665725	ANTXR2	HIC1	0.880216327
STX11	SAMSN1	0.862950867	ANTXR2	FRMD6	0.862526212
STX11	PTPRC	0.868757923	ANTXR2	IRAG1	0.910216307
STX11	LCP2	0.851570642	ANTXR2	CNRIP1	0.852428086
ZNF324B	ZNF324	0.86358926	ANTXR2	CLMP	0.876869609
H3C11	H1-5	0.925136749	ANTXR2	CCN4	0.852915255
H3C11	H1-4	0.885043395	ANTXR2	PDGFRA	0.880611798
CLEC4A	FGR	0.877479117	ANTXR2	LMOD1	0.856036587
CLEC4A	ITGAM	0.873607794	ANTXR2	FBN1	0.865194271
CLEC4A	IGSF6	0.907700332	ANTXR2	OLFML1	0.932078767
CLEC4A	PTPRC	0.877654483	ANTXR2	GLT8D2	0.855560001
CLEC4A	LAIR1	0.856738299	ANTXR2	GASK1B	0.878099267
CLEC4A	NCF2	0.86161492	ANTXR2	SRPX2	0.888790285
CLEC4A	SLAMF8	0.854292946	ANTXR2	ADGRA2	0.855076546
CLEC4A	MNDA	0.854208945	ANTXR2	CDH11	0.908319896
CLEC4A	HCK	0.888638448	ANTXR2	FILIP1L	0.907385944
CLEC4A	MS4A6A	0.862085554	ANTXR2	LHFPL6	0.852396391

CLEC4A	EVI2B	0.851567875	ANTXR2	COL6A3	0.858071246
CLEC4A	CYBB	0.852037952	ANTXR2	EMILIN1	0.850006259
CLEC4A	ITGB2	0.859674694	ANTXR2	DCN	0.864171888
CLEC4A	CD53	0.851772029	ANTXR2	TIMP2	0.883646142
CLEC4A	CD4	0.851759762	H1-5	H1-3	0.868482296
CLEC4A	LYZ	0.862744492	H1-5	H3C7	0.852028924
HOXA4	HOXA3	0.881186147	H1-5	H1-4	0.924585751
HOXA4	HOXA5	0.854981484	NBPF12	NBPF11	0.905362485
ZNF563	ZNF44	0.869136135	HIC1	CNRIP1	0.899258998
ZNF570	ZNF607	0.860706083	HIC1	CLMP	0.852889349
TLR4	SLC37A2	0.853761368	HIC1	PDGFRA	0.871546564
TLR4	ARHGEF6	0.870600939	HIC1	GLT8D2	0.853343892
TLR4	TLR1	0.855003654	HIC1	ADGRA2	0.886372035
TLR4	NCKAP1L	0.886298447	HIC1	CDH11	0.860040524
TLR4	ITGAM	0.882870673	HIC1	LHFPL6	0.852309862
TLR4	CYTH4	0.867780343	HIC1	EMILIN1	0.9199806
TLR4	PTPRC	0.873407379	CD79A	IGLL5	0.860575297
TLR4	TRPV2	0.851357606	ZNF345	ZNF567	0.858338877
TLR4	IL10RA	0.8800902	NCKAP1L	FGR	0.863321512
TLR4	GIMAP6	0.906308233	NCKAP1L	AOAH	0.940705634
TLR4	LAIR1	0.867494721	NCKAP1L	SAMSN1	0.887121941
TLR4	MNDA	0.863934707	NCKAP1L	ITGAM	0.90489489
TLR4	ADAP2	0.855298627	NCKAP1L	CYTH4	0.94000898
TLR4	SLCO2B1	0.907752339	NCKAP1L	MYO1F	0.879123798
TLR4	LRRC25	0.85206838	NCKAP1L	ITGAX	0.865298684
TLR4	HAVCR2	0.852771198	NCKAP1L	PTPRC	0.93747618
TLR4	GPR34	0.889839891	NCKAP1L	LAT2	0.853037441
TLR4	HCK	0.907256512	NCKAP1L	TRPV2	0.863428806
TLR4	MPEG1	0.911094612	NCKAP1L	FYB1	0.911069701
TLR4	MS4A6A	0.884936611	NCKAP1L	IL10RA	0.963015649
TLR4	PLEK	0.890790117	NCKAP1L	GIMAP6	0.918223854
TLR4	SH2B3	0.85306523	NCKAP1L	LILRB4	0.882335848
TLR4	CD163	0.867171477	NCKAP1L	LCP2	0.933342906
TLR4	EVI2B	0.863165689	NCKAP1L	CD300A	0.854217033
TLR4	FPR3	0.892186108	NCKAP1L	LAIR1	0.936943493
TLR4	C3AR1	0.902891879	NCKAP1L	TLR2	0.882329666
TLR4	FCGR2A	0.857358342	NCKAP1L	NCF2	0.865350517
TLR4	CYBB	0.892185369	NCKAP1L	APBB1IP	0.933288262
TLR4	ITGB2	0.868287061	NCKAP1L	CD86	0.885419379
TLR4	CSF1R	0.884387383	NCKAP1L	SIGLEC1	0.856283981
TLR4	CD53	0.852921395	NCKAP1L	NCF4	0.872014922
TLR4	FCGR3A	0.851553908	NCKAP1L	MNDA	0.893845316
SPAG8	ZMYND10	0.854440994	NCKAP1L	ARHGAP30	0.870061601
FCGR2B	BIN2	0.862345568	NCKAP1L	ADAP2	0.947795263
FCGR2B	TBXAS1	0.850804595	NCKAP1L	SLCO2B1	0.953351488
FCGR2B	FGR	0.85051975	NCKAP1L	LRRC25	0.868715858
FCGR2B	ITGAM	0.892693	NCKAP1L	MS4A7	0.880618455
FCGR2B	CYTH4	0.86943022	NCKAP1L	HAVCR2	0.912387563
FCGR2B	ITGAX	0.860804098	NCKAP1L	MS4A4A	0.876315822
FCGR2B	LAT2	0.862723163	NCKAP1L	GPR34	0.8545366
FCGR2B	IL10RA	0.875242706	NCKAP1L	SASH3	0.92969632
FCGR2B	LILRB4	0.862422875	NCKAP1L	HCK	0.922173007
FCGR2B	CD300A	0.855040247	NCKAP1L	CCR1	0.870549261
FCGR2B	LAIR1	0.885904344	NCKAP1L	FGL2	0.871053738
FCGR2B	CD86	0.858753187	NCKAP1L	MPEG1	0.946390453
FCGR2B	TNFAIP8L2	0.861037188	NCKAP1L	MS4A6A	0.902073429

FCGR2B	NCF4	0.854254544	NCKAP1L	WAS	0.869833427
FCGR2B	LRRC25	0.877715314	NCKAP1L	LPXN	0.866592277
FCGR2B	HAVCR2	0.857224746	NCKAP1L	PLEK	0.941603456
FCGR2B	HCK	0.871400077	NCKAP1L	GAL3ST4	0.855527686
FCGR2B	CD37	0.855230329	NCKAP1L	CD163	0.908860331
FCGR2B	C3AR1	0.850256744	NCKAP1L	EVI2B	0.920637534
FCGR2B	AIF1	0.8720666	NCKAP1L	STAB1	0.856416804
FCGR2B	SPI1	0.867571833	NCKAP1L	FERMT3	0.906135442
FCGR2B	CD14	0.856379795	NCKAP1L	SELPLG	0.903247379
BIN2	SLC37A2	0.875121384	NCKAP1L	C3AR1	0.934557559
BIN2	TBXAS1	0.863529226	NCKAP1L	FCGR2A	0.915122343
BIN2	CSF2RA	0.881475549	NCKAP1L	CYBB	0.968928414
BIN2	ARHGEF6	0.881110863	NCKAP1L	ITGB2	0.902950432
BIN2	ITGAL	0.909037582	NCKAP1L	CSF1R	0.937034019
BIN2	NCKAP1L	0.923731216	NCKAP1L	CD53	0.909389964
BIN2	FGR	0.858867048	NCKAP1L	CD4	0.874010949
BIN2	SAMSN1	0.899048772	NCKAP1L	VSIG4	0.854095588
BIN2	ITGAM	0.909544908	NCKAP1L	FCGR3A	0.894187633
BIN2	CYTH4	0.93084816	NCKAP1L	LAPTM5	0.913422274
BIN2	MYO1F	0.936317152	NCKAP1L	C1QC	0.855637601
BIN2	FCGR1A	0.853175	HOXA3	HOXA5	0.920454983
BIN2	DOK3	0.881597266	CD27	CD3E	0.911504056
BIN2	ITGAX	0.91217709	CD27	CD2	0.884340447
BIN2	PTPRC	0.940351575	CD27	IL2RG	0.856755532
BIN2	LAT2	0.922504747	ZNF525	ZNF761	0.851506508
BIN2	FYB1	0.93251664	ARHGAP31	WIPF1	0.868563856
BIN2	IL10RA	0.932099592	ARHGAP31	CD93	0.874854895
BIN2	GIMAP6	0.862454403	ARHGAP31	SH2B3	0.853015257
BIN2	LILRB4	0.899349255	ARHGAP31	DAB2	0.882280801
BIN2	OSCAR	0.850687555	CD3D	CD3E	0.931138787
BIN2	GNA15	0.857536667	CD3D	CD2	0.933175039
BIN2	LCP2	0.934069612	CD3D	GZMA	0.892315119
BIN2	CD300A	0.859330362	CD3D	IL2RG	0.866661107
BIN2	LAIR1	0.936226452	CD3D	NKG7	0.904743395
BIN2	CD48	0.859758538	CD3D	CCL5	0.868450373
BIN2	NCF2	0.856474593	ZNF543	ZNF304	0.870954421
BIN2	APBB1IP	0.948435521	ZNF226	ZNF227	0.905178644
BIN2	CD86	0.924783307	CCL3L3	CCL4L2	0.907112124
BIN2	SLAMF8	0.879819424	ADAMTS12	LRRC15	0.862359743
BIN2	TNFAIP8L2	0.908764797	ADAMTS12	ADAM12	0.940620584
BIN2	NCF4	0.899803327	ADAMTS12	COL5A3	0.911832323
BIN2	MNDA	0.88575885	ADAMTS12	INHBA	0.861993576
BIN2	ADAP2	0.891501932	ADAMTS12	ITGA11	0.885696425
BIN2	LRRC25	0.890443582	ADAMTS12	ADAMTS2	0.921679242
BIN2	MS4A7	0.867040874	ADAMTS12	ISM1	0.890574707
BIN2	HAVCR2	0.915275265	ADAMTS12	COL11A1	0.884290163
BIN2	GPR34	0.853851542	ADAMTS12	C1QTNF6	0.896660043
BIN2	SASH3	0.941298788	ADAMTS12	VCAN	0.857672305
BIN2	HCK	0.895315263	ADAMTS12	OLFML2B	0.858291216
BIN2	ABI3	0.905981428	ADAMTS12	POSTN	0.89560196
BIN2	FGL2	0.853115006	ADAMTS12	ECM1	0.862891465
BIN2	MPEG1	0.873053932	ADAMTS12	THBS2	0.879143954
BIN2	MS4A6A	0.879657329	ADAMTS12	COL5A2	0.930720386
BIN2	WAS	0.948133581	ADAMTS12	COL6A3	0.881544649
BIN2	CD37	0.935862766	ADAMTS12	COL5A1	0.894798001
BIN2	PLEK	0.888856647	ADAMTS12	MMP2	0.864175035

BIN2	EVI2B	0.922890403	ADAMTS12	COL3A1	0.878744355
BIN2	FERMT3	0.935948802	ADAMTS12	SPARC	0.877303015
BIN2	SELPLG	0.895063877	H1-3	H3C7	0.882256621
BIN2	C3AR1	0.894919035	H1-3	H1-4	0.85467009
BIN2	FCGR2A	0.876613866	FGR	CYTH4	0.85746549
BIN2	GIMAP4	0.884341407	FGR	PTPRC	0.859158968
BIN2	C1orf162	0.859013742	FGR	TRPV2	0.852753389
BIN2	LSP1	0.902523099	FGR	FYB1	0.858234879
BIN2	CYBB	0.910331757	FGR	IL10RA	0.868255739
BIN2	ITGB2	0.896958414	FGR	LILRB4	0.866659357
BIN2	RNASE6	0.864559475	FGR	LCP2	0.876095671
BIN2	CSF1R	0.911386054	FGR	LAIR1	0.908678584
BIN2	CD53	0.914124292	FGR	NCF2	0.865600645
BIN2	CD4	0.919884478	FGR	APBB1IP	0.851315361
BIN2	AIF1	0.894278235	FGR	NCF4	0.894932362
BIN2	SPI1	0.914240295	FGR	LRRC25	0.863710278
BIN2	LAPTM5	0.886436718	FGR	HAVCR2	0.865839326
SLC37A2	TBXAS1	0.850466914	FGR	SASH3	0.885091688
SLC37A2	IRF8	0.854017799	FGR	HCK	0.903727038
SLC37A2	NCKAP1L	0.914300099	FGR	ABI3	0.864960402
SLC37A2	ITGAM	0.926079984	FGR	WAS	0.87288264
SLC37A2	CYTH4	0.894822034	FGR	CD37	0.871291582
SLC37A2	MYO1F	0.890601085	FGR	PLEK	0.850436356
SLC37A2	ITGAX	0.88885096	FGR	EVI2B	0.852877284
SLC37A2	LAT2	0.856573024	FGR	FERMT3	0.898581009
SLC37A2	IL10RA	0.881121639	FGR	SELPLG	0.892134895
SLC37A2	LILRB4	0.852106372	FGR	ITGB2	0.850555709
SLC37A2	GNA15	0.877129433	FGR	CD53	0.87013518
SLC37A2	CD300A	0.870550428	FGR	SPI1	0.865373163
SLC37A2	LAIR1	0.882258591	FRMD6	CLMP	0.880577383
SLC37A2	APBB1IP	0.884689094	FRMD6	COLEC12	0.871666877
SLC37A2	ADAP2	0.896765771	FRMD6	PRRX1	0.88307415
SLC37A2	SLCO2B1	0.872457173	FRMD6	FBN1	0.852198911
SLC37A2	LRRC25	0.867257315	FRMD6	GLT8D2	0.892203643
SLC37A2	HAVCR2	0.859330053	FRMD6	COL6A3	0.853210404
SLC37A2	SASH3	0.876738414	OSM	GPR183	0.856143159
SLC37A2	HCK	0.900869122	ZSCAN22	ZNF324	0.878513237
SLC37A2	MPEG1	0.882353947	ZNF708	ZNF100	0.874689185
SLC37A2	PLEK	0.881347176	BRF1	PACS2	0.859410348
SLC37A2	STAB1	0.855652227	ZNF430	ZNF100	0.876130135
SLC37A2	FERMT3	0.915661305	LRRC46	ROPN1L	0.856293687
SLC37A2	C3AR1	0.86653065	LRRC46	C20orf85	0.854393222
SLC37A2	LSP1	0.866764127	ZKSCAN3	ZSCAN12	0.873744816
SLC37A2	CYBB	0.894925267	IRAG1	CCN4	0.894266923
SLC37A2	ITGB2	0.896746962	IRAG1	SSC5D	0.871640762
SLC37A2	CSF1R	0.920514007	IRAG1	MMP19	0.864124194
SLC37A2	CD4	0.877452545	IRAG1	LMOD1	0.857291672
APOBR	CLEC5A	0.853969818	IRAG1	MSRB3	0.854481364
APOBR	MYO1F	0.8660384	IRAG1	FBN1	0.863009252
APOBR	ITGAX	0.879096995	IRAG1	PODN	0.866131285
APOBR	APBB1IP	0.855383639	IRAG1	ADGRA2	0.875434209
APOBR	WAS	0.857418781	IRAG1	CDH11	0.890013371
APOBR	LSP1	0.882332999	IRAG1	FNDC1	0.864186729
DACT1	ANTXR2	0.882097215	IRAG1	COL8A1	0.906496294
DACT1	CCN4	0.851142115	IRAG1	FILIP1L	0.902025911
DACT1	FBN1	0.897958366	IRAG1	MMP2	0.859179605

DACT1	COL6A3	0.875758403	IRAG1	AEBP1	0.861408349
DACT1	COL5A1	0.879131794	IRAG1	TIMP2	0.878391247
DACT1	SPARC	0.856785519	ZNF469	ADAMTS14	0.850841622
DACT1	COL1A1	0.861241056	ZNF469	COL5A1	0.857273772
TBXAS1	NCKAP1L	0.873642854	AOAH	SAMSN1	0.878602357
TBXAS1	ITGAM	0.88798602	AOAH	CYTH4	0.867586973
TBXAS1	CYTH4	0.887406191	AOAH	PTPRC	0.946995904
TBXAS1	TMIGD3	0.864708983	AOAH	FYB1	0.903054553
TBXAS1	LAT2	0.896930797	AOAH	IL10RA	0.941158506
TBXAS1	IL10RA	0.889246426	AOAH	GIMAP6	0.870515254
TBXAS1	CD300A	0.865477596	AOAH	LILRB4	0.862258201
TBXAS1	LAIR1	0.899677596	AOAH	LCP2	0.902707595
TBXAS1	APBB1IP	0.87571021	AOAH	LAIR1	0.890674577
TBXAS1	CD86	0.859898232	AOAH	APBB1IP	0.867182106
TBXAS1	TNFAIP8L2	0.870447376	AOAH	CD86	0.875938097
TBXAS1	NCF4	0.885044927	AOAH	MNDA	0.866172208
TBXAS1	ADAP2	0.891891767	AOAH	ADAP2	0.874329484
TBXAS1	SLCO2B1	0.860933615	AOAH	SLCO2B1	0.89977075
TBXAS1	LRRC25	0.88397116	AOAH	MS4A7	0.883225336
TBXAS1	GPR34	0.897483802	AOAH	HAVCR2	0.866130728
TBXAS1	SASH3	0.871624093	AOAH	MS4A4A	0.859619827
TBXAS1	HCK	0.855860705	AOAH	SASH3	0.907835458
TBXAS1	ABI3	0.880740916	AOAH	HCK	0.882637498
TBXAS1	MS4A6A	0.875632905	AOAH	FGL2	0.88488446
TBXAS1	WAS	0.853056213	AOAH	MPEG1	0.929864638
TBXAS1	CD163	0.857686247	AOAH	MS4A6A	0.911558716
TBXAS1	STAB1	0.851296875	AOAH	PLEK	0.912819056
TBXAS1	FERMT3	0.86072893	AOAH	CD163	0.854473434
TBXAS1	C3AR1	0.879787138	AOAH	EVI2B	0.90458773
TBXAS1	CYBB	0.881508851	AOAH	FPR3	0.855955089
TBXAS1	RNASE6	0.875226074	AOAH	FERMT3	0.852506128
TBXAS1	CSF1R	0.893097227	AOAH	SELPLG	0.874870536
TBXAS1	CD53	0.857934704	AOAH	C3AR1	0.895286491
TBXAS1	AIF1	0.854279329	AOAH	FCGR2A	0.870751114
TBXAS1	CD14	0.859883228	AOAH	CYBB	0.921933829
DDX60L	DDX60	0.886985293	AOAH	CSF1R	0.859868323
DDX60L	OAS2	0.855919268	AOAH	CD53	0.902557376
CSF2RA	NCKAP1L	0.860863479	AOAH	FCGR3A	0.852175739
CSF2RA	ITGAM	0.875038129	AOAH	LAPTM5	0.866241141
CSF2RA	MYO1F	0.85137012	GZMB	NKG7	0.86956373
CSF2RA	PTPRC	0.853038815	POMK	ZRANB1	0.851062754
CSF2RA	LAT2	0.858613524	HEATR5A	HECTD1	0.895007277
CSF2RA	IL10RA	0.879818547	CNRIP1	PDGFRA	0.894833584
CSF2RA	LAIR1	0.86577866	CNRIP1	EMILIN1	0.869651265
CSF2RA	CD86	0.858914744	CLMP	MSC	0.864303089
CSF2RA	HAVCR2	0.858554281	CLMP	PRRX1	0.930269872
CSF2RA	EVI2B	0.85627379	CLMP	PDLIM3	0.850280294
CSF2RA	C3AR1	0.857493439	CLMP	FBN1	0.886023469
CSF2RA	CYBB	0.868604109	CLMP	VCAM1	0.879166702
CSF2RA	ITGB2	0.865519235	CLMP	GLT8D2	0.874684516
CSF2RA	CSF1R	0.853582305	CLMP	GASK1B	0.871977277
CSF2RA	CD4	0.86156535	CLMP	CDH11	0.914658068
CD8A	CD3D	0.878871715	CLMP	SNAI2	0.876633417
ARHGEF6	NCKAP1L	0.883605414	CLMP	VCAN	0.869001323
ARHGEF6	ITGAM	0.850857239	CLMP	COL5A2	0.867018923
ARHGEF6	CYTH4	0.871909793	CLMP	COL6A3	0.866436018

ARHGEF6	MYO1F	0.872515498	CLMP	EMILIN1	0.868850821
ARHGEF6	PTPRC	0.86308809	CLMP	MMP2	0.857194697
ARHGEF6	TRPV2	0.851749613	ZNF8	ZNF324	0.85525877
ARHGEF6	IL10RA	0.895172386	ZNF8	ZNF544	0.853958014
ARHGEF6	GIMAP6	0.904403944	ZNF181	ZNF302	0.884929765
ARHGEF6	LAIR1	0.857448524	LRRC15	ITGA11	0.887601138
ARHGEF6	APBB1IP	0.858253246	LRRC15	COL11A1	0.892370009
ARHGEF6	ADAP2	0.855286391	LRRC15	MMP11	0.866931539
ARHGEF6	SLCO2B1	0.869798432	MAG	CD22	0.938243459
ARHGEF6	SASH3	0.867828836	SAMSN1	CYTH4	0.869909278
ARHGEF6	FGL2	0.859664836	SAMSN1	FCGR1A	0.892731652
ARHGEF6	MPEG1	0.880028472	SAMSN1	PTPRC	0.926962857
ARHGEF6	WAS	0.851857056	SAMSN1	FYB1	0.915541863
ARHGEF6	PLEK	0.852442946	SAMSN1	IL10RA	0.896550354
ARHGEF6	SH2B3	0.875052333	SAMSN1	LILRB4	0.918445488
ARHGEF6	FERMT3	0.850369648	SAMSN1	LCP2	0.929470529
ARHGEF6	CYBB	0.861653629	SAMSN1	CD300A	0.873884111
ARHGEF6	CSF1R	0.895020033	SAMSN1	LAIR1	0.912902611
ITGAL	NCKAP1L	0.864047515	SAMSN1	TLR2	0.863364186
ITGAL	MYO1F	0.858930954	SAMSN1	NCF2	0.851002294
ITGAL	PTPRC	0.907208949	SAMSN1	APBB1IP	0.885489142
ITGAL	FYB1	0.904090372	SAMSN1	CD86	0.953191776
ITGAL	IL10RA	0.866433637	SAMSN1	NCF4	0.874712099
ITGAL	LCP2	0.899779573	SAMSN1	MNDA	0.883843827
ITGAL	APBB1IP	0.886522865	SAMSN1	ADAP2	0.878815545
ITGAL	CD86	0.852082239	SAMSN1	LRRC25	0.857475276
ITGAL	SASH3	0.889561284	SAMSN1	MS4A7	0.903587167
ITGAL	WAS	0.896051062	SAMSN1	HAVCR2	0.933769653
ITGAL	CD37	0.86993494	SAMSN1	MS4A4A	0.883014345
ITGAL	EVI2B	0.860040994	SAMSN1	SASH3	0.89790774
ITGAL	FERMT3	0.870631192	SAMSN1	TNFSF13B	0.850493904
ZFP30	ZNF567	0.881210536	SAMSN1	HCK	0.882885279
ZFP30	ZNF420	0.856476966	SAMSN1	CCR1	0.882979916
ZFP30	ZNF607	0.891954969	SAMSN1	MSR1	0.908228241
MORN5	CFAP45	0.861234661	SAMSN1	FGL2	0.889485944
MORN5	FAM183A	0.869568377	SAMSN1	MPEG1	0.85407119
MORN5	C20orf85	0.861584029	SAMSN1	MS4A6A	0.904665932
ANTXR2	IRAG1	0.909939229	SAMSN1	CD37	0.855268978
ANTXR2	CNRIP1	0.878756688	SAMSN1	PLEK	0.908382987
ANTXR2	CCN4	0.929253274	SAMSN1	EVI2B	0.922388581
ANTXR2	FBN1	0.888046077	SAMSN1	FERMT3	0.864245486
ANTXR2	GASK1B	0.92824349	SAMSN1	SELPLG	0.872910032
ANTXR2	CDH11	0.888844829	SAMSN1	C3AR1	0.919757573
ANTXR2	FNDC1	0.852814033	SAMSN1	FCGR2A	0.915636405
ANTXR2	FILIP1L	0.884775282	SAMSN1	GIMAP4	0.870781183
ANTXR2	VCAN	0.855809662	SAMSN1	CYBB	0.913277683
ANTXR2	COL6A3	0.898158056	SAMSN1	ITGB2	0.861503466
ANTXR2	DCN	0.862281779	SAMSN1	RNASE6	0.874464654
ANTXR2	COL5A1	0.875052901	SAMSN1	CD53	0.931664049
ANTXR2	COL1A2	0.867396698	SAMSN1	AIF1	0.85043578
ANTXR2	COL1A1	0.876025747	SAMSN1	FCGR3A	0.906560056
H1-5	H1-4	0.911987976	SAMSN1	LAPTM5	0.907057638
AQP11	EMSY	0.852036241	SAMSN1	SRGN	0.897870852
NBPF12	NBPF11	0.911615349	SAMSN1	C1QA	0.851535679
HIC1	PDGFRA	0.875725815	SAMSN1	C1QB	0.875899189
HIC1	ADGRA2	0.869414159	SAMSN1	C1QC	0.878556254

HIC1	EMILIN1	0.882419439	CLEC7A	ITGAM	0.857268073
ZFP69	EXO5	0.891525681	KLHDC4	MTHFSD	0.859793838
TLR1	TLR2	0.858814578	ZFP82	ZNF566	0.890132724
TLR1	C3AR1	0.855178398	ITGAM	CYTH4	0.852676332
TLR1	ITGB2	0.858305902	ITGAM	IL10RA	0.862653839
NCKAP1L	SAMSN1	0.892068624	ITGAM	LAIR1	0.876193344
NCKAP1L	ITGAM	0.939176205	ITGAM	TLR2	0.870876194
NCKAP1L	CYTH4	0.930041736	ITGAM	ADAP2	0.878482706
NCKAP1L	MYO1F	0.898381255	ITGAM	SLCO2B1	0.869668725
NCKAP1L	ITGAX	0.893761056	ITGAM	MPEG1	0.866165113
NCKAP1L	PTPRC	0.926879215	ITGAM	PLEK	0.852829534
NCKAP1L	LAT2	0.87595212	ITGAM	FERMT3	0.852307305
NCKAP1L	FYB1	0.884784957	ITGAM	C3AR1	0.8549596
NCKAP1L	IL10RA	0.947192123	ITGAM	CYBB	0.902222118
NCKAP1L	GIMAP6	0.886461658	ITGAM	ITGB2	0.872978085
NCKAP1L	LILRB4	0.912552239	ITGAM	CSF1R	0.887691682
NCKAP1L	GNA15	0.867237157	ITGAM	LAPTM5	0.858824652
NCKAP1L	LCP2	0.90621573	ZNF605	ZNF84	0.906658356
NCKAP1L	CD300A	0.891593977	CYTH4	MYO1F	0.90045354
NCKAP1L	LAIR1	0.942378976	CYTH4	FCGR1A	0.85348383
NCKAP1L	NCF2	0.87004612	CYTH4	ITGAX	0.865191966
NCKAP1L	APBB1IP	0.938078595	CYTH4	PTPRC	0.887448634
NCKAP1L	CD86	0.921633033	CYTH4	LAT2	0.881168858
NCKAP1L	SLAMF8	0.850690961	CYTH4	TRPV2	0.854140138
NCKAP1L	TNFAIP8L2	0.862966449	CYTH4	FYB1	0.858335575
NCKAP1L	NCF4	0.882126874	CYTH4	IL10RA	0.933456668
NCKAP1L	MNDA	0.875308038	CYTH4	GIMAP6	0.893818758
NCKAP1L	ADAP2	0.915803501	CYTH4	LILRB4	0.874605219
NCKAP1L	SLCO2B1	0.916211991	CYTH4	LCP2	0.916621751
NCKAP1L	LRRC25	0.917122105	CYTH4	CD300A	0.858454133
NCKAP1L	MS4A7	0.876139156	CYTH4	LAIR1	0.934178146
NCKAP1L	HAVCR2	0.922146044	CYTH4	APBB1IP	0.905619058
NCKAP1L	GPR34	0.877424711	CYTH4	CD86	0.860125372
NCKAP1L	SASH3	0.936361637	CYTH4	SIGLEC1	0.86534613
NCKAP1L	HCK	0.93370309	CYTH4	NCF4	0.910966946
NCKAP1L	ABI3	0.854185733	CYTH4	ARHGAP30	0.85815743
NCKAP1L	FGL2	0.874942157	CYTH4	ADAP2	0.925568241
NCKAP1L	MPEG1	0.928469285	CYTH4	SLCO2B1	0.92275858
NCKAP1L	MS4A6A	0.894096403	CYTH4	LRRC25	0.874017417
NCKAP1L	WAS	0.908522539	CYTH4	HAVCR2	0.868708153
NCKAP1L	CD37	0.87258491	CYTH4	MS4A4A	0.857687738
NCKAP1L	PLEK	0.930872865	CYTH4	SASH3	0.926352332
NCKAP1L	SH2B3	0.856834667	CYTH4	HCK	0.913407532
NCKAP1L	CD163	0.901627074	CYTH4	CCR1	0.859998016
NCKAP1L	EVI2B	0.908044307	CYTH4	ABI3	0.8514399
NCKAP1L	FPR3	0.888006978	CYTH4	MPEG1	0.898149464
NCKAP1L	FERMT3	0.924111553	CYTH4	MS4A6A	0.877207546
NCKAP1L	SELPLG	0.887151868	CYTH4	WAS	0.905474436
NCKAP1L	C3AR1	0.925558836	CYTH4	CD37	0.877888822
NCKAP1L	FCGR2A	0.881906915	CYTH4	LPXN	0.851171432
NCKAP1L	LSP1	0.882236304	CYTH4	PLEK	0.915851099
NCKAP1L	CYBB	0.950415038	CYTH4	CD163	0.884004199
NCKAP1L	ITGB2	0.913958737	CYTH4	EVI2B	0.872482497
NCKAP1L	RNASE6	0.879434647	CYTH4	STAB1	0.898183376
NCKAP1L	CSF1R	0.953068675	CYTH4	FERMT3	0.919221833
NCKAP1L	CD53	0.920315164	CYTH4	SELPLG	0.89969117

NCKAP1L	CD4	0.933596728	CYTH4	C3AR1	0.921866031
NCKAP1L	AIF1	0.852795494	CYTH4	FCGR2A	0.90336568
NCKAP1L	FCGR3A	0.878036629	CYTH4	TNFRSF1B	0.855015539
NCKAP1L	SPI1	0.875261137	CYTH4	CYBB	0.932239143
NCKAP1L	CD14	0.86430239	CYTH4	ITGB2	0.891865239
NCKAP1L	LAPTM5	0.914739076	CYTH4	CSF1R	0.924015919
HOXA3	HOXA5	0.875993732	CYTH4	CD53	0.889519251
CD3D	CD3E	0.954191528	CYTH4	CD4	0.854885629
CD3D	CD2	0.961879778	CYTH4	VSIG4	0.869882939
CD3D	GZMA	0.906438819	CYTH4	FCGR3A	0.868384129
CD3D	IL2RG	0.887818377	CYTH4	CD14	0.887024582
CD3D	NKG7	0.885030614	CYTH4	LAPTM5	0.888785088
CD3D	CCL5	0.907749816	CYTH4	C1QB	0.850693833
ZNF543	ZNF304	0.863600027	CYTH4	C1QC	0.879024835
CCL3L3	CCL4L2	0.88266456	MYO1F	IL10RA	0.866630317
ADAMTS12	LRRC15	0.906282871	MYO1F	LAIR1	0.854058931
ADAMTS12	ADAM12	0.934109431	MYO1F	APBB1IP	0.859990808
ADAMTS12	COL5A3	0.856843999	MYO1F	NCF4	0.862283579
ADAMTS12	ITGA11	0.881188123	MYO1F	WAS	0.898422669
ADAMTS12	FBN1	0.90716532	MYO1F	STAB1	0.879979039
ADAMTS12	ADAMTS2	0.88386429	MYO1F	FERMT3	0.861363015
ADAMTS12	COL11A1	0.918252012	MYO1F	CYBB	0.851082412
ADAMTS12	C1QTNF6	0.863902492	MYO1F	CSF1R	0.876191343
ADAMTS12	SNAI2	0.855299771	CAPSL	ZMYND10	0.854415332
ADAMTS12	VCAN	0.861333995	CAPSL	C5orf49	0.852670356
ADAMTS12	OLFML2B	0.876225183	CAPSL	C20orf85	0.8964007
ADAMTS12	POSTN	0.882778924	ZNF227	ZNF45	0.897910936
ADAMTS12	ECM1	0.889147659	VPS53	SMG6	0.867219304
ADAMTS12	THBS2	0.859791821	ADGRF5	CDH5	0.872484175
ADAMTS12	COL5A2	0.897145401	FCGR1A	LAT2	0.885899546
ADAMTS12	COL6A3	0.865087722	FCGR1A	FYB1	0.86180374
ADAMTS12	MMP11	0.887671539	FCGR1A	LILRB4	0.883763296
ADAMTS12	COL5A1	0.890585097	FCGR1A	LCP2	0.890644057
ADAMTS12	LUM	0.877066364	FCGR1A	CD300A	0.893705174
ADAMTS12	COL3A1	0.902477342	FCGR1A	LAIR1	0.913306512
ADAMTS12	SPARC	0.891267221	FCGR1A	APBB1IP	0.858605293
ADAMTS12	COL1A1	0.868283137	FCGR1A	CD86	0.928170027
FGR	CLEC5A	0.869158134	FCGR1A	TNFAIP8L2	0.900794031
FGR	ITGAM	0.913857655	FCGR1A	NCF4	0.908625785
FGR	CYTH4	0.853351402	FCGR1A	LRRC25	0.903149114
FGR	MYO1F	0.871214654	FCGR1A	HAVCR2	0.912193242
FGR	IGSF6	0.860714837	FCGR1A	MS4A4A	0.856358352
FGR	ITGAX	0.916877802	FCGR1A	SASH3	0.895275618
FGR	LAT2	0.855015195	FCGR1A	HCK	0.876457443
FGR	LILRB4	0.854613256	FCGR1A	CCR1	0.852344855
FGR	OSCAR	0.873375291	FCGR1A	MSR1	0.860876351
FGR	GNA15	0.897059218	FCGR1A	ABI3	0.890242418
FGR	CD300A	0.869519636	FCGR1A	MS4A6A	0.896184796
FGR	LAIR1	0.873443623	FCGR1A	WAS	0.870492257
FGR	NCF2	0.889061354	FCGR1A	CD37	0.891822122
FGR	NCF4	0.852206467	FCGR1A	LY86	0.887510728
FGR	LRRC25	0.857319233	FCGR1A	EVI2B	0.863276642
FGR	HAVCR2	0.861966695	FCGR1A	FERMT3	0.883586913
FGR	SASH3	0.860830442	FCGR1A	SELPLG	0.880908419
FGR	HCK	0.885817049	FCGR1A	C3AR1	0.902073878
FGR	WAS	0.856852516	FCGR1A	FCGR2A	0.898007005

FGR	PLEK	0.856074918	FCGR1A	GIMAP4	0.889964695
FGR	EVI2B	0.851484742	FCGR1A	CYBB	0.86958813
FGR	FERMT3	0.878574478	FCGR1A	RNASE6	0.885146642
FGR	LSP1	0.870777011	FCGR1A	CD53	0.928256352
FGR	CYBB	0.868070086	FCGR1A	AIF1	0.907098552
FGR	ITGB2	0.900500059	FCGR1A	VSIG4	0.870982873
FGR	CD53	0.87273903	FCGR1A	FCGR3A	0.900691896
FGR	CD4	0.859250955	FCGR1A	SPI1	0.899019454
FGR	ALOX5AP	0.880492998	FCGR1A	CD14	0.903249386
FGR	SPI1	0.876110925	FCGR1A	LAPTM5	0.896780815
FGR	LYZ	0.851815802	FCGR1A	FCER1G	0.895620241
FRMD6	CLMP	0.877916352	FCGR1A	C1QA	0.914734832
FRMD6	PRRX1	0.891843689	FCGR1A	TYROBP	0.866110641
H4C4	H1-4	0.857133392	FCGR1A	C1QB	0.908633864
TICRR	FANCI	0.863851898	FCGR1A	C1QC	0.912049227
IRAG1	CCN4	0.888447982	ZSCAN12	ZKSCAN4	0.872795587
IRAG1	FNDC1	0.899289012	ROPN1L	CFAP45	0.866542399
IRAG1	FILIP1L	0.865298865	ROPN1L	WDR38	0.933961086
ZNF141	ZNF721	0.894537348	ROPN1L	ZMYND10	0.900265688
ZNF141	PIGG	0.886746643	ROPN1L	C5orf49	0.921902365
CLEC5A	ITGAM	0.891330038	ROPN1L	FAM183A	0.881746891
CLEC5A	MYO1F	0.852618835	ROPN1L	C20orf85	0.874951986
CLEC5A	ITGAX	0.871642215	ZNF841	ZNF160	0.869149221
CLEC5A	LAT2	0.862410849	ITGAX	PTPRC	0.877028935
CLEC5A	GNA15	0.862039842	ITGAX	IL10RA	0.88399692
CLEC5A	OLR1	0.851114873	ITGAX	LILRB4	0.872868485
CLEC5A	ITGB2	0.86567033	ITGAX	LCP2	0.85916857
CLEC5A	ALOX5AP	0.886284623	ITGAX	LAIR1	0.853430543
CNRIP1	PDGFRA	0.852029126	ITGAX	FERMT3	0.861021517
CLMP	PRRX1	0.891825063	ITGAX	CYBB	0.852409488
CLMP	SNAI2	0.867944722	ITGAX	LAPTM5	0.854491875
CLMP	COL5A2	0.892973452	ZNF433	ZNF791	0.852396151
LRRC15	ADAM12	0.898349022	TMIGD3	ADAP2	0.854545756
LRRC15	INHBA	0.911502686	TMIGD3	HAVCR2	0.869261318
LRRC15	ITGA11	0.92473884	TMIGD3	C3AR1	0.864478213
LRRC15	FBN1	0.860210045	TMIGD3	RNASE6	0.855375801
LRRC15	COL11A1	0.921930011	TMIGD3	ALOX5AP	0.868292813
LRRC15	C1QTNF6	0.863450875	TMIGD3	C1QC	0.860389772
LRRC15	COL8A1	0.865258134	ZNF845	ZNF28	0.850261915
LRRC15	COL10A1	0.903218241	PTPRC	TRPV2	0.855942329
LRRC15	ECM1	0.902704085	PTPRC	FYB1	0.940209147
LRRC15	THBS2	0.926168377	PTPRC	IL2RB	0.854653964
LRRC15	COL5A2	0.850292963	PTPRC	IL10RA	0.942062982
LRRC15	MMP11	0.909518597	PTPRC	GIMAP6	0.885583341
LRRC15	CTSK	0.854100283	PTPRC	LILRB4	0.913865483
LRRC15	MMP2	0.854392913	PTPRC	LCP2	0.947813171
LRRC15	FN1	0.851127018	PTPRC	CD2	0.851296728
LRRC15	COL3A1	0.855933684	PTPRC	LAIR1	0.913093405
MAG	CD22	0.918086699	PTPRC	NCF2	0.877302079
SAMSN1	CLEC7A	0.890872992	PTPRC	APBB1IP	0.888192902
SAMSN1	ITGAM	0.870859452	PTPRC	CD86	0.911595791
SAMSN1	CYTH4	0.873397142	PTPRC	SLAMF8	0.882965404
SAMSN1	FCGR1A	0.856898149	PTPRC	NCF4	0.859513274
SAMSN1	PTPRC	0.947610722	PTPRC	MNDA	0.869330368
SAMSN1	LAT2	0.864605593	PTPRC	ADAP2	0.862107946
SAMSN1	FYB1	0.904189642	PTPRC	SLCO2B1	0.878144427

SAMSN1	IL10RA	0.898126581	PTPRC	MS4A7	0.859142132
SAMSN1	GIMAP6	0.850488769	PTPRC	HAVCR2	0.904025299
SAMSN1	LILRB4	0.900589275	PTPRC	MS4A4A	0.862593705
SAMSN1	LCP2	0.935603976	PTPRC	SASH3	0.930026522
SAMSN1	CD300A	0.864447251	PTPRC	HCK	0.900491498
SAMSN1	LAIR1	0.930648791	PTPRC	CCR1	0.874356718
SAMSN1	TLR2	0.867156357	PTPRC	FGL2	0.914937277
SAMSN1	CD48	0.893981017	PTPRC	MPEG1	0.914635096
SAMSN1	APBB1IP	0.893979112	PTPRC	MS4A6A	0.894115895
SAMSN1	CD86	0.96220937	PTPRC	CD37	0.859347347
SAMSN1	TNFAIP8L2	0.888546463	PTPRC	LPXN	0.85758925
SAMSN1	NCF4	0.891112088	PTPRC	PLEK	0.935601581
SAMSN1	MNDA	0.921433047	PTPRC	EVI2B	0.939287266
SAMSN1	ADAP2	0.855648876	PTPRC	FPR3	0.893215271
SAMSN1	LRRC25	0.874114546	PTPRC	FERMT3	0.884828909
SAMSN1	MS4A7	0.9004925	PTPRC	SELPLG	0.896299668
SAMSN1	HAVCR2	0.956501144	PTPRC	C3AR1	0.902024909
SAMSN1	MS4A4A	0.867820282	PTPRC	FCGR2A	0.882140634
SAMSN1	GPR34	0.869001911	PTPRC	CYBB	0.926859957
SAMSN1	SASH3	0.930347954	PTPRC	ITGB2	0.874971614
SAMSN1	HCK	0.887148434	PTPRC	CD53	0.925081971
SAMSN1	CCR1	0.878270667	PTPRC	FCGR3A	0.888437162
SAMSN1	MSR1	0.871120958	PTPRC	LAPTM5	0.896334535
SAMSN1	ABI3	0.871193495	DEPDC5	PRR14L	0.858541058
SAMSN1	FGL2	0.865539914	CD7	CTSW	0.862777513
SAMSN1	MPEG1	0.855011994	CD7	NKG7	0.85351972
SAMSN1	MS4A6A	0.920474497	CFAP45	WDR38	0.882374609
SAMSN1	WAS	0.889296915	CFAP45	ZMYND10	0.884030428
SAMSN1	CD37	0.893415576	CFAP45	C5orf49	0.851080148
SAMSN1	PLEK	0.913158294	CFAP45	RSPH1	0.88117341
SAMSN1	CD163	0.872798165	CFAP45	PIFO	0.858947888
SAMSN1	EVI2B	0.935202432	CFAP45	TEKT2	0.857168826
SAMSN1	FPR3	0.881322415	CASP8AP2	ZNF292	0.865547722
SAMSN1	FERMT3	0.887046126	ADAM12	COL5A3	0.887736068
SAMSN1	SELPLG	0.870277334	ADAM12	INHBA	0.879848386
SAMSN1	C3AR1	0.927620273	ADAM12	ITGA11	0.880024323
SAMSN1	FCGR2A	0.925305737	ADAM12	ADAMTS2	0.933344018
SAMSN1	GIMAP4	0.889245821	ADAM12	COL11A1	0.86929052
SAMSN1	C1orf162	0.8719841	ADAM12	CRISPLD2	0.876156987
SAMSN1	CYBB	0.915301283	ADAM12	C1QTNF6	0.87348286
SAMSN1	ITGB2	0.870896011	ADAM12	VCAN	0.880933515
SAMSN1	RNASE6	0.916345635	ADAM12	LOX	0.860732106
SAMSN1	CSF1R	0.870281935	ADAM12	OLFML2B	0.855309016
SAMSN1	CD53	0.951938722	ADAM12	POSTN	0.922769063
SAMSN1	CD4	0.894948296	ADAM12	ECM1	0.879760172
SAMSN1	ALOX5AP	0.852669032	ADAM12	THBS2	0.921572239
SAMSN1	AIF1	0.896902764	ADAM12	COL5A2	0.945778713
SAMSN1	VSIG4	0.855224378	ADAM12	COL6A3	0.909958605
SAMSN1	FCGR3A	0.899330058	ADAM12	COL5A1	0.93122545
SAMSN1	SPI1	0.888747118	ADAM12	MMP2	0.909188487
SAMSN1	CD14	0.861531405	ADAM12	FN1	0.871759998
SAMSN1	LAPTM5	0.930079828	ADAM12	COL3A1	0.919356621
SAMSN1	SRGN	0.895477141	ADAM12	COL1A2	0.867850907
SAMSN1	FCER1G	0.875903756	ADAM12	SPARC	0.884591997
SAMSN1	C1QB	0.876538843	ADAM12	COL1A1	0.886908369
SAMSN1	C1QC	0.883086299	WDR38	ZMYND10	0.889883385

CLEC7A	PTPRC	0.856715462	WDR38	C5orf49	0.921021701
CLEC7A	LILRB4	0.850921273	WDR38	C20orf85	0.850943279
CLEC7A	LAIR1	0.86834312	CD3E	CD2	0.969340311
CLEC7A	CD86	0.908986046	CD3E	GZMA	0.873493988
CLEC7A	MNDA	0.86228296	CD3E	SLAMF8	0.850872344
CLEC7A	HAVCR2	0.867810801	CD3E	IL2RG	0.927895267
CLEC7A	PLEK	0.852254643	CD3E	NKG7	0.909049689
CLEC7A	C3AR1	0.860364346	CD3E	CCL5	0.897148709
CLEC7A	FCGR2A	0.852042934	CCNK	BTBD7	0.8506418
CLEC7A	RNASE6	0.858103138	LAT2	FYB1	0.852842367
CLEC7A	CD53	0.866017864	LAT2	LILRB4	0.864308799
ITGAM	CYTH4	0.936867283	LAT2	LCP2	0.880241431
ITGAM	MYO1F	0.915590604	LAT2	CD300A	0.873358002
ITGAM	ITGAX	0.933221509	LAT2	LAIR1	0.887073306
ITGAM	PTPRC	0.88890628	LAT2	APBB1IP	0.92386639
ITGAM	LAT2	0.891652411	LAT2	CD86	0.886257147
ITGAM	IL10RA	0.917577606	LAT2	TNFAIP8L2	0.927004269
ITGAM	LILRB4	0.905432386	LAT2	NCF4	0.892022033
ITGAM	OSCAR	0.888509801	LAT2	ADAP2	0.883714898
ITGAM	GNA15	0.919675057	LAT2	LRRC25	0.878383882
ITGAM	LCP2	0.877953579	LAT2	HAVCR2	0.893699419
ITGAM	CD300A	0.918462842	LAT2	SASH3	0.909688844
ITGAM	LAIR1	0.938014982	LAT2	HCK	0.851124732
ITGAM	TLR2	0.869351145	LAT2	ABI3	0.90649259
ITGAM	NCF2	0.881690579	LAT2	MS4A6A	0.87018767
ITGAM	APBB1IP	0.910583045	LAT2	WAS	0.910657854
ITGAM	CD86	0.891262494	LAT2	CD37	0.913887605
ITGAM	TNFAIP8L2	0.855670871	LAT2	LPXN	0.852977304
ITGAM	NCF4	0.889447409	LAT2	LY86	0.899035501
ITGAM	MNDA	0.873122734	LAT2	EVI2B	0.865514475
ITGAM	ADAP2	0.908895989	LAT2	FERMT3	0.890171245
ITGAM	SLCO2B1	0.911452664	LAT2	SELPLG	0.901099703
ITGAM	LRRC25	0.912875153	LAT2	C3AR1	0.889782138
ITGAM	HAVCR2	0.907070464	LAT2	LSP1	0.884987209
ITGAM	GPR34	0.876872052	LAT2	CYBB	0.879604145
ITGAM	SASH3	0.909536019	LAT2	ITGB2	0.859420656
ITGAM	HCK	0.952594343	LAT2	RNASE6	0.884398913
ITGAM	MPEG1	0.896203664	LAT2	CSF1R	0.888501387
ITGAM	MS4A6A	0.880009577	LAT2	CD53	0.891646676
ITGAM	WAS	0.885698648	LAT2	TREM2	0.869902238
ITGAM	CD37	0.86162986	LAT2	ALOX5AP	0.886327564
ITGAM	PLEK	0.921742193	LAT2	AIF1	0.873769474
ITGAM	SH2B3	0.872579699	LAT2	SPI1	0.926038796
ITGAM	CD163	0.901397272	LAT2	CD14	0.887669914
ITGAM	EVI2B	0.876635865	LAT2	LAPTM5	0.881402585
ITGAM	STAB1	0.859135804	LAT2	FCER1G	0.86003569
ITGAM	FPR3	0.872147216	LAT2	C1QC	0.857116042
ITGAM	FERMT3	0.928823871	ZNF566	ZNF529	0.895034438
ITGAM	SELPLG	0.883514144	ZNF324	ZNF584	0.868964087
ITGAM	C3AR1	0.926596643	ZNF324	ZBTB45	0.861113612
ITGAM	FCGR2A	0.906698726	ZNF324	TRIM28	0.853194441
ITGAM	LSP1	0.873938501	CCN4	ADAMTS2	0.852988542
ITGAM	CYBB	0.945945856	CCN4	CRISPLD2	0.851342406
ITGAM	ITGB2	0.947488819	CCN4	COL8A1	0.86063426
ITGAM	RNASE6	0.851554658	CCN4	VCAN	0.865081288
ITGAM	CSF1R	0.948300072	CCN4	THBS2	0.85909693

ITGAM	CD53	0.902166142	CCN4	COL5A2	0.878223501
ITGAM	CD4	0.921551423	CCN4	COL6A3	0.87350841
ITGAM	ALOX5AP	0.890832129	CCN4	COL5A1	0.859741781
ITGAM	AIF1	0.866541705	CCN4	FN1	0.853579565
ITGAM	VSIG4	0.866093084	CCN4	COL3A1	0.86420177
ITGAM	SPI1	0.886208495	TRPV2	GIMAP6	0.858739358
ITGAM	CD14	0.875217837	TRPV2	LCP2	0.853507571
ITGAM	LAPTM5	0.886565448	TRPV2	HCK	0.878426264
ZNF605	ZNF84	0.860381329	TRPV2	PLEK	0.852599758
CYTH4	MYO1F	0.930071289	TRPV2	FERMT3	0.859935683
CYTH4	FCGR1A	0.902719985	FYB1	IL10RA	0.91612018
CYTH4	DOK3	0.884132575	FYB1	GIMAP6	0.864233931
CYTH4	ITGAX	0.908452969	FYB1	LILRB4	0.88804853
CYTH4	PTPRC	0.893214795	FYB1	LCP2	0.955994709
CYTH4	LAT2	0.910016639	FYB1	LAIR1	0.915443863
CYTH4	NPL	0.852091106	FYB1	APBB1IP	0.93655402
CYTH4	TRPV2	0.873595933	FYB1	CD86	0.930381384
CYTH4	FYB1	0.868696458	FYB1	NCF4	0.87674159
CYTH4	IL10RA	0.955510853	FYB1	MNDA	0.888264498
CYTH4	GIMAP6	0.890851869	FYB1	ADAP2	0.883139983
CYTH4	LILRB4	0.930354385	FYB1	MS4A7	0.873481923
CYTH4	OSCAR	0.868940811	FYB1	HAVCR2	0.909599672
CYTH4	GNA15	0.884398368	FYB1	MS4A4A	0.859588674
CYTH4	LCP2	0.905777819	FYB1	SASH3	0.90989788
CYTH4	CD300A	0.919277822	FYB1	TNFSF13B	0.869529529
CYTH4	LAIR1	0.963220481	FYB1	HCK	0.885247152
CYTH4	TLR2	0.850481809	FYB1	CCR1	0.862541967
CYTH4	APBB1IP	0.918825192	FYB1	FGL2	0.904821874
CYTH4	CD86	0.909212719	FYB1	MPEG1	0.859005999
CYTH4	TNFAIP8L2	0.919118138	FYB1	MS4A6A	0.903555013
CYTH4	NCF4	0.951880062	FYB1	WAS	0.863817975
CYTH4	MNDA	0.867833545	FYB1	CD37	0.920225309
CYTH4	ADAP2	0.931656321	FYB1	LPXN	0.864503497
CYTH4	SLCO2B1	0.914403591	FYB1	PLEK	0.892793783
CYTH4	LRRC25	0.943298377	FYB1	EVI2B	0.956961433
CYTH4	MS4A7	0.89235724	FYB1	FERMT3	0.857091209
CYTH4	HAVCR2	0.89752399	FYB1	SELPLG	0.897453863
CYTH4	GPR34	0.88766934	FYB1	C3AR1	0.891056997
CYTH4	SASH3	0.946147725	FYB1	FCGR2A	0.886724232
CYTH4	HCK	0.93678529	FYB1	GIMAP4	0.926428216
CYTH4	CCR1	0.851053648	FYB1	CYBB	0.91487296
CYTH4	ABI3	0.888276017	FYB1	CD53	0.911128638
CYTH4	MPEG1	0.900902772	FYB1	FCGR3A	0.893858488
CYTH4	MS4A6A	0.912032737	FYB1	SPI1	0.858773088
CYTH4	WAS	0.917104994	FYB1	LAPTM5	0.881152808
CYTH4	CD37	0.886164556	ZNF432	ZNF614	0.8561978
CYTH4	PLEK	0.930761056	HBA1	HBA2	0.897070332
CYTH4	SH2B3	0.871471721	HBA1	HBB	0.886095203
CYTH4	CD163	0.91044491	DOK2	LAIR1	0.868820867
CYTH4	EVI2B	0.857254875	DOK2	SASH3	0.856705078
CYTH4	STAB1	0.909184805	IL2RB	LCP2	0.861720914
CYTH4	FERMT3	0.955226923	COL5A3	ADAMTS2	0.887655558
CYTH4	SELPLG	0.883651437	COL5A3	C1QTNF6	0.854546511
CYTH4	C3AR1	0.937728829	COL5A3	POSTN	0.86139862
CYTH4	FCGR2A	0.891984958	COL5A3	COL5A2	0.885547101
CYTH4	GIMAP4	0.853254072	COL5A3	COL6A3	0.859814537

CYTH4	LSP1	0.865472935	COL5A3	MMP11	0.851274272
CYTH4	CYBB	0.927761767	COL5A3	COL5A1	0.864261371
CYTH4	ITGB2	0.912729918	COL5A3	COL3A1	0.856081517
CYTH4	RNASE6	0.892408661	ZNF439	ZNF700	0.904131351
CYTH4	CSF1R	0.9621179	MCM10	NCAPH	0.853541956
CYTH4	CD53	0.914020192	TRAPPC10	MCM3AP	0.888530566
CYTH4	CD4	0.91655817	SSC5D	MMP19	0.85651275
CYTH4	AIF1	0.905105133	SSC5D	PODN	0.850559994
CYTH4	VSIG4	0.879310499	SSC5D	COL8A1	0.888809225
CYTH4	FCGR3A	0.873781416	SSC5D	THBS2	0.877146721
CYTH4	SPI1	0.919331687	IL10RA	GIMAP6	0.892656445
CYTH4	CD14	0.935112127	IL10RA	LILRB4	0.908282477
CYTH4	LAPTM5	0.896156666	IL10RA	LCP2	0.934161492
CYTH4	C1QA	0.851438845	IL10RA	CD300A	0.851018395
CYTH4	C1QB	0.869786241	IL10RA	LAIR1	0.929536319
CYTH4	C1QC	0.882033103	IL10RA	TLR2	0.874204269
MYO1F	DOK3	0.916401859	IL10RA	APBB1IP	0.909697054
MYO1F	ITGAX	0.930277756	IL10RA	CD86	0.895549752
MYO1F	PTPRC	0.862594337	IL10RA	SIGLEC1	0.884662335
MYO1F	LAT2	0.917615867	IL10RA	NCF4	0.896421564
MYO1F	FYB1	0.867691906	IL10RA	MNDA	0.872506897
MYO1F	IL10RA	0.908511275	IL10RA	ARHGAP30	0.859218501
MYO1F	LILRB4	0.875615825	IL10RA	ADAP2	0.917057414
MYO1F	OSCAR	0.856729885	IL10RA	SLCO2B1	0.929458128
MYO1F	GNA15	0.856515854	IL10RA	LRRC25	0.852154561
MYO1F	LCP2	0.872089077	IL10RA	MS4A7	0.900305698
MYO1F	LAIR1	0.898566688	IL10RA	HAVCR2	0.885088201
MYO1F	APBB1IP	0.91840514	IL10RA	MS4A4A	0.880637796
MYO1F	TNFAIP8L2	0.867820905	IL10RA	SASH3	0.937151567
MYO1F	NCF4	0.883355812	IL10RA	HCK	0.907245825
MYO1F	ARHGAP30	0.86159309	IL10RA	CCR1	0.858617555
MYO1F	ADAP2	0.877289839	IL10RA	FGL2	0.861729839
MYO1F	LRRC25	0.875585393	IL10RA	MPEG1	0.929376441
MYO1F	SASH3	0.907594037	IL10RA	MS4A6A	0.904835957
MYO1F	HCK	0.87599209	IL10RA	WAS	0.871720673
MYO1F	ABI3	0.863159778	IL10RA	CD37	0.859492599
MYO1F	WAS	0.930020617	IL10RA	PLEK	0.937122921
MYO1F	CD37	0.889401091	IL10RA	CD163	0.902643742
MYO1F	LPXN	0.85866606	IL10RA	EVI2B	0.904253229
MYO1F	PLEK	0.850849391	IL10RA	STAB1	0.850684731
MYO1F	STAB1	0.866462079	IL10RA	FPR3	0.864365703
MYO1F	FERMT3	0.926337183	IL10RA	FERMT3	0.906309296
MYO1F	SELPLG	0.876069538	IL10RA	SELPLG	0.89104495
MYO1F	LSP1	0.883671605	IL10RA	C3AR1	0.911761359
MYO1F	CYBB	0.869190343	IL10RA	FCGR2A	0.903508025
MYO1F	ITGB2	0.878969712	IL10RA	TNFRSF1B	0.865324346
MYO1F	CSF1R	0.917668087	IL10RA	CYBB	0.950889115
MYO1F	CD4	0.891728244	IL10RA	ITGB2	0.885073323
MYO1F	SPI1	0.893002606	IL10RA	CSF1R	0.905092901
ASPN	COL11A1	0.875792238	IL10RA	CD53	0.911050992
ASPN	POSTN	0.876692341	IL10RA	VSIG4	0.85642423
ZNF227	ZNF180	0.865487317	IL10RA	FCGR3A	0.887461194
ZNF227	ZNF45	0.924331147	IL10RA	LAPTM5	0.895906793
DNAL1	BBOF1	0.875909683	IL10RA	C1QB	0.85234691
IGSF6	PTPRC	0.86732132	IL10RA	C1QC	0.873576321
IGSF6	SLAMF8	0.857204024	ZNF100	ZNF43	0.856976172

IGSF6	MNDA	0.859030241	SYNRG	AP2B1	0.851748434
IGSF6	HCK	0.866341276	XAF1	PARP14	0.866065382
IGSF6	LST1	0.869943303	EGR3	FOSB	0.894887944
IGSF6	EVI2B	0.885071501	ZNF461	ZNF567	0.898313459
IGSF6	CYBB	0.851070425	ZNF461	ZNF420	0.860665593
IGSF6	ITGB2	0.850278611	ZNF461	ZNF529	0.863248073
IGSF6	CD53	0.851664331	ZNF461	ZNF260	0.897001223
IGSF6	AIF1	0.857626778	COLEC12	PRRX1	0.88848506
IGSF6	LYZ	0.857480494	COLEC12	ADAMTS2	0.858875531
FCGR1A	LAT2	0.858933472	COLEC12	VCAN	0.864591282
FCGR1A	FYB1	0.872085238	COLEC12	OLFML2B	0.879131354
FCGR1A	IL10RA	0.866549971	COLEC12	COL5A2	0.87415034
FCGR1A	LILRB4	0.907804658	COLEC12	COL6A3	0.851636344
FCGR1A	LCP2	0.888686716	ZMYND15	CXCL16	0.858646264
FCGR1A	CD300A	0.856459726	FPR1	MS4A4A	0.855447684
FCGR1A	LAIR1	0.91106136	FPR1	FCGR2A	0.856484061
FCGR1A	APBB1IP	0.861201521	FPR1	VSIG4	0.857644897
FCGR1A	CD86	0.906301925	FPR1	SRGN	0.86493601
FCGR1A	TNFAIP8L2	0.902365519	GIMAP6	LCP2	0.921065556
FCGR1A	NCF4	0.897190577	GIMAP6	GIMAP7	0.89640143
FCGR1A	ADAP2	0.862928251	GIMAP6	LAIR1	0.88211863
FCGR1A	LRRC25	0.887740988	GIMAP6	SIGLEC1	0.855731498
FCGR1A	MS4A7	0.884148044	GIMAP6	ADAP2	0.886734189
FCGR1A	HAVCR2	0.886512038	GIMAP6	SLCO2B1	0.883973178
FCGR1A	GPR34	0.854656975	GIMAP6	HAVCR2	0.850828627
FCGR1A	SASH3	0.884484632	GIMAP6	MS4A4A	0.907503642
FCGR1A	MSR1	0.868648119	GIMAP6	SASH3	0.869212957
FCGR1A	ABI3	0.863947734	GIMAP6	HCK	0.886749356
FCGR1A	MS4A6A	0.876937378	GIMAP6	CCR1	0.91825482
FCGR1A	WAS	0.852756492	GIMAP6	MPEG1	0.894115129
FCGR1A	CD37	0.877920895	GIMAP6	MS4A6A	0.872001223
FCGR1A	LY86	0.86706302	GIMAP6	PLEK	0.900385212
FCGR1A	FERMT3	0.86352614	GIMAP6	CD163	0.892573986
FCGR1A	C3AR1	0.908621064	GIMAP6	EVI2B	0.860979614
FCGR1A	FCGR2A	0.869820862	GIMAP6	C3AR1	0.883387814
FCGR1A	GIMAP4	0.890372456	GIMAP6	FCGR2A	0.874374105
FCGR1A	RNASE6	0.879123556	GIMAP6	GIMAP4	0.889990933
FCGR1A	CD53	0.878248187	GIMAP6	CYBB	0.896913677
FCGR1A	AIF1	0.88983847	GIMAP6	CD53	0.852038813
FCGR1A	VSIG4	0.855484793	GIMAP6	VSIG4	0.852260663
FCGR1A	FCGR3A	0.9048582	GIMAP6	FCGR3A	0.882308801
FCGR1A	SPI1	0.889880839	ADGRL4	CDH5	0.85243203
FCGR1A	CD14	0.896982247	LILRB4	LCP2	0.923408486
FCGR1A	LAPTM5	0.864671982	LILRB4	CD300A	0.893060409
FCGR1A	FCER1G	0.858001932	LILRB4	LAIR1	0.931053491
FCGR1A	C1QA	0.8823192	LILRB4	TLR2	0.876427613
FCGR1A	TYROBP	0.857720985	LILRB4	NCF2	0.896192788
FCGR1A	C1QB	0.888078905	LILRB4	APBB1IP	0.867737027
FCGR1A	C1QC	0.887600904	LILRB4	CD86	0.934598789
ROPN1L	CFAP45	0.879041535	LILRB4	SLAMF8	0.865867083
ROPN1L	ZMYND10	0.878714985	LILRB4	NCF4	0.909529078
ROPN1L	C5orf49	0.857622976	LILRB4	ADAP2	0.851505568
ROPN1L	FAM183A	0.86109643	LILRB4	LRRC25	0.890929423
ROPN1L	C20orf85	0.851674136	LILRB4	HAVCR2	0.926569378
SMG6	ANKFY1	0.862530118	LILRB4	SASH3	0.937063849
DOK3	ITGAX	0.884468689	LILRB4	HCK	0.906307066

DOK3	LAT2	0.859130844	LILRB4	CCR1	0.859118264
DOK3	IL10RA	0.856310296	LILRB4	ABI3	0.872114107
DOK3	LCP2	0.852785655	LILRB4	MS4A6A	0.882553821
DOK3	LAIR1	0.862971525	LILRB4	WAS	0.85860946
DOK3	NCF4	0.866785944	LILRB4	CD37	0.874110415
DOK3	ARHGAP30	0.859959427	LILRB4	PLEK	0.89696778
DOK3	SASH3	0.851436118	LILRB4	EVI2B	0.887405291
DOK3	WAS	0.898199083	LILRB4	FERMT3	0.926019853
DOK3	CD37	0.855722794	LILRB4	SELPLG	0.884693147
DOK3	FERMT3	0.887130237	LILRB4	C3AR1	0.898269297
INVS	STX17	0.850651018	LILRB4	FCGR2A	0.880665639
ITGAX	PTPRC	0.879485697	LILRB4	CYBB	0.904140339
ITGAX	LAT2	0.888113332	LILRB4	ITGB2	0.871927267
ITGAX	FYB1	0.863321161	LILRB4	RNASE6	0.868327854
ITGAX	IL10RA	0.885078555	LILRB4	CD53	0.933857869
ITGAX	LILRB4	0.922220823	LILRB4	FCGR3A	0.865922668
ITGAX	OSCAR	0.88061934	LILRB4	SPI1	0.865250586
ITGAX	GNA15	0.877258297	LILRB4	LAPTM5	0.90534654
ITGAX	LCP2	0.879867202	LILRB4	SRGN	0.853596891
ITGAX	CD300A	0.876319558	LILRB4	C1QA	0.858895038
ITGAX	LAIR1	0.913265444	LILRB4	C1QB	0.868546676
ITGAX	NCF2	0.87334063	LILRB4	C1QC	0.885520399
ITGAX	APBB1IP	0.89761224	ZNF211	ZNF776	0.862095578
ITGAX	CD86	0.871690264	PCNX1	DCAF5	0.860597398
ITGAX	SLAMF8	0.879375522	ZSCAN30	ZNF397	0.892740737
ITGAX	NCF4	0.878820701	ZMYND10	C5orf49	0.881102644
ITGAX	MNDA	0.85221551	ZMYND10	FAM183A	0.866762152
ITGAX	OLR1	0.864157829	ZMYND10	C20orf85	0.85651344
ITGAX	ADAP2	0.854674076	ZMYND10	TEKT2	0.880448638
ITGAX	LRRC25	0.863481165	ETV7	TAP1	0.868779125
ITGAX	HAVCR2	0.887935924	PDGFRA	OLFML1	0.891181655
ITGAX	SASH3	0.889492772	PDGFRA	GASK1B	0.852553693
ITGAX	HCK	0.903342919	PDGFRA	ADGRA2	0.853805891
ITGAX	WAS	0.910844283	PDGFRA	CDH11	0.869587003
ITGAX	CD37	0.88494993	PDGFRA	LHFPL6	0.871567553
ITGAX	PLEK	0.87684465	PDGFRA	EMILIN1	0.880474661
ITGAX	EVI2B	0.86665386	PDGFRA	DCN	0.860304435
ITGAX	FERMT3	0.930916401	GNA15	LAIR1	0.86260944
ITGAX	SELPLG	0.874467989	GNA15	FERMT3	0.876627227
ITGAX	C3AR1	0.871770189	GNA15	SPI1	0.879461607
ITGAX	FCGR2A	0.862344933	GNA15	CD14	0.870313894
ITGAX	LSP1	0.898382873	ZNF567	ZNF420	0.853940668
ITGAX	CYBB	0.889313071	ZNF567	ZNF260	0.913844467
ITGAX	ITGB2	0.917909602	INHBA	ITGA11	0.909934719
ITGAX	CSF1R	0.884502136	INHBA	PDLIM3	0.873811018
ITGAX	CD53	0.874706588	INHBA	ISM1	0.881157821
ITGAX	CD4	0.89299938	INHBA	COL11A1	0.914379005
ITGAX	SPI1	0.905280248	INHBA	CRISPLD2	0.877306007
ITGAX	LAPTM5	0.856559115	INHBA	COL8A1	0.915526847
TMIGD3	LAT2	0.879490171	INHBA	VCAN	0.907726235
TMIGD3	CD300A	0.862250312	INHBA	TMEM158	0.876687524
TMIGD3	APBB1IP	0.869692435	INHBA	POSTN	0.881439015
TMIGD3	CD86	0.869133774	INHBA	THBS2	0.942034529
TMIGD3	NCF4	0.856310105	INHBA	COL5A2	0.91955135
TMIGD3	ADAP2	0.882828573	INHBA	SFRP2	0.868820709
TMIGD3	HAVCR2	0.874114571	INHBA	SERPINE1	0.856403468

TMIGD3	GPR34	0.897280656	INHBA	COL6A3	0.851439546
TMIGD3	SASH3	0.865992069	INHBA	THBS1	0.863784765
TMIGD3	MSR1	0.865519186	INHBA	COL5A1	0.868973844
TMIGD3	C3AR1	0.872897115	INHBA	MMP2	0.888257149
TMIGD3	RNASE6	0.875464386	INHBA	FN1	0.901888513
TMIGD3	TREM2	0.894103777	INHBA	COL3A1	0.871940111
ZNF845	ZNF761	0.856998565	INHBA	SPARC	0.857210808
EDIL3	FBN1	0.874217085	CTSW	CCL5	0.865072743
ZBTB43	GAPVD1	0.860892297	MMP19	COL8A1	0.877993701
PTPRC	LAT2	0.856664986	MMP19	THBS2	0.855789468
PTPRC	FYB1	0.955727743	MMP19	SERPINE1	0.891611454
PTPRC	IL10RA	0.910102515	MMP19	MMP2	0.851943717
PTPRC	GIMAP6	0.880102692	MFAP3	CSNK1A1	0.87560287
PTPRC	LILRB4	0.907222069	C5orf49	FAM183A	0.886019159
PTPRC	LCP2	0.951065035	C5orf49	C20orf85	0.877347183
PTPRC	LAIR1	0.93218427	BIN3	CCAR2	0.850861643
PTPRC	TLR2	0.870381231	RBM28	TNPO3	0.865472838
PTPRC	CD48	0.91028301	ZNF548	ZNF304	0.860882848
PTPRC	NCF2	0.881783034	LCP2	CD300A	0.861582394
PTPRC	APBB1IP	0.922133503	LCP2	LAIR1	0.943525362
PTPRC	CD86	0.939626373	LCP2	TLR2	0.872169349
PTPRC	SLAMF8	0.909430429	LCP2	NCF2	0.87120078
PTPRC	TNFAIP8L2	0.895237735	LCP2	APBB1IP	0.924140786
PTPRC	NCF4	0.864576967	LCP2	CD86	0.949219169
PTPRC	MNDA	0.935287441	LCP2	SLAMF8	0.874547932
PTPRC	LRRC25	0.884740843	LCP2	SIGLEC1	0.856358364
PTPRC	MS4A7	0.874487257	LCP2	NCF4	0.907613184
PTPRC	HAVCR2	0.944906743	LCP2	MNDA	0.890700453
PTPRC	GPR34	0.862485448	LCP2	ARHGAP30	0.853139526
PTPRC	SASH3	0.942789903	LCP2	ADAP2	0.912619058
PTPRC	HCK	0.915979197	LCP2	SLCO2B1	0.86954097
PTPRC	CCR1	0.854869204	LCP2	LRRC25	0.883115729
PTPRC	ABI3	0.862365295	LCP2	MS4A7	0.858960525
PTPRC	FGL2	0.898173771	LCP2	HAVCR2	0.932004538
PTPRC	MPEG1	0.91262888	LCP2	MS4A4A	0.877894768
PTPRC	MS4A6A	0.917220368	LCP2	SASH3	0.947057229
PTPRC	WAS	0.903525978	LCP2	TNFSF13B	0.877481523
PTPRC	CD37	0.917085213	LCP2	HCK	0.920683694
PTPRC	PLEK	0.920931233	LCP2	CCR1	0.918753262
PTPRC	IL2RG	0.854766067	LCP2	MSR1	0.856201317
PTPRC	CD163	0.858941786	LCP2	ABI3	0.887304736
PTPRC	EVI2B	0.971733719	LCP2	FGL2	0.904246416
PTPRC	FPR3	0.893915239	LCP2	MPEG1	0.889493212
PTPRC	FERMT3	0.90351264	LCP2	MS4A6A	0.908140949
PTPRC	SELPLG	0.878647594	LCP2	WAS	0.885803211
PTPRC	C3AR1	0.924291777	LCP2	CD37	0.913213083
PTPRC	FCGR2A	0.901837494	LCP2	LPXN	0.879837857
PTPRC	GIMAP4	0.898043022	LCP2	PLEK	0.912261282
PTPRC	LSP1	0.87169899	LCP2	CD163	0.860213984
PTPRC	CYBB	0.931875451	LCP2	EVI2B	0.943674203
PTPRC	ITGB2	0.903870978	LCP2	FPR3	0.869046295
PTPRC	RNASE6	0.881349535	LCP2	FERMT3	0.903935578
PTPRC	CSF1R	0.897804917	LCP2	SELPLG	0.923995252
PTPRC	CD53	0.953076568	LCP2	C3AR1	0.928642143
PTPRC	CD4	0.924009261	LCP2	FCGR2A	0.917148015
PTPRC	AIF1	0.894895221	LCP2	GIMAP4	0.936862144

PTPRC	FCGR3A	0.888204978	LCP2	CYBB	0.940607945
PTPRC	SPI1	0.891978759	LCP2	ITGB2	0.860699419
PTPRC	LYZ	0.856777571	LCP2	RNASE6	0.877232635
PTPRC	CD14	0.851510347	LCP2	CSF1R	0.859865949
PTPRC	LAPTM5	0.934086412	LCP2	CD53	0.932116354
PTPRC	FCER1G	0.850810668	LCP2	CD4	0.864563598
ZNF180	ZNF45	0.90303371	LCP2	FCGR3A	0.927685877
CFAP45	WDR38	0.859255751	LCP2	SPI1	0.866171596
CFAP45	ZMYND10	0.875135389	LCP2	LAPTM5	0.91166643
ADAM12	INHBA	0.874833805	LCP2	C1QA	0.858441621
ADAM12	ITGA11	0.860601107	LCP2	C1QB	0.87430517
ADAM12	FBN1	0.896461116	LCP2	C1QC	0.885143523
ADAM12	ADAMTS2	0.879849502	ALS2	PIKFYVE	0.864015576
ADAM12	COL11A1	0.913037209	ZNF44	ZNF136	0.878455951
ADAM12	VCAN	0.872993747	ZNF44	ZNF791	0.892612486
ADAM12	LOX	0.8625541	GIMAP7	GIMAP4	0.886598654
ADAM12	OLFML2B	0.859804926	DIP2A	PCNT	0.884582372
ADAM12	POSTN	0.899638973	DIP2A	MCM3AP	0.871029377
ADAM12	ECM1	0.861927208	SNX29	MRTFB	0.877437821
ADAM12	THBS2	0.897001953	SNX29	CLEC16A	0.871983889
ADAM12	COL5A2	0.8943098	PIKFYVE	TRIP12	0.869073821
ADAM12	COL6A3	0.874388114	PINX1	PINX1.1	1
ADAM12	MMP11	0.880069378	ZZEF1	ANKFY1	0.853858293
ADAM12	COL5A1	0.895951824	ZZEF1	MINK1	0.871431865
ADAM12	LUM	0.867642599	PRRX1	FBN1	0.880956929
ADAM12	FN1	0.861383061	PRRX1	VCAM1	0.850077435
ADAM12	COL3A1	0.919926416	PRRX1	GLT8D2	0.880257514
ADAM12	COL1A2	0.856506503	PRRX1	CDH11	0.891172655
ADAM12	SPARC	0.870815025	PRRX1	SNAI2	0.910695216
ADAM12	COL1A1	0.875210049	PRRX1	VCAN	0.881166797
CD3E	IL2RB	0.906177559	PRRX1	COL5A2	0.880348998
CD3E	CD2	0.965232254	PRRX1	COL6A3	0.86532703
CD3E	GZMA	0.924809117	PRRX1	CTSK	0.898045233
CD3E	IL2RG	0.913603388	PRRX1	LUM	0.878463661
CD3E	NKG7	0.886798312	PRRX1	COL3A1	0.852524547
CD3E	CCL5	0.931619405	CD300A	LAIR1	0.899554917
HERC1	ZNF609	0.867330867	CD300A	NCF2	0.865088817
LAT2	FYB1	0.857240217	CD300A	CD86	0.899883372
LAT2	IL10RA	0.886584143	CD300A	TNFAIP8L2	0.853414926
LAT2	LILRB4	0.894256543	CD300A	NCF4	0.885293105
LAT2	OSCAR	0.879146532	CD300A	LRRC25	0.858213712
LAT2	GNA15	0.870606506	CD300A	HAVCR2	0.909720112
LAT2	LCP2	0.899524188	CD300A	SASH3	0.917435072
LAT2	CD300A	0.884310883	CD300A	HCK	0.866091828
LAT2	LAIR1	0.904593389	CD300A	MSR1	0.860265282
LAT2	APBB1IP	0.940958394	CD300A	MS4A6A	0.865788653
LAT2	CD86	0.902756924	CD300A	WAS	0.85823385
LAT2	TNFAIP8L2	0.93310728	CD300A	PLEK	0.867876039
LAT2	NCF4	0.9089628	CD300A	FERMT3	0.923286759
LAT2	MNDA	0.876660808	CD300A	C3AR1	0.897554093
LAT2	ADAP2	0.893054705	CD300A	FCGR2A	0.880521766
LAT2	LRRC25	0.893759324	CD300A	LSP1	0.852154031
LAT2	HAVCR2	0.902044149	CD300A	CYBB	0.861167172
LAT2	GPR34	0.863523552	CD300A	ITGB2	0.876877405
LAT2	SASH3	0.928222537	CD300A	RNASE6	0.862962261
LAT2	HCK	0.854730516	CD300A	CSF1R	0.850683072

LAT2	ABI3	0.935079015	CD300A	CD53	0.931531752
LAT2	MS4A6A	0.850243607	CD300A	ALOX5AP	0.87916702
LAT2	WAS	0.911760745	CD300A	AIF1	0.852734164
LAT2	CD37	0.917637844	CD300A	SPI1	0.876079509
LAT2	LPXN	0.857954517	CD300A	CD14	0.87583704
LAT2	LY86	0.86638012	CD300A	LAPTM5	0.912951144
LAT2	EVI2B	0.850834801	CD300A	SRGN	0.86304458
LAT2	FERMT3	0.915443802	CD300A	C1QA	0.859612944
LAT2	SELPLG	0.881594224	CD300A	C1QB	0.869427841
LAT2	C3AR1	0.879080324	CD300A	C1QC	0.897520971
LAT2	GIMAP4	0.856039292	CD2	CD48	0.854785405
LAT2	C1orf162	0.877174363	CD2	GZMA	0.892978548
LAT2	LSP1	0.875354575	CD2	SLAMF8	0.856753107
LAT2	CYBB	0.878751974	CD2	IL2RG	0.917128807
LAT2	ITGB2	0.859715265	CD2	NKG7	0.899621278
LAT2	RNASE6	0.867049188	CD2	CCL5	0.900703417
LAT2	CSF1R	0.891854697	ITGA11	FBN1	0.865425399
LAT2	CD53	0.881684265	ITGA11	ADAMTS2	0.862938361
LAT2	CD4	0.876556356	ITGA11	ISM1	0.887517196
LAT2	TREM2	0.879953452	ITGA11	COL11A1	0.902144546
LAT2	ALOX5AP	0.890019146	ITGA11	FNDC1	0.871767867
LAT2	AIF1	0.875348012	ITGA11	COL8A1	0.924159617
LAT2	SPI1	0.920853401	ITGA11	COL10A1	0.88517614
LAT2	CD14	0.85038721	ITGA11	VCAN	0.874426374
LAT2	LAPTM5	0.853323291	ITGA11	POSTN	0.858909317
NPL	IL10RA	0.861430616	ITGA11	THBS2	0.931567745
NPL	LAIR1	0.857617232	ITGA11	COL5A2	0.922971294
NPL	NCF4	0.851768264	ITGA11	COL6A3	0.889431631
NPL	ADAP2	0.864077725	ITGA11	CTSK	0.871079158
NPL	SLCO2B1	0.85043122	ITGA11	COL5A1	0.894111593
NPL	MS4A7	0.855805252	ITGA11	MMP2	0.89092627
NPL	GPR34	0.850911589	ITGA11	AEBP1	0.887470264
NPL	MS4A6A	0.862320788	ITGA11	FN1	0.892222326
NPL	PLEK	0.855906262	ITGA11	COL3A1	0.874354035
NPL	CD163	0.856774696	ITGA11	COL1A2	0.882193649
NPL	C3AR1	0.864093906	ITGA11	SPARC	0.852397439
CCN4	FBN1	0.894042252	ITGA11	COL1A1	0.887149933
CCN4	GASK1B	0.872914696	IGLL5	JCHAIN	0.881433294
CCN4	SRPX2	0.853918651	MSRB3	FBN1	0.879559557
CCN4	CDH11	0.853553599	SPRTN	COG2	0.851117696
CCN4	FILIP1L	0.891667263	LAIR1	TLR2	0.879516998
CCN4	ITGA5	0.86227616	LAIR1	NCF2	0.887895617
CCN4	THBS2	0.851907185	LAIR1	APBB1IP	0.922568942
CCN4	COL6A3	0.894912488	LAIR1	CD86	0.941833404
CCN4	COL5A1	0.868544494	LAIR1	SLAMF8	0.876374423
CCN4	COL3A1	0.862502271	LAIR1	TNFAIP8L2	0.858477111
CCN4	COL1A2	0.870462365	LAIR1	NCF4	0.944337025
CCN4	COL1A1	0.870652198	LAIR1	MNDA	0.861026146
TRPV2	GIMAP6	0.861403305	LAIR1	ADAP2	0.918651092
TRPV2	LAIR1	0.856498268	LAIR1	SLCO2B1	0.892795439
TRPV2	SLCO2B1	0.874221008	LAIR1	LRRC25	0.932646442
TRPV2	HCK	0.880857186	LAIR1	MS4A7	0.863726787
TRPV2	MPEG1	0.852027983	LAIR1	HAVCR2	0.948585643
TRPV2	PLEK	0.867768855	LAIR1	MS4A4A	0.910906477
TRPV2	SH2B3	0.897755038	LAIR1	SASH3	0.948153179
TRPV2	FERMT3	0.874756121	LAIR1	HCK	0.937409753

TRPV2	C3AR1	0.851609728	LAIR1	CCR1	0.895000236
TRPV2	CYBB	0.856057905	LAIR1	MSR1	0.860897223
TRPV2	ITGB2	0.851858538	LAIR1	ABI3	0.907353342
TRPV2	CSF1R	0.861320474	LAIR1	MPEG1	0.889994974
FYB1	IL10RA	0.879506715	LAIR1	MS4A6A	0.934178342
FYB1	LILRB4	0.897140043	LAIR1	WAS	0.923169996
FYB1	LCP2	0.93907992	LAIR1	CD37	0.916615518
FYB1	GIMAP7	0.865272585	LAIR1	LPXN	0.868583165
FYB1	LAIR1	0.902308196	LAIR1	PLEK	0.924595324
FYB1	CD48	0.924863097	LAIR1	CD163	0.90324216
FYB1	APBB1IP	0.917238258	LAIR1	EVI2B	0.926999424
FYB1	CD86	0.915808847	LAIR1	FPR3	0.859279432
FYB1	SLAMF8	0.895238035	LAIR1	FERMT3	0.945989522
FYB1	TNFAIP8L2	0.885168646	LAIR1	SELPLG	0.939781285
FYB1	MNDA	0.895645836	LAIR1	C3AR1	0.954153893
FYB1	LRRC25	0.859000991	LAIR1	FCGR2A	0.952235226
FYB1	HAVCR2	0.912762689	LAIR1	GIMAP4	0.891756884
FYB1	SASH3	0.908033312	LAIR1	LSP1	0.851886046
FYB1	TNFSF13B	0.851094676	LAIR1	CYBB	0.94743524
FYB1	HCK	0.86481838	LAIR1	ITGB2	0.918228911
FYB1	ABI3	0.871061365	LAIR1	RNASE6	0.880723985
FYB1	FGL2	0.883178828	LAIR1	CSF1R	0.92412481
FYB1	MPEG1	0.852864974	LAIR1	CD53	0.95768938
FYB1	MS4A6A	0.854226209	LAIR1	CD4	0.879466864
FYB1	WAS	0.914275478	LAIR1	AIF1	0.886723042
FYB1	CD37	0.927875903	LAIR1	VSIG4	0.920984154
FYB1	PLEK	0.866127591	LAIR1	FCGR3A	0.913390086
FYB1	IL2RG	0.869777395	LAIR1	SPI1	0.919792861
FYB1	EVI2B	0.939104827	LAIR1	CD14	0.935216818
FYB1	FERMT3	0.879242346	LAIR1	LAPTM5	0.947581811
FYB1	SELPLG	0.854303415	LAIR1	SRGN	0.855181579
FYB1	C3AR1	0.872791275	LAIR1	C1QA	0.902473317
FYB1	GIMAP4	0.924424345	LAIR1	C1QB	0.921964532
FYB1	C1orf162	0.851615751	LAIR1	C1QC	0.935716043
FYB1	LSP1	0.877756008	TLR2	NCF2	0.853613373
FYB1	CYBB	0.889786031	TLR2	CD86	0.882327604
FYB1	CSF1R	0.855813291	TLR2	ADAP2	0.861532934
FYB1	CD53	0.907114002	TLR2	HAVCR2	0.881974431
FYB1	CD4	0.882820868	TLR2	SASH3	0.857888325
FYB1	AIF1	0.873809592	TLR2	HCK	0.872336776
FYB1	SPI1	0.894842328	TLR2	FERMT3	0.859934512
FYB1	LAPTM5	0.889122303	TLR2	C3AR1	0.86310342
ZNF432	ZNF614	0.851490336	TLR2	CYBB	0.890342182
HBA1	HBB	0.853314105	TLR2	CD53	0.877145022
DOK2	LAIR1	0.867015691	TLR2	LAPTM5	0.870501723
DOK2	NCF4	0.850130839	CD48	LST1	0.875497807
DOK2	SASH3	0.854984016	CD48	GMFG	0.879751684
DOK2	MS4A6A	0.85316239	CD48	CD53	0.867442349
DOK2	FERMT3	0.859696263	CD48	AIF1	0.861093161
DOK2	CD53	0.857172384	OPA3	DMWD	0.871056674
IL2RB	CTSW	0.854714237	TTC5	PIP4P1	0.871990046
IL2RB	CD2	0.88199605	NCF2	CD86	0.881695423
IL2RB	GZMA	0.891784607	NCF2	SLAMF8	0.883263551
IL2RB	IL2RG	0.872343596	NCF2	HAVCR2	0.892372329
IL2RB	NKG7	0.867908528	NCF2	SASH3	0.859688833
IL2RB	CCL5	0.856874618	NCF2	HCK	0.89189545

TRAPPC10	MCM3AP	0.85368286	NCF2	CCR1	0.851191619
IL10RA	GIMAP6	0.911446409	NCF2	PLEK	0.865828472
IL10RA	LILRB4	0.913842437	NCF2	EVI2B	0.860588004
IL10RA	GNA15	0.851397418	NCF2	FPR3	0.867551445
IL10RA	LCP2	0.894024221	NCF2	FERMT3	0.867700488
IL10RA	CD300A	0.870008623	NCF2	C3AR1	0.860536433
IL10RA	LAIR1	0.954873659	NCF2	FCGR2A	0.866539937
IL10RA	APBB1IP	0.912369712	NCF2	CYBB	0.853686019
IL10RA	CD86	0.929925839	NCF2	ITGB2	0.86445467
IL10RA	TNFAIP8L2	0.887055891	NCF2	CD53	0.881431519
IL10RA	SIGLEC1	0.853866725	NCF2	LAPTM5	0.87905132
IL10RA	NCF4	0.926669031	APOL3	GBP4	0.874410872
IL10RA	MNDA	0.851826411	GZMA	NKG7	0.902838704
IL10RA	ADAP2	0.928178491	GZMA	CCL5	0.886698602
IL10RA	SLCO2B1	0.919365691	CKAP2L	BUB1	0.861253991
IL10RA	LRRC25	0.907724491	MRTFB	ZC3H7A	0.879638964
IL10RA	MS4A7	0.923217468	MRTFB	MARF1	0.865775356
IL10RA	HAVCR2	0.901631865	MRTFB	RRN3	0.850468611
IL10RA	MS4A4A	0.861809081	APBB1IP	CD86	0.896911725
IL10RA	GPR34	0.876756446	APBB1IP	NCF4	0.87893954
IL10RA	SASH3	0.938492198	APBB1IP	MNDA	0.883609734
IL10RA	HCK	0.917091379	APBB1IP	ARHGAP30	0.852629708
IL10RA	ABI3	0.875779634	APBB1IP	ADAP2	0.936686233
IL10RA	FGL2	0.877548169	APBB1IP	SLCO2B1	0.870247099
IL10RA	MPEG1	0.91631597	APBB1IP	LRRC25	0.882824817
IL10RA	MS4A6A	0.917373141	APBB1IP	MS4A7	0.863737638
IL10RA	WAS	0.920425357	APBB1IP	HAVCR2	0.910332194
IL10RA	CD37	0.891522416	APBB1IP	GPR34	0.85919891
IL10RA	PLEK	0.91769188	APBB1IP	SASH3	0.914855228
IL10RA	SH2B3	0.863990132	APBB1IP	HCK	0.875715846
IL10RA	CD163	0.909166987	APBB1IP	ABI3	0.863404501
IL10RA	EVI2B	0.876526819	APBB1IP	MPEG1	0.855582465
IL10RA	STAB1	0.881631846	APBB1IP	MS4A6A	0.895610416
IL10RA	FPR3	0.854840588	APBB1IP	WAS	0.912434602
IL10RA	FERMT3	0.929880598	APBB1IP	CD37	0.914573058
IL10RA	SELPLG	0.880053911	APBB1IP	LPXN	0.880911591
IL10RA	C3AR1	0.921998433	APBB1IP	PLEK	0.879704989
IL10RA	FCGR2A	0.880676721	APBB1IP	LY86	0.85421022
IL10RA	GIMAP4	0.865153769	APBB1IP	EVI2B	0.933840723
IL10RA	CYBB	0.929499112	APBB1IP	FERMT3	0.88205208
IL10RA	ITGB2	0.889239417	APBB1IP	SELPLG	0.91584537
IL10RA	RNASE6	0.900780856	APBB1IP	C3AR1	0.921324978
IL10RA	CSF1R	0.951904038	APBB1IP	FCGR2A	0.891838296
IL10RA	CD53	0.909335099	APBB1IP	GIMAP4	0.870321947
IL10RA	CD4	0.908318984	APBB1IP	LSP1	0.851347048
IL10RA	AIF1	0.881791539	APBB1IP	CYBB	0.935118223
IL10RA	VSIG4	0.851286912	APBB1IP	ITGB2	0.878414896
IL10RA	FCGR3A	0.875421824	APBB1IP	RNASE6	0.852110772
IL10RA	SPI1	0.883984686	APBB1IP	CSF1R	0.931077075
IL10RA	CD14	0.899459734	APBB1IP	CD53	0.900152458
IL10RA	LAPTM5	0.888337677	APBB1IP	CD4	0.865508158
IL10RA	C1QB	0.880129507	APBB1IP	ALOX5AP	0.851486555
IL10RA	C1QC	0.887490135	APBB1IP	FCGR3A	0.871907128
ASPM	CENPF	0.85621536	APBB1IP	SPI1	0.888249225
ZNF461	ZNF567	0.888484575	APBB1IP	CD14	0.868980437
ZNF461	ZNF420	0.901241222	APBB1IP	LAPTM5	0.908292247

FPR1	CD300A	0.873780264	APBB1IP	C1QC	0.855370432
FPR1	HCK	0.859161139	CDH5	ESAM	0.881430992
FPR1	MS4A6A	0.866188508	CDH5	NID1	0.858083061
FPR1	CD163	0.876385472	CDH5	VWF	0.855614947
FPR1	C3AR1	0.877674162	CD86	SLAMF8	0.852415673
FPR1	FCGR2A	0.853486001	CD86	TNFAIP8L2	0.866752777
FPR1	CSF1R	0.85729671	CD86	NCF4	0.903721602
FPR1	VSIG4	0.892445233	CD86	MNDA	0.89751085
FPR1	CD14	0.863311039	CD86	ADAP2	0.884483832
MSC	FBN1	0.85904313	CD86	LRRC25	0.910747966
MSC	VCAN	0.853607169	CD86	MS4A7	0.875915455
MSC	DCN	0.868303178	CD86	HAVCR2	0.96008738
MSC	FN1	0.853231489	CD86	MS4A4A	0.870373863
MSC	COL3A1	0.857750844	CD86	SASH3	0.921752677
OTULIN	ICE1	0.854904376	CD86	TNFSF13B	0.884121242
GIMAP6	LCP2	0.864887199	CD86	HCK	0.896239412
GIMAP6	LAIR1	0.880136165	CD86	CCR1	0.904979202
GIMAP6	APBB1IP	0.879173316	CD86	MSR1	0.91499562
GIMAP6	CD86	0.862162551	CD86	ABI3	0.880079666
GIMAP6	ADAP2	0.899889549	CD86	FGL2	0.902127984
GIMAP6	SLCO2B1	0.89473456	CD86	MS4A6A	0.920928174
GIMAP6	LRRC25	0.86117351	CD86	WAS	0.873016783
GIMAP6	MS4A7	0.866430819	CD86	CD37	0.908824611
GIMAP6	HAVCR2	0.857242538	CD86	PLEK	0.884666615
GIMAP6	GPR34	0.884619232	CD86	LY86	0.871089305
GIMAP6	SASH3	0.898012212	CD86	EVI2B	0.934062136
GIMAP6	HCK	0.869223206	CD86	FPR3	0.8804073
GIMAP6	MPEG1	0.879520357	CD86	FERMT3	0.895136994
GIMAP6	MS4A6A	0.901018547	CD86	SELPLG	0.895715102
GIMAP6	PLEK	0.886873617	CD86	C3AR1	0.929709605
GIMAP6	SH2B3	0.863565447	CD86	FCGR2A	0.936724444
GIMAP6	CD163	0.851321942	CD86	GIMAP4	0.923208171
GIMAP6	EVI2B	0.858717521	CD86	C1orf162	0.856376011
GIMAP6	C3AR1	0.889930675	CD86	CYBB	0.91888839
GIMAP6	GIMAP4	0.885958158	CD86	ITGB2	0.865118783
GIMAP6	CYBB	0.881471052	CD86	RNASE6	0.918963852
GIMAP6	CSF1R	0.896752408	CD86	CSF1R	0.85387217
GIMAP6	CD53	0.866916036	CD86	CD53	0.955766763
GIMAP6	FCGR3A	0.865240371	CD86	AIF1	0.883856269
GIMAP6	LAPTM5	0.860372979	CD86	VSIG4	0.853081635
LILRB4	OSCAR	0.861698349	CD86	FCGR3A	0.926638447
LILRB4	GNA15	0.872333769	CD86	SPI1	0.893497823
LILRB4	LCP2	0.924542315	CD86	CD14	0.871757119
LILRB4	CD300A	0.892367799	CD86	LAPTM5	0.937779266
LILRB4	LAIR1	0.95557679	CD86	SRGN	0.897226922
LILRB4	TLR2	0.851987697	CD86	FCER1G	0.87446983
LILRB4	CD48	0.866216087	CD86	C1QA	0.898536893
LILRB4	APBB1IP	0.905738028	CD86	TYROBP	0.853691519
LILRB4	CD86	0.933558772	CD86	C1QB	0.907008992
LILRB4	SLAMF8	0.870884838	CD86	C1QC	0.914694557
LILRB4	TNFAIP8L2	0.906757842	EOLA1	EOLA2	0.85881318
LILRB4	NCF4	0.92669183	FBN1	ADAMTS2	0.851304599
LILRB4	MNDA	0.872448431	FBN1	VCAM1	0.85030124
LILRB4	ADAP2	0.876787791	FBN1	GASK1B	0.851547177
LILRB4	SLCO2B1	0.852946839	FBN1	CRISPLD2	0.85461403
LILRB4	LRRC25	0.902960286	FBN1	CDH11	0.87429024

LILRB4	MS4A7	0.852604031	FBN1	COL8A1	0.870717144
LILRB4	HAVCR2	0.928555199	FBN1	VCAN	0.883736123
LILRB4	SASH3	0.935015792	FBN1	PDGFRB	0.887020888
LILRB4	HCK	0.922342797	FBN1	THBS2	0.861619332
LILRB4	ABI3	0.887896906	FBN1	COL5A2	0.88890176
LILRB4	MPEG1	0.884196557	FBN1	COL6A3	0.885901739
LILRB4	MS4A6A	0.896325883	FBN1	COL5A1	0.865457758
LILRB4	WAS	0.910120496	FBN1	MMP2	0.881632786
LILRB4	CD37	0.899698279	FBN1	COL3A1	0.865128854
LILRB4	PLEK	0.925744101	FBN1	SPARC	0.86637022
LILRB4	CD163	0.856489102	SLAMF8	HAVCR2	0.856506907
LILRB4	EVI2B	0.885071408	SLAMF8	SASH3	0.871065306
LILRB4	FPR3	0.883581409	SLAMF8	IL2RG	0.881192382
LILRB4	FERMT3	0.932028785	SLAMF8	EVI2B	0.852585842
LILRB4	SELPLG	0.902905194	SLAMF8	FPR3	0.853816469
LILRB4	C3AR1	0.928630378	SLAMF8	FERMT3	0.867473725
LILRB4	FCGR2A	0.896102569	SLAMF8	CD53	0.880676605
LILRB4	LSP1	0.872963388	SLAMF8	LAPTM5	0.852344476
LILRB4	CYBB	0.927754521	ADAMTS2	ISM1	0.870342196
LILRB4	ITGB2	0.884020137	ADAMTS2	CRISPLD2	0.869494522
LILRB4	RNASE6	0.884164208	ADAMTS2	VCAN	0.864313034
LILRB4	CSF1R	0.901527997	ADAMTS2	OLFML2B	0.864968624
LILRB4	CD53	0.932270031	ADAMTS2	POSTN	0.86467061
LILRB4	CD4	0.911170241	ADAMTS2	ECM1	0.878501708
LILRB4	AIF1	0.891397579	ADAMTS2	PDGFRB	0.857363337
LILRB4	FCGR3A	0.871798675	ADAMTS2	THBS2	0.894524424
LILRB4	SPI1	0.925091658	ADAMTS2	COL5A2	0.932852596
LILRB4	CD14	0.887880699	ADAMTS2	COL6A3	0.904470945
LILRB4	LAPTM5	0.923910675	ADAMTS2	COL5A1	0.927500719
LILRB4	FCER1G	0.871312347	ADAMTS2	MMP2	0.880904471
LILRB4	C1QB	0.871304109	ADAMTS2	AEBP1	0.85448372
LILRB4	C1QC	0.872426353	ADAMTS2	FN1	0.865811856
ZNF211	ZNF776	0.8639282	ADAMTS2	COL6A2	0.852216461
SPEF1	C5orf49	0.863347017	ADAMTS2	COL3A1	0.905588407
ZMYND10	C5orf49	0.880259262	ADAMTS2	SPARC	0.886245694
ZMYND10	RSPH1	0.854958298	ADAMTS2	COL1A1	0.883736834
ZMYND10	FAM183A	0.856861622	TNFAIP8L2	NCF4	0.89792164
OSCAR	GNA15	0.889114087	TNFAIP8L2	LRRC25	0.865997498
OSCAR	LAIR1	0.875561502	TNFAIP8L2	HAVCR2	0.875741036
OSCAR	CD86	0.873982605	TNFAIP8L2	SASH3	0.886741761
OSCAR	TNFAIP8L2	0.860119895	TNFAIP8L2	ABI3	0.927523442
OSCAR	NCF4	0.85872544	TNFAIP8L2	LST1	0.920882522
OSCAR	MNDA	0.874837062	TNFAIP8L2	MS4A6A	0.886313242
OSCAR	OLR1	0.858775308	TNFAIP8L2	WAS	0.871934961
OSCAR	HAVCR2	0.869464708	TNFAIP8L2	GMFG	0.869321021
OSCAR	SASH3	0.857521013	TNFAIP8L2	CD37	0.884426574
OSCAR	HCK	0.85735975	TNFAIP8L2	LY86	0.930029638
OSCAR	CD37	0.873899164	TNFAIP8L2	FERMT3	0.85669354
OSCAR	FERMT3	0.869790715	TNFAIP8L2	SELPLG	0.85985419
OSCAR	C3AR1	0.8637166	TNFAIP8L2	GIMAP4	0.856459602
OSCAR	ITGB2	0.860820725	TNFAIP8L2	C1orf162	0.860097385
OSCAR	CD53	0.856878207	TNFAIP8L2	RNASE6	0.898378359
OSCAR	AIF1	0.869778453	TNFAIP8L2	CD53	0.898231337
OSCAR	SPI1	0.903816963	TNFAIP8L2	TREM2	0.872089001
PDGFRA	ADGRA2	0.882368163	TNFAIP8L2	AIF1	0.936255586
PDGFRA	LHFPL6	0.886159335	TNFAIP8L2	SPI1	0.930225105

PDGFRA	EMILIN1	0.935169151	TNFAIP8L2	ARHGDIB	0.85457824
GNA15	CD300A	0.876853146	TNFAIP8L2	CD14	0.878097378
GNA15	LAIR1	0.891456717	TNFAIP8L2	FCER1G	0.910659345
GNA15	APBB1IP	0.854438732	TNFAIP8L2	C1QA	0.908006294
GNA15	CD86	0.86169672	TNFAIP8L2	TYROBP	0.911621547
GNA15	NCF4	0.866423355	TNFAIP8L2	C1QB	0.866367455
GNA15	LRRC25	0.865713721	TNFAIP8L2	C1QC	0.877182958
GNA15	HAVCR2	0.871191129	ZNF136	ZNF791	0.9173416
GNA15	SASH3	0.871382815	ZNF562	ZNF426	0.854431465
GNA15	HCK	0.902446753	EVI2A	EVI2B	0.874460367
GNA15	WAS	0.863707616	KBTBD7	KBTBD6	0.85057663
GNA15	PLEK	0.874724893	PPAN	ZNF121	0.851825771
GNA15	FERMT3	0.902638223	PPAN	EIF3G	0.854924485
GNA15	SELPLG	0.862896086	PPAN	CDC37	0.881876104
GNA15	C3AR1	0.870031292	SIGLEC1	ADAP2	0.857929011
GNA15	FCGR2A	0.862498137	SIGLEC1	SLCO2B1	0.861348848
GNA15	LSP1	0.856637032	SIGLEC1	CCR1	0.863755543
GNA15	CYBB	0.88869561	SIGLEC1	MPEG1	0.862128573
GNA15	ITGB2	0.9155187	SIGLEC1	CD163	0.87631353
GNA15	CSF1R	0.885660748	SIGLEC1	CYBB	0.856392962
GNA15	CD53	0.879127284	VCAM1	VCAN	0.87245949
GNA15	CD4	0.869944445	VCAM1	THBS2	0.861319066
GNA15	ALOX5AP	0.870765716	VCAM1	COL5A2	0.863322202
GNA15	AIF1	0.862749429	VCAM1	MMP2	0.851604504
GNA15	SPI1	0.888087768	SRRD	TFIP11	0.889320854
GNA15	CD14	0.850357524	NCF4	ADAP2	0.862044174
GNA15	LAPTM5	0.865821579	NCF4	LRRC25	0.89930689
ZNF567	ZNF420	0.870314573	NCF4	HAVCR2	0.898227574
ZNF567	ZNF607	0.894838865	NCF4	MS4A4A	0.877333736
INHBA	EPYC	0.86289343	NCF4	SASH3	0.941517607
INHBA	ITGA11	0.881882121	NCF4	HCK	0.914893138
INHBA	PDLIM3	0.914858854	NCF4	ABI3	0.928043237
INHBA	COL11A1	0.929169296	NCF4	MS4A6A	0.93869589
INHBA	CRISPLD2	0.857663485	NCF4	WAS	0.933146051
INHBA	COL8A1	0.886504466	NCF4	CD37	0.926241975
INHBA	COL10A1	0.903291592	NCF4	LPXN	0.853578322
INHBA	VCAN	0.85496329	NCF4	PLEK	0.89256331
INHBA	ECM1	0.857504361	NCF4	LY86	0.864983269
INHBA	THBS2	0.951535833	NCF4	EVI2B	0.863888882
INHBA	COL5A2	0.875149892	NCF4	FERMT3	0.932031693
INHBA	SFRP2	0.860342561	NCF4	SELPLG	0.902944694
INHBA	MMP11	0.88166386	NCF4	C3AR1	0.903085144
INHBA	CTSK	0.856993183	NCF4	FCGR2A	0.897596361
INHBA	LUM	0.872786594	NCF4	GIMAP4	0.888280388
INHBA	FN1	0.860086454	NCF4	CYBB	0.89607896
INHBA	COL3A1	0.883356634	NCF4	ITGB2	0.864949419
INHBA	COL1A1	0.860355864	NCF4	RNASE6	0.887523124
KIF15	CKAP2L	0.860043661	NCF4	CSF1R	0.86269553
KIF15	KIF4A	0.875908084	NCF4	CD53	0.93764715
DCHS1	ADGRA2	0.86526534	NCF4	AIF1	0.928775097
EPYC	COL11A1	0.860541279	NCF4	VSIG4	0.893566859
LMOD1	AEBP1	0.856712047	NCF4	FCGR3A	0.855177053
ZNF507	GARRE1	0.887517061	NCF4	SPI1	0.942548215
RBM28	TNPO3	0.872461161	NCF4	CD14	0.92189411
LCP2	CD300A	0.878086463	NCF4	LAPTM5	0.889270297
LCP2	LAIR1	0.931509125	NCF4	FCER1G	0.887121175

LCP2	TLR2	0.873571652	NCF4	C1QA	0.931676867
LCP2	CD48	0.900209946	NCF4	TYROBP	0.857888756
LCP2	APBB1IP	0.93916949	NCF4	C1QB	0.920524227
LCP2	CD86	0.938295709	NCF4	C1QC	0.930891563
LCP2	SLAMF8	0.857210964	ZNF28	ZNF468	0.915115618
LCP2	TNFAIP8L2	0.912579213	G2E3	SCFD1	0.861600922
LCP2	NCF4	0.902330513	C19orf44	CHERP	0.873843091
LCP2	MNDA	0.930641599	MNDA	ADAP2	0.876978242
LCP2	ADAP2	0.872407078	MNDA	MS4A7	0.89006961
LCP2	LRRC25	0.884121827	MNDA	HAVCR2	0.902485048
LCP2	MS4A7	0.855457358	MNDA	SASH3	0.861658817
LCP2	HAVCR2	0.942258517	MNDA	MSR1	0.862094994
LCP2	GPR34	0.865168566	MNDA	FGL2	0.89921153
LCP2	SASH3	0.955588958	MNDA	MS4A6A	0.877489204
LCP2	HCK	0.898106072	MNDA	PLEK	0.86187649
LCP2	CCR1	0.876629979	MNDA	EVI2B	0.904685987
LCP2	MSR1	0.85458835	MNDA	C3AR1	0.880766998
LCP2	ABI3	0.902437771	MNDA	FCGR2A	0.877063227
LCP2	FGL2	0.856883493	MNDA	GIMAP4	0.865139869
LCP2	MPEG1	0.859475212	MNDA	CYBB	0.891273712
LCP2	MS4A6A	0.902157668	MNDA	CD53	0.876661311
LCP2	WAS	0.927570795	MNDA	FCGR3A	0.855691709
LCP2	CD37	0.918667878	MNDA	LAPTM5	0.860493711
LCP2	PLEK	0.9255105	OLFML1	GASK1B	0.860442943
LCP2	EVI2B	0.930626879	OLFML1	CDH11	0.899861607
LCP2	FPR3	0.862102908	OLFML1	FILIP1L	0.874493239
LCP2	FERMT3	0.921749571	OLFML1	DCN	0.875788444
LCP2	SELPLG	0.898988128	OLFML1	TIMP2	0.853481944
LCP2	C3AR1	0.924124959	ANAPC10	LSM6	0.882012118
LCP2	FCGR2A	0.897714232	KIF20B	KIF11	0.909123199
LCP2	GIMAP4	0.901202332	GAPVD1	GOLGA1	0.886274964
LCP2	C1orf162	0.859127922	ZNF776	ZNF587	0.88809445
LCP2	LSP1	0.870498105	ZNF776	ZNF304	0.879491967
LCP2	CYBB	0.92704792	ZNF776	ZNF134	0.855141657
LCP2	ITGB2	0.88012784	PODN	COL8A1	0.876042307
LCP2	RNASE6	0.89367493	ZNF653	PRKCSH	0.881281009
LCP2	CSF1R	0.890266111	HECTD4	SSH1	0.853272887
LCP2	CD53	0.954573276	HECTD4	MED13L	0.851555582
LCP2	CD4	0.910475423	HECTD4	EP400	0.878384056
LCP2	ALOX5AP	0.861716834	HECTD4	UBE3B	0.871507143
LCP2	AIF1	0.896222096	HECTD4	GOLGA3	0.872128602
LCP2	FCGR3A	0.888714446	HECTD4	SETD1B	0.885952695
LCP2	SPI1	0.91732137	USP34	BIRC6	0.891471955
LCP2	CD14	0.860983862	USP34	FBXO11	0.854454196
LCP2	LAPTM5	0.931848897	CCL3	CCL4	0.908903011
LCP2	FCER1G	0.885100377	FAM183A	C20orf85	0.860528994
LCP2	C1QB	0.865979403	ZNF587	ZNF304	0.852451407
LCP2	C1QC	0.870648646	MTRNR2L8	MTRNR2L12	0.912201078
ZNF44	ZNF791	0.863097798	C4A	C4B	0.927479163
GIMAP7	CD48	0.853866384	ISM1	COL11A1	0.86033194
GIMAP7	EVI2B	0.852369084	ISM1	COL8A1	0.88283789
GIMAP7	GIMAP4	0.889923282	ISM1	VCAN	0.861141964
DIP2A	PCNT	0.895419104	ISM1	THBS2	0.872110001
DIP2A	MCM3AP	0.852592519	ISM1	COL5A2	0.908580874
PINX1	PINX1.1	1	ISM1	COL5A1	0.873497539
ZSCAN29	VPS39	0.851038077	ISM1	COL3A1	0.852506402

PRRX1	EDNRA	0.883928692	ISM1	COL1A2	0.856778165
PRRX1	SNAI2	0.897096462	ISM1	SPARC	0.85363951
PRRX1	VCAN	0.904053927	ISM1	COL1A1	0.854775346
PRRX1	COL5A2	0.918748172	OLR1	GPR183	0.881094414
PRRX1	CTSK	0.910265691	ARHGAP30	ADAP2	0.85487821
CD300A	LAIR1	0.911359229	ARHGAP30	CYBB	0.864403452
CD300A	TLR2	0.854446256	GLT8D2	CDH11	0.878252832
CD300A	APBB1IP	0.902119898	GLT8D2	SNAI2	0.856115651
CD300A	CD86	0.884141334	GLT8D2	PDGFRB	0.87108372
CD300A	TNFAIP8L2	0.866238567	GLT8D2	COL5A2	0.853260279
CD300A	NCF4	0.898939106	GLT8D2	COL6A3	0.864274303
CD300A	MNDA	0.862674323	GLT8D2	EMILIN1	0.875709666
CD300A	ADAP2	0.916331728	GLT8D2	ANTXR1	0.854670213
CD300A	SLCO2B1	0.868761465	PIFO	TEKT2	0.879276988
CD300A	LRRC25	0.923610801	COL11A1	VCAN	0.854710008
CD300A	HAVCR2	0.919183492	COL11A1	POSTN	0.863869358
CD300A	GPR34	0.88699643	COL11A1	THBS2	0.884712992
CD300A	SASH3	0.909264741	COL11A1	COL5A2	0.874519957
CD300A	HCK	0.906407303	COL11A1	FN1	0.865020766
CD300A	MSR1	0.860887663	GASK1B	CDH11	0.863161066
CD300A	ABI3	0.852469832	GASK1B	LHFPL6	0.869321378
CD300A	MS4A6A	0.87034722	HELZ	BPTF	0.851111994
CD300A	WAS	0.852692115	ADAP2	SLCO2B1	0.913346334
CD300A	PLEK	0.909829117	ADAP2	LRRC25	0.903099801
CD300A	CD163	0.886079014	ADAP2	MS4A7	0.884240517
CD300A	FPR3	0.861851574	ADAP2	HAVCR2	0.9073215
CD300A	FERMT3	0.900753442	ADAP2	MS4A4A	0.85139774
CD300A	C3AR1	0.924787392	ADAP2	GPR34	0.872700363
CD300A	FCGR2A	0.883796022	ADAP2	SASH3	0.896209167
CD300A	CYBB	0.900771714	ADAP2	HCK	0.88636769
CD300A	ITGB2	0.881782321	ADAP2	CCR1	0.864906182
CD300A	RNASE6	0.875798819	ADAP2	MPEG1	0.907619916
CD300A	CSF1R	0.904415335	ADAP2	MS4A6A	0.887923512
CD300A	CD53	0.91120082	ADAP2	WAS	0.875869258
CD300A	CD4	0.870527375	ADAP2	CD37	0.85024327
CD300A	ALOX5AP	0.877173807	ADAP2	LPXN	0.851202663
CD300A	AIF1	0.875636786	ADAP2	PLEK	0.896396555
CD300A	VSIG4	0.88055094	ADAP2	CD163	0.871163857
CD300A	FCGR3A	0.868562835	ADAP2	EVI2B	0.899656475
CD300A	SPI1	0.872285743	ADAP2	STAB1	0.876304008
CD300A	CD14	0.891277935	ADAP2	FERMT3	0.879779479
CD300A	LAPTM5	0.896364891	ADAP2	SELPLG	0.882004498
CD2	GZMA	0.913604203	ADAP2	C3AR1	0.930151692
CD2	IL2RG	0.93123234	ADAP2	FCGR2A	0.912193539
CD2	NKG7	0.880271253	ADAP2	GIMAP4	0.863401054
CD2	CCL5	0.930025763	ADAP2	CYBB	0.95674085
ITGA11	PDLIM3	0.861832971	ADAP2	ITGB2	0.869018182
ITGA11	FBN1	0.931128087	ADAP2	CSF1R	0.938210584
ITGA11	ADAMTS2	0.853208	ADAP2	CD53	0.892294878
ITGA11	PODN	0.876147246	ADAP2	CD4	0.878120801
ITGA11	COL11A1	0.902643293	ADAP2	VSIG4	0.867008698
ITGA11	CRISPLD2	0.87085217	ADAP2	FCGR3A	0.892095408
ITGA11	FNDC1	0.856566143	ADAP2	CD14	0.87139612
ITGA11	COL8A1	0.907769588	ADAP2	LAPTM5	0.911576165
ITGA11	COL10A1	0.906008606	ADAP2	C1QC	0.875348528
ITGA11	VCAN	0.851449926	TAOK1	NUFIP2	0.886998846

ITGA11	ECM1	0.885231526	SLCO2B1	MS4A7	0.872888257
ITGA11	THBS2	0.936976896	SLCO2B1	MS4A4A	0.860110333
ITGA11	COL5A2	0.884050902	SLCO2B1	GPR34	0.863131302
ITGA11	MMP11	0.869429339	SLCO2B1	SASH3	0.858959708
ITGA11	DCN	0.864436978	SLCO2B1	HCK	0.864989709
ITGA11	THBS1	0.865211584	SLCO2B1	MPEG1	0.940230608
ITGA11	COL5A1	0.88343073	SLCO2B1	MS4A6A	0.854593497
ITGA11	MMP2	0.864306207	SLCO2B1	PLEK	0.898733822
ITGA11	LUM	0.851348155	SLCO2B1	GAL3ST4	0.870158357
ITGA11	AEBP1	0.890552255	SLCO2B1	CD163	0.90859167
ITGA11	FN1	0.878599015	SLCO2B1	EVI2B	0.863712616
ITGA11	COL3A1	0.88113477	SLCO2B1	STAB1	0.886880658
ITGA11	COL1A2	0.864078242	SLCO2B1	C3AR1	0.924626332
ITGA11	COL1A1	0.889958403	SLCO2B1	FCGR2A	0.915236978
IGLL5	JCHAIN	0.892906186	SLCO2B1	CYBB	0.935088528
USP35	KCTD21	0.853455424	SLCO2B1	ITGB2	0.870395126
MSRB3	FBN1	0.87962665	SLCO2B1	CSF1R	0.930817253
MSRB3	VCAN	0.853101299	SLCO2B1	FCGR3A	0.868303113
MSRB3	THBS2	0.857257133	SLCO2B1	LAPTM5	0.881775467
MSRB3	COL5A1	0.865594804	LRRC25	HAVCR2	0.916825944
MSRB3	ACTA2	0.901505921	LRRC25	MS4A4A	0.861294699
MSRB3	AEBP1	0.861165484	LRRC25	SASH3	0.887673111
MSRB3	COL3A1	0.87207941	LRRC25	HCK	0.90690525
MSRB3	COL1A1	0.871033083	LRRC25	CCR1	0.876859626
LAIR1	TLR2	0.866783033	LRRC25	ABI3	0.906784395
LAIR1	CD48	0.852424132	LRRC25	MS4A6A	0.885868015
LAIR1	NCF2	0.85712593	LRRC25	WAS	0.88669239
LAIR1	APBB1IP	0.924575927	LRRC25	CD37	0.878424146
LAIR1	CD86	0.953746311	LRRC25	PLEK	0.867865992
LAIR1	SLAMF8	0.873868572	LRRC25	EVI2B	0.872537928
LAIR1	TNFAIP8L2	0.929516576	LRRC25	FERMT3	0.902176847
LAIR1	NCF4	0.95833176	LRRC25	SELPLG	0.879990115
LAIR1	MNDA	0.896653421	LRRC25	C3AR1	0.91843218
LAIR1	ADAP2	0.925565117	LRRC25	FCGR2A	0.90443581
LAIR1	SLCO2B1	0.906396229	LRRC25	GIMAP4	0.872150461
LAIR1	LRRC25	0.931646148	LRRC25	CYBB	0.909380402
LAIR1	MS4A7	0.915841694	LRRC25	ITGB2	0.858201209
LAIR1	HAVCR2	0.949011146	LRRC25	RNASE6	0.868252625
LAIR1	MS4A4A	0.87735471	LRRC25	CSF1R	0.889850804
LAIR1	GPR34	0.913652707	LRRC25	CD53	0.916014775
LAIR1	SASH3	0.960821996	LRRC25	CD4	0.865206835
LAIR1	HCK	0.952484964	LRRC25	AIF1	0.868104438
LAIR1	CCR1	0.878863669	LRRC25	VSIG4	0.890606131
LAIR1	MSR1	0.867318184	LRRC25	FCGR3A	0.877921792
LAIR1	ABI3	0.913000229	LRRC25	SPI1	0.903491261
LAIR1	FGL2	0.856709496	LRRC25	CD14	0.922430155
LAIR1	MPEG1	0.914472692	LRRC25	LAPTM5	0.90947964
LAIR1	MS4A6A	0.951269852	LRRC25	FCER1G	0.871027578
LAIR1	WAS	0.926770431	LRRC25	C1QA	0.891543724
LAIR1	CD37	0.922969044	LRRC25	C1QB	0.903131059
LAIR1	PLEK	0.945914646	LRRC25	C1QC	0.916190318
LAIR1	SH2B3	0.862268999	MS4A7	HAVCR2	0.871102464
LAIR1	CD163	0.92576475	MS4A7	MS4A4A	0.909091053
LAIR1	EVI2B	0.911652193	MS4A7	GPR34	0.865954
LAIR1	STAB1	0.873710012	MS4A7	MSR1	0.873567251
LAIR1	FPR3	0.899033414	MS4A7	FGL2	0.852294704

LAIR1	FERMT3	0.953849139	MS4A7	MPEG1	0.868984323
LAIR1	SELPLG	0.909323654	MS4A7	MS4A6A	0.912532026
LAIR1	C3AR1	0.965989581	MS4A7	PLEK	0.870604322
LAIR1	FCGR2A	0.938015044	MS4A7	EVI2B	0.880428269
LAIR1	GIMAP4	0.889438225	MS4A7	C3AR1	0.89197515
LAIR1	LSP1	0.881061584	MS4A7	FCGR2A	0.899525219
LAIR1	CYBB	0.962097666	MS4A7	CYBB	0.888938005
LAIR1	ITGB2	0.927515087	MS4A7	CD53	0.869768389
LAIR1	RNASE6	0.928181907	MS4A7	FCGR3A	0.880008836
LAIR1	CSF1R	0.954165499	MS4A7	LAPTM5	0.855031834
LAIR1	CD53	0.959479559	HAVCR2	MS4A4A	0.868167066
LAIR1	CD4	0.93482868	HAVCR2	GPR34	0.850555993
LAIR1	ALOX5AP	0.853837805	HAVCR2	SASH3	0.928277204
LAIR1	AIF1	0.937316294	HAVCR2	HCK	0.91000377
LAIR1	VSIG4	0.907803378	HAVCR2	CCR1	0.895754239
LAIR1	FCGR3A	0.900214398	HAVCR2	MSR1	0.925683594
LAIR1	SPI1	0.941524443	HAVCR2	ABI3	0.879646538
LAIR1	CD14	0.939224946	HAVCR2	FGL2	0.871443037
LAIR1	LAPTM5	0.945238069	HAVCR2	MPEG1	0.855744196
LAIR1	SRGN	0.858882308	HAVCR2	MS4A6A	0.921471044
LAIR1	FCER1G	0.883111112	HAVCR2	WAS	0.863312487
LAIR1	C1QA	0.881988647	HAVCR2	CD37	0.886894941
LAIR1	C1QB	0.909249601	HAVCR2	LPXN	0.865503761
LAIR1	C1QC	0.912644655	HAVCR2	PLEK	0.900775978
TLR2	CD86	0.863658333	HAVCR2	LY86	0.866274116
TLR2	MNDA	0.877404204	HAVCR2	EVI2B	0.934826004
TLR2	HAVCR2	0.865545361	HAVCR2	FPR3	0.855542434
TLR2	SASH3	0.859490745	HAVCR2	FERMT3	0.914019828
TLR2	HCK	0.878920921	HAVCR2	SELPLG	0.905742455
TLR2	PLEK	0.8877874	HAVCR2	C3AR1	0.946116066
TLR2	C3AR1	0.887571245	HAVCR2	FCGR2A	0.937493431
TLR2	FCGR2A	0.851091411	HAVCR2	GIMAP4	0.902918413
TLR2	CYBB	0.895525546	HAVCR2	CYBB	0.928878512
TLR2	ITGB2	0.879395198	HAVCR2	ITGB2	0.890805769
TLR2	CD53	0.880386197	HAVCR2	RNASE6	0.898903059
TLR2	FCGR3A	0.850789291	HAVCR2	CSF1R	0.879636343
TLR2	LAPTM5	0.874958087	HAVCR2	CD53	0.961233379
TIMP3	COL11A1	0.864832976	HAVCR2	CD4	0.863890058
CD48	CD86	0.887703288	HAVCR2	ALOX5AP	0.860659587
CD48	TNFAIP8L2	0.890260115	HAVCR2	AIF1	0.878605134
CD48	EVI2A	0.853365631	HAVCR2	VSIG4	0.874024216
CD48	MNDA	0.878685051	HAVCR2	FCGR3A	0.913132483
CD48	HAVCR2	0.896045997	HAVCR2	SPI1	0.891133787
CD48	SASH3	0.87523859	HAVCR2	CD14	0.887823538
CD48	ABI3	0.855289182	HAVCR2	LAPTM5	0.960583358
CD48	LST1	0.888630043	HAVCR2	SRGN	0.866374624
CD48	WAS	0.851900993	HAVCR2	FCER1G	0.859166122
CD48	GMFG	0.857518472	HAVCR2	C1QA	0.873457129
CD48	CD37	0.887957098	HAVCR2	C1QB	0.898485883
CD48	IL2RG	0.870819535	HAVCR2	C1QC	0.911575705
CD48	LY86	0.854115542	DIS3	PIBF1	0.863067841
CD48	EVI2B	0.908173222	MED26	GTPBP3	0.852606681
CD48	GIMAP4	0.903539225	MED26	EPS15L1	0.899291127
CD48	C1orf162	0.864092711	MED26	SLC35E1	0.88638355
CD48	CD53	0.904699486	MED26	CHERP	0.864199946
CD48	AIF1	0.880416948	MED26	BRD4	0.853815258

CD48	SPI1	0.880382854	MS4A4A	SASH3	0.855789679
CD48	LAPTM5	0.857038514	MS4A4A	HCK	0.878798852
CD48	FCER1G	0.886895436	MS4A4A	CCR1	0.881691136
CD48	TYROBP	0.868905296	MS4A4A	MSR1	0.855753192
NCF2	MNDA	0.863249215	MS4A4A	MPEG1	0.867815467
NCF2	HAVCR2	0.861023396	MS4A4A	MS4A6A	0.924048599
NCF2	SASH3	0.856813643	MS4A4A	PLEK	0.897586646
NCF2	HCK	0.890167848	MS4A4A	CD163	0.903026254
NCF2	MPEG1	0.859350611	MS4A4A	EVI2B	0.863671957
NCF2	PLEK	0.893159369	MS4A4A	C3AR1	0.899275215
NCF2	EVI2B	0.876747823	MS4A4A	FCGR2A	0.915480764
NCF2	FERMT3	0.851114993	MS4A4A	GIMAP4	0.874805006
NCF2	CYBB	0.876953457	MS4A4A	CYBB	0.885989475
NCF2	ITGB2	0.897723458	MS4A4A	CD53	0.888278946
NCF2	CD53	0.875385833	MS4A4A	VSIG4	0.926264572
NCF2	LYZ	0.852831457	MS4A4A	FCGR3A	0.904959721
NCF2	LAPTM5	0.851722027	MS4A4A	CD14	0.852590357
APOL3	APOL6	0.853302874	MS4A4A	LAPTM5	0.85803246
ZNF420	ZNF607	0.865222837	MS4A4A	SRGN	0.858111972
GZMA	IL2RG	0.856698132	MS4A4A	C1QA	0.86596043
GZMA	NKG7	0.935746251	MS4A4A	C1QB	0.904376692
GZMA	CCL5	0.915314429	MS4A4A	C1QC	0.887992681
CMTR2	AP1G1	0.852129337	GPR34	MS4A6A	0.895720505
CKAP2L	CIP2A	0.872085645	GPR34	C3AR1	0.913595583
CKAP2L	DEPDC1	0.850203197	GPR34	FCGR2A	0.873092623
CKAP2L	KIF4A	0.869829429	GPR34	CYBB	0.871309039
CKAP2L	NCAPH	0.890601034	GPR34	CSF1R	0.859471779
CKAP2L	BUB1	0.894516434	GPR34	LAPTM5	0.857917022
CKAP2L	KIF2C	0.856962688	SASH3	HCK	0.92439122
CKAP2L	RACGAP1	0.857706618	SASH3	CCR1	0.86188973
APBB1IP	CD86	0.918070379	SASH3	ABI3	0.924869156
APBB1IP	TNFAIP8L2	0.902277458	SASH3	FGL2	0.862630022
APBB1IP	NCF4	0.882203331	SASH3	MPEG1	0.870668232
APBB1IP	MNDA	0.901033638	SASH3	MS4A6A	0.927493399
APBB1IP	ADAP2	0.92228114	SASH3	WAS	0.915209877
APBB1IP	SLCO2B1	0.853539868	SASH3	CD37	0.92010687
APBB1IP	LRRC25	0.920610018	SASH3	LPXN	0.884560144
APBB1IP	MS4A7	0.850658782	SASH3	PLEK	0.918181283
APBB1IP	HAVCR2	0.939448896	SASH3	CD163	0.860009398
APBB1IP	GPR34	0.882897768	SASH3	EVI2B	0.910371059
APBB1IP	SASH3	0.951544937	SASH3	FERMT3	0.957478292
APBB1IP	HCK	0.895277865	SASH3	SELPLG	0.934401736
APBB1IP	CCR1	0.858529884	SASH3	C3AR1	0.929244359
APBB1IP	ABI3	0.905156558	SASH3	FCGR2A	0.900159623
APBB1IP	MPEG1	0.852341224	SASH3	GIMAP4	0.886752573
APBB1IP	MS4A6A	0.863752765	SASH3	CYBB	0.933517234
APBB1IP	WAS	0.925564617	SASH3	ITGB2	0.902064401
APBB1IP	CD37	0.90651497	SASH3	RNASE6	0.892168347
APBB1IP	PLEK	0.900652064	SASH3	CSF1R	0.893929496
APBB1IP	EVI2B	0.919357505	SASH3	CD53	0.962173105
APBB1IP	FERMT3	0.928891718	SASH3	CD4	0.85971835
APBB1IP	SELPLG	0.881342196	SASH3	AIF1	0.876138329
APBB1IP	C3AR1	0.90758701	SASH3	VSIG4	0.86472342
APBB1IP	FCGR2A	0.856189456	SASH3	FCGR3A	0.890543289
APBB1IP	GIMAP4	0.878111692	SASH3	SPI1	0.906628186
APBB1IP	C1orf162	0.865918105	SASH3	CD14	0.883979818

APBB1IP	LSP1	0.901770184	SASH3	LAPTM5	0.925478909
APBB1IP	CYBB	0.923337371	SASH3	C1QA	0.902584489
APBB1IP	ITGB2	0.900765129	SASH3	C1QB	0.909147439
APBB1IP	RNASE6	0.863172002	SASH3	C1QC	0.926795417
APBB1IP	CSF1R	0.923861811	DNAJC16	FBXO42	0.850634949
APBB1IP	CD53	0.919287421	TNFSF13B	CCR1	0.862963438
APBB1IP	CD4	0.903718332	TNFSF13B	FGL2	0.85600231
APBB1IP	ALOX5AP	0.879952467	TNFSF13B	EVI2B	0.856309754
APBB1IP	AIF1	0.864198884	TNFSF13B	GIMAP4	0.903272856
APBB1IP	FCGR3A	0.853329052	TNFSF13B	CTSS	0.853009305
APBB1IP	SPI1	0.903107891	CRISPLD2	COL8A1	0.88098506
APBB1IP	LAPTM5	0.908832127	CRISPLD2	VCAN	0.900103625
KCNAB2	FERMT3	0.852606708	CRISPLD2	THBS2	0.901209124
CD86	TNFAIP8L2	0.921754523	CRISPLD2	COL5A2	0.884514114
CD86	NCF4	0.919807615	CRISPLD2	SERPINE1	0.850794154
CD86	MNDA	0.920080692	CRISPLD2	COL5A1	0.860440793
CD86	ADAP2	0.895588673	CRISPLD2	MMP2	0.871971811
CD86	LRRC25	0.908988183	CRISPLD2	FN1	0.860816609
CD86	MS4A7	0.921512944	CRISPLD2	COL3A1	0.863692102
CD86	HAVCR2	0.967490695	CRISPLD2	SPARC	0.86288896
CD86	MS4A4A	0.860368184	ZNF304	ZNF134	0.858289371
CD86	GPR34	0.886048544	ZNF506	ZNF253	0.866644986
CD86	SASH3	0.942971433	MCM8	TRMT6	0.85999319
CD86	HCK	0.90261065	SAMD9L	RSAD2	0.852278256
CD86	CCR1	0.88359816	SAMD9L	IFIT2	0.895305965
CD86	MSR1	0.900149128	SAMD9L	OAS2	0.885873824
CD86	ABI3	0.899364803	SAMD9L	IFIT3	0.8644761
CD86	FGL2	0.887457066	DDX60	OASL	0.865744457
CD86	MPEG1	0.875338105	DDX60	CMPK2	0.886197707
CD86	MS4A6A	0.926505833	DDX60	HERC6	0.870049547
CD86	WAS	0.915750805	DDX60	RSAD2	0.864389231
CD86	CD37	0.923048115	DDX60	IFI44L	0.874459575
CD86	PLEK	0.919040835	DDX60	OAS2	0.900369977
CD86	CD163	0.883765523	DDX60	OAS1	0.866854165
CD86	LY86	0.876644343	DDX60	IFIT1	0.850790267
CD86	EVI2B	0.930404603	DDX60	OAS3	0.861128513
CD86	FPR3	0.895505072	DDX60	MX1	0.885633314
CD86	FERMT3	0.914975872	DDX60	IFI44	0.885998075
CD86	SELPLG	0.90469135	DDX60	IFIT3	0.883467232
CD86	C3AR1	0.949748896	VPS8	SENP2	0.866353091
CD86	FCGR2A	0.935885467	HCK	CCR1	0.876595016
CD86	GIMAP4	0.916826586	HCK	ABI3	0.874269142
CD86	C1orf162	0.887005299	HCK	MPEG1	0.88627784
CD86	LSP1	0.879171159	HCK	MS4A6A	0.895872137
CD86	CYBB	0.929876093	HCK	WAS	0.874034176
CD86	ITGB2	0.881450913	HCK	CD37	0.874797063
CD86	RNASE6	0.946988911	HCK	PLEK	0.929891472
CD86	CSF1R	0.904383015	HCK	CD163	0.867655901
CD86	CD53	0.958474282	HCK	EVI2B	0.891756704
CD86	CD4	0.910648049	HCK	FERMT3	0.926170378
CD86	TREM2	0.851209439	HCK	SELPLG	0.880335931
CD86	AIF1	0.915234787	HCK	C3AR1	0.925286796
CD86	VSIG4	0.866028508	HCK	FCGR2A	0.888819089
CD86	FCGR3A	0.922468407	HCK	GIMAP4	0.870081012
CD86	CTSS	0.852439334	HCK	CYBB	0.919257373
CD86	SPI1	0.918210999	HCK	ITGB2	0.886276429

CD86	CD14	0.897624309	HCK	CSF1R	0.865568216
CD86	LAPTM5	0.942340299	HCK	CD53	0.921085589
CD86	SRGN	0.886873742	HCK	AIF1	0.861966764
CD86	FCER1G	0.899993805	HCK	VSIG4	0.858319474
CD86	C1QA	0.884850449	HCK	FCGR3A	0.875086717
CD86	TYROBP	0.878628105	HCK	SPI1	0.87542568
CD86	C1QB	0.909682193	HCK	CD14	0.87003766
CD86	C1QC	0.917899986	HCK	LAPTM5	0.895696888
ZNF367	SMC2	0.852414735	HCK	C1QA	0.866766996
PDLIM3	FBN1	0.859840683	HCK	C1QB	0.868963949
PDLIM3	COL8A1	0.901955804	HCK	C1QC	0.888262461
PDLIM3	COL10A1	0.892307419	C1QTNF6	CREB3L1	0.856689518
PDLIM3	THBS2	0.935572673	C1QTNF6	COL5A2	0.882643653
PDLIM3	ACTA2	0.879780382	C1QTNF6	COL6A3	0.876747812
PDLIM3	FN1	0.86623537	C1QTNF6	COL5A1	0.87635238
PDLIM3	COL3A1	0.853311278	C1QTNF6	COL3A1	0.858518919
PDLIM3	COL1A1	0.856490381	C1QTNF6	SPARC	0.850879249
EOLA1	EOLA2	0.900434856	COL16A1	COL6A3	0.856870625
FBN1	ADAMTS2	0.884622182	COL16A1	AEBP1	0.850705973
FBN1	COL11A1	0.912527938	CCR1	MSR1	0.866485535
FBN1	ACTG2	0.854304338	CCR1	MPEG1	0.859775803
FBN1	CRISPLD2	0.888534861	CCR1	PLEK	0.895972424
FBN1	CDH11	0.890497	CCR1	CD163	0.885420653
FBN1	COL8A1	0.881176191	CCR1	EVI2B	0.881551279
FBN1	SNAI2	0.888070794	CCR1	FPR3	0.885113358
FBN1	FILIP1L	0.861050042	CCR1	C3AR1	0.906052887
FBN1	VCAN	0.9084215	CCR1	FCGR2A	0.902042582
FBN1	LOX	0.861435592	CCR1	GIMAP4	0.892049829
FBN1	ECM1	0.85180698	CCR1	CYBB	0.894918855
FBN1	LOXL2	0.869494099	CCR1	CD53	0.89369243
FBN1	ANGPTL2	0.858975242	CCR1	VSIG4	0.856671992
FBN1	PDGFRB	0.875862888	CCR1	FCGR3A	0.926519827
FBN1	THBS2	0.912663573	CCR1	LAPTM5	0.890614402
FBN1	COL5A2	0.935148015	CCR1	C1QB	0.875347596
FBN1	COL6A3	0.910268783	CCR1	C1QC	0.876845248
FBN1	ANTXR1	0.877024686	MSR1	FGL2	0.862851385
FBN1	DCN	0.906510285	MSR1	MS4A6A	0.875323186
FBN1	COL5A1	0.935444388	MSR1	PLEK	0.850284969
FBN1	ACTA2	0.856343428	MSR1	EVI2B	0.87696825
FBN1	MMP2	0.855708788	MSR1	FPR3	0.868909475
FBN1	LUM	0.892551712	MSR1	C3AR1	0.90454691
FBN1	AEBP1	0.904738283	MSR1	FCGR2A	0.919771522
FBN1	FN1	0.903297572	MSR1	CYBB	0.865365652
FBN1	COL3A1	0.935215838	MSR1	RNASE6	0.85116887
FBN1	COL1A2	0.914447662	MSR1	CD53	0.895183414
FBN1	SPARC	0.934019684	MSR1	FCGR3A	0.913990784
FBN1	COL1A1	0.926069068	MSR1	LAPTM5	0.905496588
SLAMF8	HCK	0.874726308	MSR1	SRGN	0.872311186
SLAMF8	MPEG1	0.868606166	SRPX2	CDH11	0.862984075
SLAMF8	CD37	0.854616313	SRPX2	CREB3L1	0.887493377
SLAMF8	PLEK	0.860690972	SRPX2	COL6A3	0.852548969
SLAMF8	IL2RG	0.874357277	ABI3	LST1	0.861765979
SLAMF8	EVI2B	0.886555731	ABI3	MS4A6A	0.885005907
SLAMF8	FERMT3	0.868095648	ABI3	WAS	0.907890264
SLAMF8	CYBB	0.871263071	ABI3	CD37	0.905360586
SLAMF8	ITGB2	0.868034511	ABI3	LY86	0.860387394

SLAMF8	CD53	0.862321616	ABI3	FERMT3	0.915983598
SLAMF8	CD4	0.865238367	ABI3	SELPLG	0.915769873
SLAMF8	LAPTM5	0.850213265	ABI3	C3AR1	0.859446639
ADAMTS2	COL11A1	0.871523829	ABI3	GIMAP4	0.890165786
ADAMTS2	ECM1	0.856547082	ABI3	CYBB	0.863332155
ADAMTS2	THBS2	0.877785543	ABI3	RNASE6	0.86635735
ADAMTS2	COL5A2	0.868457815	ABI3	CD53	0.905417665
ADAMTS2	COL5A1	0.867427002	ABI3	CD4	0.855352343
ADAMTS2	COL3A1	0.898039672	ABI3	AIF1	0.904402304
ADAMTS2	SPARC	0.869022894	ABI3	SPI1	0.938730164
ADAMTS2	COL1A1	0.868829721	ABI3	CD14	0.892967367
SGO1	ECT2	0.855148758	ABI3	LAPTM5	0.853509447
ZNF707	SCRIB	0.873296967	ABI3	FCER1G	0.893716468
TNFAIP8L2	NCF4	0.933766855	ABI3	C1QA	0.913142629
TNFAIP8L2	MNDA	0.908965497	ABI3	C1QB	0.889781295
TNFAIP8L2	ADAP2	0.86763751	ABI3	C1QC	0.899223574
TNFAIP8L2	LRRC25	0.906780516	PCNT	MCM3AP	0.872807443
TNFAIP8L2	MS4A7	0.864762671	ZNF106	AQR	0.852815907
TNFAIP8L2	HAVCR2	0.915173762	AKAP13	ABHD2	0.854496344
TNFAIP8L2	GPR34	0.892408562	LST1	GMFG	0.936908951
TNFAIP8L2	SASH3	0.946256353	LST1	LY86	0.905630175
TNFAIP8L2	HCK	0.883118875	LST1	C1orf162	0.859734963
TNFAIP8L2	ABI3	0.936691369	LST1	RNASE6	0.859243634
TNFAIP8L2	LST1	0.905780084	LST1	AIF1	0.938238109
TNFAIP8L2	MS4A6A	0.918288529	LST1	SPI1	0.888718462
TNFAIP8L2	WAS	0.902912543	LST1	HLA-DPB1	0.868640439
TNFAIP8L2	GMFG	0.860738506	LST1	FCER1G	0.908148429
TNFAIP8L2	CD37	0.92975866	LST1	C1QA	0.88060471
TNFAIP8L2	PLEK	0.870908352	LST1	TYROBP	0.926464076
TNFAIP8L2	LY86	0.899792836	AQR	EIF2AK4	0.857082279
TNFAIP8L2	EVI2B	0.886124059	H3C4	H2AC7	0.919933236
TNFAIP8L2	FERMT3	0.911155217	FGL2	MPEG1	0.867703202
TNFAIP8L2	SELPLG	0.895596002	FGL2	MS4A6A	0.895115531
TNFAIP8L2	C3AR1	0.918905116	FGL2	PLEK	0.866351488
TNFAIP8L2	FCGR2A	0.86427624	FGL2	EVI2B	0.914336952
TNFAIP8L2	GIMAP4	0.907995842	FGL2	C3AR1	0.875893521
TNFAIP8L2	C1orf162	0.869451042	FGL2	FCGR2A	0.863715479
TNFAIP8L2	LSP1	0.87027912	FGL2	GIMAP4	0.852808513
TNFAIP8L2	CYBB	0.888264356	FGL2	CYBB	0.890177902
TNFAIP8L2	ITGB2	0.853795396	FGL2	RNASE6	0.850720128
TNFAIP8L2	RNASE6	0.918445119	FGL2	CD53	0.887360894
TNFAIP8L2	CSF1R	0.884312263	FGL2	FCGR3A	0.891674211
TNFAIP8L2	CD53	0.929117264	FGL2	LAPTM5	0.854601738
TNFAIP8L2	CD4	0.899114544	CXCL11	GBP4	0.861383112
TNFAIP8L2	TREM2	0.864077888	CXCL11	CXCL10	0.928769411
TNFAIP8L2	AIF1	0.937157978	ADGRA2	LHFPL6	0.85805412
TNFAIP8L2	SPI1	0.949931122	ADGRA2	NID1	0.855415962
TNFAIP8L2	CD14	0.910130655	ADGRA2	PDGFRB	0.908799434
TNFAIP8L2	LAPTM5	0.898019948	ADGRA2	EMILIN1	0.866844897
TNFAIP8L2	FCER1G	0.903200436	FAM210A	SEH1L	0.855118598
TNFAIP8L2	C1QA	0.882356179	SIRT7	DUS1L	0.881553264
TNFAIP8L2	TYROBP	0.894614767	EP400	GOLGA3	0.891286378
TNFAIP8L2	C1QB	0.875216922	CDK12	MED1	0.894963448
TNFAIP8L2	C1QC	0.883435992	FBXO45	SENP5	0.863783846
MGA	TP53BP1	0.854671685	FBXO45	PAK2	0.88510551
ZNF160	ZNF761	0.871017277	CACTIN	FZR1	0.856757306

EMSY	RSF1	0.884757201	C8orf76	MRPL13	0.856805754
EMSY	INTS4	0.892468908	OASL	CMPK2	0.916744538
EVI2A	MNDA	0.853904926	OASL	HERC6	0.854317281
EVI2A	HAVCR2	0.865627454	OASL	RSAD2	0.912288145
EVI2A	LST1	0.866322582	OASL	IFIH1	0.86930195
EVI2A	LY86	0.854888871	OASL	IFI44L	0.889333306
EVI2A	EVI2B	0.873408661	OASL	OAS2	0.907183616
EVI2A	LY96	0.872261089	OASL	OAS1	0.89370757
EVI2A	AIF1	0.863346555	OASL	IFIT1	0.896650272
KBTBD7	KBTBD6	0.86340454	OASL	OAS3	0.872460251
COG8	TERF2	0.870584308	OASL	MX1	0.902979862
STX17	NCBP1	0.861648094	OASL	IFI44	0.887394205
SIGLEC1	NCF4	0.850096302	OASL	IFIT3	0.909080797
SIGLEC1	ADAP2	0.865984116	HERC5	CMPK2	0.896567598
SIGLEC1	STAB1	0.867841754	TRAPPC8	ZNF24	0.872520361
SIGLEC1	CSF1R	0.855351272	TRAPPC8	DSG2	0.861719167
SIGLEC1	C1QC	0.859306423	WIPF1	DAB2	0.858875163
ZSCAN25	ZKSCAN5	0.875672975	COPZ2	VCAN	0.862661164
ZSCAN25	TRRAP	0.851385322	COPZ2	CREB3L1	0.854667484
NCF4	MNDA	0.855248303	COPZ2	THBS2	0.860800074
NCF4	ADAP2	0.902918141	COPZ2	COL5A2	0.856319445
NCF4	SLCO2B1	0.863688469	COPZ2	COL6A3	0.853957518
NCF4	LRRC25	0.904970478	COPZ2	COL5A1	0.879064963
NCF4	MS4A7	0.90376819	COPZ2	MMP2	0.881711741
NCF4	HAVCR2	0.907668088	COPZ2	COL3A1	0.882925611
NCF4	GPR34	0.889705557	COPZ2	SPARC	0.899949504
NCF4	SASH3	0.945853856	COPZ2	COL1A1	0.880010309
NCF4	HCK	0.902323465	MPEG1	MS4A6A	0.874881118
NCF4	ABI3	0.917580778	MPEG1	PLEK	0.926477224
NCF4	MS4A6A	0.926427008	MPEG1	CD163	0.886707867
NCF4	WAS	0.916558215	MPEG1	EVI2B	0.894839447
NCF4	CD37	0.909750219	MPEG1	FPR3	0.862646726
NCF4	PLEK	0.904602572	MPEG1	SELPLG	0.850680779
NCF4	CD163	0.886654828	MPEG1	C3AR1	0.905749718
NCF4	STAB1	0.895417476	MPEG1	FCGR2A	0.897910866
NCF4	FERMT3	0.942113387	MPEG1	CYBB	0.949801216
NCF4	SELPLG	0.910656687	MPEG1	ITGB2	0.865636866
NCF4	C3AR1	0.93252925	MPEG1	CSF1R	0.889624194
NCF4	FCGR2A	0.905165865	MPEG1	CD53	0.87413897
NCF4	GIMAP4	0.863027572	MPEG1	CD4	0.863082842
NCF4	C1orf162	0.860541403	MPEG1	FCGR3A	0.870365756
NCF4	LSP1	0.863800317	MPEG1	LAPTM5	0.884733517
NCF4	CYBB	0.905698213	HOXB6	HOXB5	0.906903005
NCF4	ITGB2	0.867688561	HOXB6	HOXB8	0.884051177
NCF4	RNASE6	0.927708445	FDX2	PIN1	0.868868912
NCF4	CSF1R	0.925850662	RO60	CDC73	0.911944719
NCF4	CD53	0.929949143	MS4A6A	WAS	0.863214497
NCF4	CD4	0.892301977	MS4A6A	CD37	0.889431732
NCF4	TREM2	0.864491186	MS4A6A	LPXN	0.868194866
NCF4	AIF1	0.923511392	MS4A6A	PLEK	0.909723729
NCF4	VSIG4	0.891286574	MS4A6A	LY86	0.875701172
NCF4	FCGR3A	0.864345238	MS4A6A	EVI2B	0.921209399
NCF4	SPI1	0.934153248	MS4A6A	FERMT3	0.882939785
NCF4	CD14	0.936599888	MS4A6A	SELPLG	0.903172597
NCF4	LAPTM5	0.905327476	MS4A6A	C3AR1	0.937886972
NCF4	FCER1G	0.886349355	MS4A6A	FCGR2A	0.92730594

NCF4	C1QA	0.906720538	MS4A6A	GIMAP4	0.900808346
NCF4	TYROBP	0.8727504	MS4A6A	CYBB	0.927986398
NCF4	C1QB	0.916195393	MS4A6A	ITGB2	0.865492114
NCF4	C1QC	0.923982243	MS4A6A	RNASE6	0.918917849
CIP2A	RACGAP1	0.867332999	MS4A6A	CSF1R	0.872090531
ZNF28	ZNF468	0.875920065	MS4A6A	CD53	0.956342327
MNDA	LRRC25	0.865964961	MS4A6A	AIF1	0.926908546
MNDA	HAVCR2	0.932031892	MS4A6A	VSIG4	0.890825066
MNDA	GPR34	0.877236824	MS4A6A	FCGR3A	0.896810745
MNDA	SASH3	0.917219017	MS4A6A	SPI1	0.881677737
MNDA	HCK	0.901986448	MS4A6A	CD14	0.887121545
MNDA	CCR1	0.866594742	MS4A6A	LAPTM5	0.912348737
MNDA	ABI3	0.853863182	MS4A6A	SRGN	0.8735819
MNDA	LST1	0.852659943	MS4A6A	C1QA	0.907870066
MNDA	MS4A6A	0.898408185	MS4A6A	C1QB	0.921869059
MNDA	WAS	0.857680821	MS4A6A	C1QC	0.920166111
MNDA	CD37	0.891412897	CD93	SH2B3	0.855229852
MNDA	PLEK	0.898400403	CD93	DAB2	0.864929757
MNDA	LY86	0.850179114	CDH11	COL8A1	0.867275242
MNDA	EVI2B	0.94095115	CDH11	SNAI2	0.890601456
MNDA	FPR3	0.860931566	CDH11	FILIP1L	0.88895092
MNDA	FERMT3	0.870737756	CDH11	VCAN	0.87362744
MNDA	SELPLG	0.858320271	CDH11	CREB3L1	0.857105229
MNDA	C3AR1	0.927729742	CDH11	COL5A2	0.883052102
MNDA	FCGR2A	0.882798123	CDH11	COL6A3	0.912357411
MNDA	GIMAP4	0.888604817	CDH11	EMILIN1	0.89396367
MNDA	LSP1	0.859395474	CDH11	ANTXR1	0.868769383
MNDA	CYBB	0.902048091	CDH11	DCN	0.901020684
MNDA	ITGB2	0.898345407	CDH11	COL5A1	0.869102396
MNDA	RNASE6	0.898368404	CDH11	MMP2	0.901295349
MNDA	CSF1R	0.866666436	CDH11	AEBP1	0.875500556
MNDA	CD53	0.936939082	CDH11	TIMP2	0.864694298
MNDA	CD4	0.885690598	CDH11	COL3A1	0.881792775
MNDA	ALOX5AP	0.880269965	CDH11	SPARC	0.861296729
MNDA	AIF1	0.894685537	CDH11	COL1A1	0.85585406
MNDA	FCGR3A	0.862991999	MRPL42	UBE2N	0.856234266
MNDA	SPI1	0.895904928	ZNF14	ZNF253	0.889691942
MNDA	CD14	0.85190651	GON4L	GATAD2B	0.862997771
MNDA	LAPTM5	0.916831184	FNDC1	COL8A1	0.929871556
OLFML1	AEBP1	0.850921323	FNDC1	THBS2	0.852988171
KIF20B	KIF11	0.865653944	COL8A1	COL10A1	0.89694874
GAPVD1	GOLGA1	0.852419954	COL8A1	VCAN	0.892512717
GAPVD1	RC3H2	0.884492129	COL8A1	SFRP4	0.850636792
PODN	COL8A1	0.852960583	COL8A1	THBS2	0.946036387
PODN	THBS2	0.855195579	COL8A1	COL5A2	0.888423375
USP34	FBXO11	0.862404471	COL8A1	SERPINE1	0.871867639
ZNF500	MGRN1	0.851738652	COL8A1	COL6A3	0.850477919
FAM183A	C20orf85	0.877119701	COL8A1	THBS1	0.851913203
MTRNR2L8	MTRNR2L12	0.902198487	COL8A1	COL5A1	0.876104451
LAMA4	PDGFRB	0.856264831	COL8A1	MMP2	0.910313981
SKA1	ECT2	0.868840666	COL8A1	AEBP1	0.87106003
PIGG	GAK	0.85303362	COL8A1	FN1	0.891258508
PIGG	PCGF3	0.855125469	COL8A1	COL3A1	0.875719994
ISM1	COL5A2	0.889295904	COL8A1	COL1A2	0.889637604
ISM1	AEBP1	0.876822493	COL8A1	SPARC	0.862557984
STIL	DEPDC1	0.855791369	COL8A1	COL1A1	0.89469403

OLR1	HAVCR2	0.878093435	LMTK2	TRRAP	0.852560035
OLR1	ITGB2	0.852307813	INTS9	CCAR2	0.850343518
OLR1	ALOX5AP	0.870905503	KANSL1	GPATCH8	0.916283347
ARHGAP30	FERMT3	0.851352124	WAS	CD37	0.932487204
SLC7A7	MS4A6A	0.85247144	WAS	LPXN	0.866449312
SLC7A7	C3AR1	0.851443582	WAS	EVI2B	0.850328365
COL11A1	CRISPLD2	0.889674523	WAS	STAB1	0.853206934
COL11A1	C1QTNF6	0.865641314	WAS	FERMT3	0.925903822
COL11A1	COPZ2	0.851366371	WAS	SELPLG	0.896936423
COL11A1	COL8A1	0.882444086	WAS	C3AR1	0.873735544
COL11A1	VCAN	0.88685367	WAS	FCGR2A	0.871322439
COL11A1	LOX	0.867085572	WAS	LSP1	0.884185556
COL11A1	POSTN	0.905453259	WAS	CYBB	0.883214493
COL11A1	ECM1	0.862927277	WAS	ITGB2	0.865076102
COL11A1	THBS2	0.916930227	WAS	CSF1R	0.905233393
COL11A1	COL5A2	0.912159946	WAS	CD53	0.884094398
COL11A1	SFRP2	0.856889866	WAS	CD4	0.854876398
COL11A1	MMP11	0.891462816	WAS	AIF1	0.853338295
COL11A1	CTSK	0.879379903	WAS	SPI1	0.944600696
COL11A1	COL5A1	0.860873458	WAS	CD14	0.915113245
COL11A1	MMP2	0.853908564	WAS	LAPTM5	0.880378038
COL11A1	LUM	0.893246489	WAS	FCER1G	0.851633473
COL11A1	FN1	0.873855711	WAS	C1QA	0.868664418
COL11A1	COL3A1	0.902719874	WAS	C1QB	0.858047009
COL11A1	SPARC	0.870099913	WAS	C1QC	0.88963203
GASK1B	COL6A3	0.86707415	GMFG	LY86	0.883306786
ACTG2	ACTA2	0.862646117	GMFG	LY96	0.892591364
HELZ	MED13	0.893583861	GMFG	AIF1	0.919756367
HELZ	BPTF	0.892473381	GMFG	ARHGDIB	0.852235676
RNF8	TBC1D22B	0.875327274	GMFG	FCER1G	0.875119651
ADAP2	SLCO2B1	0.904621678	GMFG	C1QA	0.878611459
ADAP2	LRRC25	0.910584849	GMFG	TYROBP	0.897558437
ADAP2	MS4A7	0.896702443	CNST	AHCTF1	0.864186186
ADAP2	HAVCR2	0.899399419	HOXB5	HOXB8	0.860959657
ADAP2	GPR34	0.911100453	CD37	LPXN	0.858967353
ADAP2	SASH3	0.911921355	CD37	LY86	0.90056629
ADAP2	HCK	0.897005358	CD37	EVI2B	0.907982595
ADAP2	MSR1	0.858068484	CD37	FERMT3	0.902639027
ADAP2	ABI3	0.867489681	CD37	SELPLG	0.916689315
ADAP2	MPEG1	0.869546061	CD37	C3AR1	0.887355236
ADAP2	MS4A6A	0.884424814	CD37	FCGR2A	0.8779959
ADAP2	WAS	0.885393578	CD37	GIMAP4	0.892928061
ADAP2	LHFPL2	0.853975926	CD37	C1orf162	0.866263889
ADAP2	PLEK	0.901346626	CD37	LSP1	0.900515645
ADAP2	SH2B3	0.856560295	CD37	CYBB	0.871390279
ADAP2	CD163	0.88899885	CD37	RNASE6	0.872753674
ADAP2	STAB1	0.909186088	CD37	CSF1R	0.852708413
ADAP2	FPR3	0.859381539	CD37	CD53	0.909347253
ADAP2	FERMT3	0.909557905	CD37	AIF1	0.891920022
ADAP2	SELPLG	0.857087626	CD37	SPI1	0.951913631
ADAP2	C3AR1	0.92243704	CD37	CD14	0.88772484
ADAP2	FCGR2A	0.879474218	CD37	LAPTM5	0.881862222
ADAP2	CYBB	0.926653675	CD37	FCER1G	0.89062812
ADAP2	ITGB2	0.865719863	CD37	C1QA	0.896865456
ADAP2	RNASE6	0.886166457	CD37	TYROBP	0.860144903
ADAP2	CSF1R	0.945665272	CD37	C1QB	0.881314481

ADAP2	CD53	0.888095204	CD37	C1QC	0.888375315
ADAP2	CD4	0.874677549	SNAI2	COL6A3	0.879878566
ADAP2	HMOX1	0.852840071	SNAI2	COL3A1	0.873698877
ADAP2	AIF1	0.860173467	SNAI2	SPARC	0.853280202
ADAP2	VSIG4	0.869513768	LPXN	EVI2B	0.875096403
ADAP2	FCGR3A	0.875738554	LPXN	FERMT3	0.862535661
ADAP2	CD14	0.896886422	LPXN	SELPLG	0.855256899
ADAP2	LAPTM5	0.891301653	LPXN	CYBB	0.856738558
ADAP2	C1QC	0.874012279	LPXN	CD53	0.857171328
SLCO2B1	LRRC25	0.887584136	TTC30A	TTC30B	0.91550006
SLCO2B1	MS4A7	0.878813443	ZNF646	ZNF629	0.881461307
SLCO2B1	MS4A4A	0.877179185	ZNF646	SETD1A	0.876254475
SLCO2B1	GPR34	0.898636099	INTS12	UBE2D3	0.853300845
SLCO2B1	SASH3	0.875960708	PLEK	CD163	0.894020745
SLCO2B1	HCK	0.90723473	PLEK	EVI2B	0.915349094
SLCO2B1	MPEG1	0.914868894	PLEK	FPR3	0.866928755
SLCO2B1	MS4A6A	0.897355526	PLEK	FERMT3	0.903682838
SLCO2B1	PLEK	0.899509669	PLEK	SELPLG	0.859198597
SLCO2B1	SH2B3	0.876376262	PLEK	C3AR1	0.92355145
SLCO2B1	CD163	0.92563535	PLEK	FCGR2A	0.910144863
SLCO2B1	STAB1	0.869723381	PLEK	CYBB	0.948168469
SLCO2B1	FPR3	0.869410968	PLEK	ITGB2	0.904134017
SLCO2B1	FERMT3	0.869160964	PLEK	CSF1R	0.877669637
SLCO2B1	C3AR1	0.919382525	PLEK	CD53	0.934357228
SLCO2B1	FCGR2A	0.875805928	PLEK	VSIG4	0.875019281
SLCO2B1	CYBB	0.908665055	PLEK	FCGR3A	0.895771092
SLCO2B1	ITGB2	0.882338345	PLEK	CD14	0.858660212
SLCO2B1	CSF1R	0.944478294	PLEK	LAPTM5	0.913022085
SLCO2B1	CD53	0.85648671	PLEK	SRGN	0.853869075
SLCO2B1	CD4	0.873763845	PLEK	C1QB	0.87590292
SLCO2B1	VSIG4	0.886719453	PLEK	C1QC	0.885983698
SLCO2B1	FCGR3A	0.866861662	GAL3ST4	C3AR1	0.871180348
SLCO2B1	CD14	0.868675742	GAL3ST4	FCGR2A	0.867146991
SLCO2B1	LAPTM5	0.864171356	GAL3ST4	CSF1R	0.864580024
POLG2	CEP95	0.865821962	GAL3ST4	LAPTM5	0.868375451
LRRC25	MS4A7	0.88342513	ATE1	SEC23IP	0.874122064
LRRC25	HAVCR2	0.908809523	SEH1L	PTPN2	0.866641118
LRRC25	GPR34	0.878713722	COL10A1	SFRP4	0.856581166
LRRC25	SASH3	0.916271947	AP1M1	EPS15L1	0.887755589
LRRC25	HCK	0.930755327	SAMD9	IFIT2	0.86644438
LRRC25	ABI3	0.86579294	SAMD9	OAS2	0.873627379
LRRC25	MPEG1	0.885192244	SAMD9	OAS3	0.862630776
LRRC25	MS4A6A	0.888991082	SAMD9	MX1	0.871022535
LRRC25	WAS	0.893109799	SAMD9	IFIT3	0.88969012
LRRC25	CD37	0.873647668	GPR183	RGS1	0.898493867
LRRC25	PLEK	0.91385128	LAMP3	MX1	0.856704564
LRRC25	CD163	0.893902746	IL2RG	NKG7	0.902738851
LRRC25	EVI2B	0.875919364	IL2RG	CCL5	0.906901479
LRRC25	FPR3	0.854590624	FILIP1L	COL6A3	0.856694816
LRRC25	FERMT3	0.910914367	FILIP1L	TIMP2	0.899918434
LRRC25	C3AR1	0.926584275	UTP6	RHOT1	0.896652838
LRRC25	FCGR2A	0.893367474	NSD3	ASH2L	0.87621785
LRRC25	C1orf162	0.856157274	ATXN1L	DYNC1LI2	0.85649957
LRRC25	LSP1	0.87915524	ATXN1L	DHX38	0.895497155
LRRC25	CYBB	0.918206858	ATXN1L	AP1G1	0.878312931
LRRC25	ITGB2	0.886910721	ATXN1L	GLG1	0.890718546

LRRC25	RNASE6	0.871455317	FBXO42	MFN2	0.881730275
LRRC25	CSF1R	0.934505968	YLPM1	AREL1	0.870128575
LRRC25	CD53	0.914289003	PPAT	PAICS	0.865923721
LRRC25	CD4	0.902103404	SH2B3	DAB2	0.865580071
LRRC25	AIF1	0.90041825	SMAD4	MEX3C	0.859104968
LRRC25	VSIG4	0.87124518	CMPK2	RSAD2	0.941878153
LRRC25	FCGR3A	0.870368775	CMPK2	IFIH1	0.880738466
LRRC25	SPI1	0.89894667	CMPK2	IFI44L	0.905463567
LRRC25	CD14	0.91614012	CMPK2	DDX58	0.850355876
LRRC25	LAPTM5	0.900890707	CMPK2	OAS2	0.905069651
LRRC25	C1QC	0.857225493	CMPK2	OAS1	0.86120195
MS4A7	HAVCR2	0.886042811	CMPK2	IFIT1	0.917583836
MS4A7	MS4A4A	0.892498975	CMPK2	OAS3	0.878085442
MS4A7	GPR34	0.900461945	CMPK2	MX1	0.906582894
MS4A7	SASH3	0.883525893	CMPK2	IFI44	0.894904219
MS4A7	HCK	0.858882094	CMPK2	IFIT3	0.886765117
MS4A7	MSR1	0.876459622	CMPK2	STAT1	0.867186696
MS4A7	FGL2	0.866864797	CD163	STAB1	0.853968131
MS4A7	MPEG1	0.862833835	CD163	FPR3	0.853529127
MS4A7	MS4A6A	0.93109195	CD163	FERMT3	0.870114341
MS4A7	WAS	0.855859691	CD163	C3AR1	0.87946593
MS4A7	CD37	0.854358948	CD163	FCGR2A	0.903150714
MS4A7	PLEK	0.868612331	CD163	TNFRSF1B	0.867091551
MS4A7	CD163	0.887055541	CD163	CYBB	0.903058099
MS4A7	STAB1	0.850859785	CD163	CSF1R	0.883666504
MS4A7	FPR3	0.854395972	CD163	CD53	0.858313028
MS4A7	FERMT3	0.856939324	CD163	VSIG4	0.921505975
MS4A7	C3AR1	0.920431826	CD163	FCGR3A	0.879120433
MS4A7	FCGR2A	0.912898528	CD163	CD14	0.856918222
MS4A7	GIMAP4	0.866551214	CD163	LAPTM5	0.863531405
MS4A7	CYBB	0.887561028	CD163	C1QB	0.867908858
MS4A7	RNASE6	0.918908235	CD163	C1QC	0.881521025
MS4A7	CSF1R	0.907141664	ZNF526	ZNF574	0.854492647
MS4A7	CD53	0.895368044	ATXN2	BRAP	0.874459216
MS4A7	CD4	0.877697823	ATXN2	PTPN11	0.871485554
MS4A7	AIF1	0.891684298	HOXB3	HOXB4	0.886114039
MS4A7	VSIG4	0.867251039	LY86	GIMAP4	0.85185992
MS4A7	FCGR3A	0.910811764	LY86	C1orf162	0.906120257
MS4A7	CD14	0.899122151	LY86	LSP1	0.857553384
MS4A7	LAPTM5	0.882167709	LY86	RNASE6	0.899761194
MS4A7	SRGN	0.850914054	LY86	CD53	0.878010134
MS4A7	C1QA	0.867845775	LY86	TREM2	0.905721225
MS4A7	C1QB	0.897908161	LY86	AIF1	0.924364719
MS4A7	C1QC	0.90603223	LY86	SPI1	0.917635338
HAVCR2	GPR34	0.906640948	LY86	ARHGDIB	0.882792176
HAVCR2	SASH3	0.955236527	LY86	CD14	0.851929222
HAVCR2	HCK	0.916296546	LY86	HLA-DPB1	0.863226001
HAVCR2	CCR1	0.893555315	LY86	FCER1G	0.921854962
HAVCR2	MSR1	0.911344852	LY86	C1QA	0.883168572
HAVCR2	ABI3	0.90521208	LY86	TYROBP	0.94023457
HAVCR2	MPEG1	0.862359573	LY86	C1QB	0.854782361
HAVCR2	MS4A6A	0.918498013	LY86	C1QC	0.853570602
HAVCR2	WAS	0.904982054	VCAN	LOX	0.899353149
HAVCR2	CD37	0.915692824	VCAN	OLFML2B	0.865395731
HAVCR2	PLEK	0.927867451	VCAN	POSTN	0.877742659
HAVCR2	CD163	0.868317829	VCAN	THBS2	0.909199211

HAVCR2	LY86	0.876064674	VCAN	COL5A2	0.941864621
HAVCR2	EVI2B	0.954313632	VCAN	SFRP2	0.859558915
HAVCR2	FPR3	0.910406449	VCAN	COL6A3	0.882980438
HAVCR2	FERMT3	0.922384633	VCAN	CTSK	0.866413108
HAVCR2	SELPLG	0.904664054	VCAN	THBS1	0.851170174
HAVCR2	C3AR1	0.951423279	VCAN	COL5A1	0.902107506
HAVCR2	FCGR2A	0.940235736	VCAN	CTHRC1	0.854842153
HAVCR2	GIMAP4	0.903863406	VCAN	MMP2	0.912213466
HAVCR2	C1orf162	0.880806882	VCAN	LUM	0.87930312
HAVCR2	LSP1	0.902903188	VCAN	AEBP1	0.85014485
HAVCR2	CYBB	0.943154518	VCAN	FN1	0.922655645
HAVCR2	ITGB2	0.907841272	VCAN	COL3A1	0.915626569
HAVCR2	RNASE6	0.922071388	VCAN	SPARC	0.885954831
HAVCR2	CSF1R	0.902309853	VCAN	COL1A1	0.873388967
HAVCR2	CD53	0.969959236	EVI2B	FERMT3	0.859489666
HAVCR2	CD4	0.91591819	EVI2B	SELPLG	0.90697471
HAVCR2	TREM2	0.867510791	EVI2B	C3AR1	0.937857362
HAVCR2	ALOX5AP	0.900110773	EVI2B	FCGR2A	0.923707033
HAVCR2	AIF1	0.91289809	EVI2B	GIMAP4	0.891281533
HAVCR2	VSIG4	0.870215144	EVI2B	CYBB	0.939330883
HAVCR2	FCGR3A	0.912212843	EVI2B	ITGB2	0.888347259
HAVCR2	CTSS	0.861982555	EVI2B	RNASE6	0.886911523
HAVCR2	SPI1	0.917002014	EVI2B	CSF1R	0.870451494
HAVCR2	CD14	0.881222218	EVI2B	CD53	0.939404058
HAVCR2	LAPTM5	0.958331238	EVI2B	CD4	0.851661347
HAVCR2	SRGN	0.87455381	EVI2B	AIF1	0.858292292
HAVCR2	FCER1G	0.904728469	EVI2B	FCGR3A	0.915628722
HAVCR2	TYROBP	0.869426153	EVI2B	SPI1	0.852841043
HAVCR2	C1QB	0.873165789	EVI2B	LAPTM5	0.934504419
HAVCR2	C1QC	0.884028929	EVI2B	C1QB	0.867201993
MS4A4A	GPR34	0.860392614	EVI2B	C1QC	0.865107548
MS4A4A	HCK	0.855368264	DHRS4L2	DHRS4	0.872531615
MS4A4A	MPEG1	0.851039496	ZNF784	ZNF579	0.858468779
MS4A4A	MS4A6A	0.92406857	STAB1	CSF1R	0.927073113
MS4A4A	PLEK	0.879452785	FPR3	C3AR1	0.858769158
MS4A4A	CD163	0.9211243	FPR3	FCGR2A	0.867492817
MS4A4A	FPR3	0.857462957	FPR3	CD53	0.859232765
MS4A4A	C3AR1	0.891088762	FPR3	FCGR3A	0.87415723
MS4A4A	FCGR2A	0.881216838	FPR3	LAPTM5	0.872006236
MS4A4A	CYBB	0.864694082	CHD6	STK4	0.856499773
MS4A4A	RNASE6	0.864555376	CHD6	NCOA6	0.852440595
MS4A4A	CD53	0.877143593	RPTOR	UBE2O	0.857445839
MS4A4A	AIF1	0.853404486	RPTOR	NPLOC4	0.857674724
MS4A4A	VSIG4	0.910662451	NKG7	CCL5	0.908885596
MS4A4A	FCGR3A	0.874265568	SP100	IFIH1	0.868212592
MS4A4A	CD14	0.873447219	SP100	OAS2	0.850192775
MS4A4A	SRGN	0.870577673	GIGYF2	TRIP12	0.861346605
MS4A4A	C1QB	0.865891276	WASHC2C	WASHC2A	0.857283365
MS4A4A	C1QC	0.871233873	KIF1B	PRDM2	0.857029912
GPR34	SASH3	0.898140309	KIF1B	MTOR	0.851863176
GPR34	HCK	0.885525647	S100A8	S100A9	0.902634747
GPR34	MSR1	0.888582819	AGO2	PTK2	0.85573715
GPR34	MPEG1	0.857087096	POLB	VDAC3	0.850025944
GPR34	MS4A6A	0.947201954	HERC6	IFI44L	0.880745561
GPR34	PLEK	0.882682465	HERC6	OAS2	0.86126101
GPR34	CD163	0.87080491	HERC6	MX1	0.888211037

GPR34	EVI2B	0.867725142	HERC6	IFI44	0.882628255
GPR34	FPR3	0.858502817	HERC6	IFIT3	0.886303308
GPR34	FERMT3	0.857858372	FERMT3	SELPLG	0.920331225
GPR34	C3AR1	0.95126461	FERMT3	C3AR1	0.908899601
GPR34	FCGR2A	0.90007382	FERMT3	FCGR2A	0.89456798
GPR34	CYBB	0.911072257	FERMT3	LSP1	0.872843954
GPR34	ITGB2	0.864098873	FERMT3	CYBB	0.917708286
GPR34	RNASE6	0.913567876	FERMT3	ITGB2	0.923925233
GPR34	CSF1R	0.903652074	FERMT3	RNASE6	0.852044016
GPR34	CD53	0.905641641	FERMT3	CSF1R	0.902547805
GPR34	CD4	0.882353742	FERMT3	CD53	0.942203088
GPR34	TREM2	0.866388205	FERMT3	CD4	0.870129894
GPR34	AIF1	0.891799581	FERMT3	AIF1	0.864822079
GPR34	VSIG4	0.908655784	FERMT3	VSIG4	0.879125813
GPR34	FCGR3A	0.889435208	FERMT3	FCGR3A	0.859143728
GPR34	CD14	0.888908399	FERMT3	SPI1	0.923239042
GPR34	LAPTM5	0.889826543	FERMT3	CD14	0.919393349
GPR34	C1QB	0.855548159	FERMT3	LAPTM5	0.928478215
GPR34	C1QC	0.874206027	FERMT3	C1QA	0.893130924
SASH3	HCK	0.920900039	FERMT3	C1QB	0.904684815
SASH3	CCR1	0.854883872	FERMT3	C1QC	0.932307078
SASH3	MSR1	0.857128121	RSAD2	IFIH1	0.893104957
SASH3	ABI3	0.933724161	RSAD2	IFI44L	0.905417832
SASH3	FGL2	0.859537736	RSAD2	IFIT2	0.873532495
SASH3	MPEG1	0.882666124	RSAD2	DDX58	0.868318264
SASH3	MS4A6A	0.926736056	RSAD2	PARP14	0.864663513
SASH3	WAS	0.934762747	RSAD2	PARP9	0.854581041
SASH3	CD37	0.930429787	RSAD2	OAS2	0.922945925
SASH3	LPXN	0.850921449	RSAD2	OAS1	0.860880051
SASH3	PLEK	0.934450183	RSAD2	IFIT1	0.905520946
SASH3	CD163	0.879489942	RSAD2	OAS3	0.8727785
SASH3	EVI2B	0.920973569	RSAD2	MX1	0.897072753
SASH3	FPR3	0.870417367	RSAD2	IFI44	0.882190293
SASH3	FERMT3	0.961662596	RSAD2	IFIT3	0.906604696
SASH3	SELPLG	0.923360866	RSAD2	STAT1	0.866815882
SASH3	C3AR1	0.945324687	MT1H	MT1G	0.918256017
SASH3	FCGR2A	0.897986565	OSGEP	METTL17	0.874375896
SASH3	GIMAP4	0.896089913	WDR53	SENP5	0.854249556
SASH3	C1orf162	0.859201596	POLR3F	CRNKL1	0.874607847
SASH3	LSP1	0.903040896	ANO8	SIN3B	0.854343449
SASH3	CYBB	0.938992251	EDC4	DHX38	0.863506501
SASH3	ITGB2	0.907993303	SELPLG	C3AR1	0.919395916
SASH3	RNASE6	0.921144824	SELPLG	FCGR2A	0.897418112
SASH3	CSF1R	0.942366637	SELPLG	GIMAP4	0.859751366
SASH3	CD53	0.963152151	SELPLG	LSP1	0.853918523
SASH3	CD4	0.924688187	SELPLG	CYBB	0.923070343
SASH3	TREM2	0.850173749	SELPLG	ITGB2	0.886859107
SASH3	ALOX5AP	0.881165702	SELPLG	RNASE6	0.86975758
SASH3	AIF1	0.908143503	SELPLG	CSF1R	0.898973406
SASH3	VSIG4	0.864539352	SELPLG	CD53	0.926738751
SASH3	FCGR3A	0.885964558	SELPLG	CD4	0.903509666
SASH3	SPI1	0.937302673	SELPLG	ALOX5AP	0.867501896
SASH3	CD14	0.903823953	SELPLG	AIF1	0.868074677
SASH3	LAPTM5	0.949629727	SELPLG	FCGR3A	0.864755763
SASH3	FCER1G	0.875817184	SELPLG	SPI1	0.898629405
SASH3	C1QA	0.861977718	SELPLG	CD14	0.886548775

SASH3	C1QB	0.887289402	SELPLG	LAPTM5	0.907725828
SASH3	C1QC	0.902374341	SELPLG	C1QA	0.862953389
DNAJC16	MTOR	0.865505362	SELPLG	C1QB	0.879804543
LTN1	USP16	0.861307275	SELPLG	C1QC	0.888104559
TNFSF13B	GIMAP4	0.858951581	LOX	THBS2	0.852003748
TLK2	MED13	0.883518525	LOX	COL5A2	0.863780971
TLK2	DCAF7	0.860313346	LOX	COL5A1	0.856480167
CRISPLD2	COL8A1	0.863324534	LOX	MMP2	0.852313233
CRISPLD2	VCAN	0.881177656	LOX	FN1	0.868128646
CRISPLD2	THBS2	0.872131732	LOX	COL3A1	0.873283047
CRISPLD2	COL5A2	0.896441457	HLA-F	PSMB9	0.878611692
CRISPLD2	COL5A1	0.871796511	HLA-F	TAP1	0.868763626
CRISPLD2	MMP2	0.8568968	HLA-F	HLA-A	0.881836205
CRISPLD2	LUM	0.865358954	HLA-F	HLA-B	0.914338058
CRISPLD2	AEBP1	0.853892144	C3AR1	FCGR2A	0.964883248
CRISPLD2	COL3A1	0.878233361	C3AR1	GIMAP4	0.87622777
CRISPLD2	SPARC	0.872750411	C3AR1	CYBB	0.960897476
CRISPLD2	COL1A1	0.85651603	C3AR1	ITGB2	0.914109103
DEPDC1	DLGAP5	0.871699273	C3AR1	RNASE6	0.907949016
DEPDC1	KIF2C	0.862463476	C3AR1	CSF1R	0.934615112
URB2	NUP133	0.855550696	C3AR1	CD53	0.957075411
URB2	PARP1	0.86101643	C3AR1	CD4	0.872993344
FADS1	FADS2	0.865974128	C3AR1	ALOX5AP	0.890523727
ZNF786	ZNF212	0.899208498	C3AR1	AIF1	0.870836009
DDX19A	SF3B3	0.854581838	C3AR1	VSIG4	0.899531392
TUBGCP6	PPP6R2	0.873469371	C3AR1	FCGR3A	0.930151953
ARHGAP11A	BUB1B	0.881168654	C3AR1	SPI1	0.878182637
SAMD9L	SAMD9	0.86752924	C3AR1	CD14	0.917248371
PRSS1	PRSS2	0.869143954	C3AR1	LAPTM5	0.963244507
DDX60	HERC6	0.856891634	C3AR1	C1QA	0.863878673
DDX60	RSAD2	0.861343986	C3AR1	C1QB	0.902802003
DDX60	OAS2	0.883857162	C3AR1	C1QC	0.920018822
HCK	MPEG1	0.932299807	POLR1A	UBR4	0.87841266
HCK	MS4A6A	0.91823215	PDPN	SPARC	0.855484366
HCK	WAS	0.878297984	SENP5	PAK2	0.874711317
HCK	CD37	0.868046199	KPNA1	TOPBP1	0.850890881
HCK	PLEK	0.950133485	ZNF791	TNPO2	0.857308124
HCK	SH2B3	0.877080491	GRIPAP1	HDAC6	0.870570465
HCK	CD163	0.902969877	GRIPAP1	OTUD5	0.885908904
HCK	EVI2B	0.905692938	DNAJC13	CDV3	0.871497239
HCK	FPR3	0.90949175	UBR4	MTOR	0.871200923
HCK	FERMT3	0.928720835	UBR4	DNAJC11	0.852776852
HCK	SELPLG	0.875247615	NCAPH	BUB1	0.919434227
HCK	C3AR1	0.947434417	NCAPH	TOP2A	0.884491111
HCK	FCGR2A	0.918246569	DHX38	GLG1	0.850422476
HCK	LSP1	0.869342933	GTPBP3	AKAP8	0.881370599
HCK	CYBB	0.954957164	FCGR2A	GIMAP4	0.8719835
HCK	ITGB2	0.935674436	FCGR2A	CYBB	0.945145145
HCK	RNASE6	0.876385241	FCGR2A	ITGB2	0.89807004
HCK	CSF1R	0.938156703	FCGR2A	RNASE6	0.882744328
HCK	CD53	0.932844677	FCGR2A	CSF1R	0.920856824
HCK	CD4	0.915802723	FCGR2A	CD53	0.942982764
HCK	ALOX5AP	0.851993071	FCGR2A	CD4	0.884414294
HCK	AIF1	0.900460449	FCGR2A	VSIG4	0.925725592
HCK	VSIG4	0.870052838	FCGR2A	FCGR3A	0.94625244
HCK	FCGR3A	0.86413915	FCGR2A	SPI1	0.861570998

HCK	SPI1	0.900165351	FCGR2A	CD14	0.92138509
HCK	LYZ	0.868831411	FCGR2A	LAPTM5	0.95927705
HCK	CD14	0.903421944	FCGR2A	SRGN	0.859809042
HCK	LAPTM5	0.923328678	FCGR2A	C1QA	0.867409851
CCR1	MSR1	0.856298407	FCGR2A	C1QB	0.913093275
CCR1	PLEK	0.886958816	FCGR2A	C1QC	0.921787583
CCR1	EVI2B	0.866960456	TMEM158	FN1	0.866798184
CCR1	C3AR1	0.885156365	GIMAP4	C1orf162	0.883296499
CCR1	FCGR2A	0.861955256	GIMAP4	CYBB	0.868793856
CCR1	GIMAP4	0.853566104	GIMAP4	RNASE6	0.857899196
CCR1	CYBB	0.860294442	GIMAP4	CD53	0.896643712
CCR1	ITGB2	0.851483008	GIMAP4	AIF1	0.864427905
CCR1	RNASE6	0.86881537	GIMAP4	FCGR3A	0.884527079
CCR1	CD53	0.891388945	GIMAP4	SPI1	0.868419737
CCR1	FCGR3A	0.860760744	GIMAP4	C1QA	0.88830646
CCR1	LAPTM5	0.880138314	GIMAP4	C1QB	0.876907011
MSR1	MS4A6A	0.861207593	GIMAP4	C1QC	0.873471817
MSR1	PLEK	0.867839661	CHMP7	CCAR2	0.850020539
MSR1	FPR3	0.873356036	NDUFA7	PET100	0.862630248
MSR1	C3AR1	0.913760082	OLFML2B	POSTN	0.856446474
MSR1	FCGR2A	0.894069055	OLFML2B	COL5A2	0.864102833
MSR1	CYBB	0.860072127	OLFML2B	FN1	0.852363703
MSR1	RNASE6	0.888738535	RBM5	RBM6	0.850240523
MSR1	CD53	0.881980043	ZNF142	ATG9A	0.879534411
MSR1	TREM2	0.85448402	BTN3A3	BTN3A1	0.851251936
MSR1	FCGR3A	0.934307797	POSTN	THBS2	0.89901816
MSR1	LAPTM5	0.889220883	POSTN	COL5A2	0.918722468
MSR1	C1QC	0.872924232	POSTN	SFRP2	0.89939271
ABI3	LST1	0.870937063	POSTN	COL6A3	0.85347243
ABI3	MS4A6A	0.878506565	POSTN	MMP11	0.863733689
ABI3	WAS	0.909145807	POSTN	CTSK	0.869635193
ABI3	CD37	0.93629524	POSTN	COL5A1	0.858491937
ABI3	LY86	0.867184823	POSTN	MMP2	0.864929957
ABI3	EVI2B	0.862195214	POSTN	FN1	0.871714755
ABI3	FERMT3	0.902567017	POSTN	COL3A1	0.890107871
ABI3	SELPLG	0.90121461	POSTN	SPARC	0.850607968
ABI3	C3AR1	0.866116478	C1orf162	AIF1	0.881094537
ABI3	GIMAP4	0.900840758	C1orf162	SPI1	0.889843687
ABI3	C1orf162	0.870877841	C1orf162	FCER1G	0.883775574
ABI3	LSP1	0.865677925	C1orf162	TYROBP	0.888248923
ABI3	CYBB	0.862976968	AP1G1	IST1	0.858455814
ABI3	RNASE6	0.881625147	POM121	GTF2I	0.878342773
ABI3	CSF1R	0.858637621	POM121	POM121C	0.913870248
ABI3	CD53	0.892957881	ECM1	THBS2	0.866184143
ABI3	TREM2	0.859939539	ECM1	COL5A2	0.881112841
ABI3	AIF1	0.893610384	ECM1	COL6A3	0.872439799
ABI3	SPI1	0.920515388	ECM1	COL5A1	0.8983177
ABI3	CD14	0.854520141	ECM1	MMP2	0.885429801
ABI3	LAPTM5	0.862619506	ECM1	AEBP1	0.868958684
ABI3	FCER1G	0.883866051	ECM1	FN1	0.852247051
ABI3	C1QA	0.867473815	ECM1	COL6A2	0.892390792
ABI3	TYROBP	0.863414147	ECM1	COL3A1	0.863908257
ABI3	C1QB	0.872941899	ECM1	COL1A1	0.873459867
ABI3	C1QC	0.868138746	CCDC90B	TMEM126B	0.872443103
DHX58	IFI35	0.860586982	KHNYN	RNF31	0.852674858
PCNT	MCM3AP	0.881168834	PPP1R37	CLPTM1	0.871285378

ZNF106	VPS39	0.862939034	TIGD5	HSF1	0.86096236
LST1	GMFG	0.923763561	BUB1	RACGAP1	0.850946862
LST1	CD37	0.894440396	BUB1	TOP2A	0.856711368
LST1	LY86	0.887507738	BUB1	TPX2	0.855620525
LST1	C1orf162	0.856136413	UQCRHL	UQCRH	0.921474371
LST1	CD53	0.857606747	BAG4	ASH2L	0.857101851
LST1	AIF1	0.92419402	BAG4	LSM1	0.85619577
LST1	SPI1	0.912283818	LOXL2	COL6A3	0.853924342
LST1	FCER1G	0.896869182	LOXL2	COL5A1	0.850295795
LST1	TYROBP	0.904218897	LAMB1	NID1	0.855688005
H3C4	H2AC7	0.955661257	NID1	PDGFRB	0.88576315
FGL2	MPEG1	0.867322703	ARFGEF2	RALGAPB	0.859329447
FGL2	MS4A6A	0.855785829	ARFGEF2	STAU1	0.850053106
FGL2	EVI2B	0.878072428	PIK3R4	GSK3B	0.851064964
FGL2	C3AR1	0.856963221	IFIH1	IFI44L	0.882352693
FGL2	GIMAP4	0.869990788	IFIH1	IFIT2	0.869029527
FGL2	CYBB	0.854107162	IFIH1	DDX58	0.85399382
FGL2	RNASE6	0.865159644	IFIH1	OAS2	0.910784266
FGL2	CD53	0.85312868	IFIH1	OAS1	0.89529887
FGL2	FCGR3A	0.85489314	IFIH1	IFIT1	0.868184018
FGL2	LAPTM5	0.853467079	IFIH1	OAS3	0.869120923
CXCL11	CXCL10	0.930292945	IFIH1	MX1	0.886003928
ADGRA2	LHFPL6	0.853003091	IFIH1	IFI44	0.863354349
ADGRA2	NID1	0.902194438	IFIH1	IFIT3	0.892269153
ADGRA2	PDGFRB	0.887779081	TAOK2	SETD1A	0.85764568
ADGRA2	EMILIN1	0.91785676	GPANK1	NELFE	0.864800444
ELF2	SMARCA5	0.855121536	STK36	ANKZF1	0.867604468
SIRT7	DUS1L	0.85815401	EPS15L1	SIN3B	0.869818651
FBXO45	RNF168	0.866059062	EPS15L1	WIZ	0.868234554
FBXO45	SENP5	0.893295199	EPS15L1	CHERP	0.899070295
FBXO45	LSG1	0.891493633	EPS15L1	BRD4	0.87293299
FBXO45	PAK2	0.88135794	POLG	ZNF592	0.869689187
C1orf216	AGO1	0.851958721	CREB3L1	COL5A2	0.8566023
OASL	CMPK2	0.895038927	CREB3L1	COL6A3	0.922169497
OASL	RSAD2	0.912887257	CREB3L1	EMILIN1	0.850503626
OASL	IFI44L	0.889752893	CREB3L1	COL5A1	0.918466598
OASL	OAS2	0.899441338	CREB3L1	MMP2	0.881855849
OASL	OAS1	0.920768098	CREB3L1	AEBP1	0.888986018
OASL	IFI35	0.857671125	CREB3L1	COL6A2	0.897759366
OASL	IFI44	0.863063007	CREB3L1	COL6A1	0.879848853
OASL	IFIT3	0.868769737	CREB3L1	COL3A1	0.872453816
OASL	ISG15	0.85262041	CREB3L1	COL1A2	0.892527797
HERC5	CMPK2	0.878362844	CREB3L1	SPARC	0.879639948
HERC5	HERC6	0.860120084	CREB3L1	COL1A1	0.909385935
HERC5	RSAD2	0.868619495	MRPS23	SUPT4H1	0.863058078
COPZ2	LUM	0.851535241	IFIT5	IFIT1	0.870707479
COPZ2	COL3A1	0.850250424	IFIT5	IFIT3	0.865684536
ZBTB6	RAB14	0.862095859	LSP1	ITGB2	0.859897374
MPEG1	MS4A6A	0.901688424	LSP1	CD53	0.854716594
MPEG1	PLEK	0.936271351	LSP1	ALOX5AP	0.861629871
MPEG1	SH2B3	0.881066061	LSP1	SPI1	0.927232503
MPEG1	CD163	0.894622848	LSP1	CD14	0.866693481
MPEG1	EVI2B	0.888610975	LSP1	LAPTM5	0.857135867
MPEG1	FPR3	0.916382347	LSP1	FCER1G	0.870746822
MPEG1	FERMT3	0.876855217	TBC1D25	OTUD5	0.864494511
MPEG1	C3AR1	0.912524755	ITGA5	MMP14	0.863621085

MPEG1	FCGR2A	0.883017131	GCDH	FARSA	0.866764776
MPEG1	CYBB	0.940179692	GTF2H3	DENR	0.854584926
MPEG1	ITGB2	0.902638785	ANGPTL2	PDGFRB	0.864618945
MPEG1	RNASE6	0.854709846	ANGPTL2	ANTXR1	0.8533344
MPEG1	CSF1R	0.932026536	IFI44L	IFIT2	0.85190206
MPEG1	CD53	0.897529343	IFI44L	OAS2	0.901705862
MPEG1	CD4	0.907971156	IFI44L	OAS1	0.880977797
MPEG1	FCGR3A	0.859953491	IFI44L	IFIT1	0.917974702
MPEG1	CD14	0.861833889	IFI44L	OAS3	0.871603004
MPEG1	LAPTM5	0.905748126	IFI44L	MX1	0.918899904
SNIP1	C1orf109	0.904815056	IFI44L	IFI44	0.943041401
SNIP1	GNL2	0.860686656	IFI44L	IFIT3	0.923861941
SNIP1	MEAF6	0.87296647	IFI44L	PLSCR1	0.864073135
HOXB6	HOXB5	0.920323583	IFIT2	PARP14	0.850028979
HOXB6	HOXB8	0.861866086	IFIT2	OAS2	0.896430272
EHMT1	BRD3	0.85072187	IFIT2	IFIT1	0.876986052
RAD54L	KIF2C	0.873545575	IFIT2	OAS3	0.860720126
RAP1B	TBC1D15	0.858118779	IFIT2	MX1	0.853808565
RO60	CDC73	0.852047206	IFIT2	IFIT3	0.933337142
MS4A6A	WAS	0.859512638	YEATS2	EIF4G1	0.874008696
MS4A6A	CD37	0.880551893	GTF2I	POM121C	0.862121897
MS4A6A	PLEK	0.914848088	RALGAPB	RPRD1B	0.850791535
MS4A6A	CD163	0.895861928	RALGAPB	NCOA6	0.882984558
MS4A6A	EVI2B	0.902805645	SAFB2	SAFB	0.902805892
MS4A6A	FPR3	0.88885915	MLXIP	NCOR2	0.856007367
MS4A6A	FERMT3	0.883305698	POP4	UQCRFS1	0.908813666
MS4A6A	SELPLG	0.875062691	MSH6	MSH2	0.874281436
MS4A6A	C3AR1	0.961764974	DNAJC11	MFN2	0.868396586
MS4A6A	FCGR2A	0.925486542	PDGFRB	COL5A2	0.879652563
MS4A6A	GIMAP4	0.891680968	PDGFRB	COL6A3	0.902728134
MS4A6A	CYBB	0.933002968	PDGFRB	EMILIN1	0.867871902
MS4A6A	ITGB2	0.87736879	PDGFRB	COL5A1	0.860327533
MS4A6A	RNASE6	0.944331371	PDGFRB	MMP2	0.851963824
MS4A6A	CSF1R	0.920021786	PDGFRB	AEBP1	0.876469259
MS4A6A	CD53	0.947234568	PDGFRB	COL3A1	0.861731579
MS4A6A	CD4	0.907026978	SENP2	LSG1	0.854889033
MS4A6A	AIF1	0.940102817	SDHAF2	SELENOH	0.860073034
MS4A6A	VSIG4	0.915753445	TONSL	RECQL4	0.866180276
MS4A6A	FCGR3A	0.9117789	TONSL	CPSF1	0.851306004
MS4A6A	SPI1	0.88062513	CD248	PCOLCE	0.851977745
MS4A6A	CD14	0.928039448	SERTAD1	SERTAD3	0.867687942
MS4A6A	LAPTM5	0.922500045	FAM222B	SUPT6H	0.893920205
MS4A6A	SRGN	0.868832629	KRAS	ETFRF1	0.863442121
MS4A6A	FCER1G	0.864472174	CYBB	ITGB2	0.914456677
MS4A6A	C1QA	0.880350967	CYBB	RNASE6	0.877519384
MS4A6A	C1QB	0.912431173	CYBB	CSF1R	0.941932651
MS4A6A	C1QC	0.918500354	CYBB	CD53	0.945929991
CD93	DAB2	0.850315073	CYBB	CD4	0.894691058
CDH11	SNAI2	0.875820508	CYBB	VSIG4	0.880026794
CDH11	VCAN	0.888007261	CYBB	FCGR3A	0.926662102
CDH11	COL5A2	0.867459911	CYBB	SPI1	0.851652108
CDH11	COL6A3	0.895388133	CYBB	CD14	0.893077194
CDH11	DCN	0.882917747	CYBB	LAPTM5	0.947538413
CDH11	COL5A1	0.890870922	CYBB	C1QB	0.88183889
CDH11	AEBP1	0.869135102	CYBB	C1QC	0.899627727
CDH11	COL3A1	0.875075214	PDCD11	PPRC1	0.881495099

CDH11	COL1A2	0.871286547	CCDC120	OTUD5	0.864695113
CDH11	SPARC	0.868136147	TMEM33	SLC30A9	0.872117805
CDH11	COL1A1	0.87705933	PDS5A	UBE2K	0.853342373
FANCI	PRC1	0.865390591	AKAP8	SIN3B	0.893078952
MRPL42	UBE2N	0.861105391	AKAP8	AKAP8L	0.882738184
HEATR6	MED13	0.851146326	AKAP8	WIZ	0.907655234
FNDC1	COL8A1	0.853898596	AKAP8	BRD4	0.891799596
FNDC1	COL1A1	0.851383394	ITGB2	CSF1R	0.915346989
COL8A1	COL10A1	0.91681087	ITGB2	CD53	0.918286535
COL8A1	THBS2	0.924745108	ITGB2	ALOX5AP	0.852288
COL8A1	DCN	0.852228847	ITGB2	SPI1	0.865212718
COL8A1	ACTA2	0.854679342	ITGB2	CD14	0.895795524
COL8A1	AEBP1	0.869107229	ITGB2	LAPTM5	0.932524459
COL8A1	FN1	0.882398381	ITGB2	C1QB	0.85167902
COL8A1	COL3A1	0.866959444	ITGB2	C1QC	0.874351948
COL8A1	COL1A1	0.861371745	SCAF4	SON	0.908366013
FBXO11	MCFD2	0.853159817	MAEA	CTBP1	0.872482554
LMTK2	TRRAP	0.884333055	XPO5	PPP2R5D	0.856227071
KANSL1	GPATCH8	0.882074438	TRIM39-RPP21	RPP21	1
RANBP10	EDC4	0.882652294	NDUFA11	MICOS13	0.856229352
WAS	CD37	0.934204768	TM2D2	LSM1	0.875030494
WAS	LPXN	0.850549396	HOOK2	TNPO2	0.853368107
WAS	PLEK	0.877585524	ATAD3A	NOC2L	0.860099356
WAS	EVI2B	0.893847936	RRN3	PARN	0.855933291
WAS	FERMT3	0.925000545	RRN3	PDXDC1	0.869337419
WAS	SELPLG	0.916193246	RRN3	GSPT1	0.868508716
WAS	C3AR1	0.880747208	DDX58	OAS2	0.856714128
WAS	FCGR2A	0.861890208	PNMA8A	CCDC8	0.855532353
WAS	GIMAP4	0.868532863	UQCC2	SNRPC	0.873228717
WAS	C1orf162	0.882056574	NR4A1	FOSB	0.88648638
WAS	LSP1	0.901000129	CCDC22	GPKOW	0.884881386
WAS	CYBB	0.898769571	FOXK2	NPLOC4	0.862569277
WAS	ITGB2	0.870243463	TCEAL3	TCEAL4	0.869862184
WAS	RNASE6	0.871922695	RNASE6	CD53	0.933384876
WAS	CSF1R	0.905896229	RNASE6	TREM2	0.850006653
WAS	CD53	0.905772529	RNASE6	ALOX5AP	0.892424496
WAS	CD4	0.923634111	RNASE6	AIF1	0.922109984
WAS	AIF1	0.877425059	RNASE6	FCGR3A	0.858536277
WAS	SPI1	0.92691091	RNASE6	SPI1	0.894032423
WAS	LAPTM5	0.889670854	RNASE6	CD14	0.865560903
WAS	FCER1G	0.858751437	RNASE6	LAPTM5	0.900465862
GMFG	LY86	0.880223585	RNASE6	SRGN	0.850201137
GMFG	LY96	0.857587288	RNASE6	FCER1G	0.896062013
GMFG	AIF1	0.893615932	RNASE6	C1QA	0.900159948
GMFG	FCER1G	0.876628825	RNASE6	TYROBP	0.890014974
GMFG	TYROBP	0.902504695	RNASE6	C1QB	0.900981906
CAMSAP1	BRD3	0.853421252	RNASE6	C1QC	0.905902218
PROSER3	KMT2B	0.870457452	KRI1	TYK2	0.857561106
CD37	LY86	0.88215073	KRI1	KEAP1	0.850821758
CD37	EVI2B	0.914894841	KRI1	ILF3	0.877201916
CD37	FERMT3	0.912984001	KRI1	CDC37	0.860826165
CD37	SELPLG	0.907771951	HLA-DQA1	HLA-DQB1	0.893642791
CD37	C3AR1	0.891999222	HLA-DQA1	HLA-DPA1	0.892989687
CD37	FCGR2A	0.870206972	HLA-DQA1	HLA-DPB1	0.852554653
CD37	GIMAP4	0.915169729	HLA-DQA1	HLA-DRB1	0.886887405
CD37	C1orf162	0.893404527	HLA-DQA1	HLA-DRA	0.881598483

CD37	LSP1	0.905989743	CHD8	TOX4	0.881260683
CD37	CYBB	0.880406161	CHD8	SUPT16H	0.914123575
CD37	ITGB2	0.856163846	KIF2C	CDCA8	0.851038342
CD37	RNASE6	0.887505689	KIF2C	CDC20	0.859229593
CD37	CSF1R	0.875792226	SMIM7	BABAM1	0.861658968
CD37	CD53	0.913930595	CYHR1	HGH1	0.871941593
CD37	CD4	0.888601427	CYHR1	HSF1	0.883948323
CD37	AIF1	0.913005438	TBC1D20	STK35	0.893319274
CD37	SPI1	0.951782274	THBS2	COL5A2	0.941033399
CD37	CD14	0.858152594	THBS2	SERPINE1	0.870160618
CD37	LAPTM5	0.88162379	THBS2	COL6A3	0.900267902
CD37	FCER1G	0.893720641	THBS2	CTSK	0.853136719
CD37	C1QA	0.856128248	THBS2	THBS1	0.86954053
CD37	TYROBP	0.881975009	THBS2	COL5A1	0.929029753
CD37	C1QB	0.857839236	THBS2	ACTA2	0.883913441
SNAI2	VCAN	0.897000622	THBS2	MMP2	0.94211993
SNAI2	LHFPL6	0.873511947	THBS2	LUM	0.855351192
SNAI2	PDGFRB	0.891604305	THBS2	AEBP1	0.884040821
SNAI2	COL5A2	0.918727	THBS2	FN1	0.936048732
SNAI2	COL6A3	0.859584495	THBS2	COL3A1	0.926903596
SNAI2	COL5A1	0.894526315	THBS2	COL1A2	0.895680752
SNAI2	LUM	0.856045894	THBS2	SPARC	0.890221924
SNAI2	COL3A1	0.874065208	THBS2	COL1A1	0.920586372
SNAI2	COL1A2	0.853803064	CSF1R	CD53	0.888694977
SNAI2	SPARC	0.890662949	CSF1R	CD4	0.899389638
TTC30A	TTC30B	0.911062615	CSF1R	ALOX5AP	0.860064318
ZNF646	ZNF629	0.887758126	CSF1R	VSIG4	0.882995742
PLEK	SH2B3	0.874697277	CSF1R	SPI1	0.8623144
PLEK	CD163	0.928799296	CSF1R	CD14	0.92271545
PLEK	EVI2B	0.909074645	CSF1R	LAPTM5	0.925238337
PLEK	FPR3	0.927494994	CSF1R	C1QC	0.879291593
PLEK	FERMT3	0.926546465	PARP14	PARP9	0.866143929
PLEK	SELPLG	0.863632687	PARP14	OAS2	0.896223203
PLEK	C3AR1	0.948488161	PARP14	OAS3	0.862680889
PLEK	FCGR2A	0.922816346	PARP14	MX1	0.876468838
PLEK	LSP1	0.857343052	PARP14	IFIT3	0.861672933
PLEK	CYBB	0.962438156	CD53	CD4	0.866764971
PLEK	ITGB2	0.927334053	CD53	ALOX5AP	0.875517699
PLEK	RNASE6	0.890783068	CD53	AIF1	0.927839534
PLEK	CSF1R	0.928576692	CD53	VSIG4	0.902131355
PLEK	CD53	0.960183245	CD53	FCGR3A	0.924643996
PLEK	CD4	0.911922131	CD53	SPI1	0.914957975
PLEK	AIF1	0.896554671	CD53	CD14	0.921362911
PLEK	VSIG4	0.891531484	CD53	LAPTM5	0.968377523
PLEK	FCGR3A	0.904477953	CD53	SRGN	0.905878024
PLEK	SPI1	0.875010613	CD53	FCER1G	0.886711083
PLEK	CD14	0.910798232	CD53	C1QA	0.934603976
PLEK	LAPTM5	0.955370257	CD53	TYROBP	0.85941962
PLEK	SRGN	0.87818802	CD53	C1QB	0.951202349
PLEK	C1QB	0.862308574	CD53	C1QC	0.957063164
PLEK	C1QC	0.877088492	PARP9	OAS2	0.868969821
HTT	FAM193A	0.89362687	PARP9	IFIT1	0.851328372
USP38	SMARCA5	0.896094104	PARP9	DTX3L	0.926406954
COL10A1	ECM1	0.857383013	PARP9	STAT1	0.860686413
COL10A1	THBS2	0.939313478	TMEM176A	TMEM176B	0.948996417
COL10A1	MMP11	0.866129409	FOSB	FOS	0.892411868

COL10A1	DCN	0.858667218	FOSB	EGR1	0.884522204
COL10A1	COL1A1	0.851251788	FOSB	DUSP1	0.850439828
GPR183	C5AR1	0.862777998	PARN	PDXDC1	0.876877737
GPR183	RGS1	0.876869114	INTS13	MRPS35	0.852659726
IL2RG	EVI2B	0.852602189	PNPLA6	MAP2K7	0.866589075
IL2RG	NKG7	0.878713768	REEP3	NRBF2	0.870640394
IL2RG	CCL5	0.925036354	LAMTOR3	UBE2D3	0.855444941
SPTY2D1	GTF2H1	0.866737109	WIPI2	EIF2AK1	0.885386039
FILIP1L	COL6A3	0.863453135	SIN3B	AKAP8L	0.862316941
FILIP1L	COL5A1	0.887103875	SIN3B	MYO9B	0.912298303
FILIP1L	COL1A2	0.885871994	SIN3B	WIZ	0.893765227
FILIP1L	COL1A1	0.889995874	SIN3B	SLC35E1	0.86155313
ATXN1L	DHX38	0.882538916	SIN3B	CHERP	0.877348263
ATXN1L	AP1G1	0.8521342	SIN3B	BRD4	0.882815272
RRAGC	AKIRIN1	0.853438455	ATG4B	STK25	0.903312177
MED1	DHX8	0.858237216	NBL1	MICOS10-NBL1	1
SH2B3	CYBB	0.882150572	COL5A2	SFRP2	0.867463327
SH2B3	ITGB2	0.87621774	COL5A2	COL6A3	0.954707642
SH2B3	CSF1R	0.898301342	COL5A2	MMP11	0.86169841
CMPK2	HERC6	0.852276176	COL5A2	ANTXR1	0.859743761
CMPK2	RSAD2	0.949547677	COL5A2	CTSK	0.888071714
CMPK2	IFIH1	0.867059116	COL5A2	COL5A1	0.955488572
CMPK2	IFI44L	0.886124896	COL5A2	CTHRC1	0.871704509
CMPK2	OAS2	0.918265441	COL5A2	MMP2	0.941225242
CMPK2	OAS1	0.886825024	COL5A2	LUM	0.873624414
CMPK2	MX1	0.883253991	COL5A2	AEBP1	0.902358031
CMPK2	IFI44	0.869659256	COL5A2	FN1	0.912415269
RLF	FOXJ3	0.870302422	COL5A2	COL3A1	0.966903747
RLF	PPT1	0.850453761	COL5A2	COL1A2	0.888186021
TBC1D15	CCT2	0.856291866	COL5A2	SPARC	0.927020635
CD163	STAB1	0.872008748	COL5A2	COL1A1	0.928645198
CD163	FPR3	0.894661086	MAP2K7	XAB2	0.884949799
CD163	FERMT3	0.871862212	PTPN11	ATP2A2	0.889998312
CD163	C3AR1	0.925515886	CD4	CD14	0.864242546
CD163	FCGR2A	0.903650827	CD4	LAPTM5	0.896881488
CD163	CYBB	0.926026029	CD4	C1QC	0.853262112
CD163	ITGB2	0.869289384	SETD1A	FBRS	0.86484418
CD163	RNASE6	0.870124293	STXBP2	XAB2	0.854911789
CD163	CSF1R	0.928949376	MRPL27	NME1	0.902966185
CD163	CD53	0.907887866	TREM2	ALOX5AP	0.861327509
CD163	CD4	0.862870197	TREM2	SPI1	0.857403943
CD163	HMOX1	0.873451633	TREM2	FCER1G	0.861362327
CD163	AIF1	0.857672492	TREM2	TYROBP	0.892816834
CD163	VSIG4	0.936166202	ALOX5AP	SPI1	0.858866765
CD163	FCGR3A	0.88911153	ALOX5AP	CD14	0.865275036
CD163	CD14	0.910202365	ALOX5AP	LAPTM5	0.892303347
CD163	LAPTM5	0.895183421	DHX16	ABCF1	0.862751127
CD163	SRGN	0.871826839	FBXL6	HGH1	0.888178245
CD163	C1QB	0.868295012	MZT2A	MZT2B	0.898836141
CD163	C1QC	0.883288777	TOX4	SUPT16H	0.881511657
ZNF526	ZNF574	0.861387907	TOX4	PRMT5	0.88130788
HOXB3	HOXB4	0.885093486	ZBTB45	TRIM28	0.85099702
HOXB3	HOXB2	0.851393189	AIF1	SPI1	0.935618203
KIF4A	NCAPH	0.8630465	AIF1	ARHGDIB	0.868613255
KIF4A	RACGAP1	0.858589879	AIF1	CD14	0.896647655
LY86	GIMAP4	0.868581423	AIF1	LAPTM5	0.858253222

LY86	C1orf162	0.864016494	AIF1	HLA-DPB1	0.858737291
LY86	RNASE6	0.887994307	AIF1	FCER1G	0.934603524
LY86	CD53	0.857243039	AIF1	C1QA	0.937493227
LY86	TREM2	0.886326446	AIF1	TYROBP	0.92737234
LY86	AIF1	0.892633287	AIF1	C1QB	0.908256622
LY86	SPI1	0.883185267	AIF1	C1QC	0.897532549
LY86	FCER1G	0.901382111	APOL2	APOL1	0.924543984
LY86	TYROBP	0.927505105	VSIG4	FCGR3A	0.883003028
VCAN	PDGFRB	0.854780127	VSIG4	CD14	0.931643335
VCAN	THBS2	0.873672014	VSIG4	LAPTM5	0.898974317
VCAN	COL5A2	0.940040234	VSIG4	C1QA	0.895497352
VCAN	ANTXR1	0.858180054	VSIG4	C1QB	0.938318164
VCAN	CTSK	0.910545342	VSIG4	C1QC	0.941628881
VCAN	DCN	0.889857305	FCGR3A	CD14	0.858715839
VCAN	COL5A1	0.922460515	FCGR3A	LAPTM5	0.935732142
VCAN	SERPINF1	0.862571975	FCGR3A	SRGN	0.872265249
VCAN	MMP2	0.869584606	FCGR3A	C1QA	0.851155591
VCAN	LUM	0.890725765	FCGR3A	C1QB	0.904656033
VCAN	AEBP1	0.9034897	FCGR3A	C1QC	0.907710652
VCAN	FN1	0.874929632	ZNF865	EPN1	0.877248702
VCAN	COL3A1	0.908242637	CSTF1	RTF2	0.859799894
VCAN	COL1A2	0.858760586	RFC4	ACTL6A	0.860265296
VCAN	SPARC	0.873616818	RAF1	MKRN2	0.856650949
VCAN	COL1A1	0.900973696	PPRC1	GBF1	0.857552685
ICE1	TENT4A	0.858533578	ZFP91	CTNND1	0.856373598
EVI2B	FPR3	0.882715422	SERPINE1	THBS1	0.850990803
EVI2B	FERMT3	0.87518611	AKAP8L	BRD4	0.857468486
EVI2B	SELPLG	0.869030261	MRPS36	TBCA	0.867770332
EVI2B	C3AR1	0.912602824	OAS2	OAS1	0.921678764
EVI2B	FCGR2A	0.903244877	OAS2	IFIT1	0.900193852
EVI2B	GIMAP4	0.899219462	OAS2	OAS3	0.945402135
EVI2B	C1orf162	0.862389429	OAS2	MX1	0.927765246
EVI2B	LSP1	0.896076534	OAS2	IFI44	0.873256932
EVI2B	CYBB	0.924663834	OAS2	IFIT3	0.930206158
EVI2B	ITGB2	0.903153581	PRPF4	POLE3	0.875494182
EVI2B	RNASE6	0.883744532	ZNF414	RAB11B	0.902344055
EVI2B	CSF1R	0.871596885	COL6A3	EMILIN1	0.875306489
EVI2B	CD53	0.951282714	COL6A3	CTSK	0.856994163
EVI2B	CD4	0.906657439	COL6A3	COL5A1	0.959290635
EVI2B	ALOX5AP	0.863597732	COL6A3	MMP2	0.934484348
EVI2B	AIF1	0.902277003	COL6A3	AEBP1	0.913720696
EVI2B	FCGR3A	0.864254561	COL6A3	FN1	0.857768047
EVI2B	SPI1	0.881178766	COL6A3	COL6A2	0.902018057
EVI2B	LYZ	0.85799708	COL6A3	COL6A1	0.859376544
EVI2B	LAPTM5	0.936588648	COL6A3	COL3A1	0.953687228
EVI2B	FCER1G	0.867589744	COL6A3	COL1A2	0.907179529
GTF3C4	BRD3	0.878436563	COL6A3	SPARC	0.900363847
BUB1B	NUSAP1	0.892960584	COL6A3	COL1A1	0.944353604
STAB1	FERMT3	0.878922501	SCNM1	VPS72	0.867615602
STAB1	CSF1R	0.921175556	PSMB9	PSMB8	0.916384698
STAB1	CD14	0.864213587	PIN1	UBL5	0.8732385
FPR3	FERMT3	0.854563538	OAS1	IFIT1	0.864989361
FPR3	C3AR1	0.931176571	OAS1	OAS3	0.913402917
FPR3	FCGR2A	0.920231944	OAS1	IFI35	0.850529864
FPR3	CYBB	0.923203309	OAS1	MX1	0.91444628
FPR3	ITGB2	0.86448096	OAS1	IFI44	0.892922306

FPR3	RNASE6	0.854740689	OAS1	IFIT3	0.8892461
FPR3	CSF1R	0.882089219	EMILIN1	DCN	0.853370489
FPR3	CD53	0.918157804	EMILIN1	AEBP1	0.860367912
FPR3	CD4	0.869843322	NSFL1C	PSMF1	0.857918482
FPR3	VSIG4	0.851015487	MMP11	CTSK	0.867099038
FPR3	FCGR3A	0.896828969	IFIT1	OAS3	0.865025248
FPR3	LAPTM5	0.927970677	IFIT1	MX1	0.897691716
INTS4	ALG8	0.88441364	IFIT1	IFI44	0.901813708
INTS4	CLNS1A	0.883283232	IFIT1	IFIT3	0.946047773
E4F1	TELO2	0.870871776	IFIT1	PLSCR1	0.881284039
FBXW2	RABGAP1	0.87797924	IFIT1	IFI6	0.855348586
NKG7	CCL5	0.929718537	OAS3	MX1	0.933494828
KIF1B	UBE4B	0.858024186	OAS3	IFIT3	0.884705984
GBP4	TAP1	0.862433724	SPI1	ARHGDIB	0.85208551
S100A8	S100A9	0.900304053	SPI1	CD14	0.937113876
MAD2L1	CCNA2	0.883561383	SPI1	LAPTM5	0.886118181
HERC6	RSAD2	0.885290712	SPI1	HLA-DPB1	0.85267296
HERC6	IFI44L	0.877540614	SPI1	FCER1G	0.944919101
FERMT3	SELPLG	0.914322866	SPI1	C1QA	0.92821689
FERMT3	C3AR1	0.921147237	SPI1	TYROBP	0.927772755
FERMT3	FCGR2A	0.891395207	SPI1	C1QB	0.899201775
FERMT3	LSP1	0.914030091	SPI1	C1QC	0.916018332
FERMT3	CYBB	0.931181156	CPSF3	YWHAQ	0.856670185
FERMT3	ITGB2	0.926009453	MYO9B	WIZ	0.875465278
FERMT3	PLEKHO2	0.855980962	MYO9B	CHERP	0.851308541
FERMT3	RNASE6	0.877444111	MYO9B	BRD4	0.862961797
FERMT3	CSF1R	0.940114892	BFAR	CEP20	0.889078328
FERMT3	CD53	0.931550187	PLAU	FN1	0.888523928
FERMT3	CD4	0.912022139	XRCC1	ZNF428	0.872277231
FERMT3	ALOX5AP	0.854465587	TOP2A	TPX2	0.864345394
FERMT3	AIF1	0.886736673	IFI35	UBE2L6	0.864701392
FERMT3	SPI1	0.942214023	TIMM50	YIF1B	0.85939017
FERMT3	CD14	0.892652419	TIMM50	PSMD8	0.869546707
FERMT3	LAPTM5	0.91331598	ANTXR1	AEBP1	0.855135934
FERMT3	FCER1G	0.850988608	MRPL52	DAD1	0.896445664
FERMT3	C1QB	0.86206958	CCDC97	HNRNPUL1	0.862067685
FERMT3	C1QC	0.873689091	POLR1D	GTF3A	0.85244206
RSAD2	IFIH1	0.883012218	USE1	BABAM1	0.885110231
RSAD2	IFI44L	0.931415156	MX1	IFI44	0.899241305
RSAD2	OAS2	0.920561106	MX1	IFIT3	0.918381722
RSAD2	OAS1	0.906128971	CTSK	SERPINF1	0.896774616
RSAD2	IFIT1	0.865949013	CTSK	MMP2	0.875871662
RSAD2	MX1	0.899026822	CTSK	LUM	0.905108337
RSAD2	IFI44	0.913468014	CTSK	COL3A1	0.857580423
RSAD2	IFIT3	0.856108111	WIZ	CHERP	0.883558218
UFD1	RANBP1	0.850818752	WIZ	BRD4	0.899035556
EPOR	RGL3	0.861069253	DCTN3	RPP25L	0.854712261
PPIE	TRIT1	0.872225	SLC35E1	CHERP	0.869640286
SELPLG	C3AR1	0.898746572	DCN	MMP2	0.867546841
SELPLG	FCGR2A	0.881450329	DCN	LUM	0.896271201
SELPLG	LSP1	0.869197931	TAP1	HLA-B	0.863315202
SELPLG	CYBB	0.891384392	TFE3	OTUD5	0.882166095
SELPLG	ITGB2	0.857685413	H4C9	H2BC12	0.953932562
SELPLG	RNASE6	0.885986328	COL5A1	MMP2	0.956854016
SELPLG	CSF1R	0.890459018	COL5A1	AEBP1	0.915830305
SELPLG	CD53	0.907266939	COL5A1	FN1	0.883548629

SELPLG	CD4	0.901600187	COL5A1	COL6A2	0.919614761
SELPLG	AIF1	0.860544775	COL5A1	COL6A1	0.860904606
SELPLG	SPI1	0.897740662	COL5A1	COL3A1	0.965280099
SELPLG	CD14	0.854462414	COL5A1	COL1A2	0.931564986
SELPLG	LAPTM5	0.897139637	COL5A1	BGN	0.851886413
SELPLG	C1QB	0.864843819	COL5A1	SPARC	0.928414979
SELPLG	C1QC	0.873308899	COL5A1	COL1A1	0.974422283
NUP107	CCT2	0.865722791	MT1X	MT2A	0.856207031
LOX	POSTN	0.85679462	DDA1	BABAM1	0.896004084
LOX	THBS2	0.859414967	NDUFAF8	ANAPC11	0.86771617
LOX	FN1	0.866254633	YIF1B	PSMD8	0.884872501
LOX	COL3A1	0.866829282	IFI44	IFIT3	0.902056478
BTN3A2	BTN3A3	0.85669731	SMARCA4	ILF3	0.853711698
BTN3A2	BTN3A1	0.85399991	ANAPC11	CENPX	0.896444966
BPTF	DDX42	0.873652333	CHERP	BRD4	0.880962532
HLA-F	HLA-A	0.900157423	DHPS	GET3	0.864558613
HLA-F	HLA-B	0.891726862	DHPS	TRIR	0.875456942
C3AR1	FCGR2A	0.958031883	DGAT1	BOP1	0.85292383
C3AR1	GIMAP4	0.878677416	HGH1	ZC3H3	0.874718571
C3AR1	LSP1	0.85981544	HGH1	SHARPIN	0.858843987
C3AR1	CYBB	0.959081523	HGH1	HSF1	0.875516985
C3AR1	ITGB2	0.924180386	MRPL54	UQCR11	0.856763275
C3AR1	RNASE6	0.95105163	TMEM14B	TMEM14C	0.913122189
C3AR1	CSF1R	0.94580825	SERPINF1	MMP2	0.883107149
C3AR1	CD53	0.965641673	SERPINF1	LUM	0.862489903
C3AR1	CD4	0.929654236	MRPL36	NDUFS6	0.91038552
C3AR1	ALOX5AP	0.873560013	UQCRB	COX6C	0.87929439
C3AR1	AIF1	0.930809924	UQCRB	RPL30	0.865460058
C3AR1	VSIG4	0.930786346	EPRS1	IARS2	0.863897246
C3AR1	FCGR3A	0.944146319	SUPT5H	PAF1	0.888196012
C3AR1	SPI1	0.90645379	SUPT5H	SAMD4B	0.929892219
C3AR1	CD14	0.940462714	MICOS13	ALKBH7	0.88007688
C3AR1	LAPTM5	0.961456289	ZC3H3	SCRIB	0.850154273
C3AR1	SRGN	0.869041703	ZC3H3	HSF1	0.850666176
C3AR1	FCER1G	0.869216525	VPS72	PSMD4	0.869905412
C3AR1	C1QA	0.866531795	IFIT3	PLSCR1	0.86339766
C3AR1	C1QB	0.906500323	PAF1	MED29	0.899593497
C3AR1	C1QC	0.919962032	PAF1	SAMD4B	0.861113055
LHFPL6	PDGFRB	0.882731825	BABAM1	MRPL34	0.865195141
LHFPL6	EMILIN1	0.9135904	BABAM1	FAM32A	0.854716289
SENP5	DLG1	0.858512588	CTHRC1	SPARC	0.850138563
SENP5	LSG1	0.862784831	C1S	C1R	0.90392141
GRIPAP1	OTUD5	0.85372316	LSM7	UQCR11	0.855389037
DLGAP5	CDKN3	0.861436882	LSM7	RPS15	0.850326275
NAA35	ISCA1	0.85076579	BOP1	HSF1	0.865168418
ARFGEF1	NCOA2	0.877114424	PARL	POLR2H	0.855720062
NCAPH	BUB1	0.865794054	PARL	MRPL47	0.88029321
NCAPH	CENPA	0.86465854	COL4A1	COL4A2	0.915273186
NCAPH	KIF2C	0.873055016	MRFAP1L1	MRFAP1	0.885256652
NCAPH	KIFC1	0.858486356	SAMD4B	ACTN4	0.855737043
NCAPH	TOP2A	0.856737944	POLR2H	MRPL47	0.853050738
NCAPH	TPX2	0.883256887	MYBL2	TPX2	0.868262683
C2CD5	ETNK1	0.858568168	SLC52A2	HSF1	0.87425004
C2CD5	AEBP2	0.858832556	ARHGDIB	HLA-DPA1	0.853075196
SAP30BP	NUP85	0.857311182	ARHGDIB	HLA-DPB1	0.879250199
SAP30BP	SRP68	0.8835543	SCGB1D2	SCGB2A1	0.869001822

FCGR2A	CYBB	0.930116721	HLA-DMA	HLA-DPA1	0.930100081
FCGR2A	ITGB2	0.899461625	HLA-DMA	HLA-DPB1	0.934688559
FCGR2A	RNASE6	0.913730702	HLA-DMA	HLA-DRB1	0.936649592
FCGR2A	CSF1R	0.904035987	HLA-DMA	HLA-DRA	0.921763203
FCGR2A	CD53	0.941044319	HLA-DMA	CD74	0.918726953
FCGR2A	CD4	0.906005107	NELFE	LSM2	0.853871934
FCGR2A	ALOX5AP	0.855791486	NELFE	RNF5	0.859914674
FCGR2A	AIF1	0.915632141	TMEM258	TRMT112	0.896593259
FCGR2A	VSIG4	0.906477142	SELENOH	POLR2G	0.861917945
FCGR2A	FCGR3A	0.923074161	MMP2	LUM	0.901222835
FCGR2A	SPI1	0.88177312	MMP2	AEBP1	0.913203944
FCGR2A	CD14	0.902425282	MMP2	TIMP2	0.855347771
FCGR2A	LAPTM5	0.938145889	MMP2	FN1	0.892858823
FCGR2A	SRGN	0.872327304	MMP2	COL6A2	0.884965862
FCGR2A	FCER1G	0.877613974	MMP2	COL3A1	0.961826616
FCGR2A	C1QB	0.885959011	MMP2	COL1A2	0.93027246
FCGR2A	C1QC	0.896817371	MMP2	BGN	0.852887791
SNAPC4	INPP5E	0.862215667	MMP2	SPARC	0.921106518
GIMAP4	C1orf162	0.86716308	MMP2	COL1A1	0.951457617
GIMAP4	CYBB	0.860115611	PSME2	PSME1	0.923747144
GIMAP4	RNASE6	0.897383378	HLA-DPA1	HLA-DPB1	0.949114577
GIMAP4	CD53	0.899335033	HLA-DPA1	HLA-DRB1	0.926683891
GIMAP4	AIF1	0.897153753	HLA-DPA1	HLA-DRA	0.95246984
GIMAP4	FCGR3A	0.867391628	HLA-DPA1	CD74	0.933685302
GIMAP4	SPI1	0.867723169	FARSA	RAD23A	0.875791731
GIMAP4	LAPTM5	0.873306043	CD14	LAPTM5	0.925031833
GIMAP4	FCER1G	0.855399944	CD14	FCER1G	0.889944888
GIMAP4	C1QA	0.858309723	CD14	C1QA	0.919135293
GIMAP4	TYROBP	0.854435596	CD14	TYROBP	0.8589277
GIMAP4	C1QB	0.859431459	CD14	C1QB	0.936017381
GIMAP4	C1QC	0.859709051	CD14	C1QC	0.951320463
OLFML2B	POSTN	0.869057417	LUM	COL3A1	0.887884406
OLFML2B	FN1	0.85172701	LUM	SPARC	0.892334782
BTN3A3	BTN3A1	0.882672689	LAMTOR4	FIS1	0.852064543
POSTN	LUM	0.883475037	LAMTOR4	ATP5MF	0.851286283
POSTN	COL3A1	0.878905512	POLR2I	PSENEN	0.85494318
HOXB4	HOXB2	0.858738269	LAMTOR5	GNG5	0.854748449
C1orf162	LSP1	0.871306278	SHARPIN	HSF1	0.859472912
C1orf162	RNASE6	0.854998995	SHARPIN	MAF1	0.872249782
C1orf162	CD53	0.875905885	HSF1	MAF1	0.884301674
C1orf162	ALOX5AP	0.851250551	HSF1	GRINA	0.869810731
C1orf162	AIF1	0.872426739	SSNA1	EDF1	0.869287212
C1orf162	SPI1	0.892856624	ATP5F1E	RPS21	0.867116585
C1orf162	FCER1G	0.881675092	CCDC167	LSM2	0.858259009
C1orf162	TYROBP	0.889913393	CCDC167	SNRPC	0.893774704
AP1G1	IST1	0.890078629	RPL36A	RPL39	0.851366604
POM121	POM121C	0.909206378	SCAND1	ROMO1	0.850254127
MTOR	MFN2	0.903761557	LAPTM5	SRGN	0.864689147
ECM1	THBS2	0.891255296	LAPTM5	C1QA	0.867392548
ECM1	MMP11	0.899037004	LAPTM5	C1QB	0.912518274
ECM1	CTSK	0.859003072	LAPTM5	C1QC	0.933620792
ECM1	DCN	0.864143066	AEBP1	COL6A2	0.893156123
ECM1	LUM	0.873537776	AEBP1	COL6A1	0.851200799
ECM1	COL3A1	0.856538786	AEBP1	COL3A1	0.878921401
ECM1	COL1A1	0.862933101	AEBP1	COL1A2	0.852807916
C1orf109	GNL2	0.906122028	AEBP1	BGN	0.873898037

C1orf109	MEAF6	0.866166282	AEBP1	SPARC	0.868615174
LY96	AIF1	0.880258643	AEBP1	COL1A1	0.907222109
DLG1	PAK2	0.890962584	NDUFA2	UQCRQ	0.852671483
UQCRHL	UQCRH	0.889294757	FN1	COL3A1	0.878557891
TOPORS	KLHL9	0.862226125	FN1	COL1A1	0.854720645
NID1	PDGFRB	0.89837168	SRGN	C1QA	0.863213582
NID1	EMILIN1	0.853217518	SRGN	C1QB	0.885777554
MED14	USP9X	0.853277697	SRGN	C1QC	0.877392569
MED14	DDX3X	0.860668929	EIF3G	CDC37	0.85957716
USP36	UBE2O	0.863290102	CCN1	CCN2	0.904400055
IFIH1	OAS2	0.866849702	WDR83OS	TRIR	0.873778527
SDAD1	G3BP2	0.872761412	HLA-DPB1	HLA-DRB1	0.936418121
SDAD1	USO1	0.886268382	HLA-DPB1	HLA-DRA	0.940253945
ZNF212	ZNF282	0.853171378	HLA-DPB1	CD74	0.929139472
LRRC14	CYHR1	0.864232363	FCER1G	C1QA	0.926591814
GAK	LETM1	0.873200295	FCER1G	TYROBP	0.973295173
FBXO28	BROX	0.864179409	FCER1G	C1QB	0.894536252
CREB3L1	COL6A3	0.895225197	FCER1G	C1QC	0.893021723
CREB3L1	COL1A2	0.857016336	RPS29	RPL35	0.865354328
CREB3L1	COL1A1	0.859092128	EIF3H	EIF3E	0.851477028
TENT4A	NSUN2	0.874146265	STOML2	CLTA	0.853200384
MRPS23	SUPT4H1	0.9075415	LSM2	SNRPC	0.91364751
HLA-DOA	HLA-DPA1	0.860508794	C1QA	TYROBP	0.917568486
HLA-DOA	HLA-DPB1	0.860191134	C1QA	C1QB	0.978860322
HLA-DOA	HLA-DRA	0.855191457	C1QA	C1QC	0.969792162
LSP1	CYBB	0.866266369	RPS15A	RPS14	0.851550722
LSP1	ITGB2	0.889725819	COL6A2	COL6A1	0.961250495
LSP1	CSF1R	0.866978461	COL6A2	COL3A1	0.871963072
LSP1	CD53	0.891539719	COL6A2	COL1A2	0.884186716
LSP1	CD4	0.877569521	COL6A2	COL1A1	0.93627083
LSP1	ALOX5AP	0.855258746	NDUFB10	ELOB	0.862645812
LSP1	AIF1	0.857410424	COL6A1	COL1A1	0.877258931
LSP1	SPI1	0.927359587	FOS	EGR1	0.934987206
LSP1	LAPTM5	0.878214007	FOS	DUSP1	0.880177495
LSP1	FCER1G	0.853321212	UXT	NDUFB11	0.872674945
DCAF13	SLC25A32	0.856714473	UQCRQ	HINT1	0.873937672
ANGPTL2	PDGFRB	0.855283929	TYROBP	C1QB	0.875543608
IFI44L	OAS2	0.881586614	TYROBP	C1QC	0.871928246
IFI44L	OAS1	0.890348773	C1QB	C1QC	0.989713211
IFI44L	IFIT1	0.860233609	RPL14	RPL32	0.85269276
IFI44L	MX1	0.883027214	COX7C	RPS14	0.857395779
IFI44L	IFI44	0.947974562	ISG15	IFI6	0.910690254
IFI44L	ISG15	0.862247938	RPL23	RPL19	0.897720678
IFI44L	IFI6	0.879481662	RPL34	RPS13	0.877958743
IFIT2	IFIT3	0.897624651	COL3A1	COL1A2	0.914197986
YEATS2	EIF4G1	0.865389271	COL3A1	SPARC	0.942993122
GTF3C1	RNF40	0.857559458	COL3A1	COL1A1	0.95990078
C1orf174	RER1	0.867733229	COL1A2	SPARC	0.884760737
CENPA	KIF2C	0.860941712	COL1A2	COL1A1	0.946926656
CENPA	TPX2	0.853151373	BGN	SPARC	0.859856478
POP4	C19orf12	0.855626768	RPL27A	RPS13	0.932272317
MSH6	MSH2	0.891681031	RPL27A	RPS27	0.85795659
DNAJC11	NADK	0.872521112	HLA-DRB1	HLA-DRA	0.952291916
DNAJC11	MFN2	0.891967181	HLA-DRB1	CD74	0.924036633
PDGFRB	COL5A2	0.897674971	SPARC	COL1A1	0.9131586
PDGFRB	COL6A3	0.852488703	UBA52	RPL18A	0.866650459

PDGFRB	EMILIN1	0.887193578	HLA-A	HLA-B	0.866432034
PDGFRB	COL5A1	0.894235408	RPS13	RPL32	0.854694315
PDGFRB	AEBP1	0.852235993	RPS9	RPL18	0.880705024
PDGFRB	COL6A2	0.854914276	RPS9	RPL13A	0.889536484
PDGFRB	COL1A1	0.862140805	RPS9	RPS11	0.9072898
OPA1	ATP13A3	0.878372791	FAU	RPS14	0.857227039
OPA1	LSG1	0.87398658	RPL18	RPL13A	0.883334247
ANAPC2	NDOR1	0.863721026	RPL18	RPS11	0.922697274
TONSL	RECQL4	0.86587639	RPL15	RPSA	0.870393081
TONSL	CPSF1	0.857466476	RPL15	RPL32	0.850326243
NIP7	UTP4	0.850797603	RPL15	RPS8	0.863602334
UBN1	GLYR1	0.859351821	KRT18	KRT8	0.871148542
BRD3	FUBP3	0.869875868	RPS27A	RPS10	0.860805343
FAM193A	ADD1	0.881387235	RPS27A	RPS18	0.873019267
CYBB	ITGB2	0.942080688	IFITM1	IFITM3	0.865993794
CYBB	RNASE6	0.905619722	RPS10	RPS18	0.944124608
CYBB	CSF1R	0.953256196	RPS17	RPLP1	0.866566198
CYBB	CD53	0.962621821	RPL32	RPL29	0.883107652
CYBB	CD4	0.931591721	HLA-DRA	CD74	0.933291788
CYBB	ALOX5AP	0.88796333	RPS8	RPS18	0.860932444
CYBB	AIF1	0.912842719	RPL10A	RPS18	0.871699245
CYBB	VSIG4	0.894644892	RPL13A	RPS11	0.915275202
CYBB	FCGR3A	0.90450579			
CYBB	SPI1	0.891543876			
CYBB	LYZ	0.851062426			
CYBB	CD14	0.909938771			
CYBB	LAPTM5	0.958418727			
CYBB	SRGN	0.856594033			
CYBB	C1QB	0.870834605			
CYBB	C1QC	0.878705298			
EID2	TIMM50	0.882491315			
EID2	MED29	0.85236395			
ITGB2	RNASE6	0.857832279			
ITGB2	CSF1R	0.927802862			
ITGB2	CD53	0.921371156			
ITGB2	CD4	0.912392551			
ITGB2	ALOX5AP	0.887765855			
ITGB2	AIF1	0.876776661			
ITGB2	SPI1	0.88431402			
ITGB2	LYZ	0.851718264			
ITGB2	CD14	0.871796299			
ITGB2	LAPTM5	0.922555558			
SMU1	DCAF12	0.884517182			
MAEA	NELFA	0.850690756			
MAEA	CTBP1	0.85387948			
TRIM39-RPP21	RPP21	1			
MRM2	EIF3B	0.877038365			
CLASRP	SNRNP70	0.871569716			
HMOX2	NMRAL1	0.852288355			
SFRP4	THBS2	0.8599293			
SFRP4	COL3A1	0.852121593			
NUP54	G3BP2	0.88322627			
NR4A1	FOSB	0.902720419			
FOXK2	NPLOC4	0.853814847			
FOXK2	CSNK1D	0.852745039			
RNASE6	CSF1R	0.894723079			

RNASE6	CD53	0.941055605
RNASE6	CD4	0.875962705
RNASE6	TREM2	0.873018962
RNASE6	AIF1	0.928700013
RNASE6	VSIG4	0.886690287
RNASE6	FCGR3A	0.916173919
RNASE6	SPI1	0.896076573
RNASE6	CD14	0.916045171
RNASE6	LAPTM5	0.924750796
RNASE6	SRGN	0.889620265
RNASE6	FCER1G	0.893576088
RNASE6	C1QA	0.907369181
RNASE6	TYROBP	0.886584381
RNASE6	C1QB	0.928154716
RNASE6	C1QC	0.941102552
KRI1	CDC37	0.856910956
HLA-DQA1	HLA-DQB1	0.893363385
HLA-DQA1	HLA-DPB1	0.863214835
HBA2	HBB	0.855611112
CHD8	SUPT16H	0.890770748
KIF2C	CDC20	0.913285817
CYHR1	HSF1	0.872080861
THBS2	COL5A2	0.889224633
THBS2	MMP11	0.893969831
THBS2	DCN	0.882308812
THBS2	THBS1	0.879289137
THBS2	COL5A1	0.889521849
THBS2	ACTA2	0.879419688
THBS2	MMP2	0.862629976
THBS2	LUM	0.902067291
THBS2	AEBP1	0.867385046
THBS2	FN1	0.927304256
THBS2	COL3A1	0.930781618
THBS2	COL1A2	0.877738714
THBS2	SPARC	0.859057018
THBS2	COL1A1	0.913360985
CSF1R	CD53	0.920075617
CSF1R	CD4	0.929798907
CSF1R	HMOX1	0.853157155
CSF1R	ALOX5AP	0.85098279
CSF1R	AIF1	0.883649132
CSF1R	VSIG4	0.885167515
CSF1R	FCGR3A	0.877457867
CSF1R	SPI1	0.889242205
CSF1R	CD14	0.92378023
CSF1R	LAPTM5	0.91980525
CSF1R	C1QB	0.854024377
CSF1R	C1QC	0.877019195
CD53	CD4	0.92676949
CD53	ALOX5AP	0.894236597
CD53	AIF1	0.947880712
CD53	VSIG4	0.899499251
CD53	FCGR3A	0.922015994
CD53	CTSS	0.87322026
CD53	SPI1	0.925047937
CD53	LYZ	0.853947549

CD53	CD14	0.924857574
CD53	LAPTM5	0.98015234
CD53	SRGN	0.911558909
CD53	FCER1G	0.925242766
CD53	C1QA	0.885587055
CD53	TYROBP	0.885098987
CD53	C1QB	0.911705936
CD53	C1QC	0.917294709
PARP9	DTX3L	0.916102778
TMEM176A	TMEM176B	0.958641214
FOSB	FOS	0.855190352
FOSB	EGR1	0.865962109
FOSB	DUSP1	0.877608443
GLG1	MBTPS1	0.856238041
NBL1	MICOS10-NBL1	1
COL5A2	COL6A3	0.863701345
COL5A2	MMP11	0.850101278
COL5A2	ANTXR1	0.851586702
COL5A2	CTSK	0.925698227
COL5A2	DCN	0.88070425
COL5A2	COL5A1	0.955247162
COL5A2	SERPINF1	0.850699517
COL5A2	MMP2	0.880260494
COL5A2	LUM	0.908323028
COL5A2	AEBP1	0.917293436
COL5A2	FN1	0.85667592
COL5A2	COL3A1	0.92466024
COL5A2	COL1A2	0.896766182
COL5A2	SPARC	0.907895971
COL5A2	COL1A1	0.923751828
MAP2K7	XAB2	0.876722697
CD4	ALOX5AP	0.850200577
CD4	AIF1	0.882150398
CD4	FCGR3A	0.852130398
CD4	SPI1	0.908288915
CD4	CD14	0.878053461
CD4	LAPTM5	0.913553844
CD4	FCER1G	0.853741265
FAAP100	HGS	0.876031085
TREM2	SPI1	0.85493792
TREM2	TYROBP	0.883497711
ALOX5AP	AIF1	0.860542166
ALOX5AP	SPI1	0.868184737
ALOX5AP	LAPTM5	0.881425353
UTP11	SF3A3	0.85546335
LSG1	PAK2	0.880876509
RACGAP1	FAM83D	0.850172642
AIF1	VSIG4	0.88872408
AIF1	FCGR3A	0.874928395
AIF1	SPI1	0.929339793
AIF1	ARHGDIB	0.865014087
AIF1	CD14	0.93812817
AIF1	LAPTM5	0.917135812
AIF1	SRGN	0.873522043
AIF1	FCER1G	0.930455812
AIF1	C1QA	0.904606649

AIF1	TYROBP	0.911018782
AIF1	C1QB	0.900123898
AIF1	C1QC	0.89362658
APOL2	APOL1	0.888144585
DNAJA2	VPS35	0.890303362
VSIG4	FCGR3A	0.88653293
VSIG4	CD14	0.937751746
VSIG4	LAPTM5	0.8869481
VSIG4	SRGN	0.871178
VSIG4	C1QB	0.881312076
VSIG4	C1QC	0.894623345
FCGR3A	CD14	0.884257797
FCGR3A	LAPTM5	0.92561976
FCGR3A	SRGN	0.850872558
FCGR3A	C1QA	0.861380125
FCGR3A	C1QB	0.902950831
FCGR3A	C1QC	0.91628038
TYK2	RAVER1	0.852619462
GNL2	MEAF6	0.855037687
CTSS	LAPTM5	0.879965621
OAS2	OAS1	0.925102803
OAS2	OAS3	0.9268734
OAS2	MX1	0.894034941
OAS2	IFI44	0.861285
PRPF4	POLE3	0.893288496
COL6A3	DCN	0.867323632
COL6A3	COL5A1	0.941600449
COL6A3	COL6A2	0.862358479
COL6A3	COL3A1	0.933843314
COL6A3	COL1A2	0.940773954
COL6A3	SPARC	0.872276058
COL6A3	COL1A1	0.942511219
PSMB9	PSMB8	0.906727787
OAS1	OAS3	0.869470869
OAS1	MX1	0.902757945
OAS1	IFI44	0.895493416
COX17	NDUFB4	0.853402611
EMILIN1	COL6A2	0.874325089
EMILIN1	BGN	0.856348738
TMEM208	CIAO2B	0.862895351
UQCC3	COX8A	0.85157634
MMP11	CTSK	0.850126226
MMP11	MMP2	0.853472732
MMP11	LUM	0.874947694
MMP11	COL3A1	0.885708365
MMP11	COL1A1	0.86767767
IFIT1	IFIT3	0.90013553
SPI1	CD14	0.898411172
SPI1	LAPTM5	0.894729523
SPI1	FCER1G	0.91813623
SPI1	C1QA	0.870211568
SPI1	TYROBP	0.905072489
SPI1	C1QB	0.857004107
SPI1	C1QC	0.858552721
RPS27AP5	RPS27A	0.854222392
G3BP2	USO1	0.897421374

STK40	AKIRIN1	0.854640265
SPTLC1	UBQLN1	0.91070139
TIMM50	PAF1	0.88929944
TIMM50	MED29	0.889052297
TIMM50	PSMC4	0.877325242
ANTXR1	COL5A1	0.857710257
NUDT5	CDC123	0.855331103
MX1	IFI44	0.866007271
ZNF687	PI4KB	0.877889265
CTSK	DCN	0.86455072
CTSK	SERPINF1	0.864082564
CTSK	MMP2	0.854418813
CTSK	LUM	0.897396483
CTSK	AEBP1	0.853534768
DCN	COL5A1	0.875571315
DCN	MMP2	0.880105548
DCN	LUM	0.923228654
DCN	AEBP1	0.899957511
DCN	COL3A1	0.916743206
DCN	COL1A2	0.879026687
DCN	SPARC	0.875815698
DCN	COL1A1	0.925262084
ALG8	CLNS1A	0.892535996
THBS1	COL1A1	0.850686911
H4C9	H2BC12	0.970102833
COL5A1	MMP2	0.892683987
COL5A1	LUM	0.872516452
COL5A1	AEBP1	0.923169758
COL5A1	FN1	0.872002761
COL5A1	COL6A2	0.88071679
COL5A1	COL3A1	0.93982041
COL5A1	COL1A2	0.951550564
COL5A1	SPARC	0.903299353
COL5A1	COL1A1	0.968110235
MT1X	MT1F	0.866442827
YIF1B	PSMD8	0.869387503
EMC4	EMC7	0.857693808
PAK4	SUPT5H	0.891278402
PAK4	PAF1	0.904201095
PAK4	SAMD4B	0.911683855
C14orf119	PSMB5	0.851569884
C14orf119	DAD1	0.898511287
ZMPSTE24	CAP1	0.890961611
STT3A	EI24	0.852595591
LSM10	TRAPPC3	0.893328995
LSM10	MRPS15	0.917397654
MRPL58	ATP5PD	0.866369978
MRPL36	NDUFS6	0.866940824
TRAPPC3	ADPRS	0.886715176
TRAPPC3	MRPS15	0.893524614
SUPT5H	PAF1	0.911028256
SUPT5H	MED29	0.878217406
SUPT5H	SAMD4B	0.900130215
ZC3H3	FAM83H	0.850774411
ZC3H3	SCRIB	0.882209687
PAF1	MED29	0.9032971

PAF1	SAMD4B	0.873810546
GID8	YTHDF1	0.853548734
ZNHIT1	LAMTOR4	0.854833458
PPCS	EBNA1BP2	0.867879865
MRPL20	AURKAIP1	0.910046915
VPS29	ARPC3	0.887862057
C1S	C1R	0.880493286
BOP1	HSF1	0.851744121
COL4A1	COL4A2	0.897499943
MRFAP1L1	MRFAP1	0.906425772
SAMD4B	ACTN4	0.866226999
MRPS12	EIF3K	0.877546889
MRPL57	SAP18	0.874259288
TAGLN	ACTA2	0.850701798
SCGB1D2	SCGB2A1	0.852523333
HLA-DMA	HLA-DPA1	0.876815655
HLA-DMA	HLA-DPB1	0.939072723
HLA-DMA	HLA-DRB1	0.950523252
HLA-DMA	HLA-DRA	0.93160572
HLA-DMA	CD74	0.903048143
ACTA2	COL3A1	0.852006293
MMP2	LUM	0.866896517
MMP2	AEBP1	0.886179591
MMP2	COL3A1	0.876669897
MMP2	COL1A2	0.856575223
MMP2	COL1A1	0.896224096
HLA-DPA1	HLA-DPB1	0.897086868
HLA-DPA1	HLA-DRB1	0.923918534
HLA-DPA1	HLA-DRA	0.911979433
HLA-DPA1	CD74	0.864606639
CD14	LAPTM5	0.908630761
CD14	SRGN	0.869352632
CD14	FCER1G	0.873158058
CD14	C1QA	0.900562409
CD14	TYROBP	0.863622689
CD14	C1QB	0.904461307
CD14	C1QC	0.914459537
LUM	COL3A1	0.936788424
LUM	SPARC	0.894338948
LUM	COL1A1	0.892318276
SHARPIN	MAF1	0.866819561
LAPTM5	SRGN	0.880039159
LAPTM5	FCER1G	0.898810578
LAPTM5	C1QA	0.860702992
LAPTM5	C1QB	0.901601575
LAPTM5	C1QC	0.909671209
AEBP1	COL3A1	0.87510924
AEBP1	COL1A2	0.883705763
AEBP1	SPARC	0.85884575
AEBP1	COL1A1	0.925141515
NDUFA2	UQCRQ	0.90858165
FN1	COL3A1	0.881880095
FN1	SPARC	0.867907504
FN1	COL1A1	0.864097138
SRGN	FCER1G	0.862890966
SRGN	C1QA	0.857473431

SRGN	C1QB	0.873411521
SRGN	C1QC	0.881304349
HLA-DPB1	HLA-DRB1	0.953729278
HLA-DPB1	HLA-DRA	0.94702099
HLA-DPB1	CD74	0.908372676
FCER1G	C1QA	0.906121014
FCER1G	TYROBP	0.969318455
FCER1G	C1QB	0.899385007
FCER1G	C1QC	0.88757841
C1QA	TYROBP	0.906784052
C1QA	C1QB	0.978222209
C1QA	C1QC	0.967262724
PSMB5	DAD1	0.877123669
COL6A2	COL6A1	0.953585034
COL6A2	COL1A2	0.850756018
COL6A2	COL1A1	0.874253018
FOS	EGR1	0.871957546
FOS	DUSP1	0.886303896
TYROBP	C1QB	0.877390062
TYROBP	C1QC	0.871880348
DUSP1	ZFP36	0.8533262
C1QB	C1QC	0.988592747
RPL14	RPL32	0.870110065
ISG15	IFI6	0.887874322
RPL23	RPL19	0.894848768
COL3A1	COL1A2	0.923470391
COL3A1	SPARC	0.930481541
COL3A1	COL1A1	0.957496586
COL1A2	SPARC	0.89503895
COL1A2	COL1A1	0.954503297
RPL31	RPS27	0.872152718
C19orf53	NDUFB7	0.899184688
RPS7	RPL24	0.850780274
RPL27A	RPS13	0.897799222
RPL27A	RPLP2	0.900859542
HLA-DRB1	HLA-DRA	0.970389551
HLA-DRB1	CD74	0.928992196
SPARC	COL1A1	0.898048366
HLA-A	HLA-B	0.89485482
RPS13	RPLP2	0.863678628
RPL18	RPL13A	0.865918643
RPL18	RPS11	0.924131275
RPL15	RPL32	0.872858624
RPL21	TPT1	0.860370417
RPL24	RPL32	0.855251968
RPS10	RPS18	0.881161966
RPL32	RPL29	0.86774383
HLA-DRA	CD74	0.905797555
RPL10A	RPS18	0.879483088
RPL13A	RPS11	0.919107506

Table S2. Top 200 GO biological processes in cohort with better prognosis

ID	Biological processes	Description	GeneRatio	BgRatio	pvalue	p.adjust	qvalue	Count
GO:0002831	respose to stimulus	regulation of response to biotic stimulus	41/804	327/18723	7.6E-10	1.59E-07	1.35E-07	41
GO:0002237	respose to stimulus	response to molecule of bacterial origin	41/804	363/18723	1.65E-08	1.49E-06	1.26E-06	41
GO:0032103	respose to stimulus	positive regulation of response to external stimulus	41/804	427/18723	1.35E-06	5.54E-05	4.69E-05	41
GO:0032102	respose to stimulus	negative regulation of response to external stimulus	40/804	420/18723	2.22E-06	8.6E-05	7.28E-05	40
GO:0032496	respose to stimulus	response to lipopolysaccharide	39/804	343/18723	3.09E-08	2.48E-06	2.1E-06	39
GO:0009615	respose to stimulus	response to virus	38/804	367/18723	5.21E-07	2.4E-05	2.03E-05	38
GO:0060326	respose to stimulus	cell chemotaxis	37/804	310/18723	1.95E-08	1.73E-06	1.46E-06	37
GO:0071216	respose to stimulus	cellular response to biotic stimulus	34/804	246/18723	1.74E-09	2.87E-07	2.43E-07	34
GO:0030595	respose to stimulus	leukocyte chemotaxis	32/804	230/18723	4.44E-09	5.18E-07	4.39E-07	32
GO:0071219	respose to stimulus	cellular response to molecule of bacterial origin	31/804	221/18723	6.35E-09	6.8E-07	5.76E-07	31
GO:0031348	respose to stimulus	negative regulation of defense response	31/804	258/18723	2.37E-07	1.36E-05	1.15E-05	31
GO:0051607	respose to stimulus	defense response to virus	31/804	265/18723	4.29E-07	2.06E-05	1.74E-05	31
GO:0140546	respose to stimulus	defense response to symbiont	31/804	265/18723	4.29E-07	2.06E-05	1.74E-05	31
GO:0050727	respose to stimulus	regulation of inflammatory response	30/804	386/18723	0.001321	0.017419	0.014747	30
GO:0071222	respose to stimulus	cellular response to lipopolysaccharide	28/804	209/18723	9.28E-08	6.57E-06	5.56E-06	28
GO:0042742	respose to stimulus	defense response to bacterium	27/804	350/18723	0.002494	0.02892	0.024483	27
GO:0019058	respose to stimulus	viral life cycle	25/804	317/18723	0.002646	0.030259	0.025617	25
GO:0002221	respose to stimulus	pattern recognition receptor signaling pathway	24/804	172/18723	3.57E-07	1.84E-05	1.56E-05	24
GO:0050920	respose to stimulus	regulation of chemotaxis	24/804	223/18723	3.42E-05	0.000884	0.000749	24
GO:0070372	respose to stimulus	regulation of ERK1 and ERK2 cascade	24/804	309/18723	0.003864	0.041262	0.034932	24
GO:0071560	respose to stimulus	cellular response to transforming growth factor beta stimulus	22/804	250/18723	0.001195	0.016364	0.013854	22
GO:0002833	respose to stimulus	positive regulation of response to biotic stimulus	21/804	168/18723	1.11E-05	0.000333	0.000282	21
GO:0060759	respose to stimulus	regulation of response to cytokine stimulus	18/804	162/18723	0.000213	0.003995	0.003382	18
GO:0002832	respose to stimulus	negative regulation of response to biotic stimulus	14/804	108/18723	0.000214	0.003997	0.003384	14
GO:0071230	respose to stimulus	cellular response to amino acid stimulus	13/804	71/18723	8.86E-06	0.000275	0.000233	13
GO:0090288	respose to stimulus	negative regulation of cellular response to growth factor stimulus	12/804	111/18723	0.002905	0.032855	0.027815	12
GO:0009595	respose to stimulus	detection of biotic stimulus	10/804	38/18723	3.19E-06	0.000115	9.77E-05	10
GO:0098581	respose to stimulus	detection of external biotic stimulus	9/804	25/18723	5.22E-07	2.4E-05	2.03E-05	9
GO:0060760	respose to stimulus	positive regulation of response to cytokine stimulus	8/804	57/18723	0.002861	0.032513	0.027525	8
GO:0036006	respose to stimulus	cellular response to macrophage colony-stimulating factor stimulus	4/804	13/18723	0.001768	0.021999	0.018624	4
GO:0071559	respose to stimulus	response to transforming growth factor beta	22/804	256/18723	0.001619	0.020643	0.017476	22
GO:0031349	respose to stimulus	positive regulation of defense response	22/804	278/18723	0.004439	0.046338	0.039229	22
GO:0001819	metabolic process	positive regulation of cytokine production	52/804	467/18723	3.21E-10	8.21E-08	6.95E-08	52
GO:0019221	metabolic process	cytokine-mediated signaling pathway	47/804	472/18723	7.72E-08	5.55E-06	4.7E-06	47
GO:0001818	metabolic process	negative regulation of cytokine production	30/804	357/18723	0.000373	0.006685	0.005659	30
GO:0051052	metabolic process	regulation of DNA metabolic process	30/804	359/18723	0.00041	0.007228	0.006119	30
GO:0018108	metabolic process	peptidyl-tyrosine phosphorylation	29/804	375/18723	0.001699	0.021485	0.018189	29
GO:0018212	metabolic process	peptidyl-tyrosine modification	29/804	378/18723	0.001913	0.023361	0.019777	29
GO:0002181	metabolic process	cytoplasmic translation	25/804	148/18723	4.13E-09	5.14E-07	4.35E-07	25

GO:0071706	metabolic process	tumor necrosis factor superfamily cytokine production	24/804	186/18723	1.51E-06	6.09E-05	5.16E-05	24
GO:1903555	metabolic process	regulation of tumor necrosis factor superfamily cytokine production	24/804	186/18723	1.51E-06	6.09E-05	5.16E-05	24
GO:0032640	metabolic process	tumor necrosis factor production	23/804	181/18723	3.23E-06	0.000115	9.77E-05	23
GO:0032680	metabolic process	regulation of tumor necrosis factor production	23/804	181/18723	3.23E-06	0.000115	9.77E-05	23
GO:0032609	metabolic process	interferon-gamma production	22/804	112/18723	1.92E-09	2.94E-07	2.49E-07	22
GO:0032649	metabolic process	regulation of interferon-gamma production	22/804	112/18723	1.92E-09	2.94E-07	2.49E-07	22
GO:1903557	metabolic process	positive regulation of tumor necrosis factor superfamily cytokine production	19/804	107/18723	1.32E-07	8.68E-06	7.35E-06	19
GO:0032642	metabolic process	regulation of chemokine production	18/804	98/18723	1.65E-07	1.04E-05	8.83E-06	18
GO:0032602	metabolic process	chemokine production	18/804	99/18723	1.94E-07	1.16E-05	9.82E-06	18
GO:0032760	metabolic process	positive regulation of tumor necrosis factor production	18/804	103/18723	3.61E-07	1.84E-05	1.56E-05	18
GO:0032729	metabolic process	positive regulation of interferon-gamma production	17/804	72/18723	7.15E-09	7.48E-07	6.33E-07	17
GO:0032479	metabolic process	regulation of type I interferon production	17/804	95/18723	5.35E-07	2.42E-05	2.05E-05	17
GO:0032606	metabolic process	type I interferon production	17/804	95/18723	5.35E-07	2.42E-05	2.05E-05	17
GO:0032635	metabolic process	interleukin-6 production	17/804	165/18723	0.000757	0.011732	0.009932	17
GO:0032675	metabolic process	regulation of interleukin-6 production	17/804	165/18723	0.000757	0.011732	0.009932	17
GO:0032722	metabolic process	positive regulation of chemokine production	15/804	71/18723	2.64E-07	1.5E-05	1.27E-05	15
GO:0032608	metabolic process	interferon-beta production	11/804	56/18723	2.17E-05	0.000588	0.000498	11
GO:0032648	metabolic process	regulation of interferon-beta production	11/804	56/18723	2.17E-05	0.000588	0.000498	11
GO:0032481	metabolic process	positive regulation of type I interferon production	11/804	58/18723	3.07E-05	0.000811	0.000687	11
GO:0032613	metabolic process	interleukin-10 production	11/804	62/18723	5.85E-05	0.001382	0.00117	11
GO:0032623	metabolic process	interleukin-2 production	11/804	62/18723	5.85E-05	0.001382	0.00117	11
GO:0032653	metabolic process	regulation of interleukin-10 production	11/804	62/18723	5.85E-05	0.001382	0.00117	11
GO:0032663	metabolic process	regulation of interleukin-2 production	11/804	62/18723	5.85E-05	0.001382	0.00117	11
GO:0032728	metabolic process	positive regulation of interferon-beta production	10/804	39/18723	4.13E-06	0.000143	0.000121	10
GO:0032689	metabolic process	negative regulation of interferon-gamma production	8/804	42/18723	0.00036	0.006494	0.005498	8
GO:0032703	metabolic process	negative regulation of interleukin-2 production	6/804	27/18723	0.000839	0.012545	0.010621	6
GO:0032480	metabolic process	negative regulation of type I interferon production	6/804	30/18723	0.001509	0.019498	0.016506	6
GO:0032633	metabolic process	interleukin-4 production	6/804	33/18723	0.002523	0.029034	0.02458	6
GO:0032673	metabolic process	regulation of interleukin-4 production	6/804	33/18723	0.002523	0.029034	0.02458	6
GO:0032743	metabolic process	positive regulation of interleukin-2 production	6/804	34/18723	0.002954	0.033248	0.028147	6
GO:0072567	metabolic process	chemokine (C-X-C motif) ligand 2 production	5/804	20/18723	0.001305	0.017256	0.014608	5
GO:2000341	metabolic process	regulation of chemokine (C-X-C motif) ligand 2 production	5/804	20/18723	0.001305	0.017256	0.014608	5
GO:0032753	metabolic process	positive regulation of interleukin-4 production	5/804	25/18723	0.003743	0.040064	0.033917	5
GO:0060907	metabolic process	positive regulation of macrophage cytokine production	4/804	12/18723	0.001267	0.016905	0.014311	4
GO:0071639	metabolic process	positive regulation of monocyte chemotactic protein-1 production	4/804	16/18723	0.004061	0.042878	0.0363	4
GO:0002764	immune system process	immune response-regulating signaling pathway	61/804	468/18723	8.23E-15	3.79E-11	3.21E-11	61
GO:0002683	immune system process	negative regulation of immune system process	57/804	434/18723	4.66E-14	7.16E-11	6.06E-11	57
GO:0002443	immune system process	leukocyte mediated immunity	56/804	440/18723	2.96E-13	3.41E-10	2.89E-10	56
GO:0042110	immune system process	T cell activation	56/804	487/18723	1.85E-11	7.75E-09	6.56E-09	56
GO:0002460	immune system process	adaptive immune response based on somatic recombination of immune receptors built from immunoglobulin superfamily domains	47/804	356/18723	6.91E-12	3.18E-09	2.69E-09	47

GO:1903706	immune system process	regulation of hemopoiesis	47/804	367/18723	2.04E-11	7.81E-09	6.61E-09	47
GO:0002253	immune system process	activation of immune response	45/804	375/18723	4.74E-10	1.09E-07	9.23E-08	45
GO:0002449	immune system process	lymphocyte mediated immunity	44/804	350/18723	1.66E-10	4.76E-08	4.03E-08	44
GO:0002697	immune system process	regulation of immune effector process	43/804	339/18723	2.01E-10	5.45E-08	4.62E-08	43
GO:1903131	immune system process	mononuclear cell differentiation	43/804	426/18723	1.9E-07	1.15E-05	9.76E-06	43
GO:0002366	immune system process	leukocyte activation involved in immune response	42/804	275/18723	7.44E-13	6.85E-10	5.8E-10	42
GO:0002263	immune system process	cell activation involved in immune response	42/804	279/18723	1.22E-12	8.01E-10	6.78E-10	42
GO:0050900	immune system process	leukocyte migration	42/804	369/18723	8.81E-09	8.81E-07	7.46E-07	42
GO:0070661	immune system process	leukocyte proliferation	40/804	318/18723	1.12E-09	2.05E-07	1.74E-07	40
GO:0030099	immune system process	myeloid cell differentiation	40/804	381/18723	1.81E-07	1.11E-05	9.42E-06	40
GO:0002768	immune system process	immune response-regulating cell surface receptor signaling pathway	39/804	315/18723	2.85E-09	3.97E-07	3.36E-07	39
GO:0046651	immune system process	lymphocyte proliferation	38/804	288/18723	7.46E-10	1.59E-07	1.35E-07	38
GO:0032943	immune system process	mononuclear cell proliferation	38/804	291/18723	1.01E-09	2.01E-07	1.7E-07	38
GO:0002429	immune system process	immune response-activating cell surface receptor signaling pathway	36/804	291/18723	1.18E-08	1.11E-06	9.42E-07	36
GO:0002757	immune system process	immune response-activating signal transduction	36/804	291/18723	1.18E-08	1.11E-06	9.42E-07	36
GO:0030098	immune system process	lymphocyte differentiation	36/804	374/18723	5.6E-06	0.000185	0.000157	36
GO:1902105	immune system process	regulation of leukocyte differentiation	35/804	279/18723	1.32E-08	1.21E-06	1.03E-06	35
GO:0051251	immune system process	positive regulation of lymphocyte activation	35/804	362/18723	6.83E-06	0.00022	0.000186	35
GO:0070663	immune system process	regulation of leukocyte proliferation	33/804	245/18723	5.84E-09	6.4E-07	5.41E-07	33
GO:0042113	immune system process	B cell activation	33/804	334/18723	7.91E-06	0.000251	0.000213	33
GO:0097529	immune system process	myeloid leukocyte migration	32/804	220/18723	1.45E-09	2.56E-07	2.17E-07	32
GO:0050670	immune system process	regulation of lymphocyte proliferation	32/804	225/18723	2.56E-09	3.68E-07	3.12E-07	32
GO:0032944	immune system process	regulation of mononuclear cell proliferation	32/804	227/18723	3.2E-09	4.09E-07	3.46E-07	32
GO:0002440	immune system process	production of molecular mediator of immune response	32/804	308/18723	3.8E-06	0.000134	0.000113	32
GO:0002703	immune system process	regulation of leukocyte mediated immunity	30/804	226/18723	4E-08	3.02E-06	2.56E-06	30
GO:0002699	immune system process	positive regulation of immune effector process	30/804	235/18723	9.7E-08	6.76E-06	5.73E-06	30
GO:0002285	immune system process	lymphocyte activation involved in immune response	29/804	194/18723	4.5E-09	5.18E-07	4.39E-07	29
GO:0050777	immune system process	negative regulation of immune response	29/804	194/18723	4.5E-09	5.18E-07	4.39E-07	29
GO:0045637	immune system process	regulation of myeloid cell differentiation	29/804	210/18723	2.78E-08	2.37E-06	2.01E-06	29
GO:0045088	immune system process	regulation of innate immune response	28/804	218/18723	2.27E-07	1.32E-05	1.12E-05	28
GO:0019882	immune system process	antigen processing and presentation	27/804	106/18723	3.73E-14	7.16E-11	6.06E-11	27
GO:0016064	immune system process	immunoglobulin mediated immune response	27/804	207/18723	2.72E-07	1.53E-05	1.29E-05	27
GO:0019724	immune system process	B cell mediated immunity	27/804	210/18723	3.65E-07	1.84E-05	1.56E-05	27
GO:0006959	immune system process	humoral immune response	27/804	317/18723	0.000584	0.009472	0.008019	27
GO:0071674	immune system process	mononuclear cell migration	26/804	196/18723	3.23E-07	1.79E-05	1.52E-05	26
GO:0002685	immune system process	regulation of leukocyte migration	26/804	210/18723	1.24E-06	5.19E-05	4.4E-05	26
GO:0050851	immune system process	antigen receptor-mediated signaling pathway	26/804	240/18723	1.45E-05	0.000422	0.000357	26
GO:0050870	immune system process	positive regulation of T cell activation	25/804	216/18723	6.69E-06	0.000218	0.000185	25
GO:0030217	immune system process	T cell differentiation	25/804	257/18723	0.000124	0.002474	0.002095	25
GO:0002819	immune system process	regulation of adaptive immune response	24/804	183/18723	1.12E-06	4.74E-05	4.02E-05	24

GO:0042098	immune system process	T cell proliferation	24/804	199/18723	5.01E-06	0.000167	0.000141	24
GO:0002573	immune system process	myeloid leukocyte differentiation	24/804	208/18723	1.07E-05	0.000325	0.000275	24
GO:0002822	immune system process	regulation of adaptive immune response based on somatic recombination of immune receptors built from immunoglobulin superfamily domains	22/804	168/18723	3.2E-06	0.000115	9.77E-05	22
GO:0002698	immune system process	negative regulation of immune effector process	19/804	110/18723	2.08E-07	1.23E-05	1.04E-05	19
GO:0002700	immune system process	regulation of production of molecular mediator of immune response	19/804	164/18723	8.2E-05	0.001806	0.001529	19
GO:0002381	immune system process	immunoglobulin production involved in immunoglobulin-mediated immune response	16/804	70/18723	3.25E-08	2.54E-06	2.15E-06	16
GO:0002824	immune system process	positive regulation of adaptive immune response based on somatic recombination of immune receptors built from immunoglobulin superfamily domains	15/804	107/18723	5.18E-05	0.00127	0.001076	15
GO:0002821	immune system process	positive regulation of adaptive immune response	15/804	112/18723	8.84E-05	0.0019	0.001609	15
GO:0002702	immune system process	positive regulation of production of molecular mediator of immune response	14/804	117/18723	0.000493	0.008401	0.007112	14
GO:0045089	immune system process	positive regulation of innate immune response	14/804	131/18723	0.001512	0.019498	0.016506	14
GO:0002718	immune system process	regulation of cytokine production involved in immune response	12/804	96/18723	0.000825	0.012404	0.010501	12
GO:0002367	immune system process	cytokine production involved in immune response	12/804	98/18723	0.000992	0.014135	0.011967	12
GO:0045824	immune system process	negative regulation of innate immune response	11/804	71/18723	0.000208	0.003939	0.003335	11
GO:0002820	immune system process	negative regulation of adaptive immune response	9/804	59/18723	0.000868	0.012615	0.01068	9
GO:0002720	immune system process	positive regulation of cytokine production involved in immune response	9/804	65/18723	0.001756	0.021999	0.018624	9
GO:0002823	immune system process	negative regulation of adaptive immune response based on somatic recombination of immune receptors built from immunoglobulin superfamily domains	8/804	54/18723	0.002016	0.024294	0.020567	8
GO:0140374	immune system process	antiviral innate immune response	4/804	15/18723	0.003152	0.034545	0.029246	4
GO:0002377	immune system process	immunoglobulin production	22/804	216/18723	0.000161	0.003128	0.002648	22
GO:0050867	immune system process	positive regulation of cell activation	43/804	420/18723	1.28E-07	8.54E-06	7.23E-06	43
GO:0002696	immune system process	positive regulation of leukocyte activation	41/804	409/18723	4.4E-07	2.09E-05	1.77E-05	41
GO:0050863	immune system process	regulation of T cell activation	39/804	329/18723	9.76E-09	9.56E-07	8.09E-07	39
GO:0050866	immune system process	negative regulation of cell activation	34/804	210/18723	2.27E-11	8.04E-09	6.8E-09	34
GO:0002274	immune system process	myeloid leukocyte activation	31/804	223/18723	7.9E-09	8.08E-07	6.84E-07	31
GO:0002695	immune system process	negative regulation of leukocyte activation	30/804	187/18723	4.3E-10	1.04E-07	8.82E-08	30
GO:0051250	immune system process	negative regulation of lymphocyte activation	26/804	157/18723	3.14E-09	4.09E-07	3.46E-07	26
GO:0050864	immune system process	regulation of B cell activation	22/804	198/18723	4.42E-05	0.001118	0.000947	22
GO:0042116	immune system process	macrophage activation	20/804	106/18723	2.13E-08	1.85E-06	1.57E-06	20
GO:0046631	immune system process	alpha-beta T cell activation	20/804	156/18723	1.22E-05	0.000365	0.000309	20
GO:0050868	immune system process	negative regulation of T cell activation	18/804	122/18723	4.58E-06	0.000154	0.000131	18
GO:0046634	immune system process	regulation of alpha-beta T cell activation	16/804	104/18723	8.9E-06	0.000275	0.000233	16
GO:0002275	immune system process	myeloid cell activation involved in immune response	15/804	91/18723	7.13E-06	0.000228	0.000193	15
GO:0002286	immune system process	T cell activation involved in immune response	15/804	114/18723	0.000108	0.002238	0.001894	15
GO:0002312	immune system process	B cell activation involved in immune response	13/804	82/18723	4.41E-05	0.001118	0.000947	13
GO:0001774	immune system process	microglial cell activation	11/804	47/18723	3.61E-06	0.000128	0.000108	11
GO:0046635	immune system process	positive regulation of alpha-beta T cell activation	10/804	67/18723	0.000543	0.009063	0.007673	10
GO:0030101	immune system process	natural killer cell activation	10/804	88/18723	0.004419	0.046227	0.039135	10
GO:0043030	immune system process	regulation of macrophage activation	9/804	61/18723	0.001109	0.015381	0.013021	9
GO:0050869	immune system process	negative regulation of B cell activation	8/804	34/18723	7.5E-05	0.001676	0.001418	8
GO:0042119	immune system process	neutrophil activation	8/804	36/18723	0.000116	0.002349	0.001988	8
GO:0036230	immune system process	granulocyte activation	8/804	43/18723	0.000426	0.007371	0.00624	8

GO:0032814	immune system process	regulation of natural killer cell activation	7/804	35/18723	0.000615	0.009901	0.008382	7
GO:0033622	immune system process	integrin activation	6/804	25/18723	0.00054	0.009063	0.007673	6
GO:0002291	immune system process	T cell activation via T cell receptor contact with antigen bound to MHC molecule on antigen	5/804	10/18723	3.03E-05	0.000807	0.000684	5
GO:1902563	immune system process	regulation of neutrophil activation	5/804	12/18723	8.88E-05	0.0019	0.001609	5
GO:0002283	immune system process	neutrophil activation involved in immune response	5/804	18/18723	0.000774	0.011763	0.009958	5
GO:0002281	immune system process	macrophage activation involved in immune response	5/804	19/18723	0.001014	0.014299	0.012105	5
GO:0001909	immune system process	leukocyte mediated cytotoxicity	18/804	124/18723	5.79E-06	0.00019	0.000161	18
GO:0002705	immune system process	positive regulation of leukocyte mediated immunity	18/804	134/18723	1.73E-05	0.000495	0.000419	18
GO:0070665	immune system process	positive regulation of leukocyte proliferation	18/804	150/18723	7.93E-05	0.001763	0.001492	18
GO:0022411	cellular component organiza	cellular component disassembly	39/804	443/18723	1.88E-05	0.000527	0.000446	39
GO:0030198	cellular component organiza	extracellular matrix organization	36/804	301/18723	2.87E-08	2.4E-06	2.04E-06	36
GO:0043062	cellular component organiza	extracellular structure organization	36/804	302/18723	3.13E-08	2.48E-06	2.1E-06	36
GO:0045229	cellular component organiza	external encapsulating structure organization	36/804	304/18723	3.71E-08	2.85E-06	2.41E-06	36
GO:0048285	cellular component organiza	organelle fission	36/804	488/18723	0.001173	0.01612	0.013647	36
GO:0006325	cellular component organiza	chromatin organization	33/804	409/18723	0.000405	0.007177	0.006076	33
GO:0000280	cellular component organiza	nuclear division	33/804	439/18723	0.001359	0.017875	0.015132	33
GO:0022613	cellular component organiza	ribonucleoprotein complex biogenesis	33/804	463/18723	0.003175	0.034719	0.029392	33
GO:0007059	cellular component organiza	chromosome segregation	30/804	346/18723	0.000219	0.004081	0.003455	30
GO:0140014	cellular component organiza	mitotic nuclear division	29/804	287/18723	1.82E-05	0.000514	0.000435	29
GO:0140694	cellular component organiza	non-membrane-bounded organelle assembly	28/804	367/18723	0.002457	0.028565	0.024183	28
GO:0071824	cellular component organiza	protein-DNA complex subunit organization	26/804	241/18723	1.57E-05	0.00045	0.000381	26
GO:0071103	cellular component organiza	DNA conformation change	26/804	290/18723	0.000337	0.006133	0.005192	26
GO:0098813	cellular component organiza	nuclear chromosome segregation	25/804	281/18723	0.000488	0.008344	0.007064	25
GO:0006338	cellular component organiza	chromatin remodeling	24/804	255/18723	0.000277	0.005072	0.004294	24
GO:0061448	cellular component organiza	connective tissue development	25/804	252/18723	9.08E-05	0.001925	0.00163	25
GO:0034728	cellular component organiza	nucleosome organization	22/804	167/18723	2.89E-06	0.000107	9.1E-05	22
GO:0000819	cellular component organiza	sister chromatid segregation	21/804	202/18723	0.000169	0.003273	0.002771	21
GO:0000070	cellular component organiza	mitotic sister chromatid segregation	19/804	168/18723	0.000113	0.002309	0.001955	19
GO:0031503	cellular component organiza	protein-containing complex localization	19/804	220/18723	0.003085	0.034408	0.02913	19
GO:0007159	cell adhesion	leukocyte cell-cell adhesion	44/804	371/18723	1.08E-09	2.05E-07	1.74E-07	44
GO:0022407	cell adhesion	regulation of cell-cell adhesion	40/804	448/18723	1.06E-05	0.000324	0.000275	40
GO:1903037	cell adhesion	regulation of leukocyte cell-cell adhesion	37/804	336/18723	1.59E-07	1.01E-05	8.58E-06	37
GO:0045785	cell adhesion	positive regulation of cell adhesion	37/804	437/18723	6.97E-05	0.001572	0.001331	37
GO:0007162	cell adhesion	negative regulation of cell adhesion	29/804	303/18723	4.96E-05	0.001226	0.001038	29
GO:1903039	cell adhesion	positive regulation of leukocyte cell-cell adhesion	27/804	239/18723	4.59E-06	0.000154	0.000131	27
GO:0022409	cell adhesion	positive regulation of cell-cell adhesion	27/804	284/18723	9.98E-05	0.002106	0.001783	27
GO:0022408	cell adhesion	negative regulation of cell-cell adhesion	20/804	196/18723	0.000307	0.005605	0.004745	20
GO:1903038	cell adhesion	negative regulation of leukocyte cell-cell adhesion	18/804	141/18723	3.48E-05	0.000894	0.000757	18
GO:0033627	cell adhesion	cell adhesion mediated by integrin	12/804	72/18723	5.19E-05	0.00127	0.001076	12
GO:0033628	cell adhesion	regulation of cell adhesion mediated by integrin	7/804	48/18723	0.004157	0.043589	0.036902	7

GO:0033631	cell adhesion	cell-cell adhesion mediated by integrin	4/804	16/18723	0.004061	0.042878	0.0363	4

Table S3. Top 200 GO biological processes in cohort with poor prognosis

ID	Biological processes	Description	GeneRatio	BgRatio	pvalue	p.adjust	qvalue	Count
GO:0016032	respose to stimulus	viral process	49/982	415/18723	9.27E-08	6.90E-06	5.90E-06	49
GO:0019058	respose to stimulus	viral life cycle	41/982	317/18723	8.89E-08	6.72E-06	5.74E-06	41
GO:0002831	respose to stimulus	regulation of response to biotic stimulus	48/982	327/18723	9.67E-11	3.90E-08	3.33E-08	48
GO:0009615	respose to stimulus	response to virus	47/982	367/18723	1.40E-08	1.85E-06	1.58E-06	47
GO:0002237	respose to stimulus	response to molecule of bacterial origin	45/982	363/18723	7.52E-08	5.93E-06	5.06E-06	45
GO:0032103	respose to stimulus	positive regulation of response to external stimulus	45/982	427/18723	6.87E-06	0.000266	0.000227	45
GO:0032496	respose to stimulus	response to lipopolysaccharide	42/982	343/18723	2.86E-07	1.82E-05	1.56E-05	42
GO:0060326	respose to stimulus	cell chemotaxis	40/982	310/18723	1.36E-07	9.82E-06	8.39E-06	40
GO:0032102	respose to stimulus	negative regulation of response to external stimulus	40/982	420/18723	0.000203	0.004387	0.003748	40
GO:0043410	respose to stimulus	positive regulation of MAPK cascade	40/982	480/18723	0.002661	0.033602	0.028702	40
GO:0051607	respose to stimulus	defense response to virus	38/982	265/18723	1.67E-08	1.85E-06	1.58E-06	38
GO:0140546	respose to stimulus	defense response to symbiont	38/982	265/18723	1.67E-08	1.85E-06	1.58E-06	38
GO:0042060	respose to stimulus	wound healing	37/982	422/18723	0.001591	0.02353	0.020099	37
GO:0071216	respose to stimulus	cellular response to biotic stimulus	35/982	246/18723	7.46E-08	5.93E-06	5.06E-06	35
GO:0050727	respose to stimulus	regulation of inflammatory response	34/982	386/18723	0.002236	0.030208	0.025802	34
GO:0030595	respose to stimulus	leukocyte chemotaxis	33/982	230/18723	1.43E-07	1.02E-05	8.70E-06	33
GO:0071219	respose to stimulus	cellular response to molecule of bacterial origin	32/982	221/18723	1.78E-07	1.23E-05	1.05E-05	32
GO:0030111	respose to stimulus	regulation of Wnt signaling pathway	31/982	328/18723	0.001144	0.018088	0.01545	31
GO:0071222	respose to stimulus	cellular response to lipopolysaccharide	30/982	209/18723	5.19E-07	2.95E-05	2.52E-05	30
GO:0070372	respose to stimulus	regulation of ERK1 and ERK2 cascade	30/982	309/18723	0.00089	0.014547	0.012425	30
GO:0070371	respose to stimulus	ERK1 and ERK2 cascade	30/982	330/18723	0.002471	0.032042	0.027369	30
GO:0031348	respose to stimulus	negative regulation of defense response	29/982	258/18723	9.33E-05	0.002287	0.001954	29
GO:0031349	respose to stimulus	positive regulation of defense response	28/982	278/18723	0.00074	0.012433	0.010619	28
GO:0034341	respose to stimulus	response to interferon-gamma	27/982	141/18723	4.20E-09	8.46E-07	7.23E-07	27
GO:0060759	respose to stimulus	regulation of response to cytokine stimulus	23/982	162/18723	1.29E-05	0.000457	0.00039	23
GO:0002833	respose to stimulus	positive regulation of response to biotic stimulus	23/982	168/18723	2.35E-05	0.00073	0.000624	23
GO:0071230	respose to stimulus	cellular response to amino acid stimulus	15/982	71/18723	3.24E-06	0.000147	0.000125	15
GO:0002832	respose to stimulus	negative regulation of response to biotic stimulus	15/982	108/18723	0.000508	0.009033	0.007716	15
GO:0009595	respose to stimulus	detection of biotic stimulus	11/982	38/18723	2.54E-06	0.000121	0.000103	11
GO:0060760	respose to stimulus	positive regulation of response to cytokine stimulus	11/982	57/18723	0.000156	0.003484	0.002976	11
GO:0098581	respose to stimulus	detection of external biotic stimulus	10/982	25/18723	2.39E-07	1.56E-05	1.33E-05	10
GO:0036006	respose to stimulus	cellular response to macrophage colony-stimulating factor stimulus	5/982	13/18723	0.000355	0.006759	0.005773	5
GO:0050792	Response to stimulus	regulation of viral process	26/982	164/18723	4.25E-07	2.47E-05	2.11E-05	26
GO:0050920	Response to stimulus	regulation of chemotaxis	25/982	223/18723	0.000288	0.00585	0.004997	25
GO:0034612	Response to stimulus	response to tumor necrosis factor	25/982	253/18723	0.001807	0.026317	0.022479	25
GO:1903900	Response to stimulus	regulation of viral life cycle	24/982	148/18723	7.69E-07	4.18E-05	3.57E-05	24
GO:0072593	Response to stimulus	reactive oxygen species metabolic process	23/982	239/18723	0.003753	0.042868	0.036616	23
GO:0033674	metabolic process	positive regulation of kinase activity	43/982	467/18723	0.00025	0.005188	0.004431	43
GO:0002181	metabolic process	cytoplasmic translation	40/982	148/18723	2.91E-18	1.41E-14	1.20E-14	40

GO:0052547	metabolic process	regulation of peptidase activity	38/982	461/18723	0.004022	0.045028	0.038461	38
GO:0018108	metabolic process	peptidyl-tyrosine phosphorylation	36/982	375/18723	0.000356	0.006759	0.005773	36
GO:0018212	metabolic process	peptidyl-tyrosine modification	36/982	378/18723	0.000414	0.007645	0.00653	36
GO:0045860	metabolic process	positive regulation of protein kinase activity	34/982	386/18723	0.002236	0.030208	0.025802	34
GO:0045862	metabolic process	positive regulation of proteolysis	33/982	372/18723	0.0023	0.030957	0.026443	33
GO:0018205	metabolic process	peptidyl-lysine modification	33/982	376/18723	0.002724	0.034223	0.029232	33
GO:0051052	metabolic process	regulation of DNA metabolic process	31/982	359/18723	0.004568	0.04999	0.0427	31
GO:0071706	metabolic process	tumor necrosis factor superfamily cytokine production	27/982	186/18723	1.52E-06	7.85E-05	6.70E-05	27
GO:1903555	metabolic process	regulation of tumor necrosis factor superfamily cytokine production	27/982	186/18723	1.52E-06	7.85E-05	6.70E-05	27
GO:0050730	metabolic process	regulation of peptidyl-tyrosine phosphorylation	27/982	264/18723	0.000723	0.012189	0.010411	27
GO:0001819	metabolic process	positive regulation of cytokine production	58/982	467/18723	9.09E-10	2.59E-07	2.21E-07	58
GO:0019221	metabolic process	cytokine-mediated signaling pathway	58/982	472/18723	1.36E-09	3.38E-07	2.89E-07	58
GO:0001818	metabolic process	negative regulation of cytokine production	31/982	357/18723	0.004211	0.046612	0.039814	31
GO:0032640	metabolic process	tumor necrosis factor production	26/982	181/18723	2.89E-06	0.000133	0.000114	26
GO:0032680	metabolic process	regulation of tumor necrosis factor production	26/982	181/18723	2.89E-06	0.000133	0.000114	26
GO:1903557	metabolic process	positive regulation of tumor necrosis factor superfamily cytokine production	22/982	107/18723	2.96E-08	2.98E-06	2.55E-06	22
GO:0032760	metabolic process	positive regulation of tumor necrosis factor production	21/982	103/18723	7.08E-08	5.91E-06	5.05E-06	21
GO:0032609	metabolic process	interferon-gamma production	21/982	112/18723	3.17E-07	1.94E-05	1.66E-05	21
GO:0032649	metabolic process	regulation of interferon-gamma production	21/982	112/18723	3.17E-07	1.94E-05	1.66E-05	21
GO:0032479	metabolic process	regulation of type I interferon production	18/982	95/18723	1.86E-06	9.28E-05	7.93E-05	18
GO:0032606	metabolic process	type I interferon production	18/982	95/18723	1.86E-06	9.28E-05	7.93E-05	18
GO:0032635	metabolic process	interleukin-6 production	18/982	165/18723	0.002618	0.033586	0.028688	18
GO:0032675	metabolic process	regulation of interleukin-6 production	18/982	165/18723	0.002618	0.033586	0.028688	18
GO:0032642	metabolic process	regulation of chemokine production	17/982	98/18723	1.23E-05	0.000438	0.000374	17
GO:0032602	metabolic process	chemokine production	17/982	99/18723	1.42E-05	0.000493	0.000421	17
GO:0032729	metabolic process	positive regulation of interferon-gamma production	15/982	72/18723	3.90E-06	0.000173	0.000148	15
GO:0032722	metabolic process	positive regulation of chemokine production	14/982	71/18723	1.59E-05	0.000524	0.000448	14
GO:0032608	metabolic process	interferon-beta production	13/982	56/18723	4.83E-06	0.000201	0.000172	13
GO:0032648	metabolic process	regulation of interferon-beta production	13/982	56/18723	4.83E-06	0.000201	0.000172	13
GO:0032481	metabolic process	positive regulation of type I interferon production	13/982	58/18723	7.32E-06	0.000279	0.000238	13
GO:0032613	metabolic process	interleukin-10 production	13/982	62/18723	1.59E-05	0.000524	0.000448	13
GO:0032653	metabolic process	regulation of interleukin-10 production	13/982	62/18723	1.59E-05	0.000524	0.000448	13
GO:0032677	metabolic process	regulation of interleukin-8 production	13/982	102/18723	0.002584	0.033331	0.02847	13
GO:0032637	metabolic process	interleukin-8 production	13/982	103/18723	0.002819	0.035075	0.029959	13
GO:0032728	metabolic process	positive regulation of interferon-beta production	11/982	39/18723	3.37E-06	0.000151	0.000129	11
GO:0032623	metabolic process	interleukin-2 production	11/982	62/18723	0.000339	0.006557	0.005601	11
GO:0032663	metabolic process	regulation of interleukin-2 production	11/982	62/18723	0.000339	0.006557	0.005601	11
GO:0032757	metabolic process	positive regulation of interleukin-8 production	10/982	62/18723	0.001347	0.020755	0.017728	10
GO:0032689	metabolic process	negative regulation of interferon-gamma production	9/982	42/18723	0.000269	0.005498	0.004696	9
GO:0032743	metabolic process	positive regulation of interleukin-2 production	7/982	34/18723	0.001654	0.024174	0.020648	7

GO:0032607	metabolic process	interferon-alpha production	6/982	28/18723	0.002857	0.035075	0.029959	6
GO:0032647	metabolic process	regulation of interferon-alpha production	6/982	28/18723	0.002857	0.035075	0.029959	6
GO:0061082	metabolic process	myeloid leukocyte cytokine production	6/982	28/18723	0.002857	0.035075	0.029959	6
GO:0072567	metabolic process	chemokine (C-X-C motif) ligand 2 production	5/982	20/18723	0.003147	0.037404	0.031949	5
GO:2000341	metabolic process	regulation of chemokine (C-X-C motif) ligand 2 production	5/982	20/18723	0.003147	0.037404	0.031949	5
GO:0060907	metabolic process	positive regulation of macrophage cytokine production	4/982	12/18723	0.002652	0.033586	0.028688	4
GO:0002764	immune system process	immune response-regulating signaling pathway	61/982	468/18723	4.57E-11	3.62E-08	3.09E-08	61
GO:0002683	immune system process	negative regulation of immune system process	59/982	434/18723	1.72E-11	2.07E-08	1.77E-08	59
GO:0002443	immune system process	leukocyte mediated immunity	57/982	440/18723	2.54E-10	8.79E-08	7.51E-08	57
GO:1903706	immune system process	regulation of hemopoiesis	52/982	367/18723	6.04E-11	3.62E-08	3.09E-08	52
GO:1903131	immune system process	mononuclear cell differentiation	50/982	426/18723	8.28E-08	6.36E-06	5.43E-06	50
GO:0002253	immune system process	activation of immune response	48/982	375/18723	9.95E-09	1.46E-06	1.25E-06	48
GO:0030099	immune system process	myeloid cell differentiation	48/982	381/18723	1.66E-08	1.85E-06	1.58E-06	48
GO:0050900	immune system process	leukocyte migration	46/982	369/18723	4.55E-08	4.09E-06	3.49E-06	46
GO:0002460	immune system process	adaptive immune response based on somatic recombination of immune receptors built from immunoglobulin superfamily domains	45/982	356/18723	4.21E-08	3.91E-06	3.34E-06	45
GO:0070661	immune system process	leukocyte proliferation	43/982	318/18723	1.16E-08	1.60E-06	1.37E-06	43
GO:0002697	immune system process	regulation of immune effector process	43/982	339/18723	7.60E-08	5.93E-06	5.06E-06	43
GO:0002449	immune system process	lymphocyte mediated immunity	43/982	350/18723	1.88E-07	1.28E-05	1.10E-05	43
GO:0030098	immune system process	lymphocyte differentiation	42/982	374/18723	2.84E-06	0.000133	0.000114	42
GO:0002263	immune system process	cell activation involved in immune response	41/982	279/18723	2.19E-09	4.82E-07	4.12E-07	41
GO:0002768	immune system process	immune response-regulating cell surface receptor signaling pathway	41/982	315/18723	7.46E-08	5.93E-06	5.06E-06	41
GO:0002366	immune system process	leukocyte activation involved in immune response	40/982	275/18723	4.65E-09	9.00E-07	7.69E-07	40
GO:1902105	immune system process	regulation of leukocyte differentiation	40/982	279/18723	7.08E-09	1.18E-06	1.01E-06	40
GO:0046651	immune system process	lymphocyte proliferation	40/982	288/18723	1.76E-08	1.89E-06	1.62E-06	40
GO:0032943	immune system process	mononuclear cell proliferation	40/982	291/18723	2.36E-08	2.48E-06	2.12E-06	40
GO:0002429	immune system process	immune response-activating cell surface receptor signaling pathway	38/982	291/18723	2.05E-07	1.36E-05	1.16E-05	38
GO:0002757	immune system process	immune response-activating signal transduction	38/982	291/18723	2.05E-07	1.36E-05	1.16E-05	38
GO:0070663	immune system process	regulation of leukocyte proliferation	37/982	245/18723	6.29E-09	1.17E-06	9.99E-07	37
GO:0050670	immune system process	regulation of lymphocyte proliferation	35/982	225/18723	7.38E-09	1.19E-06	1.02E-06	35
GO:0032944	immune system process	regulation of mononuclear cell proliferation	35/982	227/18723	9.33E-09	1.41E-06	1.21E-06	35
GO:0045088	immune system process	regulation of innate immune response	34/982	218/18723	1.13E-08	1.60E-06	1.37E-06	34
GO:0097529	immune system process	myeloid leukocyte migration	34/982	220/18723	1.43E-08	1.85E-06	1.58E-06	34
GO:0045637	immune system process	regulation of myeloid cell differentiation	33/982	210/18723	1.53E-08	1.85E-06	1.58E-06	33
GO:0002703	immune system process	regulation of leukocyte mediated immunity	32/982	226/18723	3.00E-07	1.88E-05	1.61E-05	32
GO:0002440	immune system process	production of molecular mediator of immune response	32/982	308/18723	0.000184	0.004061	0.003469	32
GO:0002699	immune system process	positive regulation of immune effector process	30/982	235/18723	6.09E-06	0.000242	0.000206	30
GO:0050777	immune system process	negative regulation of immune response	29/982	194/18723	3.37E-07	2.04E-05	1.74E-05	29
GO:0071674	immune system process	mononuclear cell migration	29/982	196/18723	4.20E-07	2.47E-05	2.11E-05	29
GO:0002573	immune system process	myeloid leukocyte differentiation	28/982	208/18723	4.44E-06	0.000189	0.000161	28
GO:0002685	immune system process	regulation of leukocyte migration	28/982	210/18723	5.35E-06	0.000216	0.000184	28

GO:0030217	immune system process	T cell differentiation	28/982	257/18723	0.000208	0.004467	0.003816	28
GO:0019882	immune system process	antigen processing and presentation	27/982	106/18723	4.20E-12	6.78E-09	5.79E-09	27
GO:0050851	immune system process	antigen receptor-mediated signaling pathway	27/982	240/18723	0.000158	0.003511	0.002999	27
GO:0042110	immune system process	T cell activation	65/982	487/18723	3.58E-12	6.78E-09	5.79E-09	65
GO:0002696	immune system process	positive regulation of leukocyte activation	47/982	409/18723	3.77E-07	2.25E-05	1.93E-05	47
GO:0050863	immune system process	regulation of T cell activation	43/982	329/18723	3.18E-08	3.14E-06	2.68E-06	43
GO:0051251	immune system process	positive regulation of lymphocyte activation	40/982	362/18723	7.20E-06	0.000276	0.000236	40
GO:0042113	immune system process	B cell activation	35/982	334/18723	7.89E-05	0.00201	0.001717	35
GO:0002274	immune system process	myeloid leukocyte activation	33/982	223/18723	6.79E-08	5.76E-06	4.92E-06	33
GO:0002695	immune system process	negative regulation of leukocyte activation	32/982	187/18723	2.99E-09	6.30E-07	5.38E-07	32
GO:0050870	immune system process	positive regulation of T cell activation	30/982	216/18723	1.06E-06	5.59E-05	4.77E-05	30
GO:0051250	immune system process	negative regulation of lymphocyte activation	27/982	157/18723	4.56E-08	4.09E-06	3.49E-06	27
GO:0050867	immune system process	positive regulation of cell activation	50/982	420/18723	5.28E-08	4.64E-06	3.96E-06	50
GO:0050866	immune system process	negative regulation of cell activation	36/982	210/18723	2.95E-10	9.52E-08	8.13E-08	36
GO:0002285	immune system process	lymphocyte activation involved in immune response	26/982	194/18723	1.05E-05	0.000383	0.000327	26
GO:0050864	immune system process	regulation of B cell activation	24/982	198/18723	0.000115	0.002715	0.002319	24
GO:0042116	immune system process	macrophage activation	19/982	106/18723	2.33E-06	0.000113	9.61E-05	19
GO:0046631	immune system process	alpha-beta T cell activation	19/982	156/18723	0.000538	0.009425	0.00805	19
GO:0050868	immune system process	negative regulation of T cell activation	18/982	122/18723	6.60E-05	0.001754	0.001498	18
GO:0002275	immune system process	myeloid cell activation involved in immune response	16/982	91/18723	1.85E-05	0.000595	0.000509	16
GO:0002286	immune system process	T cell activation involved in immune response	15/982	114/18723	0.000903	0.014658	0.01252	15
GO:0046634	immune system process	regulation of alpha-beta T cell activation	14/982	104/18723	0.001055	0.016972	0.014497	14
GO:0043030	immune system process	regulation of macrophage activation	11/982	61/18723	0.000292	0.005894	0.005034	11
GO:0036230	immune system process	granulocyte activation	10/982	43/18723	5.92E-05	0.00161	0.001375	10
GO:0046635	immune system process	positive regulation of alpha-beta T cell activation	10/982	67/18723	0.002455	0.032042	0.027369	10
GO:0042119	immune system process	neutrophil activation	9/982	36/18723	7.55E-05	0.001942	0.001659	9
GO:0001774	immune system process	microglial cell activation	9/982	47/18723	0.000651	0.011083	0.009467	9
GO:0050869	immune system process	negative regulation of B cell activation	8/982	34/18723	0.000298	0.005959	0.00509	8
GO:0032814	immune system process	regulation of natural killer cell activation	7/982	35/18723	0.001976	0.027302	0.023321	7
GO:0016064	immune system process	immunoglobulin mediated immune response	26/982	207/18723	3.30E-05	0.000981	0.000838	26
GO:0002819	immune system process	regulation of adaptive immune response	25/982	183/18723	1.10E-05	0.000399	0.000341	25
GO:0002822	immune system process	regulation of adaptive immune response based on somatic recombination of immune receptors built from immunoglobulin superfamily domains	22/982	168/18723	6.96E-05	0.00181	0.001546	22
GO:0002700	immune system process	regulation of production of molecular mediator of immune response	21/982	164/18723	0.000139	0.003147	0.002688	21
GO:0002698	immune system process	negative regulation of immune effector process	17/982	110/18723	5.76E-05	0.001592	0.00136	17
GO:0045089	immune system process	positive regulation of innate immune response	17/982	131/18723	0.000498	0.008892	0.007595	17
GO:0002821	immune system process	positive regulation of adaptive immune response	15/982	112/18723	0.00075	0.012494	0.010672	15
GO:0002718	immune system process	regulation of cytokine production involved in immune response	14/982	96/18723	0.000468	0.008414	0.007187	14
GO:0002367	immune system process	cytokine production involved in immune response	14/982	98/18723	0.000579	0.010039	0.008575	14
GO:0002824	immune system process	positive regulation of adaptive immune response based on somatic recombination of immune receptors built from immunoglobulin superfamily domains	14/982	107/18723	0.001396	0.021159	0.018073	14
GO:0002702	immune system process	positive regulation of production of molecular mediator of immune response	14/982	117/18723	0.00326	0.038552	0.032929	14

GO:0002381	immune system process	immunoglobulin production involved in immunoglobulin-mediated immune response	13/982	70/18723	6.14E-05	0.001651	0.00141	13
GO:0045824	immune system process	negative regulation of innate immune response	13/982	71/18723	7.17E-05	0.001853	0.001583	13
GO:0002720	immune system process	positive regulation of cytokine production involved in immune response	10/982	65/18723	0.001947	0.027064	0.023117	10
GO:0140374	immune system process	antiviral innate immune response	6/982	15/18723	6.82E-05	0.001782	0.001522	6
GO:0002283	immune system process	neutrophil activation involved in immune response	6/982	18/18723	0.000221	0.004682	0.003999	6
GO:0033622	immune system process	integrin activation	6/982	25/18723	0.001536	0.022792	0.019468	6
GO:0002291	immune system process	T cell activation via T cell receptor contact with antigen bound to MHC molecule on antigen presenting cell	5/982	10/18723	7.94E-05	0.00201	0.001717	5
GO:1902563	immune system process	regulation of neutrophil activation	5/982	12/18723	0.000228	0.004781	0.004084	5
GO:0061081	immune system process	positive regulation of myeloid leukocyte cytokine production involved in immune	5/982	21/18723	0.003955	0.044694	0.038176	5
GO:0046643	immune system process	regulation of gamma-delta T cell activation	4/982	11/18723	0.001844	0.026317	0.022479	4
GO:0042098	Immune system process	T cell proliferation	26/982	199/18723	1.65E-05	0.000536	0.000458	26
GO:0019724	Immune system process	B cell mediated immunity	26/982	210/18723	4.24E-05	0.001213	0.001036	26
GO:0042129	Immune system process	regulation of T cell proliferation	24/982	171/18723	1.03E-05	0.000381	0.000325	24
GO:0070665	Immune system process	positive regulation of leukocyte proliferation	22/982	150/18723	1.17E-05	0.000421	0.00036	22
GO:0022411	cellular component organiza	cellular component disassembly	48/982	443/18723	1.57E-06	7.99E-05	6.82E-05	48
GO:0030198	cellular component organiza	extracellular matrix organization	46/982	301/18723	6.01E-11	3.62E-08	3.09E-08	46
GO:0043062	cellular component organiza	extracellular structure organization	46/982	302/18723	6.74E-11	3.62E-08	3.09E-08	46
GO:0045229	cellular component organiza	external encapsulating structure organization	46/982	304/18723	8.46E-11	3.90E-08	3.33E-08	46
GO:0022613	cellular component organiza	ribonucleoprotein complex biogenesis	38/982	463/18723	0.004319	0.04748	0.040556	38
GO:0071824	cellular component organiza	protein-DNA complex subunit organization	24/982	241/18723	0.00199	0.027425	0.023425	24
GO:0034728	cellular component organiza	nucleosome organization	20/982	167/18723	0.000484	0.008679	0.007413	20
GO:0030199	cellular component organiza	collagen fibril organization	17/982	61/18723	9.04E-09	1.41E-06	1.20E-06	17
GO:2000249	cellular component organiza	regulation of actin cytoskeleton reorganization	7/982	39/18723	0.003767	0.042868	0.036616	7
GO:1903054	cellular component organiza	negative regulation of extracellular matrix organization	4/982	13/18723	0.003674	0.042307	0.036137	4
GO:0140014	Cellular component organiz	mitotic nuclear division	27/982	287/18723	0.002464	0.032042	0.027369	27
GO:0061448	Cellular component organiz	connective tissue development	26/982	252/18723	0.000789	0.013032	0.011132	26
GO:0007159	cell adhesion	leukocyte cell-cell adhesion	48/982	371/18723	7.02E-09	1.18E-06	1.01E-06	48
GO:0045785	cell adhesion	positive regulation of cell adhesion	47/982	437/18723	2.47E-06	0.000118	0.000101	47
GO:0022407	cell adhesion	regulation of cell-cell adhesion	47/982	448/18723	4.87E-06	0.000201	0.000172	47
GO:1903037	cell adhesion	regulation of leukocyte cell-cell adhesion	41/982	336/18723	4.34E-07	2.50E-05	2.14E-05	41
GO:0031589	cell adhesion	cell-substrate adhesion	40/982	363/18723	7.70E-06	0.000291	0.000248	40
GO:0022409	cell adhesion	positive regulation of cell-cell adhesion	33/982	284/18723	1.60E-05	0.000524	0.000448	33
GO:0007162	cell adhesion	negative regulation of cell adhesion	33/982	303/18723	5.93E-05	0.00161	0.001375	33
GO:1903039	cell adhesion	positive regulation of leukocyte cell-cell adhesion	32/982	239/18723	1.06E-06	5.59E-05	4.77E-05	32
GO:0007160	cell adhesion	cell-matrix adhesion	27/982	233/18723	9.63E-05	0.002328	0.001989	27
GO:0010810	cell adhesion	regulation of cell-substrate adhesion	22/982	221/18723	0.00301	0.036396	0.031088	22
GO:0022408	cell adhesion	negative regulation of cell-cell adhesion	21/982	196/18723	0.001517	0.022653	0.019349	21
GO:1903038	cell adhesion	negative regulation of leukocyte cell-cell adhesion	18/982	141/18723	0.000422	0.007705	0.006582	18
GO:0033627	cell adhesion	cell adhesion mediated by integrin	17/982	72/18723	1.32E-07	9.69E-06	8.28E-06	17
GO:0033628	cell adhesion	regulation of cell adhesion mediated by integrin	11/982	48/18723	2.95E-05	0.000897	0.000766	11

GO:0034113	cell adhesion	heterotypic cell-cell adhesion	9/982	61/18723	0.004304	0.047425	0.040508	9

Table S4. gene pairs of causal network with strenth >0.85 and direction>0.5 in immune system process

Cohort with better prognosis			Cohort with poor prognosis		
Source	Target	weight	Source	Target	weight
H4C6	H2BC10	1	CCL3L3	CCL3	1
CXCL3	CXCL2	1	GPR89B	GPR89A	1
MZB1	CD79A	1	STXBP2	MAP2K7	1
TYROBP	FCER1G	1	CCL3	CCL4	1
CXCL10	CXCL11	1	MSH6	MSH2	1
MSH6	MSH2	1	FCER1G	TYROBP	1
GPR183	C5AR1	1	CXCL10	CXCL11	1
HLA-DQA1	HLA-DQB1	1	C4A	C4B	1
C1QA	C1QB	1	S100A8	S100A9	1
HLA-DRB1	HLA-DRA	1	C1S	C1R	1
S100A8	S100A9	1	PDGFRA	EMILIN1	1
C1S	C1R	1	BTN3A3	BTN3A1	1
PDGFRA	EMILIN1	1	HSF1	SCRIB	1
DLG1	PAK2	1	C1QB	C1QC	1
C1QB	C1QC	1	FOS	EGR1	1
H4C9	H2BC12	1	TMEM176A	TMEM176B	1
TMEM176A	TMEM176B	1	MZB1	CD79A	0.995
TAP1	PSMB8	1	CD3E	CD2	0.995
NPLOC4	NUP85	1	HLA-F	HLA-B	0.995
CD3E	CD2	0.995	HLA-DQA1	HLA-DQB1	0.995
DHX58	IFI35	0.99	RNF31	HECTD1	0.995
HSF1	LRRC14	0.99	ITGAL	RASAL3	0.99
PRF1	GZMB	0.985	C1QA	C1QB	0.99
LILRB4	NCF1	0.98	FOS	DUSP1	0.99
MAP2K7	CDC37	0.98	HAVCR2	LAPTM5	0.985
SLAMF7	CXCL9	0.975	OAS1	OAS3	0.985
IGLL5	JCHAIN	0.975	MZB1	IGLL5	0.985
BTN3A3	BTN3A1	0.975	PRF1	GZMB	0.985
DUSP1	FOS	0.975	INHBA	FN1	0.985
KIF14	PARP1	0.97	ZNF683	XCL2	0.98
FBN1	COL3A1	0.97	LAPTM5	C3AR1	0.98
AKIRIN1	MTOR	0.97	CD3E	CD27	0.97
CD84	NCKAP1L	0.965	SUPT6H	MED1	0.97
RSAD2	IFIH1	0.965	HSF1	PTK2	0.97
H2BC10	H4C4	0.96	FLI1	PECAM1	0.965
AIF1	LST1	0.96	SPI1	WAS	0.965
PAF1	ZFP36	0.96	PAF1	CLPTM1	0.96
FOS	EGR1	0.955	HLA-DRB1	HLA-DRA	0.955
SLAMF7	MZB1	0.95	CD93	ADGRF5	0.955
PTPRC	EVI2B	0.95	GBF1	TMEM14C	0.955
C7	INHBA	0.95	SLAMF7	CXCL9	0.955
NCKAP1L	CSF1R	0.945	APBB1IP	GAPT	0.95
SASH3	FERMT3	0.945	FBN1	PDGFRB	0.95
FBN1	CCN4	0.945	FYB1	EVI2B	0.95
HLA-DRB1	HLA-DPA1	0.945	LAT2	TNFAIP8L2	0.945
TYROBP	TREM2	0.94	MX1	OAS3	0.945
CD14	VSIG4	0.94	MTOR	ATAD3A	0.945
OAS1	OASL	0.935	CD86	SAMSN1	0.935
FN1	FBN1	0.935	DUSP1	GPR183	0.935
MX1	RSAD2	0.935	SPI1	FCER1G	0.93
EMILIN1	PDGFRB	0.935	SASH3	FERMT3	0.93
COL3A1	INHBA	0.935	CCN2	DUSP1	0.93
UBE2N	RAP1B	0.93	CCL5	ZNF683	0.925
OAS1	MX1	0.925	RNF31	PSME1	0.925
TLR7	FGR	0.925	H4C6	H4C4	0.925
HLA-A	HLA-B	0.925	GON4L	NCOA6	0.925
HLA-DRB1	CD74	0.925	CXCL11	TNFSF13B	0.92
EGR1	ZFP36	0.925	CD27	MZB1	0.915

CD3D	CD8A	0.92
LAPTM5	PLEK	0.92
MED1	MTOR	0.92
HLA-DMA	HLA-DPB1	0.92
LGALS7B	PAF1	0.91
HAVCR2	CD86	0.905
TLR7	CX3CR1	0.905
PRF1	XCL2	0.905
IFIH1	PARP9	0.905
HLA-DMA	HLA-DRB1	0.9
OAS1	OAS3	0.9
CD86	CLEC7A	0.9
CD93	PDGFRB	0.9
HLA-F	HLA-A	0.895
CD2	CD3D	0.89
CD3E	PRF1	0.89
AIF1	LY96	0.885
DUSP1	ZFP36	0.885
CD86	SAMSN1	0.885
C7	PDGFRA	0.88
CD22	C7	0.88
AP1G1	RC3H2	0.875
RSAD2	IFIT1	0.87
CDC73	KIF14	0.87
BTK	ITGAM	0.865
FN1	INHBA	0.865
LAPTM5	C3AR1	0.865
HAVCR2	EVI2B	0.86
SCRIB	MAP2K7	0.86
LAPTM5	FCER1G	0.855
PTPRC	FYB1	0.85
CXCL2	DUSP1	0.85
RNF8	C7	0.85
RNF8	C7	0.85

AIF1	LST1	0.905
MCM3AP	EIF2AK4	0.905
NCKAP1L	CD84	0.9
HLA-DPA1	HLA-DRA	0.9
FYB1	LCP2	0.895
PARP9	STAT1	0.89
BTK	CLEC4A	0.89
TYROBP	TREM2	0.885
PIBF1	HSF1	0.885
IFIT1	ISG15	0.88
PARP14	PARP9	0.88
PSMB8	PSME1	0.88
MAP2K7	CDC37	0.875
PDGFRB	EMILIN1	0.875
IFIT1	PLSCR1	0.87
INHBA	THBS1	0.865
CD93	SH2B3	0.865
POLB	POLR3F	0.865
COL3A1	LOX	0.86
TAP1	BTN3A3	0.86
MZB1	SLAMF7	0.855
HAVCR2	CD86	0.855
OAS1	IFI35	0.855

Table S5. gene pairs of causal network with strenth >0.85 and direction>0.5 in cell adhesion.

Cohort with better prognosis			Cohort with poor prognosis		
Source	Target	weight	Source	Target	weight
CD3E	CCL5	1	CD3E	CD2	1
HLA-DRB1	HLA-DRA	1	ADAMTS12	COL5A3	1
HLA-DQA1	HLA-DQB1	1	CCN1	CCN2	1
S100A8	S100A9	1	CCN1	DUSP1	1
ITGA11	FBN1	0.995	COL1A1	COL3A1	1
CD86	HAVCR2	0.995	HLA-DQA1	HLA-DQB1	1
POSTN	SFRP2	0.985	S100A8	S100A9	1
RASAL3	ITGAL	0.975	FN1	PLAU	0.995
FERMT3	SASH3	0.975	CD3E	CD27	0.995
FBN1	SNAI2	0.97	POSTN	SFRP2	0.995
SASH3	TNFAIP8L2	0.945	STK4	GSK3B	0.995
TNFAIP8L2	SPI1	0.935	ITGAL	RASAL3	0.99
COL1A1	EMILIN1	0.935	ITGA5	MMP14	0.99
FBN1	EDIL3	0.93	HAVCR2	LAPTM5	0.985
PTPRC	ITGAL	0.93	PTK2	SCRIB	0.98
NID1	EMILIN1	0.93	HLA-DRA	HLA-DRB1	0.98
FERMT3	SPI1	0.92	COL8A1	ITGA11	0.975
HLA-DPB1	HLA-DMA	0.915	CCL5	HLA-A	0.97
SNAI2	NID1	0.91	FBN1	ECM2	0.965
HLA-DRB1	HLA-DMA	0.905	DSG2	PTPN2	0.955
HLA-DRB1	HLA-DPA1	0.905	DUSP1	EGR3	0.945
FBN1	COL1A1	0.895	COL8A1	SERPINE1	0.945
AIF1	VSIG4	0.895	LAPTM5	VSIG4	0.945
NCKAP1L	CD4	0.885	LAPTM5	ITGB2	0.94
ITGA11	THBS1	0.885	SPI1	AIF1	0.94
HLA-DRB1	CD74	0.885	TAOK2	SCRIB	0.935
HLA-DPB1	HLA-DOA	0.87	COL3A1	SNAI2	0.93
ITGB2	SH2B3	0.865	HLA-DPA1	HLA-DRA	0.93
TNFAIP8L2	AIF1	0.86	FERMT3	SASH3	0.925
COL8A1	SFRP2	0.855	CD86	HAVCR2	0.925
AIF1	ARHGDIB	0.855	THBS1	CCN1	0.92
ITGA11	COL8A1	0.85	FN1	ANTXR1	0.91
			SELPLG	CD4	0.905
			HLA-DPA1	HLA-DMA	0.895
			LILRB1	APBB1IP	0.88
			ITGAL	CD3E	0.88
			FERMT3	SPI1	0.88
			ITGA4	SH2B3	0.875
			LILRB1	LILRB2	0.875
			FERMT3	CD300A	0.87
			COL3A1	POSTN	0.87
			TNFAIP8L2	SPI1	0.865
			HLA-DRA	CD74	0.865
			VWF	PECAM1	0.865
			ADAMTS12	PDPN	0.86
			NID1	VWF	0.86
			NCKAP1L	APBB1IP	0.855
			PTPRC	FGL2	0.855
			CD300A	FCGR2B	0.855
			DSG2	CD2	0.855
			NCKAP1L	PTPRC	0.85

Table S6. gene pairs of causal network with strenth >0.85 and direction>0.5 in metabolic process.

Cohort with better prognosis			Cohort with poor prognosis		
Source	Target	weight	Source	Target	weight
TLR7	CX3CR1	1	FOXL2	GATA4	1
H1-5	H1-4	1	DIP2A	MCM3AP	1
CD3E	CD2	1	CD3E	CD2	1
RPL23	RPL19	1	EIF3H	EIF3E	1
OAS3	OAS2	1	S100A8	S100A9	1
CXCL3	CXCL2	1	C4A	C4B	1
FCER1G	TYROBP	1	CCDC22	OTUD5	1
RPL27A	RPLP2	1	CACTIN	FZR1	1
HLA-DRB1	HLA-DPB1	1	CCL3L3	CCL3	1
BTN3A2	BTN3A1	1	RPL23	RPL19	1
TYROBP	TREM2	1	NSD3	ASH2L	1
DDX3X	USP9X	1	CCL3	CCL4	1
RLF	ZMPSTE24	1	DDA1	BABAM1	1
MSH6	MSH2	1	RPL27A	RPS13	1
PARP9	DTX3L	1	PSME2	PSME1	1
CXCL11	CXCL10	1	UBA52	RPL18A	1
EIF3K	ACTN4	1	HSF1	SHARPIN	1
TYK2	CDC37	1	TYROBP	FCER1G	1
RNF168	PAK2	1	MSH6	MSH2	1
UBE2N	CCT2	1	ISG15	IFI6	1
HSF1	SHARPIN	1	PSMB9	PSMB8	1
NPLOC4	SIRT7	0.995	CCN1	CCN2	1
RPL18	RPS11	0.995	PARP9	DTX3L	1
RPL32	RPL15	0.995	RPTOR	NPLOC4	1
RPS11	RPL13A	0.995	SETD1A	TAOK2	1
RPS18	RPS10	0.995	RFC4	ACTL6A	1
ZFP36	EGR1	0.99	CXCL11	CXCL10	1
SNAI2	PDGFRB	0.99	AKAP8	BRD4	1
SIRT7	POLG2	0.985	KRT18	KRT8	1
CD84	NCKAP1L	0.98	IFITM1	IFITM3	1
PTPRC	CCR5	0.98	CD14	VSIG4	0.995
RPL31	RPL23	0.98	YEATS2	EIF4G1	0.995
RPL24	RPS7	0.98	RPS10	RPS18	0.995
POSTN	PDGFRA	0.975	DHPS	RAD23A	0.995
RPL27A	RPS13	0.975	H1-5	H1-4	0.99
AIF1	LY96	0.975	CD27	CD3E	0.99
CD74	HLA-DRB1	0.97	KIF14	TPX2	0.99
HLA-DRB1	HLA-DPA1	0.97	ALS2	TRIP12	0.99
CD14	VSIG4	0.97	SENP5	PAK2	0.99
CSF2RB	CLEC4A	0.965	RPTOR	UBE2O	0.99
LILRB4	NCF1	0.965	TPX2	CDC20	0.99
RPL10A	RPS18	0.965	EIF3G	CDC37	0.985
INHBA	FN1	0.96	PSMB9	HLA-F	0.985
RSAD2	MX1	0.96	PARN	GSPT1	0.985
CCL5	IL2RG	0.96	SPI1	WAS	0.985
RPL31	RPS27	0.955	HLA-F	HLA-B	0.985
LUM	SNAI2	0.95	RPS17	RPLP1	0.985
INHBA	SFRP2	0.945	CDC37	TYK2	0.985
HLA-A	HLA-F	0.945	RPL18	RPS11	0.98
RSAD2	IFIH1	0.945	HAVCR2	LAPTM5	0.975
OAS1	OAS2	0.94	OAS3	OAS2	0.97
MAP2K7	TYK2	0.94	KPNA1	PIK3R4	0.97
POSTN	LUM	0.93	ITGA5	MMP14	0.97
RPL32	RPL14	0.93	PIK3R4	GSK3B	0.97
SCRIB	SHARPIN	0.925	RPL30	EIF3H	0.97
MED1	TERF2	0.92	POSTN	SFRP2	0.965
GZMA	XCL2	0.91	LMTK2	TRRAP	0.965
NCKAP1L	CSF1R	0.91	NCKAP1L	CYBB	0.96

HAVCR2	CD86	0.905
OAS1	OASL	0.9
ZFP36	DUSP1	0.9
SCRIB	UBE2N	0.895
CD86	CLEC7A	0.89
AIF1	FCER1G	0.89
HLA-A	HLA-B	0.89
LUM	ECM1	0.885
C3AR1	VSIG4	0.88
LAPTM5	C3AR1	0.88
CXCL3	SAA1	0.88
CD3E	GZMA	0.875
POLG2	DDX39B	0.87
RPL10A	RPL21	0.865
SIRT7	TICRR	0.865
TLR7	FGR	0.86
INHBA	THBS1	0.86
CD86	SAMSN1	0.86
OAS1	MX1	0.86
GZMA	IL2RB	0.86
GZMA	CCL5	0.86
RPL24	RPL10A	0.86
IFIH1	PARP9	0.855
CD14	AIF1	0.855
WAS	CD4	0.85

INHBA	FN1	0.96
HLA-DPA1	HLA-DPB1	0.96
HSF1	SCRIB	0.96
RPS11	RPL13A	0.96
MX1	LAMP3	0.955
TYROBP	TREM2	0.95
TNFSF13B	CXCL11	0.95
RPL32	RPL29	0.945
CD84	NCKAP1L	0.945
SH2B3	DAB2	0.94
RPL36A	RPL39	0.93
RPS29	RPL35	0.93
RPS18	RPL10A	0.93
H1-5	H1-3	0.925
PTPRC	CSF2RB	0.925
CD86	SAMSN1	0.925
TAOK1	MED1	0.925
CSF2RB	PECAM1	0.92
ERCC4	UBE2K	0.92
COL6A3	SNAI2	0.92
RPL32	RAF1	0.915
COL6A3	ECM1	0.915
COL6A3	PDGFRB	0.915
CCL5	XCL2	0.91
SENP5	SENP2	0.905
PTPN11	DENR	0.905
HLA-F	HLA-A	0.9
KIF14	CASP8AP2	0.895
NCKAP1L	IL10RA	0.895
RPS13	RPL34	0.895
RPL34	LAMTOR3	0.895
AIF1	LY96	0.895
IL10RA	PIK3R5	0.895
ELOB	TRMT112	0.895
CD84	TLR4	0.89
NCKAP1L	TLR7	0.89
ERCC4	GSPT1	0.885
RPL27A	RPS27	0.885
HSF1	PTK2	0.88
RPL15	RPS8	0.88
SETD1B	PTPN11	0.88
NPLOC4	SIRT7	0.88
RECK	DDR2	0.88
RPS17	AKAP13	0.875
UBE2N	DENR	0.875
EIF3G	KEAP1	0.875
NR4A1	EGR1	0.87
HLA-DPB1	HLA-DRB1	0.87
EP400	SETD1B	0.865
KANSL1	PIK3R4	0.865
EGR1	DUSP1	0.865
SERPINE1	SSC5D	0.86
HAVCR2	CD86	0.855
LAPTM5	C3AR1	0.855
CD86	FCGR1A	0.855
IFIH1	SP100	0.855
OAS3	MX1	0.855
NR4A1	OSM	0.855
PSMB9	PSME2	0.855
SENP2	YEATS2	0.855
SERPINF1	LUM	0.855
CD2	CXCL9	0.85

Table S7. gene pairs of causal network with strenth >0.85 and direction>0.5 in cellular component organization.

Cohort with better prognosis			Cohort with poor prognosis		
Source	Target	weight	Source	Target	weight
TLR7	CX3CR1	1	FOXL2	GATA4	1
H1-5	H1-4	1	DIP2A	MCM3AP	1
CD3E	CD2	1	CD3E	CD2	1
RPL23	RPL19	1	EIF3H	EIF3E	1
OAS3	OAS2	1	S100A8	S100A9	1
CXCL3	CXCL2	1	C4A	C4B	1
FCER1G	TYROBP	1	CCDC22	OTUD5	1
RPL27A	RPLP2	1	CACTIN	FZR1	1
HLA-DRB1	HLA-DPB1	1	CCL3L3	CCL3	1
BTN3A2	BTN3A1	1	RPL23	RPL19	1
TYROBP	TREM2	1	NSD3	ASH2L	1
DDX3X	USP9X	1	CCL3	CCL4	1
RLF	ZMPSTE24	1	DDA1	BABAM1	1
MSH6	MSH2	1	RPL27A	RPS13	1
PARP9	DTX3L	1	PSME2	PSME1	1
CXCL11	CXCL10	1	UBA52	RPL18A	1
EIF3K	ACTN4	1	HSF1	SHARPIN	1
TYK2	CDC37	1	TYROBP	FCER1G	1
RNF168	PAK2	1	MSH6	MSH2	1
UBE2N	CCT2	1	ISG15	IFI6	1
HSF1	SHARPIN	1	PSMB9	PSMB8	1
NPLOC4	SIRT7	0.995	CCN1	CCN2	1
RPL18	RPS11	0.995	PARP9	DTX3L	1
RPL32	RPL15	0.995	RPTOR	NPLOC4	1
RPS11	RPL13A	0.995	SETD1A	TAOK2	1
RPS18	RPS10	0.995	RFC4	ACTL6A	1
ZFP36	EGR1	0.99	CXCL11	CXCL10	1
SNAI2	PDGFRB	0.99	AKAP8	BRD4	1
SIRT7	POLG2	0.985	KRT18	KRT8	1
CD84	NCKAP1L	0.98	IFITM1	IFITM3	1
PTPRC	CCR5	0.98	CD14	VSIG4	0.995
RPL31	RPL23	0.98	YEATS2	EIF4G1	0.995
RPL24	RPS7	0.98	RPS10	RPS18	0.995
POSTN	PDGFRA	0.975	DHPS	RAD23A	0.995
RPL27A	RPS13	0.975	H1-5	H1-4	0.99
AIF1	LY96	0.975	CD27	CD3E	0.99
CD74	HLA-DRB1	0.97	KIF14	TPX2	0.99
HLA-DRB1	HLA-DPA1	0.97	ALS2	TRIP12	0.99
CD14	VSIG4	0.97	SENP5	PAK2	0.99
CSF2RB	CLEC4A	0.965	RPTOR	UBE2O	0.99
LILRB4	NCF1	0.965	TPX2	CDC20	0.99
RPL10A	RPS18	0.965	EIF3G	CDC37	0.985
INHBA	FN1	0.96	PSMB9	HLA-F	0.985
RSAD2	MX1	0.96	PARN	GSPT1	0.985
CCL5	IL2RG	0.96	SPI1	WAS	0.985
RPL31	RPS27	0.955	HLA-F	HLA-B	0.985
LUM	SNAI2	0.95	RPS17	RPLP1	0.985
INHBA	SFRP2	0.945	CDC37	TYK2	0.985
HLA-A	HLA-F	0.945	RPL18	RPS11	0.98
RSAD2	IFIH1	0.945	HAVCR2	LAPTM5	0.975
OAS1	OAS2	0.94	OAS3	OAS2	0.97
MAP2K7	TYK2	0.94	KPNA1	PIK3R4	0.97
POSTN	LUM	0.93	ITGA5	MMP14	0.97
RPL32	RPL14	0.93	PIK3R4	GSK3B	0.97
SCRIB	SHARPIN	0.925	RPL30	EIF3H	0.97
MED1	TERF2	0.92	POSTN	SFRP2	0.965
GZMA	XCL2	0.91	LMTK2	TRRAP	0.965
NCKAP1L	CSF1R	0.91	NCKAP1L	CYBB	0.96
HAVCR2	CD86	0.905	INHBA	FN1	0.96
OAS1	OASL	0.9	HLA-DPA1	HLA-DPB1	0.96
ZFP36	DUSP1	0.9	HSF1	SCRIB	0.96
SCRIB	UBE2N	0.895	RPS11	RPL13A	0.96

CD86	CLEC7A	0.89
AIF1	FCER1G	0.89
HLA-A	HLA-B	0.89
LUM	ECM1	0.885
C3AR1	VSIG4	0.88
LAPTM5	C3AR1	0.88
CXCL3	SAA1	0.88
CD3E	GZMA	0.875
POLG2	DDX39B	0.87
RPL10A	RPL21	0.865
SIRT7	TICRR	0.865
TLR7	FGR	0.86
INHBA	THBS1	0.86
CD86	SAMSN1	0.86
OAS1	MX1	0.86
GZMA	IL2RB	0.86
GZMA	CCL5	0.86
RPL24	RPL10A	0.86
IFIH1	PARP9	0.855
CD14	AIF1	0.855
WAS	CD4	0.85

MX1	LAMP3	0.955
TYROBP	TREM2	0.95
TNFSF13B	CXCL11	0.95
RPL32	RPL29	0.945
CD84	NCKAP1L	0.945
SH2B3	DAB2	0.94
RPL36A	RPL39	0.93
RPS29	RPL35	0.93
RPS18	RPL10A	0.93
H1-5	H1-3	0.925
PTPRC	CSF2RB	0.925
CD86	SAMSN1	0.925
TAOK1	MED1	0.925
CSF2RB	PECAM1	0.92
ERCC4	UBE2K	0.92
COL6A3	SNAI2	0.92
RPL32	RAF1	0.915
COL6A3	ECM1	0.915
COL6A3	PDGFRB	0.915
CCL5	XCL2	0.91
SENP5	SENP2	0.905
PTPN11	DENR	0.905
HLA-F	HLA-A	0.9
KIF14	CASP8AP2	0.895
NCKAP1L	IL10RA	0.895
RPS13	RPL34	0.895
RPL34	LAMTOR3	0.895
AIF1	LY96	0.895
IL10RA	PIK3R5	0.895
ELOB	TRMT112	0.895
CD84	TLR4	0.89
NCKAP1L	TLR7	0.89
ERCC4	GSPT1	0.885
RPL27A	RPS27	0.885
HSF1	PTK2	0.88
RPL15	RPS8	0.88
SETD1B	PTPN11	0.88
NPLOC4	SIRT7	0.88
RECK	DDR2	0.88
RPS17	AKAP13	0.875
UBE2N	DENR	0.875
EIF3G	KEAP1	0.875
NR4A1	EGR1	0.87
HLA-DPB1	HLA-DRB1	0.87
EP400	SETD1B	0.865
KANSL1	PIK3R4	0.865
EGR1	DUSP1	0.865
SERPINE1	SSC5D	0.86
HAVCR2	CD86	0.855
LAPTM5	C3AR1	0.855
CD86	FCGR1A	0.855
IFIH1	SP100	0.855
OAS3	MX1	0.855
NR4A1	OSM	0.855
PSMB9	PSME2	0.855
SENP2	YEATS2	0.855
SERPINF1	LUM	0.855
CD2	CXCL9	0.85

Table S8. gene pairs of causal network with strenth >0.85 and direction>0.5 in response to stimulus.

Cohort with better prognosis			Cohort with poor prognosis		
Source	Target	weight	Source	Target	weight
PDGFRA	EMILIN1	1	STAR	GATA4	1
FAP	LRRC15	1	CCL3L3	CCL3	1
CXCL3	CXCL2	1	RPTOR	NPLOC4	1
IGLL5	JCHAIN	1	CCL3	CCL4	1
PARP9	DTX3L	1	HLA-F	HLA-B	1
RSAD2	CMPK2	1	PARP9	DTX3L	1
GPR183	C5AR1	1	CDH5	VWF	1
EID2	PAF1	1	CXCL11	CXCL10	1
COL4A1	COL4A2	1	GPR183	OSM	1
HLA-DRB1	CD74	1	S100A8	S100A9	1
CXCL11	CXCL10	1	SENP2	PAK2	1
S100A8	S100A9	1	PTPN11	ATP2A2	1
DLG1	PAK2	1	HSF1	SLC52A2	1
HSF1	LRRC14	1	ISG15	IFI6	1
OAS2	OAS3	1	AP1G1	IST1	1
FOS	DUSP1	1	CHMP7	CCAR2	1
AP1G1	IST1	1	CHD8	PRMT5	1
SAP30BP	NUP85	1	COL1A1	COL5A1	1
IFI44L	IFI44	1	SMARCA4	ILF3	1
FCER1G	TYROBP	1	IFITM1	IFITM3	1
USP9X	DDX3X	1	TYROBP	FCER1G	1
IFIT3	IFIT2	1	ASPM	TOP2A	1
TLR7	CX3CR1	0.995	AKAP8	BRD4	1
CSF1R	STAB1	0.995	FN1	PLAU	1
HGS	NPLOC4	0.995	CCN1	CCN2	1
TOP2A	FAM83D	0.995	EGR1	FOS	1
COL1A2	COL1A1	0.99	COL5A2	FAP	0.995
XCL2	PRF1	0.985	RSAD2	CMPK2	0.995
HAVCR2	CD86	0.985	HSF1	SHARPIN	0.995
DHX58	IFI35	0.985	SPI1	WAS	0.995
EMILIN1	ADGRA2	0.98	CD14	VSIG4	0.995
IFI44L	IFI6	0.98	IFIT3	IFIT2	0.995
RRAGC	AKIRIN1	0.975	MED1	TAOK1	0.99
HSF1	SHARPIN	0.97	IFI44L	IFI44	0.99
CD14	VSIG4	0.97	CDC37	EIF3G	0.99
PTPRC	CCR5	0.965	IFIT1	IFIT3	0.99
HLA-F	HLA-A	0.965	MRC1	F13A1	0.985
TNFAIP8L2	SPI1	0.96	CD209	MRC1	0.98
ISG15	IFI6	0.96	HAVCR2	LAPTM5	0.98
AKIRIN1	MTOR	0.96	CCNK	AREL1	0.975
AIF1	LY96	0.955	OAS3	OAS2	0.975
CREB3L1	COL6A1	0.945	ZRANB1	CCNK	0.975
TYROBP	LY86	0.945	STK4	STAU1	0.975
OAS1	OAS2	0.94	ADGRA2	PDGFRB	0.97
FGR	TLR7	0.94	EIF3G	PIN1	0.97
FGR	ITGAX	0.94	CXCR3	CD27	0.96
PPIE	ZMPSTE24	0.94	GSK3B	KPNA1	0.96
RSAD2	IFI44L	0.935	NCKAP1L	CYBB	0.955
PAK2	OPA1	0.935	HSF1	SCRIB	0.955
FAM83D	NUP85	0.93	TLR7	NCKAP1L	0.95
MTOR	MED1	0.93	SLAMF8	CXCL9	0.95
IFIT1	IFIT3	0.93	HLA-DRB1	CD74	0.95
CD86	CLEC7A	0.925	ILF3	CDC37	0.95
CCR5	CXCR3	0.925	DACT3	HIC1	0.945
HLA-A	HLA-B	0.925	PTPRC	CSF2RB	0.945
DUSP1	ZFP36	0.925	MTOR	ATAD3A	0.945
STAB1	SIGLEC1	0.92	CCL5	XCL2	0.945
FOS	ZFP36	0.91	HIC1	EMILIN1	0.94
FBN1	COL5A2	0.905	PDGFRA	DCN	0.935
AP1G1	RAB14	0.905	DACT3	DACT1	0.935
FBN1	TIMP3	0.9	NCKAP1L	IL10RA	0.935
LAPTM5	C3AR1	0.9	DAB2	SH2B3	0.935

C3AR1	RNASE6	0.9	CMPK2	HERC5	0.93
DUSP1	NR4A1	0.895	MED1	LAMTOR5	0.93
SIGLEC10	AOAH	0.895	POSTN	SFRP2	0.925
CXCL9	SLAMF8	0.895	EGR3	NR4A1	0.915
PDGFRB	COL4A1	0.89	CDH5	COL4A1	0.915
ACTA2	SERPINF1	0.89	FOS	DUSP1	0.91
NCKAP1L	CSF1R	0.89	LY86	TYROBP	0.905
COL1A1	COL3A1	0.89	CD74	HLA-DPA1	0.905
AP1G1	VPS35	0.885	AIF1	LY96	0.905
PRF1	CCL5	0.885	PTPN11	DENR	0.9
RRAGC	PPIE	0.875	CXCL16	MINK1	0.9
CDH5	PDGFRB	0.875	COL3A1	SPARC	0.9
FBN1	SPARC	0.875	HLA-F	HLA-A	0.9
LAPTM5	CTSS	0.87	COL3A1	MMP2	0.895
MAP2K7	CDC37	0.87	COL5A2	COL3A1	0.89
BTK	NCKAP1L	0.865	IL10RA	PIK3R5	0.885
HGS	CDC37	0.865	ILF3	AKAP8	0.885
AIF1	FCER1G	0.865	CXCL11	GBP4	0.885
ALOX5AP	CLEC5A	0.865	LY86	TREM2	0.885
FAM83D	PBK	0.86	RPL13A	RPS15A	0.885
LRRC15	ECM1	0.855	HAVCR2	CD86	0.88
ADGRA2	CDH5	0.855	FAP	ADAMTS12	0.875
AIF1	CD14	0.855	MNDA	FGL2	0.875
COL3A1	POSTN	0.855	LAPTM5	C3AR1	0.875
PAF1	ZFP36	0.855	HIC1	ADGRA2	0.875
OASL	OAS1	0.85	OAS3	MX1	0.87
MPEG1	CSF2RB	0.85	HSF1	PTK2	0.87
LRRC15	ADAMTS12	0.85	COL1A1	COL6A1	0.87
HLA-B	HLA-DRB1	0.85	MX1	LAMP3	0.87
NR4A1	ZFP36	0.85	DUSP1	CCN1	0.87
VPS35	PAF1	0.85	LILRB1	SIGLEC10	0.865
			SELPLG	CD4	0.865
			ZEB2	DAB2	0.855
			KRAS	OTUD5	0.855
			THBS1	CCN1	0.855
			KPNA1	PARP9	0.85
			SPI1	CD14	0.85

Table S9. 49 different chemotherapy node genes

ID	SYMBOL
1	AIF1
2	CCN1
3	HLA-DRB1
4	FBN1
5	COL1A1
6	RPS27
7	H1-5
8	HLA-DRA
9	AP1G1
10	COL3A1
11	INHBA
12	COL6A3
13	HTT
14	C1QB
15	CDC37
16	SPI1
17	DUSP1
18	HIC1
19	RPL10A
20	HLA-F
21	C7
22	RPL24
23	C1QA
24	FAM83D
25	HSF1
26	SIRT7
27	ITGA11
28	ILF3
29	NCKAP1L
30	KIF2C
31	RPS15
32	TNFAIP8L2
33	CD3E
34	IFI44L
35	PIK3R4
36	MZB1
37	NCBP1
38	FERMT3
39	LRRC15
40	GZMA
41	PSMB9
42	LAPTM5
43	RSF1
44	PAF1
45	LUM
46	ZFP36
47	OAS1
48	PRF1
49	RSAD2

Table S10. 346 Chemotherapy expanded feature genes

1	MZB1	51	LILRB1	101	CCL5	151	ZNF683
2	SPI1	52	IL10RA	102	AEBP1	152	COL1A2
3	LY86	53	PLEK	103	KPNA1	153	COL8A1
4	CD79A	54	HLA-DPB1	104	POSTN	154	DACT3
5	FCER1G	55	SLAMF7	105	DAB2	155	CIAO2B
6	LST1	56	CCR5	106	BTN3A2	156	MMP19
7	TYROBP	57	MPEG1	107	IFIT1	157	OASL
8	FCGR2B	58	IKZF1	108	HERC5	158	LUM
9	LY96	59	HLA-DRB1	109	DUSP1	159	ITGA4
10	IGLL5	60	VSIG4	110	SAA1	160	STK4
11	TNFAIP8L2	61	NCKAP1L	111	COL3A1	161	COL1A1
12	RNASE6	62	STAB1	112	AOAH	162	EMILIN1
13	FCGR1A	63	CD84	113	CREB3L1	163	SERPINE1
14	TREM2	64	HLA-DPA1	114	CLEC7A	164	HSF1
15	C1QA	65	RASAL3	115	SH2B3	165	RRAGC
16	GMFG	66	SIGLEC1	116	BOP1	166	MX1
17	AIF1	67	CXCR3	117	CD74	167	BRD4
18	JCHAIN	68	FERMT3	118	HLA-B	168	COL6A3
19	GAPT	69	C5AR1	119	DTX3L	169	SLC52A2
20	CD14	70	HLA-DMA	120	FLI1	170	COL10A1
21	ABI3	71	FGR	121	CD2	171	PSME2
22	C3AR1	72	ITGB2	122	IFIT3	172	LAMTOR5
23	C1QB	73	LOX	123	GSK3B	173	LRRC15
24	C1QC	74	PTPRC	124	SHARPIN	174	CMPK2
25	CD86	75	IL2RG	125	ITGA11	175	PARP9
26	APBB1IP	76	PIK3R5	126	MMP2	176	OAS1
27	LAT2	77	CLEC4A	127	SNAI2	177	COL5A2
28	TLR7	78	SFRP2	128	FIS1	178	CD8A
29	FYB1	79	SIGLEC10	129	PSMB8	179	XCL2
30	HCK	80	ITGAL	130	DCN	180	SPARC
31	EVI2B	81	CSF2RB	131	INHBA	181	FBN1
32	WAS	82	SLAMF8	132	RSAD2	182	SERPINF1
33	BIN2	83	ITGAM	133	CD3E	183	LRRC14
34	FGL2	84	ITGAX	134	HIC1	184	EIF3K
35	MNDA	85	ECM1	135	HLA-A	185	CTSK
36	LILRB2	86	ISG15	136	IFI6	186	CXCL9
37	BTK	87	HLA-F	137	TIMP3	187	SP100
38	SELPLG	88	PIK3R4	138	COL5A1	188	CD93
39	HAVCR2	89	OSM	139	ZFP36	189	BTN3A1
40	CD300A	90	CD3D	140	FAP	190	IFI44L
41	LCP2	91	HLA-DRA	141	CCN4	191	PECAM1
42	CSF1R	92	IFIT2	142	COL11A1	192	STAU1
43	SASH3	93	ZEB2	143	HLA-DOA	193	ELOB
44	LAPTM5	94	PSMB9	144	CRISPLD2	194	SETD1A
45	CD4	95	IFI35	145	FN1	195	TRMT112
46	CYBB	96	ACTA2	146	RNF8	196	CD22
47	CTSS	97	TLR4	147	SSC5D	197	PDPN
48	CX3CR1	98	ARHGDIB	148	ATAD3A	198	IL2RB
49	GPR183	99	MMP11	149	ADAMTS12	199	SCRIB
50	SAMSN1	100	CD27	150	ECM2	200	DHX58

201	PLSCR1	251	RNF31	301	CSNK1D
202	CLPTM1	252	PAF1	302	AKIRIN1
203	COL6A1	253	STAT1	303	HGS
204	EDIL3	254	ADGRA2	304	EP400
205	FOS	255	MRPL58	305	SIRT7
206	CXCL2	256	NPLOC4	306	UBE2N
207	PDGFRB	257	CCN2	307	KEAP1
208	CCN1	258	GZMB	308	CENPA
209	PDGFRA	259	CDC20	309	RAD54L
210	DACT1	260	C7	310	IST1
211	THBS1	261	HECTD1	311	UTP4
212	EIF3G	262	STXBP2	312	COL4A2
213	PSME1	263	TPX2	313	TYK2
214	IFIH1	264	SUPT6H	314	TLK2
215	TAP1	265	CENPX	315	RAB14
216	IFI44	266	ZMPSTE24	316	MAP2K7
217	LGALS7B	267	MED1	317	VPS35
218	LSG1	268	SENP5	318	ATP2A2
219	COL5A3	269	SETD1B	319	DLG1
220	ANTXR1	270	ATXN1L	320	CASP8AP2
221	PAK2	271	LAMB1	321	RLF
222	PLAU	272	RAP1B	322	TAOK1
223	EIF4G1	273	NR4A1	323	DDX39B
224	OAS3	274	PARP1	324	SPTY2D1
225	AEBP2	275	ADGRF5	325	UBE2O
226	EGR3	276	SENP2	326	KRI1
227	OAS2	277	KANSL1	327	AP1G1
228	OPA1	278	COL4A1	328	TOP2A
229	PRF1	279	MTOR	329	GATA4
230	SUPT4H1	280	PTPN11	330	PIBF1
231	LMOD1	281	DENR	331	SMARCA4
232	LAMP3	282	RPTOR	332	ILF3
233	RNF168	283	CDC73	333	TICRR
234	TAOK2	284	TUBGCP6	334	NUP85
235	VWF	285	PTK2	335	RACGAP1
236	BTN3A3	286	ACTN4	336	DSG2
237	EGR1	287	YEATS2	337	ASPM
238	NID1	288	FOXL2	338	RC3H2
239	GZMA	289	SAP30BP	339	CLNS1A
240	CDC37	290	STAR	340	BPTF
241	RECQL4	291	POLG2	341	BUB1
242	NIP7	292	FAM83D	342	PBK
243	EID2	293	NCAPH	343	KIF14
244	CDH5	294	PTPN2	344	CENPF
245	PIN1	295	TERF2	345	RSF1
246	PPAN	296	USP36	346	EMSY
247	EBNA1BP2	297	CXCL3		
248	PPIE	298	AKAP8		
249	ESAM	299	CCT2		
250	PARP14	300	KIF2C		

Fig S1. Multivariate Cox regression analysis of GSE17260

Characteristics	Total(N)	HR (95% CI)	P value
CBI	110	0.304 (0.111 - 0.830)	0.02
stage	110		
IIIa	6	Reference	
IIIb	18	0.559 (0.100 - 3.128)	0.508
IIIc	69	0.603 (0.116 - 3.145)	0.548
IV	17	0.833 (0.158 - 4.386)	0.83
grade	110		
G1	26	Reference	
G2	41	1.622 (0.658 - 4.003)	0.294
G3	43	2.641 (1.051 - 6.637)	0.039

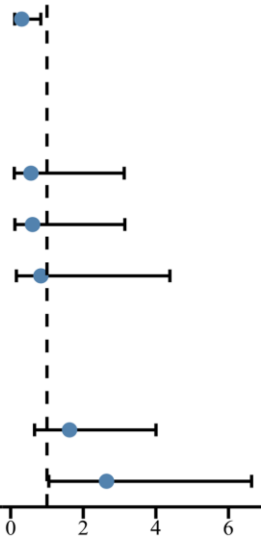

Fig S2. Multivariate Cox regression analysis of GSE26193

Characteristics	Total(N)	HR (95% CI)	P value
CBI	107	0.412 (0.201 - 0.844)	0.015
stage	107		
I	20	Reference	
II	11	1.841 (0.541 - 6.270)	0.329
IIIa	2	3.143 (0.358 - 27.556)	0.301
IIIb	3	4.654 (1.112 - 19.481)	0.035
IIIc	54	5.484 (2.146 - 14.018)	< 0.001
IV	17	10.213 (3.562 - 29.284)	< 0.001
grade	107		
G1	7	Reference	
G2	33	1.879 (0.415 - 8.503)	0.413
G3	67	1.024 (0.224 - 4.690)	0.975

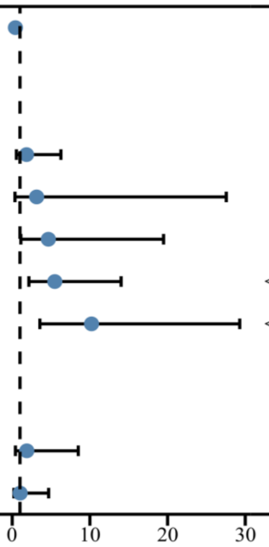

Fig S3. Multivariate Cox regression analysis of GSE30161

Characteristics	Total(N)	HR (95% CI)	P value
CBI	58	0.443 (0.263 - 0.747)	0.002
age	58	0.994 (0.959 - 1.030)	0.741
race	58		
White	54	Reference	
Black	4	1.196 (0.411 - 3.480)	0.742
histo	58		
Serous	47	Reference	
Clear	5	0.209 (0.028 - 1.533)	0.124
Transitional	1	0.000 (0.000 - Inf)	0.867
Undiff	1	0.000 (0.000 - Inf)	0.917
Unknown	2	0.466 (0.062 - 3.480)	0.457
Mucinous	1	0.000 (0.000 - Inf)	0.004
Endometrioid	1	0.000 (0.000 - Inf)	1
grade	58		
G1	2	Reference	
G2	19	1.408 (0.663 - 2.988)	0.373
G3	33	2.690 (1.315 - 5.502)	0.007
GX	4	2.997 (0.407 - 22.055)	0.281
stage	58		
IIIC	38	Reference	
IIIB	11	1.181 (0.447 - 3.122)	0.737
IIIA	4	0.000 (0.000 - Inf)	0.795
IVA	5	1.760 (0.595 - 5.208)	0.307

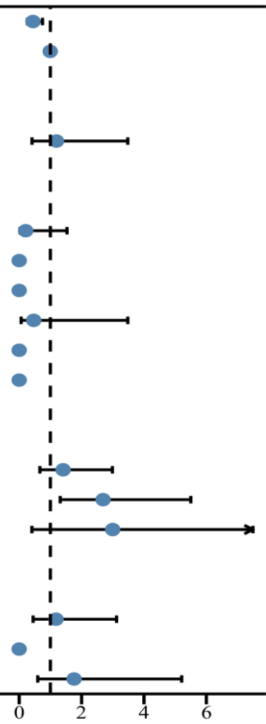

Fig S4. Multivariate Cox regression analysis of GSE32062

Characteristics	Total(N)	HR (95% CI)	P value
CBI	260	0.360 (0.211 - 0.615)	< 0.001
grade	260		
G2	131	Reference	
G3	129	1.103 (0.761 - 1.598)	0.606
stage	260		
IIIa	4	Reference	
IIIb	20	0.662 (0.133 - 3.288)	0.614
IIIc	180	1.083 (0.264 - 4.433)	0.912
IV	56	1.439 (0.342 - 6.063)	0.62

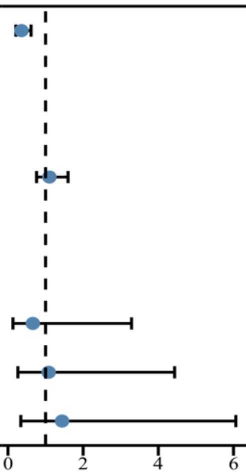

Supplement: S1 File — (PDF) [file pone.0322130.s001.pdf]
